# Surviving host - and food relevant stresses: phenotype of *L*. *monocytogenes* strains isolated from food and clinical sources

**DOI:** 10.1038/s41598-018-30723-z

**Published:** 2018-08-28

**Authors:** Jule Anna Horlbog, David Kent, Roger Stephan, Claudia Guldimann

**Affiliations:** 1 Institute for Food Safety and Hygiene, Vetsuisse Faculty, University of Zurich, Zurich, Switzerland; 2 Department of Food Science, Cornell University, Ithaca, NY United States

## Abstract

The aim of this study was to compare the phenotype of 40 strains of *L*. *monocytogenes* under food and host relevant stress conditions. The strains were chosen to represent food and clinical isolates and to be equally distributed between the most relevant clonal complexes for clinical and food isolates (CC1 and CC6 vs CC121 and CC9), plus one group of eight strains of rare clonal complexes. Human-associated CC1 had a faster maximal growth rate than the other major complexes, and the lag time of CC1 and CC6 was significantly less affected by the addition of 4% NaCl to the medium. Food-associated CC9 strains were hypohemolytic compared to other clonal complexes, and all strains found to be resistant to increased concentrations of benzalkonium chloride belonged to CC121 and were positive for Tn6188 carrying the *qacH* gene. Lactic acid affected the survival of *L*. *monocytogenes* more than HCl, and there was a distinct, strain specific pattern of acid tolerant and sensitive strains. Strains from CC6 and human clinical isolates are less resilient under acid stress than those from other complexes and from food. One strain isolated from a human patient exhibited significant growth defects across all conditions.

## Introduction

The severe clinical conditions resulting from food-borne infections with *Listeria monocytogenes* are a constant concern to public health agencies and food producers. The European Food Safety Authority reported 2‘161 confirmed cases of human listeriosis in 2014, which accounts for a notification rate of 0.52 cases per 100‘000 people^1^. Although the incidence level is low, the high hospitalization rate and a case fatality rate ranging from 15–30%^2–4^ rank listeriosis among the most threatening foodborne illnesses.

The ubiquitous nature and the wide genetic variability of *L*. *monocytogenes* have inspired efforts to identify markers of virulence by analyzing and comparing the compositions of strain populations found in clinical patients, the food production context and the natural environment^5,6^. The underlying aim across studies to determine the virulence potential of a given strain has been (i) to assess the level of risk associated with a strain found in the food chain and (ii) to gain deeper insight into the mechanisms of virulence^7,8^. *L*. *monocytogenes* are divided into four deeply separated evolutionary lineages^9^ and 13 serotypes^10^. Even though all *L*. *monocytogenes* are considered pathogenic, the vast majority of human clinical cases can be attributed to a narrow set of serotype 1/2a, 1/2b and 4b strains^11,12^. Further subtyping by multilocus sequence typing (MLST) identified up to six epidemic clones of genetically related strains, isolated from geographically and temporally distinct outbreaks^13–15^. Recently, Maury *et al*.^16^ analyzed the relative prevalence of different clonal complexes (CC) in a large dataset of 6633 *L*. *monocytogenes* strains isolated from human clinical cases and food. CC1, CC2, CC4 and CC6 strains showed the highest relative frequency in clinical cases, while CC121 and CC9 strains were mostly found in food. The same authors described that host factors played a role in infections with strains that are not typically associated with clinical cases. For example, CC121 or CC9 strains were found more frequently in patients with clinical comorbidities^16^. This may explain why an infrequent number of strains with mutations in important virulence factors are found in human patients^6,17,18^. Moura *et al*.^15^ identified groups of highly related strains responsible for multistate outbreaks and showed that sub lineages of *L*. *monocytogenes* were repeatedly spread internationally.

Based on the data provided by Maury *et al*.^16^, we hypothesized that the skew in relative frequencies of CC1 and CC6 towards clinical cases might be due to the fact that they are able to grow faster in food and that their defense mechanisms against host barriers such as hydrochloric acidity in the stomach might be superior to that of strains from CC121 or CC9.

We characterized a set of 40 *L*. *monocytogenes* isolates for their response to food associated stresses (preservatives like salt and lactic acid, and the antimicrobial benzalkonium chloride (BC)) as well as attributes relevant in the host environment (resistance to HCl, hemolytic activity as an approximation for the expression rate of virulence genes). The strain set was chosen to be equally distributed between food and clinical isolates as well as CC1, CC6, CC121 and CC9, plus an additional set of nine strains to represent rare isolates not commonly found in clinical cases or the food environment.

## Results

### Growth in BHI

**Figure 1:**
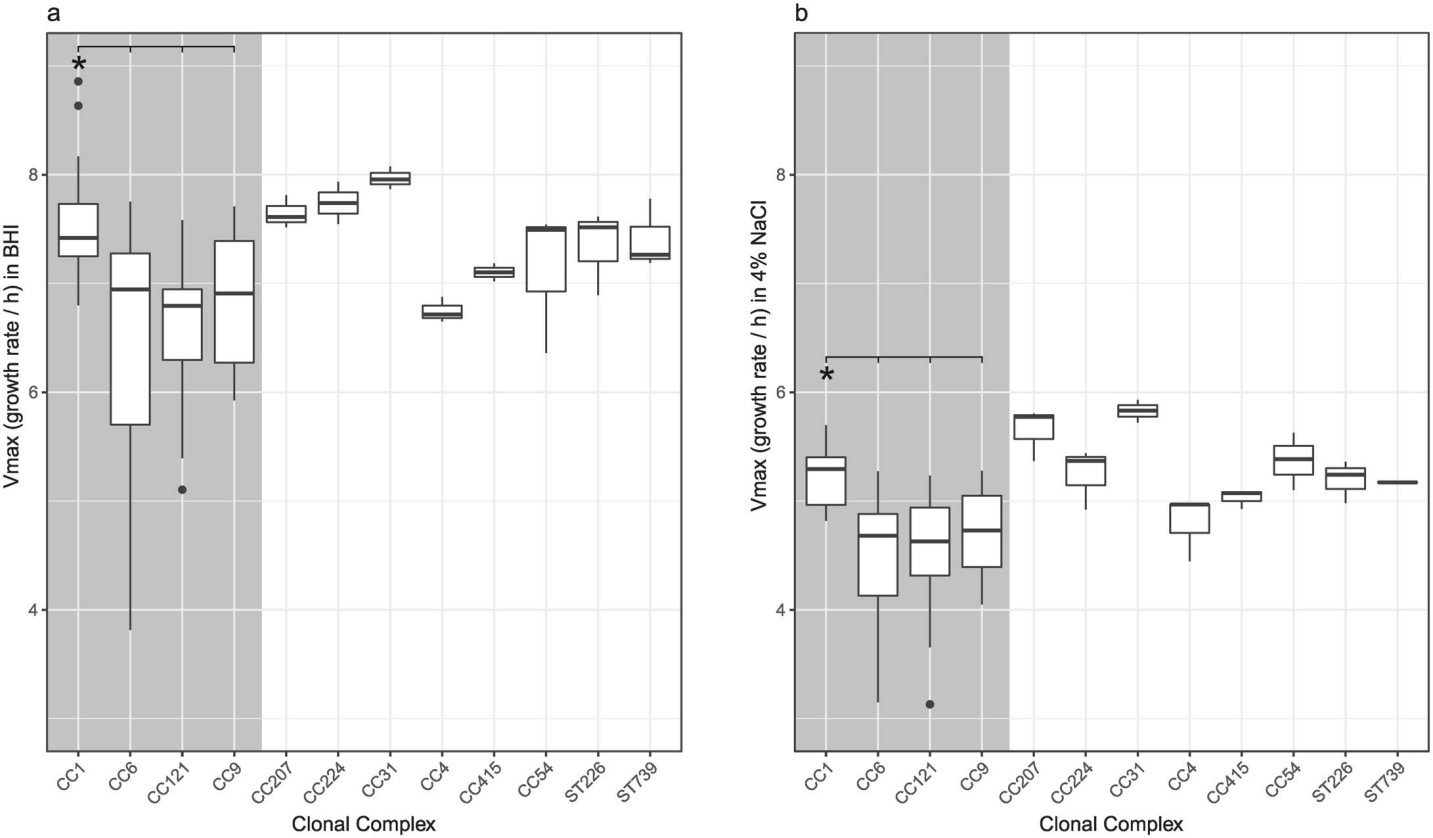
Maximal growth rate. The y axis represents the maximum growth rate (defined as the maximum slope calculated by a sliding window approach) in BHI (**a**) and BHI with 4% NaCl (**b**) plotted against clonal complex. The shaded area denotes the four larger CC included in the study. The white area contains CC which are represented by only one strain. Boxplots represent the mean, 25th and 75th quantile with 1.5*IQR whiskers. Dots represent outliers and *denotes a significant contribution of CC to the phenotype.

Growth characteristics of every strain were measured in BHI in order to create a baseline to which growth under stress can be compared to. All strains were grown in BHI at 37 °C and the maximal growth rate (Vmax) as well as the lag time were determined. The mean Vmax in BHI was 6.98 (SD = 0.8). There was no meaningful difference in Vmax between strains from different lineages or serotypes. CC1 showed a significantly higher Vmax (7.54, SD = 0.49) than CC6, CC121 and CC9 (combined mean Vmax 6.66, SD = 0.81; p < 0.001) (Fig. 1a). The highest Vmax was observed in strain N12-1996 (CC1, serotype 4b, food isolate; 8.1, SD = 0.5), the slowest Vmax was in strain N12-1387 (CC6, serotype 4b, human clinical isolate; 3.86, SD = 0.05).

The average lag time in BHI was 124 min (SD = 15 min). There was no meaningful difference in lag time between strains from different lineages, serotypes or clonal complexes. Isolates from food had a marginally shorter lag time (120.5 min, SD = 18.7) than isolates from human clinical cases (129.6 min, SD = 19.7), p = 0.002. The shortest lag time was in strain N13-0703 (CC6, serotype 4b, food isolate; 108.8 min, SD = 0.005) and the longest lag time was observed in strain N12-1387 (CC6, serotype 4b, human clinical isolate; 166 min, SD = 0.0004).

### Growth in BHI supplemented with 4% NaCl

When the BHI was supplemented with 4% w/v NaCl, the average Vmax (4.85, SD = 0.51, p < 0.0001) was significantly slower than without the added salt. At this slower growth rate, the pattern observed for Vmax across strains remained unchanged. There was no meaningful difference in Vmax between lineages and serotypes, while CC1 showed significantly faster growth (5.23, SD = 0.25) compared to CC6, CC121 and CC9 (combined mean Vmax 4.6, SD = 0.44, p < 0.001) (Fig. 1b). The fastest Vmax was observed in strain N13-0228 (CC31, serotype 1/2a, food isolate; 5.83, SD = 0.11), the slowest Vmax in strain N12-1387 (CC6, serotype 4b, human clinical isolate; 3.24, SD = 0.09).

When cells were grown in BHI supplemented with 4% NaCl compared to the growth in BHI, the average lag times significantly increased to 167 min (SD = 20.1), p < 0.001. Strains from lineage I showed a significantly shorter lag time (156 min, SD = 15.0) than strains from lineage II (176 min SD = 19.4), p < 0.001. No significant difference in lag times was observed between serotypes, and strains from CC1 had significantly shorter lag times (151.5 min, SD = 7.7) than CC9 and CC121 (combined mean lag time175.3 min, SD = 20.9) (p < 0.001). The shortest lag time in BHI with 4% NaCl was observed in strain N12-1339 (CC1, serotype 4b, food isolate; 143.7 min, SD = 0.005) and the longest lag time in BHI with 4% NaCl was for strain N11-1837 (CC9, serotype 1/2a, human clinical isolate; 237 min, SD = 0.01).

To differentiate strains that are generally fast growers (e.g. strain N13-0228 (CC31, serotype 1/2a, food isolate) which had a high Vmax in BHI as well as in BHI supplemented with NaCl) from strains that adapt faster than others to an increase in NaCl in the medium, relative values for Vmax (Vmax in 4% NaCl/Vmax in BHI) and lag time (lag time in BHI supplemented with 4% NaCl/lag time in BHI) were calculated.

Overall, Vmax decreased by a factor of 0.70 (SD = 0.05) in BHI with the added salt compared to BHI. No significant differences in relative Vmax was observed between lineages, serotypes or clonal complexes, indicating that Vmax was a property of the strain regardless of the two media tested here. The highest relative Vmax was observed for strain N12-1387 (CC6, serotype 4b, human clinical isolate; 0.84, SD = 0.02), the lowest for strain N12-0710 (CC9, serotype 1/2c, food isolate; 0.60, SD = 0.02).

**Figure 2:**
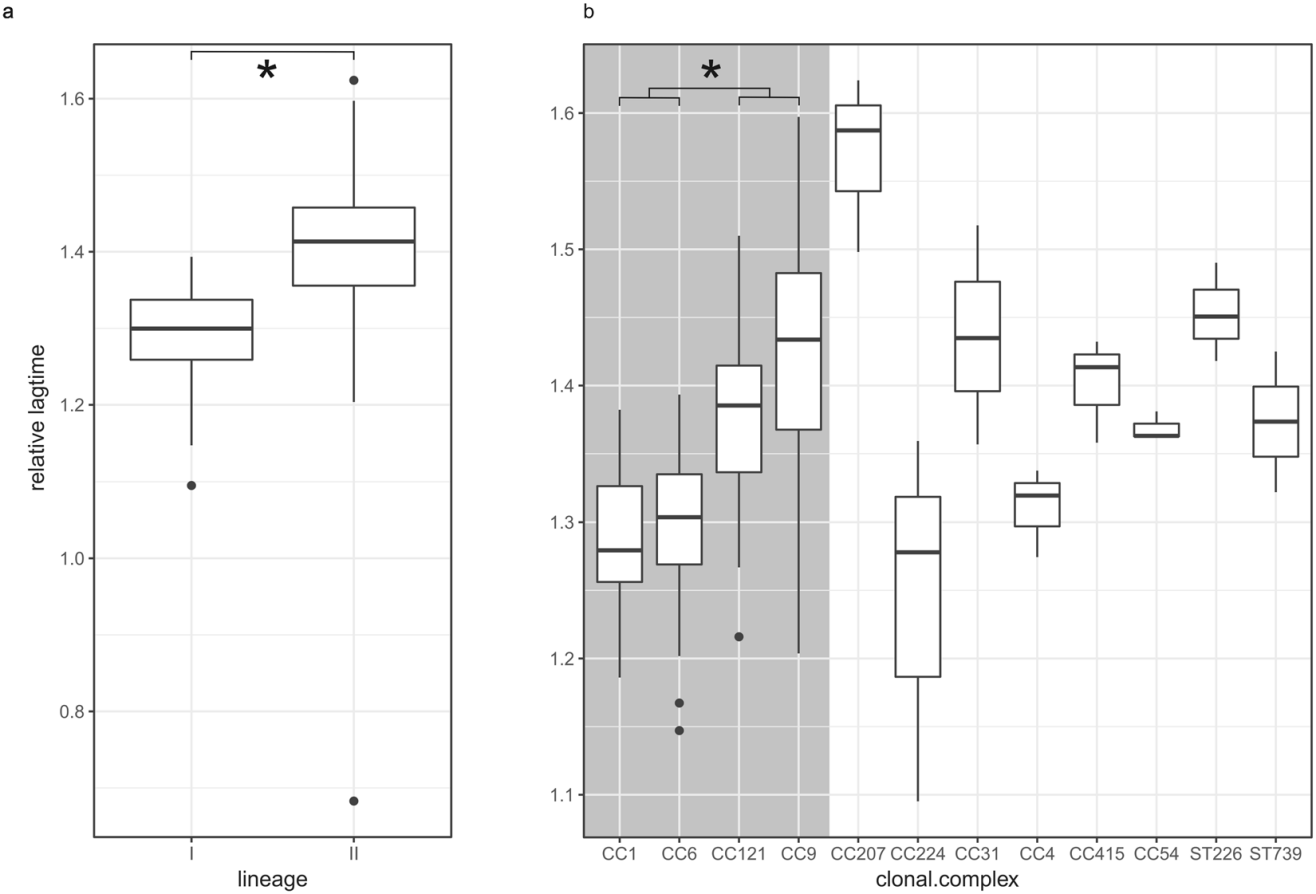
Relative lag time. The y axis represents the relative lag time (lag time in BHI supplemented with 4% NaCl/lag time in BHI), plotted against lineage (**a**) and clonal complex (**b**). The shaded area denotes the four larger CC included in the study. The white area contains CC which are represented by only one strain. Boxplots represent the mean, 25th and 75th quantile with 1.5*IQR whiskers. Dots represent outliers and *denotes a significant contribution of the qualifier to the phenotype.

The relative lag times indicate the factor by which the lag time increased upon the addition of 4% NaCl. The lag times increased less in strains that belong to lineage I (1.29, SD = 0.0.04) than in strains from lineage II (1.39, SD = 0.08, p < 0.001) (Fig. 2a). CC1 and CC6 had significantly shorter relative lag times than CC121 and CC9 (p < 0.01 for all contrasts) (Fig. 2b). Nested F-tests showed that while serotype explained a significant amount of variance in relative lag-time by itself, most of that variance was explained by clonal complex since adding serotype to the clonal complex model did not yield a significantly better model fit. The highest relative lag time was observed for strain N12-1107 (CC207, serotype 1/2a, human clinical isolate; 1.57, SD = 0.06), the lowest for strain N11-2542 (ST739, serotype 1/2a, food isolate; 1.03, SD 0.40).

### Acid stress

**Figure 3:**
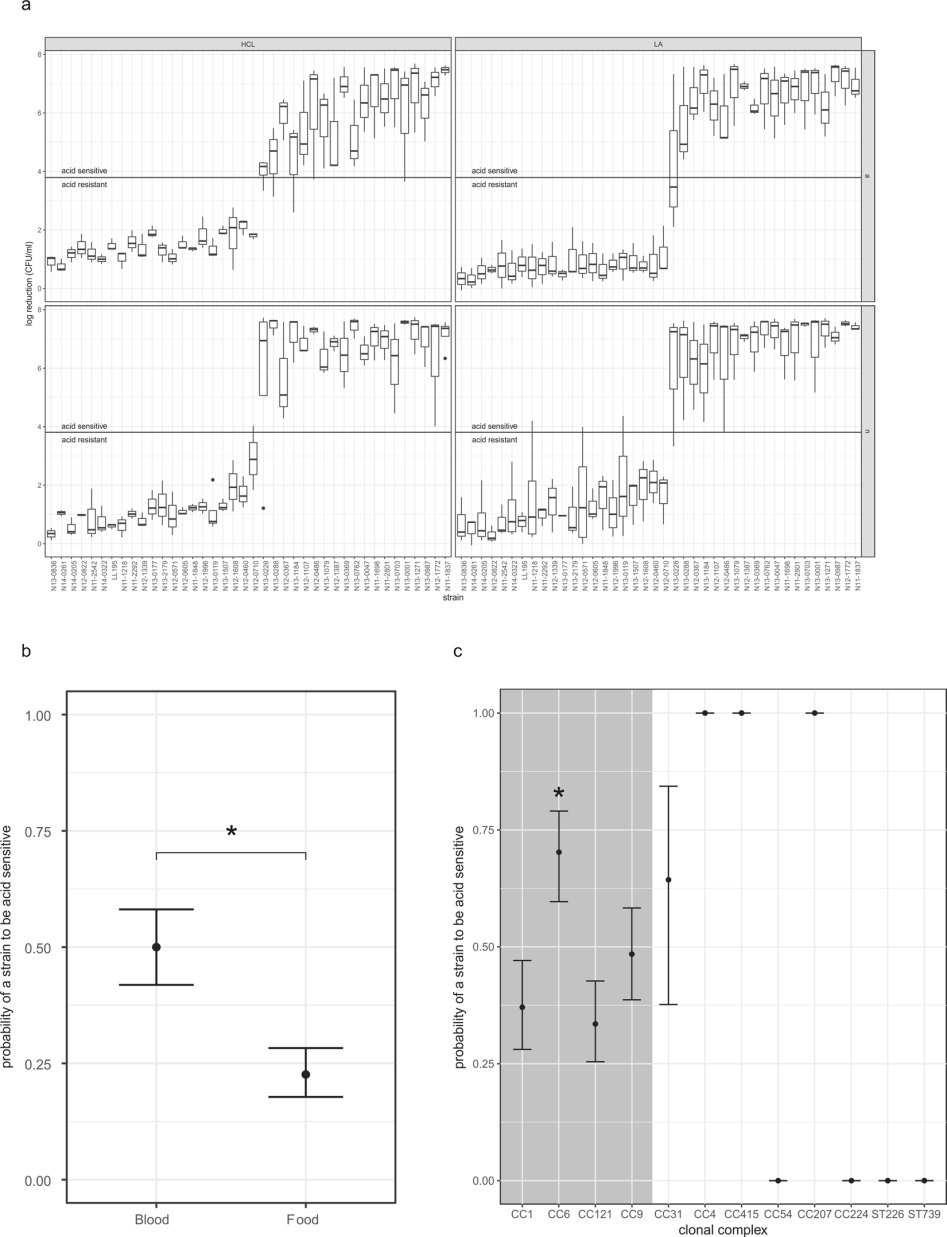
(**a**) Log reduction of CFU/ml in individual strains between before and after 1 h acid exposure at pH3 for HCl, pH3.5 for LA. The upper panel represents data from strains that were pre-exposed to mild acid stress at pH5.5 for one hour prior to exposure the acid stress. The lower panel represents data from acid-shocked strains without prior adaptation to pH5.5. The horizontal line represents the delineation between “acid sensitive” or “acid resistant” phenotypes as determined by k-means. (**b**) Probabilities of belonging to the “acid sensitive” phenotype by source. **(c)** Probabilities of belonging to the “acid sensitive” phenotype by clonal complex. The shaded area denotes the four larger CC included in the study. The white area contains CC which are represented by only one strain. CCs with collapsed error bars had only acid-sensitive or acid-resistant observations, making a realistic estimate of the variance impossible. *Denotes a significant association of the qualifier with the phenotype.

The response of *L*. *monocytogenes* to inorganic (hydrochloric acid, HCl) and organic (lactic acid, LA) acid stress at 37 °C was determined with and without a prior adaptation step at an intermediate pH to determine the ability of the strains to survive an acid shock as well as their adaptive acid tolerance. The results are presented as the log reduction between before and after one hour incubation at the final pH (Fig. 3a).

Interestingly, there was a distinct pattern of acid-resistant and acid-sensitive strains that seemed mainly driven by properties of the individual strains. This is reflected in the large standard deviations within the dataset. The mean log reduction resulting from exposing *L*. *monocytogenes* to one hour of HCl stress at pH 3.0 was 3.84 (SD 2.99) with previous adaptation to pH 5.5 and 3.58 (SD 2.49) for acid shock. Initial experiments with LA showed that *L*. *monocytogenes* did not survive exposure to LA at pH 3.0 for an hour (data not shown). We therefore increased the pH to 3.5 in order to measure inter-strain differences in LA acid survival. In general, the results obtained with LA at pH 3.5 mirror the patterns obtained with HCl at pH 3.0 (Fig. 3a). The mean log reduction resulting from exposing *L*. *monocytogenes* to one hour of LA stress at pH 3.5 was 3.51 (SD 3.01) with previous adaptation to pH 5.5 and 3.77 (SD 3.00) for acid shock.

We used k-means to assign binary phenotypes of “acid resistant” and “acid sensitive” to each individual strain. Given that the large variance between strains overrode the effect of acid adaptation in individual strains, adaptation was not a good predictor of acid susceptibility. Neither lineage nor serotype were significantly associated with either the acid-susceptible or acid-resistant phenotype. Strains isolated from food had a significantly lower probability to be “acid sensitive” than strains isolated from human clinical cases (p < 0.001, Fig. 3b). Clonal complex was a significant predictor of acid susceptibility with CC6 having a slightly higher chance to fall into the “sensitive” category (p < 0.0001, Fig. 3c). For CCs with only one strain it was not possible to differentiate between the effect of CC and the strain itself to get a realistic estimate of the variance (e.g. CC54, CC224, ST226 and ST375 had only acid-resistant observations, while CC4, CC415 and CC207 had only acid-susceptible observations).

### Resistance to benzalkonium chloride

**Figure 4:**
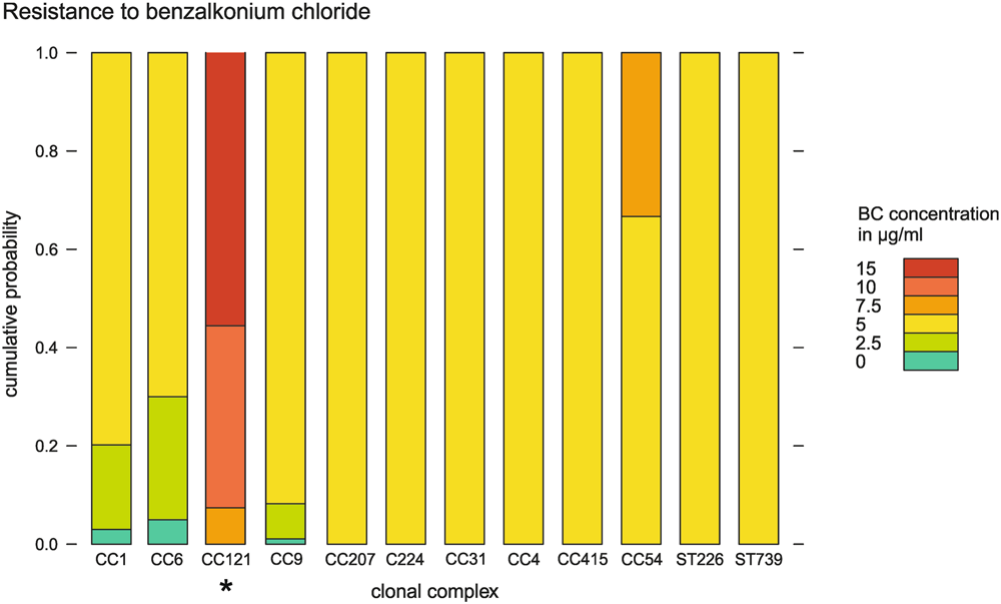
The concentration of benzalkonium chloride (μg/ml) to which strains were resistant plotted against clonal complex. The y-axis represents the probability of a strain to be resistant to each concentration of BC. *Denotes CCs that were significantly associated with the phenotype.

The majority of strains included in this study (31/40) was resistant to 5 μg/mL BC or lower (Fig. 4). Strains were considered BC resistant if they were able to grow at double the concentration that inhibited growth in >50% of all strains, e.g. at 10 μg/mL^19,20^.

Strains from lineage II were more likely to be more highly resistant than strains from lineage I (p < 0.001). This was likely due to the underlying effect of the clonal complex, as CC121 was resistant to significantly higher concentrations of BC (median = 15 μg/mL BC), compared to the other clonal complexes (median = 5 μg/mL BC, p < 0.001) (Fig. 4). While all CC121 strains were resistant to high concentrations of BC; strain N12-1387 (CC6, serotype 4b, human clinical isolate) was the most sensitive (median = 0 μg/mL BC).

PCR revealed that all CC121 strains were positive for Tn6188 that carried *qacH*, an efflux pump for BC^21^. None of the strains carried any of the other known efflux pumps such as *emrE*^22^ or *bcrABC*^23^.

### Hemolysis

We analyzed the hemolytic activity of the supernatants of all 40 strains. The hemolytic activity is a direct function of the amount of listeriolysin O (LLO) a strain secretes. LLO is encoded by *hly* under the control of the major transcriptional regulator for virulence genes, PrfA, and can be used as an approximation for the virulence of a given strain.

**Figure 5:**
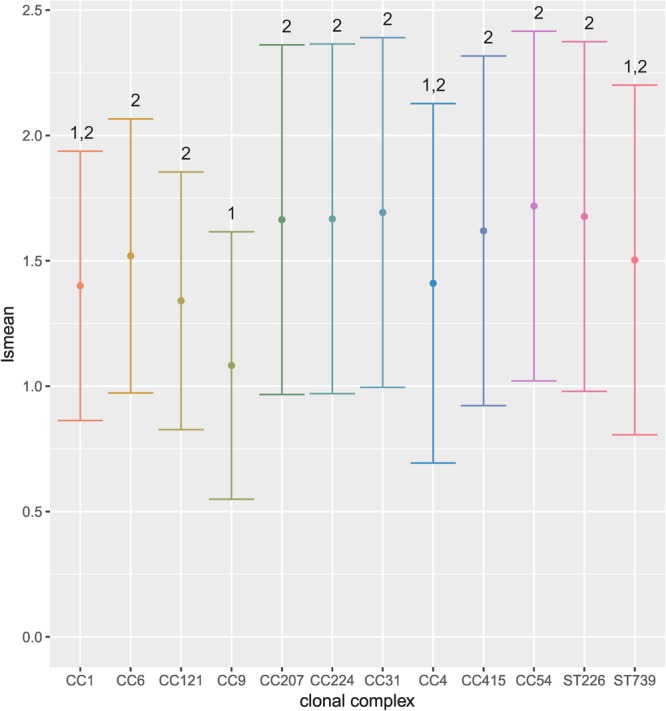
Hemolysis. The y-axis represents the amount of hemolysis caused by the supernatant of each strain, as determined by a mixed effects linear model, plotted against clonal complex. The dots represent the least square means, the whiskers the upper and lower 95% confidence level. CCs that share a number above the bars are not significantly different from each other, as determined by a false discovery rate of <0.05.

There was no significant difference in hemolytic activity between lineages. Strains belonging to serotype 1/2c had significantly lower hemolytic activity compared to 1/2a, 1/2b and 4b (p = 0.01). CC9 strains caused significantly less hemolysis compared to other CCs (Fig. 5). The highest hemolysis was observed for strain N13-0369 (CC121, serotype 1/2a, food isolate; 1.85, SD = 0.3), the lowest for strain N12–1387 (CC6, serotype 4b, human clinical isolate; 0.96, SD = 0.05).

## Discussion

We found that lineage I strains (comprising CC1 and CC6 and also serotype 4b) recover faster after exposure to 4% NaCl in the medium and CC1 had a higher Vmax compared to CC121 and CC9 in both BHI supplemented with NaCl and BHI. In combination, this might increase the chance of a CC1 strain to reach high numbers in food faster than strains from other clonal complexes and might in part explain why CC1 strains are among the most frequently isolated in human clinical cases^16^. This is in line with other authors who found that serotype 4b (lineage I) strains had a shorter lag time than serotype 1/2a (lineage II) strains in 11% NaCl grown at 10 °C^24^. However, in the data provided in Magalhães *et al*.^25^, lineage seems not to be a significant predictor of lag time in TSBYE with 4% NaCl as determined by a t-test on the data in Table 2 of their data (t = −0.13138, p-value = 0.9).

Resistance to acid is a relevant factor in the ability of a given *L*. *monocytogenes* strain to reach a human host and result in an infection. Lactic acid is an important preservative in the food industry, and is often added to meat or fish in the form of potassium or sodium lactate. In addition, lactic acid is naturally present in dairy products through fermentation. The relatively common use of a low pH as a preservative measure in food may exert a selective pressure that explains the higher likelihood of food isolates to be acid tolerant. Interestingly, human clinical isolates were significantly less resilient under acid stress. The low pH in the mammalian stomach is achieved by the secretion of HCl and is an important “first line of defense” mechanism against pathogens entering the body. *L*. *monocytogenes* is capable of surviving acidic conditions as low as pH 2.5 for 2 h^26^. In our initial experiments, BHI at pH 3 was used for the HCl as well as the LA assays, resulting in less than 100 CFU/ml (limit of detection) surviving bacteria after LA stress. We therefore concluded that *L*. *monocytogenes* was more sensitive to LA than to HCl, which is in line with literature on the adaptive acid tolerance in *L*. *monocytogenes* which was more effectively induced by LA than HCl^27,28^. We found a distinct pattern of acid tolerant and acid sensitive strains that did not consistently align with any of the classification systems according to lineage, serotype, or CC of the strains. Throughout the literature on acid tolerance of *L*. *monocytogenes*, a strain-dependent survival pattern is discernable. For instance, Lianou *et al*.^29^ challenged different strains of *L*. *monocytogenes* with LA at pH 3.5, and a strain specific pattern that did not follow serotype or origin designation is visible in the data. Strains grown on food matrices for different time intervals were challenged with pH 1.5 (HCl) and the survival pattern was strain dependent^30^. After 2h of exposure to pH 2.4 achieved with HCl, Lundén *et al*.^31^ observed a variability in log reductions between strains that exceeds 6 log. A strain specific pattern of acid resistance is also visible in the datasets presented by Ramalheira *et al*.^32^ and Cheng *et al*.^28^. Mobile genetic elements such as plasmids, transposons or insertion sequences are likely candidates to explain phenotypic patterns that refuse to align with any classification systems derived from the genome such as lineage or CC. It is tempting to speculate their involvement in the pattern observed here and in other studies. Bacteria faced with the challenge of an increased concentration of H+ ions have the options of (i) consuming H+ ions through decarboxylation reactions, (ii) exporting H+ ions through pumps and (iii) repairing the damage resulting from low pH, for instance mediated by heat shock proteins. All these mechanisms are growth-phase and temperature dependent^27^, have been described for *L*. *monocytogenes* and were reviewed by Ryan, Hill & Gahan^33^. Any of the above mechanisms could be encoded on mobile genetic elements.

We found that all strains with an increased resistance to BC belonged to CC121 and harbored the Tn1688 transposon that carries the *qacH* gene encoding an efflux pump^21^, which goes along with previous findings^34,35^. While strains coding for *bcrABC* are commonly found in Switzerland^34,35^, we found no strains carrying these genes in the panel of strains used in this study. Not surprisingly, we did not find any strains carrying the *emrE* gene; this efflux pump for BC has so far only been demonstrated in strains from Canada^22^ and in one strain originating from Finland^34^.

The hemolytic activity was used as an indicator of virulence for a given strain. Differences in hemolytic activity between strains may indicate differences in PrfA activity as the main transcriptional activator of *hly*, or in the post-transcriptional regulation of LLO. *hly* mutants are unable to escape phagocytic vacuoles^36^, are avirulent *in vivo*^37^, and the controlled regulation of LLO production is directly linked to virulence *in vivo*^38^. However, while LLO encoded by *hly* is the main hemolysin of *L*. *monocytogenes*, a variety of genes is known to influence the hemolytic activity of a strain, which should caution against using LLO activity as the only explanation for phenotypic differences in hemolysis^39,40^. Hypohemolytic mutants occur at low frequencies in *L*. *monocytogenes* belonging to CC9 and CC121 as well as CC1 and CC6, as was shown in a screening of 57820 strains^40^. All 60 hypohemolytic strains found in that study carried mutations in the *prfa-hly* regulation pathway. In this context, it is interesting that strains from CC9, which are mostly isolated from the food environment, showed the lowest hemolytic activity and might partially explain why strains from CC9 are less frequently isolated from human clinical cases. On the other hand, CC121 that is also mainly associated with the food environment had significantly higher hemolytic activity compared to CC9.

Across the board, we found little association of the phenotype with the source a strain was isolated from (clinical vs food). This indicates that the genotype rather than the environment a strain is isolated from dictates its ability to grow under the tested conditions, a finding that is confirmed by other authors as well^24^.

Among the panel of 40 strains used in this study, strain N12-1387 (CC6, serotype 4b, human clinical isolate) stood out in several assays: it was the strain with the lowest tolerance to BC (<2.5 mg/ml), had the lowest hemolytic activity, by far the lowest Vmax in BHI with and without 4% added NaCl, and belonged to the group of acid sensitive strains. Interestingly, this strain was isolated from blood in a human clinical case. No further information is available on the patient or his treatment, but it is conceivable that there were considerable comorbidities involved to result in invasive listeriosis by a strain with such substantial growth impairments.

In conclusion, CC1 had a high maximal growth rate, CC1 and CC6 strains recovered faster after the addition of 4% NaCl to the medium and CC1 and CC6 showed higher hemolytic activity than CC9 strains, which may provide them with a growth advantage in food and increased virulence in human hosts. The molecular mechanism of the distinct pattern of acid resistance is subject to further investigation.

## Materials and Methods

### Bacterial Strains and Growth Conditions

Forty *L*. *monocytogenes* strains were selected out of two existing strain collections described by Ebner *et al*.^35^ and Althaus *et al*.^6^ (Table 1). The strains were isolated either from food or human patients and collected at the Swiss National Reference Laboratory (NENT) between 2011 and 2014. The selection comprised eight strains of each CC1, CC9, CC121, and seven strains of CC6. An additional group consisted of nine rare strains^35^ with less than nine isolates recorded at the Listeria MLST database hosted by the Institute Pasteur (http://bigsdb.web.pasteur.fr/listeria/). If available, equal numbers of strains isolated from food and clinical sources were included. Where the strain collection contained more than the desired number of strains with given parameters, Google’s random number generator (accessed in March 2017) was used to facilitate the strain selection.

**Table 1:** Strains used in this study.

Strain ID	Clonal Complex	Sequence type	Source	serotype	Lineage
N12-0605	CC1	727	Food	4b	I
N12-1339	CC1	746	Food	4b	I
N12-1996	CC1	1	Food	4b	I
N13-0047	CC1	1	Food	4b	I
N11-2292	CC1	1	Human blood	4b	I
LL195	CC1	3	Human blood	4b	I
N13-0987	CC1	1	Human blood	4b	I
N13-1079	CC1	1	Human blood	4b	I
N11-1218	CC121	108	Food	1/2a	II
N12-0571	CC121	755	Food	1/2a	II
N13-0369	CC121	121	Food	1/2a	II
N13-0836	CC121	121	Food	1/2b	II
N14-0205	CC121	121	Food	1/2a	II
N14-0322	CC121	121	Food	3c	II
N12-0367	CC121	121	Human blood	1/2a	II
N13-0119	CC121	121	Human blood	1/2a	II
N12-1107	CC207	207	Human blood	1/2a	II
N12-1608	CC224	224	Human blood	1/2b	I
N13-0228	CC31	748	Food	1/2a	II
N12-1772	CC4	6	Food	4b	I
N13-0762	CC415	683	Human blood	1/2b	II
N13-0177	CC54	54	Human blood	4b	I
N12-0460	CC6	6	Food	4b	I
N13-0703	CC6	6	Food	4b	I
N13-1184	CC6	6	Food	4b	I
N11-2801	CC6	6	Human blood	4b	I
N12-1387	CC6	6	Human blood	4b	I
N13-1271	CC6	6	Human blood	4b	I
N13-1507	CC6	6	Human blood	4b	I
N11-1698	CC9	9	Food	1/2c	II
N12-0710	CC9	477	Food	1/2c	II
N12-0822	CC9	751	Food	1/2c	II
N11-1848	CC9	9	Food	1/2c	II
N14-0261	CC9	9	Food	1/2c	II
N11-1837	CC9	9	Human blood	1/2a	II
N12-0486	CC9	9	Human blood	1/2c	II
N13-0001	CC9	9	Human blood	1/2c	II
N13-2179	ST226	307	Food	1/2a	II
N13-0288	ST28	CC121	Food	1/2a	II
N11-2542	ST739	739	Food	1/2a	II

Bacterial strains were stored in brain heart infusion (BHI) (Oxoid, Pratteln, Switzerland) broth and 15% glycerol (Sigma-Aldrich Chemie GmbH, Buchs, Switzerland) at −80 °C. Fresh cultures were obtained for each experiment by streaking frozen stock on BHI agar followed by overnight incubation at 37 °C. Single colonies were inoculated into five ml BHI broth and incubated at 37 °C shaking (200 rpm) for 15–18 h. These overnight cultures were then sub cultured (1:100) into fresh BHI broth and grown for six hours at 37 °C with shaking to an early stationary phase (corresponding to a culture grown to OD_590_ 1.0 plus 1 h, 1–3 × 10^9^ colony forming units (CFU)/ml). Where indicated, strains were adjusted to equal an OD_590_ of 1.5 in fresh BHI. All experiments were carried out in triplicates.

### Growth rate in rich medium

Early stationary phase cultures were prepared and adjusted to the same OD_590_. Five μl of each of the adjusted cultures were inoculated into 200 μl BHI in a 96 well plate in triplicate. The plates were incubated for 12 h at 37 °C with continuous shaking in an EON microplate reader (BioTek, Lucerne, Switzerland). OD_600_ was measured every 15 min. Data acquisition (lag phase, maximal growth rate and OD_600_ max) and visualization was done using the proprietary software Gen5 All-In-One Microplate Reader Software (Version 2.01.14, BioTek, Lucerne, Switzerland).

### Growth under 4% salt stress

Early stationary phase cultures were prepared and adjusted to the same OD_590_. Triplicate 5 μl aliquots of each strain were transferred into 200 μl of BHI broth with 4% (w/v) in a 96 well plate. The plates were incubated for 12 h at 37 °C with continuous shaking in an EON microplate reader (BioTek, Lucerne, Switzerland). Data acquisition was the same as described above for the growth rate in rich medium.

### HCl Acid Stress Assay

Acid resistance at pH3 with and without prior acid adaptation was tested as previously described by Ferreira *et al*.^41^ with minor modifications. The pH of the BHI was adjusted with hydrochloric acid solution (1 M HCL) (Sigma-Aldrich Chemie GmbH, Buchs, Switzerland) immediately prior to the experiment. Aliquots of 1 ml of early stationary phase cultures were transferred to Eppendorf tubes and centrifuged for 5 min at 6000 × g. For acid shock experiments, the pellets were resuspended in 1 ml BHI pH 3 and incubated for 1 h at 37 °C without shaking. For acid adaptation experiments, 1 ml of the same culture were pelleted, resuspended in 1 ml BHI adjusted to pH 5.5 and incubated for 1 h at 37 °C without shaking. Then, the culture was centrifuged again, resuspended in BHI pH 3 and incubated for 1 h at 37 °C without shaking. Bacteria were enumerated by direct colony count from serial dilutions before and after the one-hour incubation at pH 3. Serial dilutions were performed in 96-well plates in BHI (pH 7.4). 10 μl of each well were spotted onto the edge of a BHI agar plate and run over the plate by tilting as described by Jordan *et al*.^10^. Plates were incubated at 37 °C for 18 h prior to performing colony counts.

### Organic Acid Stress Assay

DL-Lactic acid ~90% (LA, Sigma-Aldrich Chemie GmbH, Buchs, Switzerland) was used as a second acid stress. The experimental procedure was carried out in the same way as described above, apart from the final pH being 3.5 instead of 3. This change in final pH was determined after preliminary experiments showed no growth after incubation at pH 3 in LA in an effort to expose the strains to the most selective possible acid stress.

### Benzalkonium Chloride Resistance

BC susceptibility was tested as previously described by Meier *et al*.^34^. Early stationary phase cultures were prepared. All cultures were adjusted to the same OD_590_ and 10 μl of the adjusted cultures were spotted onto BHI agar plates containing increasing BC (Sigma-Aldrich Chemie GmbH, Buchs, Switzerland) concentrations (0, 2.5, 5, 7.5, 10, 15, 20, 25 and 30 μg/ml). The strains could either show (i) no growth, (ii) growth of single colonies or (iii) confluent growth in the spotted area after incubation at 37 °C for 48 h. Strains were considered resistant to the concentration of BC where confluent growth was observed. To further characterize resistant strains PCR analysis for resistance genes (*bcrABC*, *Tn6188*, *emrE*) was carried out with primers and conditions described by Meier *et al*.^34^.

### Hemolysis Assay

The hemolytic activity in the supernatant of all *L*. *monocytogenes* strains was measured similarly to the method previously described by Schärer *et al*.^42^. Human blood (blood type A+) was obtained from Blutspende Zürich, Zürich, Switzerland. The erythrocytes were washed twice by centrifugation (5 min, 500 × g) and resuspension in phosphate buffered saline (PBS, pH 7.4, Sigma, Buchs, Switzerland). Early stationary phase cultures of *L*. *monocytogenes* were obtained as described above. The OD_590_ of the cultures was measured and 4 ml were centrifuged for 10 min at 10 000 × g, 4 °C. The supernatants were diluted in BHI to account for differences in OD_590_ of the cultures. The adjusted supernatants were sterile filtered and 5 μl dithiotreitol (DTT Sigma, Buchs, Switzerland) were added to 995 μl of the supernatant. After one hour of incubation at 37 °C, 100 μl of the supernatants were mixed with an equal amount of 0.05% washed human erythrocytes in a 96-well round bottom plate in triplicate technical replicates. After 40 minutes of incubation at 37 °C, the plate was centrifuged for 5 min at 4300 × g and 100 μl of the supernatants were transferred into a new 96-well plate. The OD at 540 nm was measured with a Microplate reader (Synergy HT, BioTek, Lucerne, Switzerland). Supernatant from washed erythrocytes mixed with DTT reduced BHI served as a blank. In every run, *L*. *monocytogenes* 10403S prfA* (G145S^43^, and *ΔprfA* mutants^44^ served as positive and negative controls, respectively.

### Statistical analysis

All statistical analysis was performed in R (Version 3.4.2)^45^ using Studio (Version 1.1.383)^46^. All figures were produced using the ggplot2 package^47^.

Medians of Vmax and lag times in BHI were compared using Kruskal-Wallis with Dunn’s post hoc tests as the data did not satisfy the assumptions of a linear model. For the relative growth rates and relative lag times in BHI with 4% NaCl linear models were calculated. Contrasts were calculated using lsmeans^48^. Model selection was done by nested F-tests.

The acid data did not satisfy the assumptions of a linear model, and exploratory graphs made a clear clustering into two groups apparent. We therefore used k-means^49^ clustering to group the observations into two clusters and then modelled the probabilities of an observation falling into a cluster using logistic regressions.

BC resistance was modelled using an ordinal regression with polr in MASS^50^. The six categories of BC concentration were considered in order (0 < 2.5 < 5 < 7.5 < 10 < 15) and an effect for the probability of a subject to be in the next higher category was fitted.

The hemolysis data was analyzed with linear mixed effect models (lmer in LmerTest^51^) using the OD_540_ in the supernatant as the dependent variable and lineage, serotype, clonal complex, and source of the strain as fixed effects. To hedge for differences in different batches of human blood obtained at different days, “date” was used as random effect. Lsmeans^48^ was used to create contrasts and p-values were corrected for multiple comparisons using a false discovery rate of 0.05. Since there were generally no differences in phenotype between strains isolated from food vs clinical sources, they are only discussed in the results section where source was identified as a predictor of the phenotype.

All statistical analysis can be obtained as supplementary materials.

## Electronic supplementary material

Supplementary information

## Data Availability

All data generated or analyzed during this study are included in this published article (and its Supplementary Information files).
