# Supplementary material for: Surviving host - and food relevant stresses: phenotype of L. monocytogenes strains isolated from food and clinical sources
Source: Sci Rep. 2018 Aug 28;8:12931. doi: 10.1038/s41598-018-30723-z (PMC6113203; doi:10.1038/s41598-018-30723-z)
Supplement: Supplementary file 1 — Supplementary information [file 41598_2018_30723_MOESM1_ESM.pdf]

Supplementary files

Surviving host- and food relevant stresses: phenotype of *L. monocytogenes* strains isolated from food and clinical sources

Jule Anna Horlbog[1], David Kent[2], Roger Stephan[1], Claudia Guldimann [1*]

[1] Institute for Food Safety and Hygiene, Vetsuisse Faculty, University of Zurich, Switzerland

[2] Department of Food Science, Cornell University, Ithaca, NY, United States

* address correspondence to: Claudia Guldimann, Institute for Food Safety and Hygiene, Vetsuisse Faculty, University of Zurich, Switzerland; claudia.guldimann@uzh.ch

BHI/Salt

David Kent (dk657)

1/6/2018

Contents

Spreadsheet notes **2**
Method: 2
Readout: 2
Questions: 2
A second set of questions we can ask this dataset: 2
questions: 2

The data **3**
Huge ugly table 3

Relative Lag-Time Models **6**
Plots 6
Clonal Complex 10
Diagnostic plots 10
Model summary 12
ANOVA 12
Pairwise comparisons 13
Serotype 14
Diagnostic plots 14
Model summary 16
ANOVA 16
Pairwise comparisons 16
Lineage 17
Diagnostic plots 17
Model summary 19
ANOVA 19
Pairwise comparisons 19
Source 20
Diagnostic plots 20
Model summary 22
ANOVA 22

Relative Vmax **22**
Plots 22
Clonal Complex 27
Diagnostic plots 27
Model summary 29
ANOVA 29
Serotype 29
Diagnostic plots 29
Model summary 31
ANOVA 31
Lineage 31
Diagnostic plots 31
Model summary 33

ANOVA 33
Source 33
Diagnostic plots 33
Model summary 35
ANOVA 35

Spreadsheet notes

What are the lag-time and Vmax after you add NaCl to the medium? E.g., in a food context, are there strains that adapt faster to salt stress than others?

Method:

Make log phase culture. Dilute the same culture either in BHI or in BHI with 4% NaCl. Use a plate reader to measure OD every 15 minutes over 12 h. So there is now a growth curve for growth with and without the salt that we can compare. Three biological replicates with three technical replicates each.

Readout:

The platereader software calculated the lagtime and Vmax. Each replicate is the mean of 3 wells. The lagtime numbers represent hh:mm:ss. The Vmax I think represents slope. It was calculated by a sliding window approach that determines the maximum slope in the growth curve, this much I am sure of. With the relative values we account for the fact that certain strains will grow faster than others regardless of the medium. e.g. a strain might have a long lag time in BHI, but also in salt. That does not make it a slow adapter to salt, just a generally slow grower.

We calculated a linear model on that. The relative lagtime of CC121 and CC9 is shorter compared to CC1 and CC6. Interpretation: CC121 for example is quicker (shorter lag time) to adapt to salt compared to CC1.

Questions:

Is there a correlation between relative lag time/Vmax and CC? Is there a correlation between relative lag time/Vmax and source? Is there a correlation between relative lag time/Vmax and lineage? Is there a correlation between relative lag time/Vmax and serotype?

A second set of questions we can ask this dataset:

Are there strains that generally grow faster than others? regardless of salt or BHI, I think we can just calculate this for BHI since there seems to be no differences in relative Vmax anyway (e.g., the strains that are slow in BHI are also slow in salt and vice versa) .

questions:

Is there a correlation between Vmax and CC? Is there a correlation between Vmax and source? Is there a correlation between Vmax and lineage? Is there a correlation between Vmax and serotype?

The data

Huge ugly table

```
library(multcompView)
library(ggplot2)
library(lsmeans)
```

```
## Warning: package 'lsmeans' was built under R version 3.4.4
```

```
## The 'lsmeans' package is being deprecated.
## Users are encouraged to switch to 'emmeans'.
## See help('transition') for more information, including how
## to convert 'lsmeans' objects and scripts to work with 'emmeans'.
```

```
library(knitr)
```

```
## Warning: package 'knitr' was built under R version 3.4.3
```

```
library(kableExtra)
```

```
## Warning: package 'kableExtra' was built under R version 3.4.4
```

```
library(dplyr)
```

```
## Warning: package 'dplyr' was built under R version 3.4.4
```

```
##
## Attaching package: 'dplyr'
```

```
## The following objects are masked from 'package:stats':
##
##     filter, lag
```

```
## The following objects are masked from 'package:base':
##
##     intersect, setdiff, setequal, union
```

```
library(tidyr)
```

```
## Warning: package 'tidyr' was built under R version 3.4.4
```

```
salt <- read.csv(file="bhi_salt.csv") %>%
  as.tbl()
salt %>%
  kable("latex", longtable = T, booktabs = T) %>%
  kable_styling(latex_options = c("repeat_header",  "scale_down"), font_size = 4) %>%
  landscape()
```

```
## Warning in styling_latex_scale_down(out, table_info): Longtable cannot be
## resized.
```

ID	clonal.complex	serotype	lineage	source	NENT.Nr.	sequencer.ID	replicate	BHI.Vmax	BHI.lagtime	BHI.lagtime.hhmm	NaCl.Vmax	NaCl.lagtime	NaCl.lagtime.hhmm	rel.lagtime	rel.Vmax
1	CC1	4b	I	Food	N12-0605	S176	1	8.857	110.72	1:50:43 AM	5.418	148.20	2:28:12 AM	1.3385116	1.634675
2	CC1	4b	I	Food	N12-1339	S181	1	7.140	103.52	1:43:31 AM	5.420	141.62	2:21:37 AM	1.3680448	1.317363
3	CC1	4b	I	Food	N12-1996	S191	1	8.633	112.33	1:52:20 AM	5.699	139.07	2:19:04 AM	1.2380486	1.514797
4	CC1	4b	I	Food	N13-0047	S195	1	7.742	106.07	1:46:04 AM	5.396	144.42	2:24:25 AM	1.3615537	1.434855
5	CC1	4b	I	Blood	N11-2292	S163	1	7.866	119.75	1:59:45 AM	5.303	150.33	2:30:20 AM	1.2553653	1.483281
6	CC1	4b	I	Blood	LL195	LL195_CG	1	7.344	113.68	1:53:41 AM	4.960	142.83	2:22:50 AM	1.2564215	1.480546
7	CC1	4b	I	Blood	N13-0987	21931_2#72	1	8.169	120.95	2:00:57 AM	5.679	161.55	2:41:33 AM	1.3356759	1.438314
8	CC1	4b	I	Blood	N13-1079	21931_2#75	1	7.475	110.60	1:50:36 AM	4.889	146.37	2:26:22 AM	1.3234177	1.528906
9	CC6	4b	I	Food	N12-0460		1	7.054	123.53	2:03:32 AM	4.681	151.83	2:31:50 AM	1.2290941	1.506979
10	CC4	4b	I	Food	N12-1772	S187	1	6.649	106.83	1:46:50 AM	4.969	140.97	2:20:58 AM	1.3195732	1.338073
11	CC6	4b	I	Food	N13-0703		1	7.276	102.10	1:42:06 AM	4.871	132.67	2:12:40 AM	1.2994123	1.493841
12	CC6	4b	I	Food	N13-1184		1	7.271	101.92	1:41:55 AM	4.864	132.87	2:12:52 AM	1.3036695	1.494860
13	CC6	4b	I	Blood	N11-2801	S166	1	5.634	125.87	2:05:52 AM	4.195	159.73	2:39:44 AM	1.2690077	1.343214
14	CC6	4b	I	Blood	N12-1387	S182	1	3.813	166.70	2:46:42 AM	3.150	200.33	3:20:20 AM	1.2017397	1.210476
15	CC6	4b	I	Blood	N13-1271	21931_2#78	1	7.205	102.03	1:42:02 AM	4.818	133.35	2:13:21 AM	1.3069685	1.495468
16	CC6	4b	I	Blood	N13-1507	21931_2#84	1	7.465	115.08	1:55:05 AM	5.275	148.88	2:28:53 AM	1.2937087	1.415166
39	CC54	4b	I	Blood	N13-0177	S196	1	7.493	116.48	1:56:29 AM	5.629	158.68	2:38:41 AM	1.3622940	1.331083
40	CC224	1/2b	I	Blood	N12-1608	S122	1	7.739	116.80	1:56:48 AM	5.441	158.77	2:38:46 AM	1.3593322	1.422349
1	CC1	4b	I	Food	N12-0605	S176	2	7.017	122.03	2:02:02 AM	5.387	154.05	2:34:03 AM	1.2623945	1.302599
2	CC1	4b	I	Food	N12-1339	S181	2	7.220	113.55	1:53:33 AM	5.315	138.33	2:18:20 AM	1.2182299	1.358505
3	CC1	4b	I	Food	N12-1996	S191	2	8.060	121.70	2:01:42 AM	5.238	151.72	2:31:43 AM	1.2466721	1.538853
4	CC1	4b	I	Food	N13-0047	S195	2	7.433	116.65	1:56:39 AM	5.308	151.63	2:31:38 AM	1.2998714	1.400314
5	CC1	4b	I	Blood	N11-2292	S163	2	7.372	125.15	2:05:09 AM	5.099	148.43	2:28:26 AM	1.1860168	1.445803
7	CC1	4b	I	Blood	N13-0987	21931_2#72	2	7.518	123.30	2:03:18 AM	4.817	170.43	2:50:26 AM	1.3822384	1.560900
8	CC1	4b	I	Blood	N13-1079	21931_2#75	2	7.484	116.95	1:56:57 AM	4.905	154.60	2:34:36 AM	1.3219325	1.525962
9	CC6	4b	I	Food	N12-0460		2	7.618	115.48	1:55:29 AM	4.932	149.97	2:29:58 AM	1.2986664	1.544502
10	CC4	4b	I	Food	N12-1772	S187	2	6.715	115.93	1:55:56 AM	4.446	155.10	2:35:06 AM	1.3378763	1.510158
11	CC6	4b	I	Food	N13-0703		2	7.098	108.93	1:48:56 AM	4.881	148.27	2:28:16 AM	1.3611494	1.454241
12	CC6	4b	I	Food	N13-1184		2	6.902	115.23	1:55:14 AM	4.544	151.60	2:31:36 AM	1.3156296	1.519111
13	CC6	4b	I	Blood	N11-2801	S166	2	5.702	144.60	2:24:36 AM	4.024	183.88	3:03:53 AM	1.2716459	1.417081
14	CC6	4b	I	Blood	N12-1387	S182	2	3.861	165.58	2:45:35 AM	3.332	193.28	3:13:17 AM	1.1672907	1.158879
15	CC6	4b	I	Blood	N13-1271	21931_2#78	2	6.647	111.45	1:51:27 AM	4.344	151.08	2:31:05 AM	1.3555855	1.530197
16	CC6	4b	I	Blood	N13-1507	21931_2#84	2	7.370	121.63	2:01:38 AM	4.957	162.40	2:42:24 AM	1.3351969	1.486686
39	CC54	4b	I	Blood	N13-0177	S196	2	7.542	117.65	1:57:39 AM	5.100	162.48	2:42:29 AM	1.3810455	1.478920
40	CC224	1/2b	I	Blood	N12-1608	S122	2	7.545	146.48	2:26:29 AM	4.921	160.42	2:40:25 AM	1.0951666	1.533329
1	CC1	4b	I	Food	N12-0605	S176	3	7.404	119.87	1:59:52 AM	5.397	153.93	2:33:56 AM	1.2841412	1.371935
2	CC1	4b	I	Food	N12-1339	S181	3	6.949	114.98	1:54:59 AM	5.285	151.22	2:31:13 AM	1.3151853	1.314853
3	CC1	4b	I	Food	N12-1996	S191	3	7.644	123.28	2:03:17 AM	5.201	152.65	2:32:39 AM	1.2382382	1.469876
4	CC1	4b	I	Food	N13-0047	S195	3	6.798	121.90	2:01:54 AM	4.887	163.22	2:43:13 AM	1.3389664	1.390969
5	CC1	4b	I	Blood	N11-2292	S163	3	7.271	125.32	2:05:19 AM	4.936	159.73	2:39:44 AM	1.2745771	1.473023
7	CC1	4b	I	Blood	N13-0987	21931_2#72	3	7.727	123.87	2:03:52 AM	5.441	160.42	2:40:25 AM	1.2950674	1.420169
8	CC1	4b	I	Blood	N13-1079	21931_2#75	3	7.325	119.85	1:59:51 AM	4.966	151.70	2:31:42 AM	1.2657489	1.474963
9	CC6	4b	I	Food	N12-0460		3	7.658	112.75	1:52:45 AM	5.126	138.13	2:18:08 AM	1.2250998	1.493985
10	CC4	4b	I	Food	N12-1772	S187	3	6.878	111.70	1:51:42 AM	4.968	142.35	2:22:21 AM	1.2743957	1.384394
11	CC6	4b	I	Food	N13-0703		3	6.733	115.38	1:55:23 AM	4.814	154.42	2:34:25 AM	1.3383602	1.398601
12	CC6	4b	I	Food	N13-1184		3	6.504	123.45	2:03:27 AM	4.130	162.12	2:42:07 AM	1.3132442	1.574772
13	CC6	4b	I	Blood	N11-2801	S166	3	5.636	146.13	2:26:08 AM	3.895	192.32	3:12:19 AM	1.3160884	1.447022
14	CC6	4b	I	Blood	N12-1387	S182	3	3.921	165.88	2:45:53 AM	3.238	190.28	3:10:17 AM	1.1470943	1.210954
15	CC6	4b	I	Blood	N13-1271	21931_2#78	3	6.945	114.47	1:54:28 AM	4.493	159.50	2:39:30 AM	1.3933782	1.545549
16	CC6	4b	I	Blood	N13-1507	21931_2#84	3	7.753	123.88	2:03:53 AM	5.103	165.42	2:45:25 AM	1.3353245	1.519368
39	CC54	4b	I	Blood	N13-0177	S196	3	6.360	124.57	2:04:34 AM	5.385	169.80	2:49:48 AM	1.3630890	1.180985
40	CC224	1/2b	I	Blood	N12-1608	S122	3	7.936	117.98	1:57:59 AM	5.369	150.77	2:30:46 AM	1.2779285	1.477961
17	CC9	1/2c	II	Food	N11-1698	21931_2#78	1	7.709	107.33	1:47:20 AM	5.261	154.22	2:34:13 AM	1.4368769	1.465467
18	CC9	1/2c	II	Food	N12-0710	S142	1	6.709	115.37	1:55:22 AM	4.248	154.98	2:34:59 AM	1.3433302	1.579377
19	CC9	1/2c	II	Food	N12-0822	S143	1	6.971	121.47	2:01:28 AM	5.085	167.88	2:47:53 AM	1.3820696	1.370919
20	CC9	1/2c	II	Food	N11-1848		1	6.223	107.38	1:47:23 AM	4.405	148.17	2:28:10 AM	1.3798659	1.412713
21	CC9	1/2c	II	Food	N14-0261		1	7.504	120.92	2:00:55 AM	5.075	163.38	2:43:23 AM	1.3511412	1.478784
22	CC9	1/2a	II	Blood	N11-1837	S82	1	6.272	158.47	2:38:28 AM	4.727	218.35	3:38:21 AM	1.3778633	1.326869
23	CC9	1/2c	II	Blood	N12-0486	S139	1	6.920	122.12	2:02:07 AM	4.732	186.17	3:06:10 AM	1.5244841	1.462384
24	CC9	1/2c	II	Blood	N13-0001	S149	1	5.985	111.75	1:51:45 AM	5.161	172.98	2:52:59 AM	1.5479195	1.159669
25	CC121	1/2a	II	Food	N11-1218	S10	1	6.914	111.12	1:51:07 AM	5.180	157.05	2:37:03 AM	1.4133369	1.334749
26	CC121	1/2a	II	Food	N12-0571	S44	1	6.373	116.75	1:56:45 AM	4.960	165.37	2:45:22 AM	1.4164454	1.284860
27	CC121	1/2a	II	Food	N13-0369	S75	1	6.739	114.67	1:54:40 AM	4.718	155.57	2:35:34 AM	1.3566757	1.428360
28	CC121	1/2b	II	Food	N13-0836		1	5.393	111.53	1:51:32 AM	3.654	153.55	2:33:33 AM	1.3767596	1.476052
29	CC121	1/2a	II	Food	N14-0205	21903_6#63	1	6.921	112.57	1:52:34 AM	5.235	150.08	2:30:05 AM	1.3332149	1.321979

(continued)

ID	clonal.complex	serotype	lineage	source	NENT.Nr.	sequencer.ID	replicate	BHI.Vmax	BHI.lagtime	BHI.lagtime.hhmm	NaCl.Vmax	NaCl.lagtime	NaCl.lagtime.hhmm	rel.lagtime	rel.Vmax
30	CC121	3c	II	Food	N14-0322	21903_6#64	1	6.802	114.75	1:54:45 AM	5.064	153.78	2:33:47 AM	1.3401307	1.343119
31	CC121	1/2a	II	Blood	N12-0367	S109	1	6.293	122.47	2:02:28 AM	4.111	182.17	3:02:10 AM	1.4874663	1.530976
32	CC121	1/2a	II	Blood	N13-0119	S102	1	6.794	114.85	1:54:51 AM	4.629	159.17	2:39:10 AM	1.3858946	1.467881
33	ST739	1/2a	II	Food	N11-2542	S30	1	7.779	255.50	4:15:30 AM	5.166	174.47	2:54:28 AM	0.6828571	1.505710
34	CC121	1/2a	II	Food	N13-0288	S74	1	7.360	123.13	2:03:08 AM	5.171	172.30	2:52:18 AM	1.3993340	1.423258
35	ST226	1/2a	II	Food	N13-2179	21903_6#61	1	7.516	122.83	2:02:50 AM	4.980	174.18	2:54:11 AM	1.4180575	1.509271
36	CC31	1/2a	II	Food	N13-0228	S70	1	8.077	123.97	2:03:58 AM	5.932	177.88	2:57:53 AM	1.4348633	1.361618
37	CC207	1/2a	II	Blood	N12-1107	S93	1	7.813	129.52	2:09:31 AM	5.805	205.58	3:25:35 AM	1.5872452	1.346043
38	CC415	1/2b	II	Blood	N13-0762	S133	1	7.186	125.38	2:05:23 AM	5.073	179.57	2:59:34 AM	1.4322061	1.416360
17	CC9	1/2c	II	Food	N11-1698	21931_2#78	2	7.353	134.02	2:14:01 AM	4.605	200.45	3:20:27 AM	1.4956723	1.596931
18	CC9	1/2c	II	Food	N12-0710	S142	2	7.233	127.13	2:07:08 AM	4.302	171.50	2:51:30 AM	1.3490128	1.681258
19	CC9	1/2c	II	Food	N12-0822	S143	2	7.633	108.25	1:48:15 AM	5.195	157.28	2:37:17 AM	1.4529330	1.469297
20	CC9	1/2c	II	Food	N11-1848		2	6.271	113.32	1:53:19 AM	4.808	173.83	2:53:50 AM	1.5339746	1.304444
21	CC9	1/2c	II	Food	N14-0261		2	7.704	119.33	1:59:20 AM	5.040	174.15	2:54:09 AM	1.4593983	1.528638
22	CC9	1/2a	II	Blood	N11-1837	S82	2	6.680	165.87	2:45:52 AM	4.660	237.55	3:57:33 AM	1.4321457	1.433476
23	CC9	1/2c	II	Blood	N12-0486	S139	2	7.176	116.63	1:56:38 AM	4.650	186.28	3:06:17 AM	1.5971877	1.543044
24	CC9	1/2c	II	Blood	N13-0001	S149	2	6.493	128.67	2:08:40 AM	4.233	198.95	3:18:57 AM	1.5462035	1.533701
25	CC121	1/2a	II	Food	N11-1218	S10	2	6.870	116.18	1:56:11 AM	4.768	174.55	2:54:33 AM	1.5024101	1.440825
26	CC121	1/2a	II	Food	N12-0571	S44	2	6.840	119.37	1:59:22 AM	4.853	169.02	2:49:01 AM	1.4159337	1.409341
27	CC121	1/2a	II	Food	N13-0369	S75	2	7.100	115.20	1:55:12 AM	4.919	160.32	2:40:19 AM	1.3916667	1.443285
28	CC121	1/2b	II	Food	N13-0836		2	5.776	108.95	1:48:57 AM	4.178	157.82	2:37:49 AM	1.4485544	1.382510
29	CC121	1/2a	II	Food	N14-0205	21903_6#63	2	7.415	111.22	1:51:13 AM	5.187	154.08	2:34:05 AM	1.3853623	1.429563
30	CC121	3c	II	Food	N14-0322	21903_6#64	2	6.972	112.98	1:52:59 AM	5.000	159.65	2:39:39 AM	1.4130820	1.394333
31	CC121	1/2a	II	Blood	N12-0367	S109	2	6.562	127.70	2:07:42 AM	4.150	188.00	3:08:00 AM	1.4722005	1.581412
32	CC121	1/2a	II	Blood	N13-0119	S102	2	7.264	124.53	2:04:32 AM	4.540	169.67	2:49:40 AM	1.3624829	1.600073
33	ST739	1/2a	II	Food	N11-2542	S30	2	7.264	126.55	2:06:33 AM	5.184	180.33	3:00:20 AM	1.4249704	1.401209
34	CC121	1/2a	II	Food	N13-0288	S74	2	6.884	114.62	1:54:37 AM	4.638	173.07	2:53:04 AM	1.5099459	1.484439
35	ST226	1/2a	II	Food	N13-2179	21903_6#61	2	7.615	119.60	1:59:36 AM	5.242	178.22	2:58:13 AM	1.4901338	1.452661
36	CC31	1/2a	II	Food	N13-0228	S70	2	7.867	113.22	1:53:13 AM	5.831	171.82	2:51:49 AM	1.5175764	1.349111
37	CC207	1/2a	II	Blood	N12-1107	S93	2	7.515	124.55	2:04:33 AM	5.367	202.27	3:22:16 AM	1.6240064	1.400286
38	CC415	1/2b	II	Blood	N13-0762	S133	2	7.018	122.45	2:02:27 AM	4.926	173.08	2:53:05 AM	1.4134749	1.424521
17	CC9	1/2c	II	Food	N11-1698	21931_2#78	3	7.536	120.33	2:00:20 AM	4.954	174.68	2:54:41 AM	1.4516746	1.521025
18	CC9	1/2c	II	Food	N12-0710	S142	3	6.896	132.98	2:12:59 AM	4.048	172.28	2:52:17 AM	1.2955332	1.703475
19	CC9	1/2c	II	Food	N12-0822	S143	3	7.502	117.72	1:57:43 AM	5.279	167.80	2:47:48 AM	1.4254162	1.421166
20	CC9	1/2c	II	Food	N11-1848		3	6.267	118.27	1:58:16 AM	4.793	174.82	2:54:49 AM	1.4781432	1.307553
21	CC9	1/2c	II	Food	N14-0261		3	7.309	123.18	2:03:11 AM	4.833	176.80	2:56:48 AM	1.4352979	1.512311
22	CC9	1/2a	II	Blood	N11-1837	S82	3	6.172	211.80	3:31:48 AM	4.418	254.95	4:14:57 AM	1.2037299	1.397118
23	CC9	1/2c	II	Blood	N12-0486	S139	3	6.568	156.07	2:36:04 AM	4.358	211.10	3:31:06 AM	1.3525982	1.507037
24	CC9	1/2c	II	Blood	N13-0001	S149	3	5.924	133.10	2:13:06 AM	4.198	182.70	3:02:42 AM	1.3726521	1.411228
25	CC121	1/2a	II	Food	N11-1218	S10	3	6.759	132.97	2:12:58 AM	4.809	180.50	3:00:30 AM	1.3574490	1.405657
26	CC121	1/2a	II	Food	N12-0571	S44	3	5.899	140.40	2:20:24 AM	4.244	187.08	3:07:05 AM	1.3324786	1.389775
27	CC121	1/2a	II	Food	N13-0369	S75	3	6.342	135.20	2:15:12 AM	4.434	183.25	3:03:15 AM	1.3553994	1.430386
28	CC121	1/2b	II	Food	N13-0836		3	5.104	137.62	2:17:37 AM	3.131	176.03	2:56:02 AM	1.2791019	1.630083
29	CC121	1/2a	II	Food	N14-0205	21903_6#63	3	6.290	129.90	2:09:54 AM	4.386	172.58	2:52:35 AM	1.3285604	1.434000
30	CC121	3c	II	Food	N14-0322	21903_6#64	3	6.301	134.25	2:14:15 AM	4.506	173.58	2:53:35 AM	1.2929609	1.398254
31	CC121	1/2a	II	Blood	N12-0367	S109	3	7.005	146.98	2:26:59 AM	3.942	186.20	3:06:12 AM	1.2668390	1.777101
32	CC121	1/2a	II	Blood	N13-0119	S102	3	7.583	133.23	2:13:14 AM	4.502	162.00	2:42:00 AM	1.2159424	1.684362
33	ST739	1/2a	II	Food	N11-2542	S30	3	7.188	133.97	2:13:58 AM	5.171	177.12	2:57:07 AM	1.3220870	1.389995
34	CC121	1/2a	II	Food	N13-0288	S74	3	6.133	128.57	2:08:34 AM	4.478	181.72	3:01:43 AM	1.4133935	1.369761
35	ST226	1/2a	II	Food	N13-2179	21903_6#61	3	6.892	122.15	2:02:09 AM	5.362	177.20	2:57:12 AM	1.4506754	1.285261
36	CC31	1/2a	II	Food	N13-0228	S70	3	7.956	128.77	2:08:46 AM	5.720	174.72	2:54:43 AM	1.3568378	1.390828
37	CC207	1/2a	II	Blood	N12-1107	S93	3	7.611	128.30	2:08:18 AM	5.774	192.20	3:12:12 AM	1.4980514	1.318132
38	CC415	1/2b	II	Blood	N13-0762	S133	3	7.102	126.50	2:06:30 AM	5.091	171.80	2:51:48 AM	1.3581028	1.394985
6	CC1	4b		Blood	LL195	LL195_CG	2	7.256	117.02	1:57:01 AM	5.420	147.18	2:27:11 AM	1.2577337	1.338807
6	CC1	4b		Blood	LL195	LL195_CG	3	7.231	119.38	1:59:23 AM	5.168	151.47	2:31:28 AM	1.2688055	1.399342

Relative Lag-Time Models

Plots

I have removed ST739 rep 1, which is the only observation with a relative lag greater than 1 (and it's quite a lot larger than 1), and which is quite different from the other two reps for ST739.

I've ensured that all CC1 observations are listed as lineage I.

Here are plots by Strain, Serotype, CC, Lineage, and Source:

```
salt <- salt %>%
  mutate(lineage=as.character(lineage)) %>%
  mutate(lineage=ifelse(clonal.complex=="CC1","I",lineage)) %>%
  filter(clonal.complex!="ST739" | replicate!=1)
```

```
## Warning: package 'bindrcpp' was built under R version 3.4.4
```

```
salt %>%
  ggplot(aes(x=NENT.Nr., y=BHI.lagtime)) +
  geom_boxplot()+
  theme(axis.text.x=element_text(angle=90,hjust=1))
```

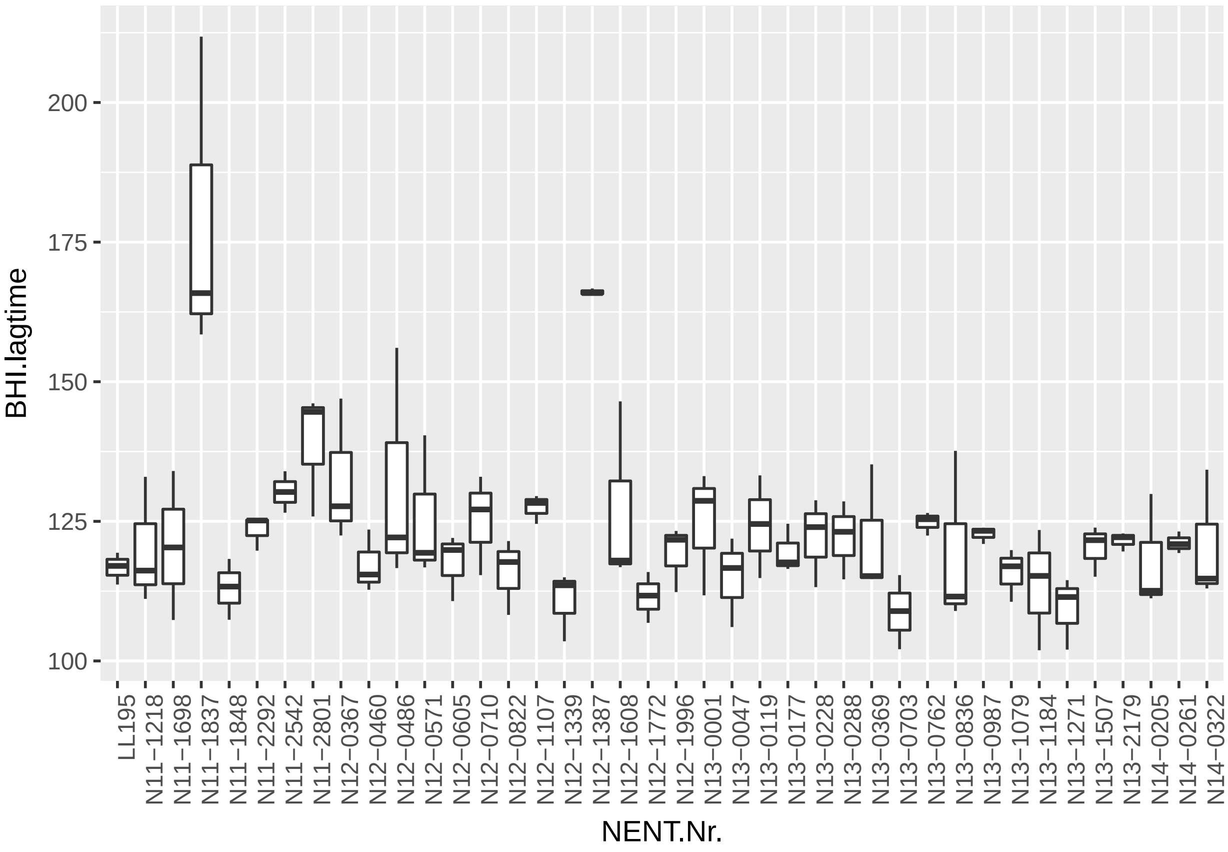


```
salt %>%
  ggplot(aes(x=serotype, y=rel.lagtime)) +
  geom_boxplot()
```

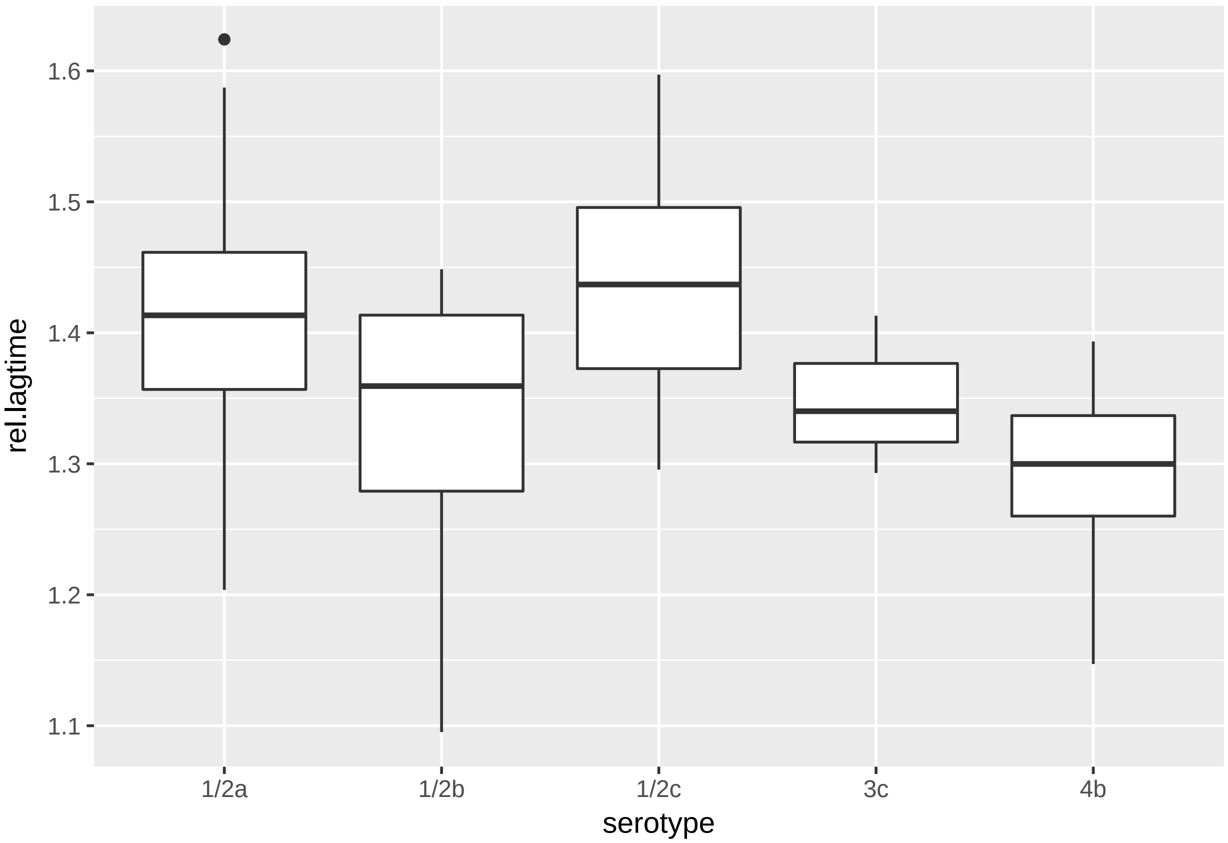


```
salt %>%
  ggplot(aes(x=clonal.complex, y=rel.lagtime)) +
  geom_boxplot()
```

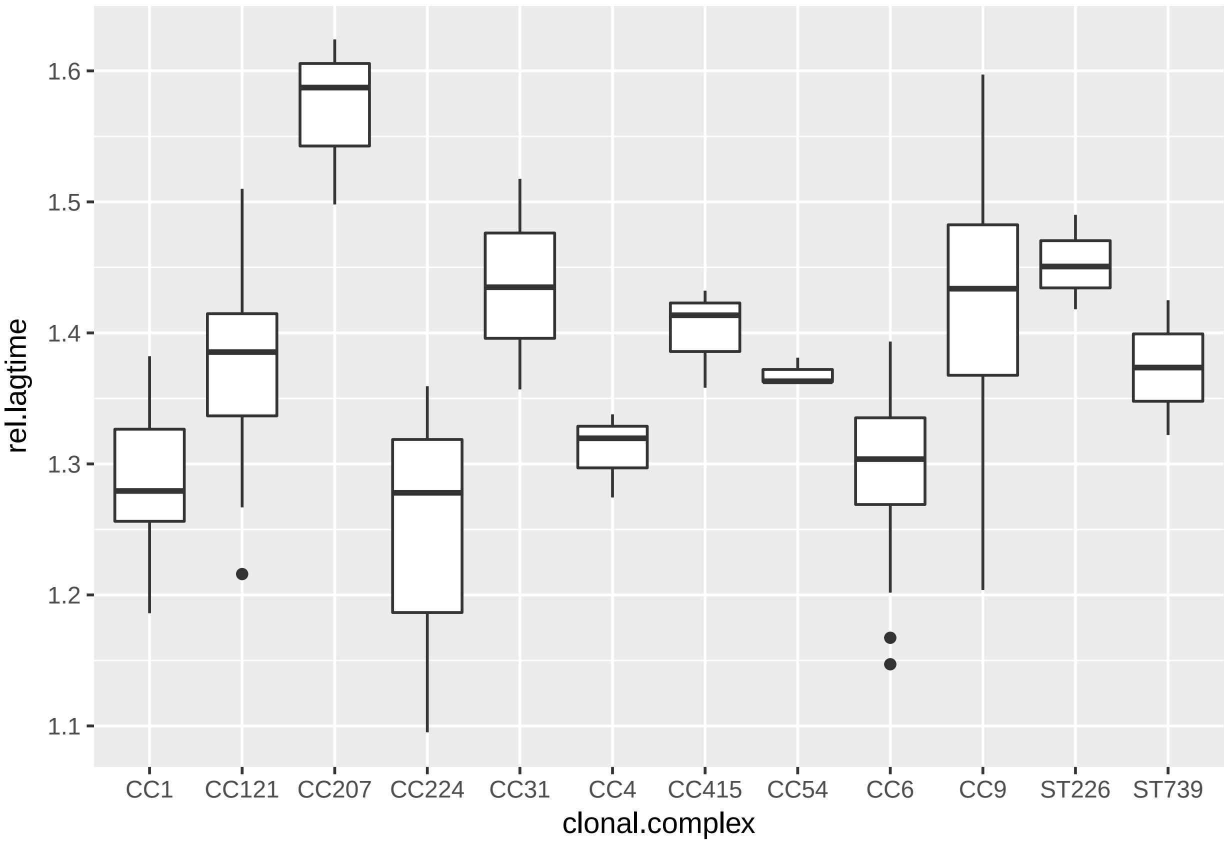


```
salt %>%
  ggplot(aes(x=lineage, y=rel.lagtime)) +
  geom_boxplot()
```

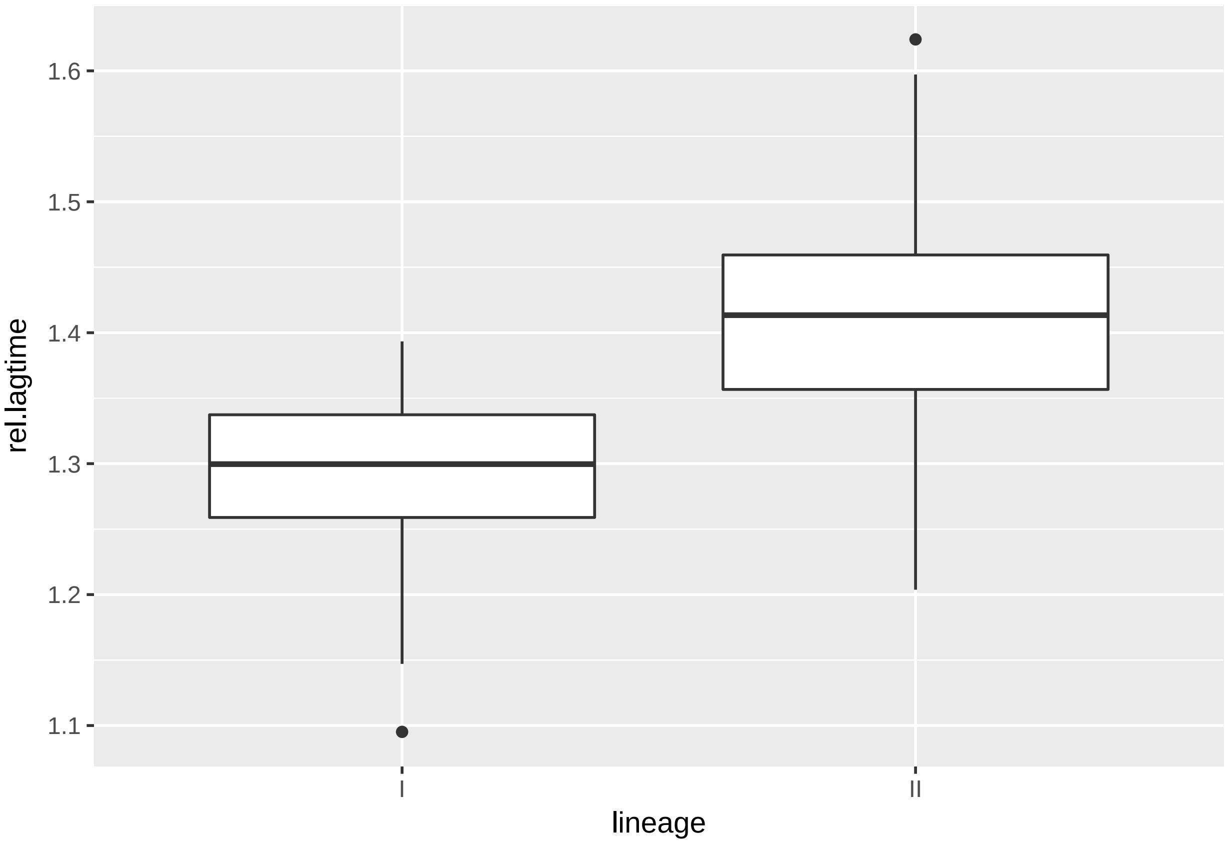


```
salt %>%
  ggplot(aes(x=source, y=rel.lagtime)) +
  geom_boxplot()
```

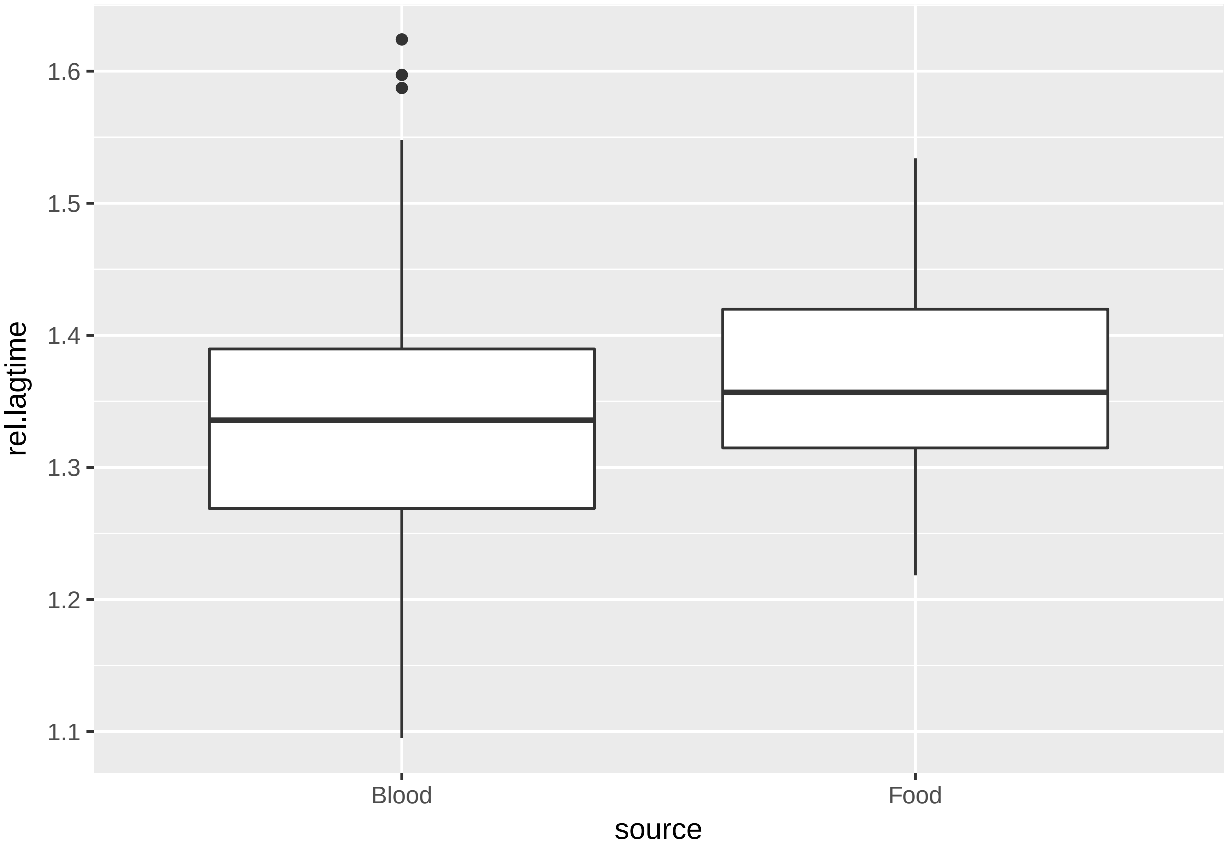

Looks like CC (except CC1, which has a subject with no lineage? is that a mistake?) is nested within lineage (each CC belongs to exactly one lineage) but the same is not true of serotype/lineage or serotype/CC. So let's try a `serotype + CC` model and examine the lineage effect with contrasts. Afterwards, we'll investigate the source

I'm going to fit one model for each question here, rather than trying to fit one "best" model and doing a contrast. This is how I did the rest of the analyses.

Clonal Complex

Diagnostic plots

```
m_cc <- lm(rel.lagtime ~ clonal.complex, data=salt)
plot(predict(m_cc),resid(m_cc))
```

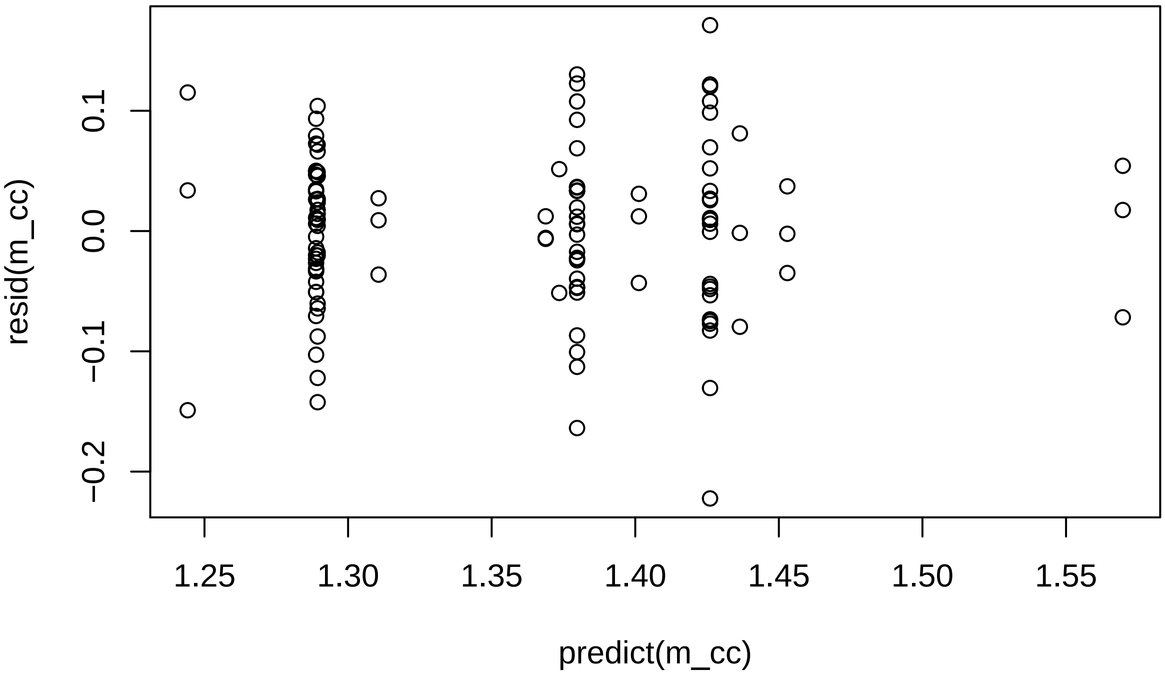


```r
qqnorm(resid(m_cc))
qqline(resid(m_cc))
```

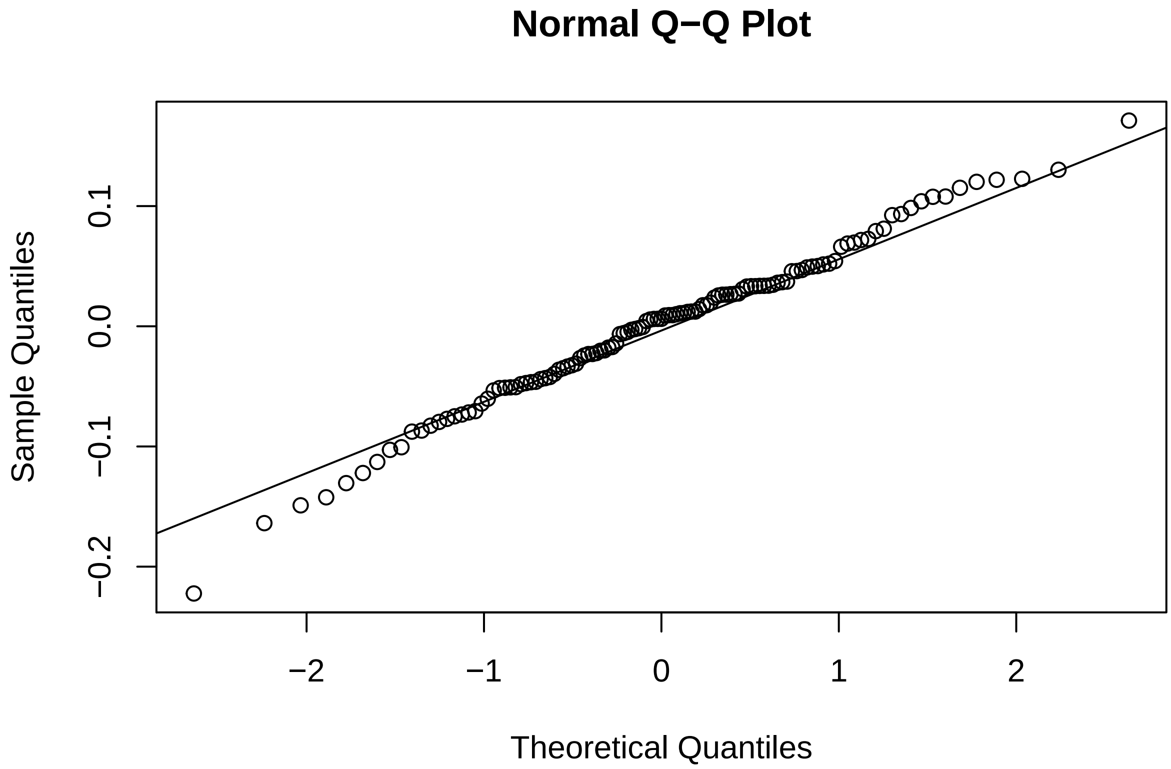

Looks fine.

Model summary

```r
summary(m_cc)
```

```
##
## Call:
## lm(formula = rel.lagtime ~ clonal.complex, data = salt)
##
## Residuals:
##       Min        1Q    Median        3Q       Max
## -0.222317 -0.043568  0.006198  0.036448  0.171141
##
## Coefficients:
##                       Estimate Std. Error t value Pr(>|t|)
## (Intercept)          1.2888691  0.0143818  89.618  < 2e-16 ***
## clonal.complexCC121  0.0908725  0.0197659   4.597 1.18e-05 ***
## clonal.complexCC207  0.2808986  0.0431455   6.510 2.49e-09 ***
## clonal.complexCC224 -0.0447267  0.0431455  -1.037 0.302237
## clonal.complexCC31   0.1475567  0.0431455   3.420 0.000887 ***
## clonal.complexCC4    0.0217460  0.0431455   0.504 0.615287
## clonal.complexCC415  0.1123922  0.0431455   2.605 0.010496 *
## clonal.complexCC54   0.0799404  0.0431455   1.853 0.066665 .
## clonal.complexCC6    0.0005287  0.0210528   0.025 0.980010
## clonal.complexCC9    0.1371777  0.0203390   6.745 8.10e-10 ***
## clonal.complexST226  0.1640865  0.0431455   3.803 0.000238 ***
## clonal.complexST739  0.0846596  0.0518544   1.633 0.105484
## ---
## Signif. codes:  0 '***' 0.001 '**' 0.01 '*' 0.05 '.' 0.1 ' ' 1
##
## Residual standard error: 0.07046 on 107 degrees of freedom
## Multiple R-squared:  0.5174, Adjusted R-squared:  0.4678
## F-statistic: 10.43 on 11 and 107 DF,  p-value: 9.398e-13
```

ANOVA

```r
anova(m_cc)
```

```
## Analysis of Variance Table
##
## Response: rel.lagtime
##                 Df  Sum Sq  Mean Sq F value    Pr(>F)
## clonal.complex  11 0.56953 0.051776   10.43 9.398e-13 ***
## Residuals      107 0.53116 0.004964
## ---
## Signif. codes:  0 '***' 0.001 '**' 0.01 '*' 0.05 '.' 0.1 ' ' 1
```

This is a regular old ANOVA table; each row tests whether the sum of squares due to that factor is greater than zero – for example, whether `clonal.complex` accounts for a significant amount of the variance in relative lag time.

Pairwise comparisons

```
cld_cc <- lsmeans(m_cc,pairwise~clonal.complex) %>%
  cld(adjust="fdr")
cld_cc %>%
  kable("latex") %>%
  kable_styling(bootstrap_options=c("striped",
                                    "hover",
                                    "condensed",
                                    "responsive"))
```

	clonal.complex	lsmean	SE	df	lower.CL	upper.CL	.group
4	CC224	1.244142	0.0406780	107	1.125031	1.363254	1
1	CC1	1.288869	0.0143818	107	1.246757	1.330981	1
9	CC6	1.289398	0.0153748	107	1.244378	1.334418	1
6	CC4	1.310615	0.0406780	107	1.191503	1.429727	12
8	CC54	1.368810	0.0406780	107	1.249698	1.487921	123
12	ST739	1.373529	0.0498201	107	1.227647	1.519410	123
2	CC121	1.379741	0.0135593	107	1.340038	1.419445	23
7	CC415	1.401261	0.0406780	107	1.282150	1.520373	23
10	CC9	1.426047	0.0143818	107	1.383935	1.468159	3
5	CC31	1.436426	0.0406780	107	1.317314	1.555538	234
11	ST226	1.452956	0.0406780	107	1.333844	1.572067	34
3	CC207	1.569768	0.0406780	107	1.450656	1.688879	4

```
cld_cc %>%
  ggplot(aes(x=clonal.complex,
             y=lsmean)) +
  geom_point(aes(color=clonal.complex)) +
  geom_errorbar(aes(ymin=lower.CL,
                    ymax=upper.CL,
                    color=clonal.complex)) +
  geom_text(aes(label=.group,y=upper.CL),vjust=-1)
```

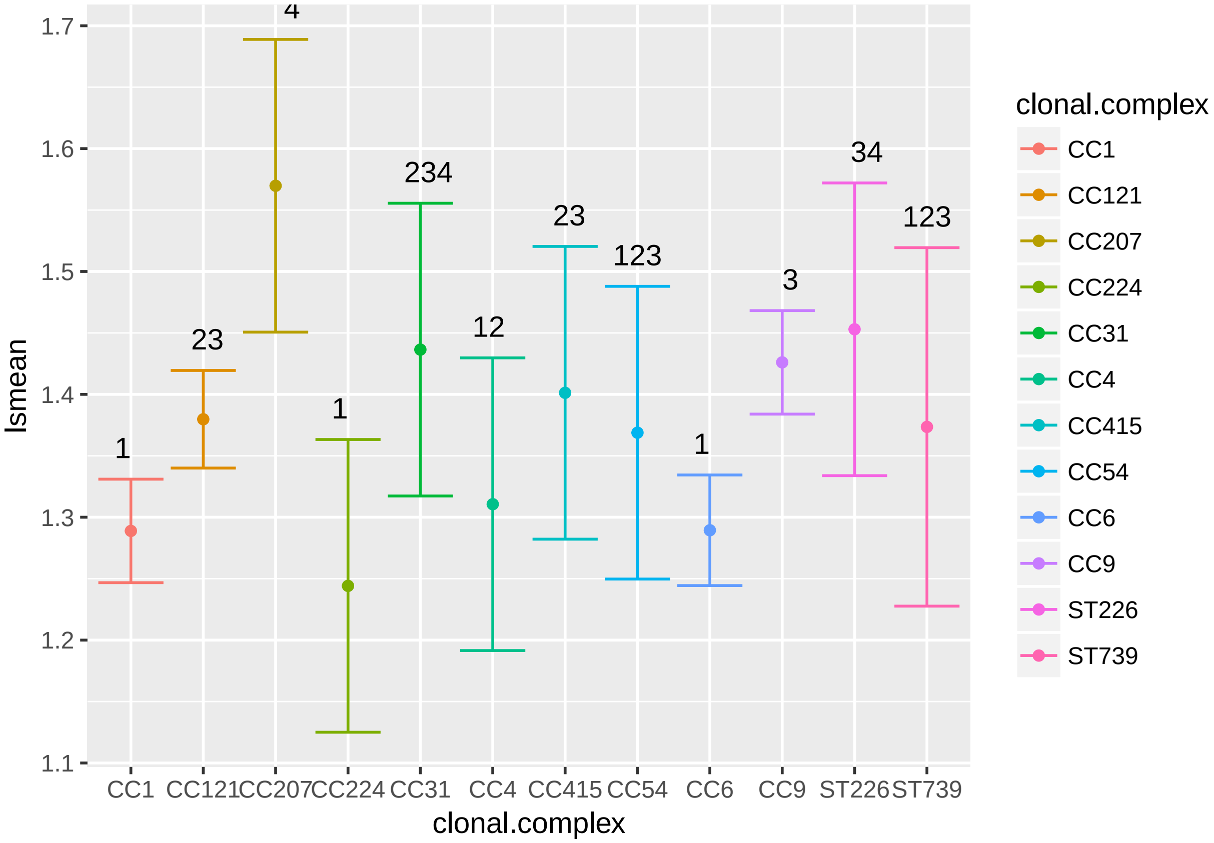

Serotype

Diagnostic plots

```
m_s <- lm(rel.lagtime ~ serotype,data=salt)
plot(predict(m_s),resid(m_s))
```

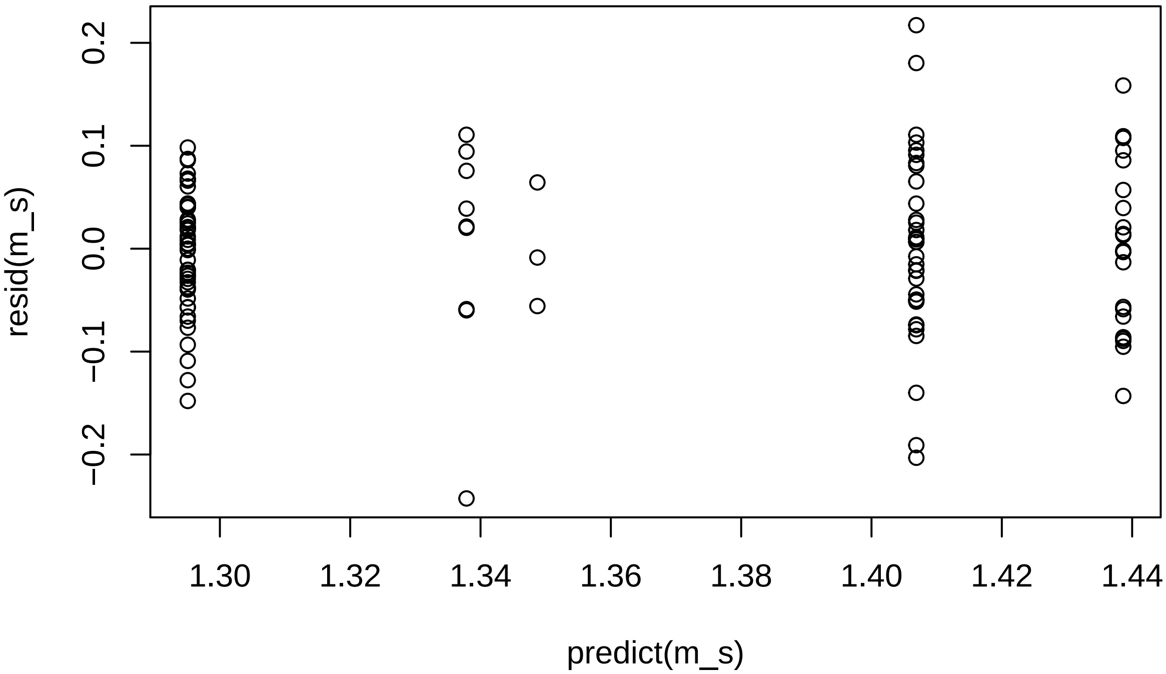


```r
qqnorm(resid(m_s))
qqline(resid(m_s))
```

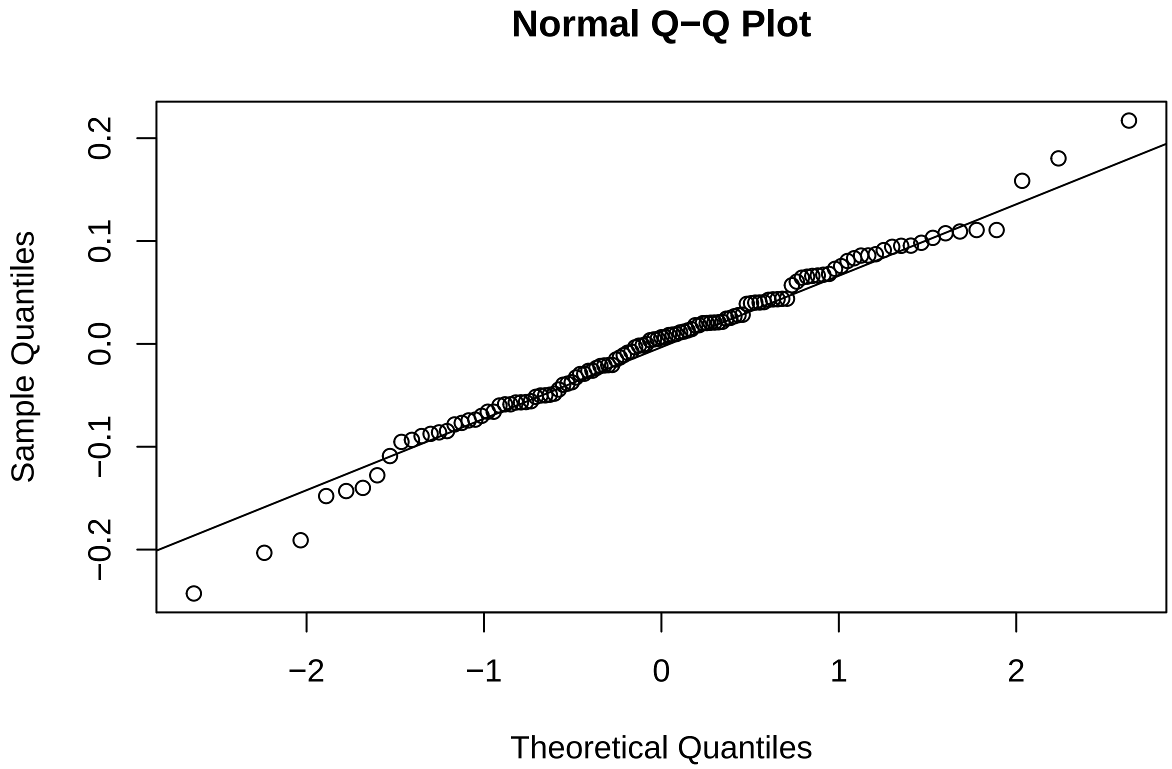

Looks fine.

Model summary

```
summary(m_s)
```

```
##
## Call:
## lm(formula = rel.lagtime ~ serotype, data = salt)
##
## Residuals:
##      Min       1Q   Median       3Q      Max
## -0.24268 -0.05012  0.00646  0.04362  0.21713
##
## Coefficients:
##              Estimate Std. Error t value Pr(>|t|)
## (Intercept)   1.40688    0.01300 108.195  < 2e-16 ***
## serotype1/2b -0.06903    0.02875  -2.401    0.018 *
## serotype1/2c  0.03176    0.02123   1.496    0.137
## serotype3c   -0.05815    0.04628  -1.257    0.211
## serotype4b   -0.11181    0.01689  -6.622 1.22e-09 ***
## ---
## Signif. codes:  0 '***' 0.001 '**' 0.01 '*' 0.05 '.' 0.1 ' ' 1
##
## Residual standard error: 0.07693 on 114 degrees of freedom
## Multiple R-squared:  0.3871, Adjusted R-squared:  0.3656
## F-statistic:    18 on 4 and 114 DF,  p-value: 1.758e-11
```

ANOVA

```
anova(m_s)
```

```
## Analysis of Variance Table
##
## Response: rel.lagtime
##            Df  Sum Sq  Mean Sq F value    Pr(>F)
## serotype    4 0.42605 0.106513  17.999 1.758e-11 ***
## Residuals 114 0.67464 0.005918
## ---
## Signif. codes:  0 '***' 0.001 '**' 0.01 '*' 0.05 '.' 0.1 ' ' 1
```

Pairwise comparisons

```
cld_s <- lsmeans(m_s,pairwise~serotype) %>%
  cld(adjust="fdr")
cld_s %>%
  kable("latex") %>%
  kable_styling(bootstrap_options=c("striped",
                                    "hover",
                                    "condensed",
                                    "responsive"))
```

	serotype	lsmean	SE	df	lower.CL	upper.CL	.group
5	4b	1.295068	0.0107720	114	1.266849	1.323287	1
2	1/2b	1.337847	0.0256425	114	1.270673	1.405022	1
4	3c	1.348725	0.0444142	114	1.232375	1.465074	12
1	1/2a	1.406877	0.0130031	114	1.372813	1.440940	2
3	1/2c	1.438637	0.0167870	114	1.394661	1.482613	2

```
cld_s %>%
  ggplot(aes(x=serotype,
             y=lsmean)) +
  geom_point(aes(color=serotype)) +
  geom_errorbar(aes(ymin=lower.CL,
                    ymax=upper.CL,
                    color=serotype)) +
  geom_text(aes(label=.group,y=upper.CL),vjust=-1)
```

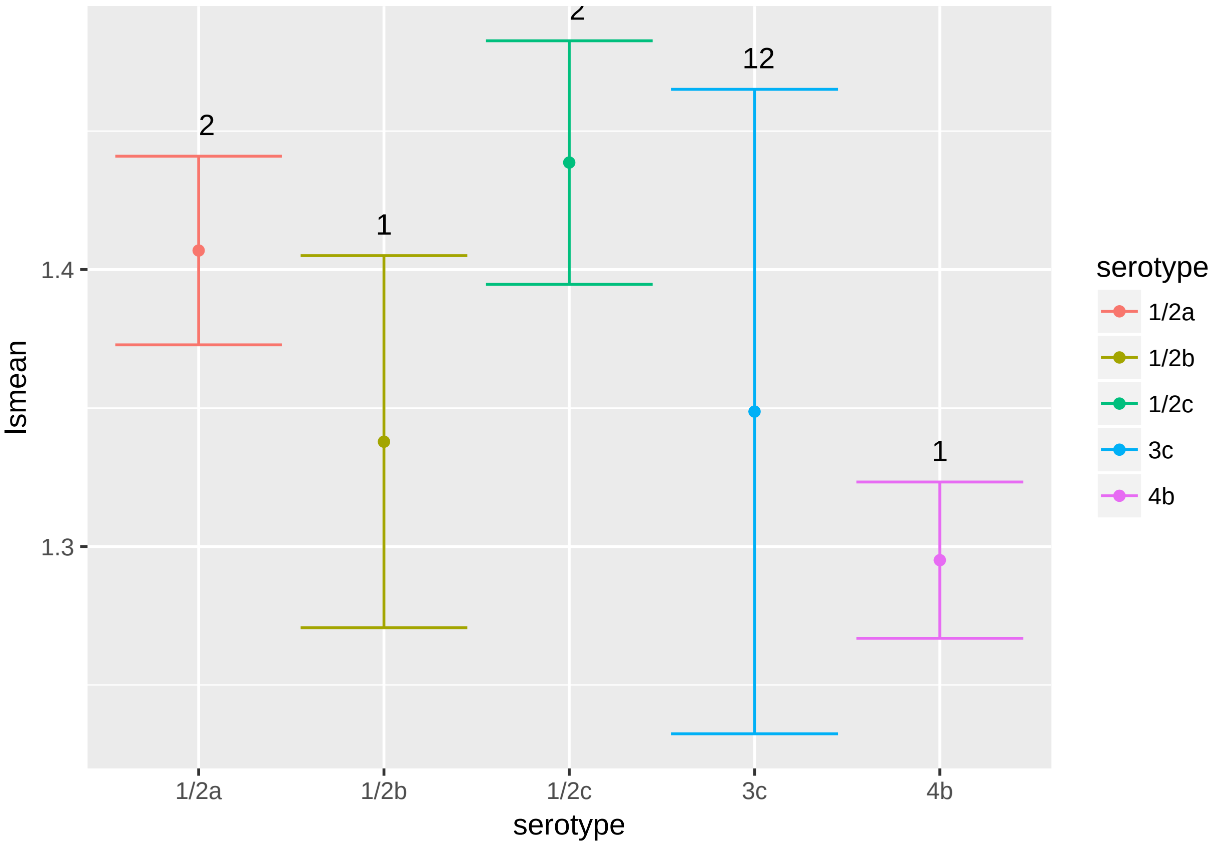

Lineage

Diagnostic plots

```
m_l <- lm(rel.lagtime ~ lineage,data=salt)
plot(predict(m_l),resid(m_l))
```

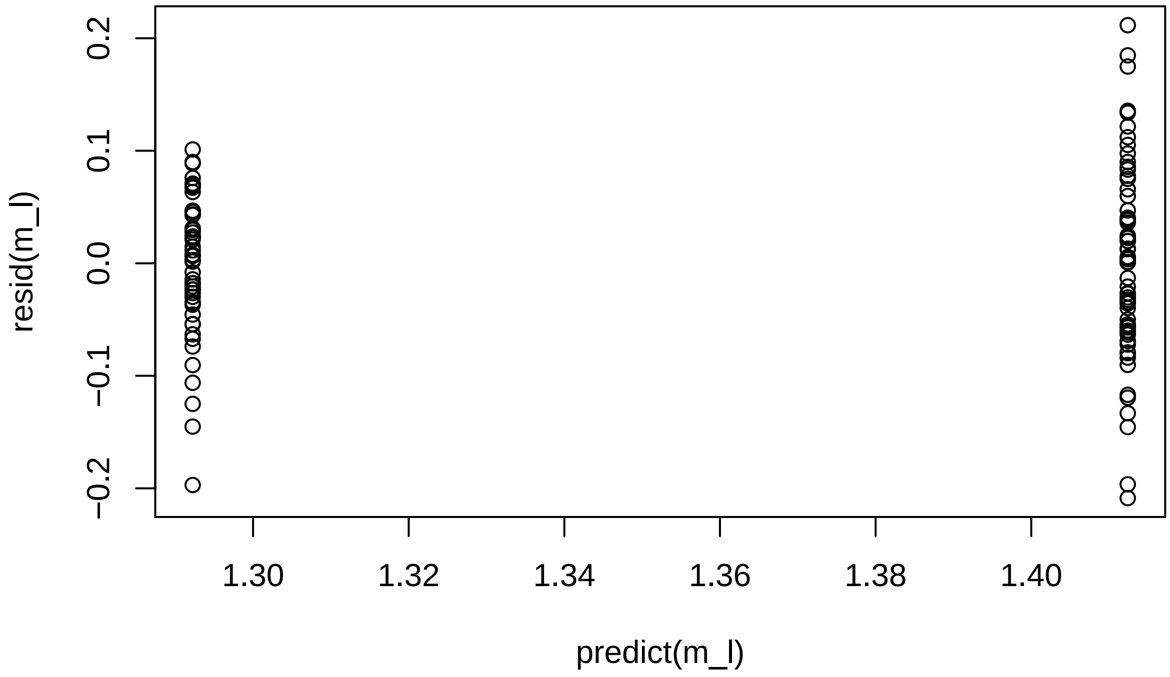


```r
qqnorm(resid(m_l))
qqline(resid(m_l))
```

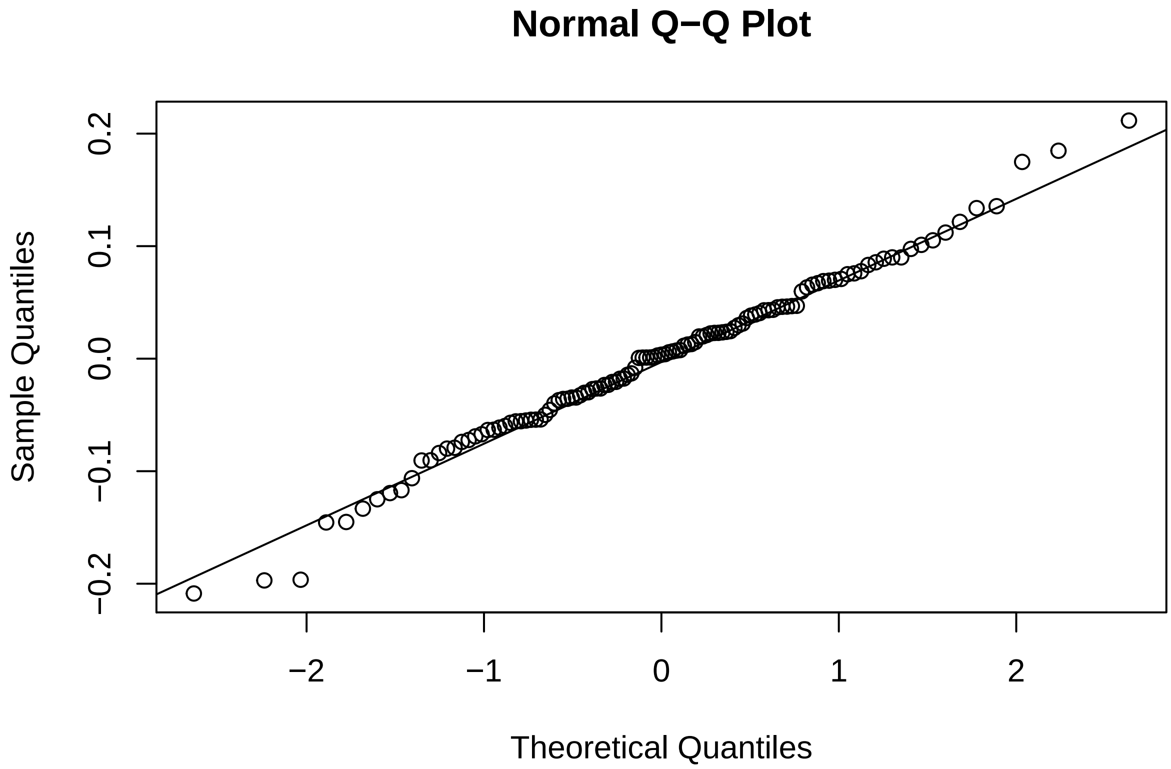

Looks fine.

Model summary

```
summary(m_l)
```

```
##
## Call:
## lm(formula = rel.lagtime ~ lineage, data = salt)
##
## Residuals:
##       Min        1Q    Median        3Q       Max
## -0.208677 -0.051962  0.003527  0.045879  0.211600
##
## Coefficients:
##             Estimate Std. Error t value Pr(>|t|)
## (Intercept)  1.29224    0.01033 125.042  < 2e-16 ***
## lineageII    0.12017    0.01398   8.594 4.32e-14 ***
## ---
## Signif. codes:  0 '***' 0.001 '**' 0.01 '*' 0.05 '.' 0.1 ' ' 1
##
## Residual standard error: 0.07594 on 117 degrees of freedom
## Multiple R-squared:  0.387,  Adjusted R-squared:  0.3817
## F-statistic: 73.85 on 1 and 117 DF,  p-value: 4.32e-14
```

ANOVA

```
anova(m_l)
```

```
## Analysis of Variance Table
##
## Response: rel.lagtime
##            Df  Sum Sq Mean Sq F value   Pr(>F)
## lineage     1 0.42593 0.42593  73.853 4.32e-14 ***
## Residuals 117 0.67476 0.00577
## ---
## Signif. codes:  0 '***' 0.001 '**' 0.01 '*' 0.05 '.' 0.1 ' ' 1
```

Pairwise comparisons

```
cld_l <- lsmeans(m_l,pairwise~lineage) %>%
  cld(adjust="fdr")
cld_l %>%
  kable("latex") %>%
  kable_styling(bootstrap_options=c("striped",
                                    "hover",
                                    "condensed",
                                    "responsive"))
```

lineage	lsmean	SE	df	lower.CL	upper.CL	.group
I	1.292239	0.0103344	117	1.268774	1.315705	1
II	1.412407	0.0094195	117	1.391019	1.433795	2

```
cld_l %>%
  ggplot(aes(x=lineage,
             y=lsmean)) +
  geom_point(aes(color=lineage)) +
  geom_errorbar(aes(ymin=lower.CL,
                    ymax=upper.CL,
                    color=lineage)) +
  geom_text(aes(label=.group,y=upper.CL),vjust=-1)
```

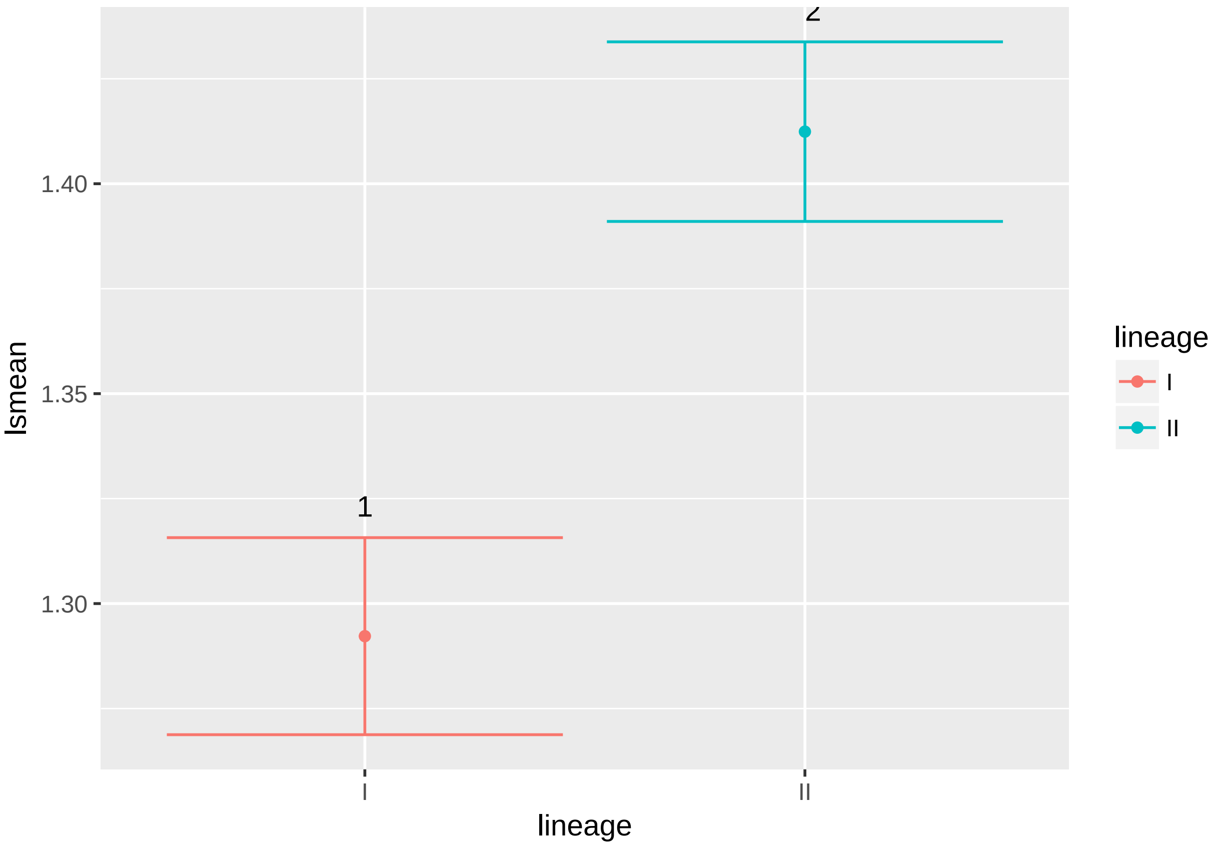

Source

Diagnostic plots

```
m_source <- lm(rel.lagtime ~ source,data=salt)
plot(predict(m_source),resid(m_source))
```

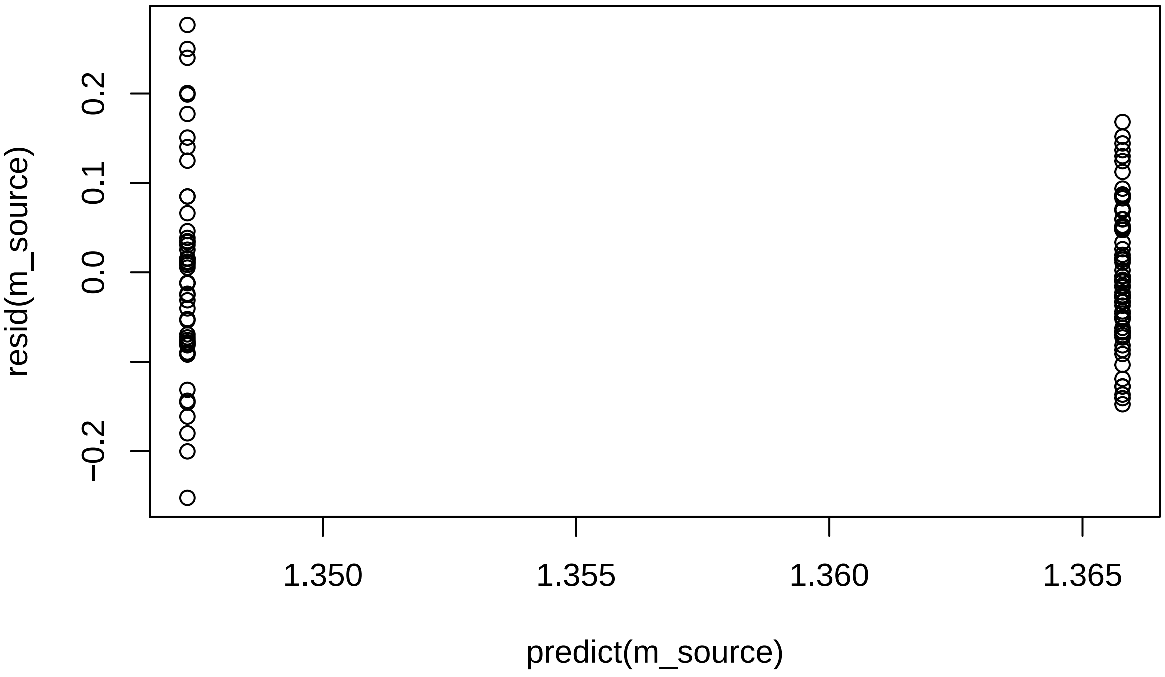


```r
qqnorm(resid(m_source))
qqline(resid(m_source))
```

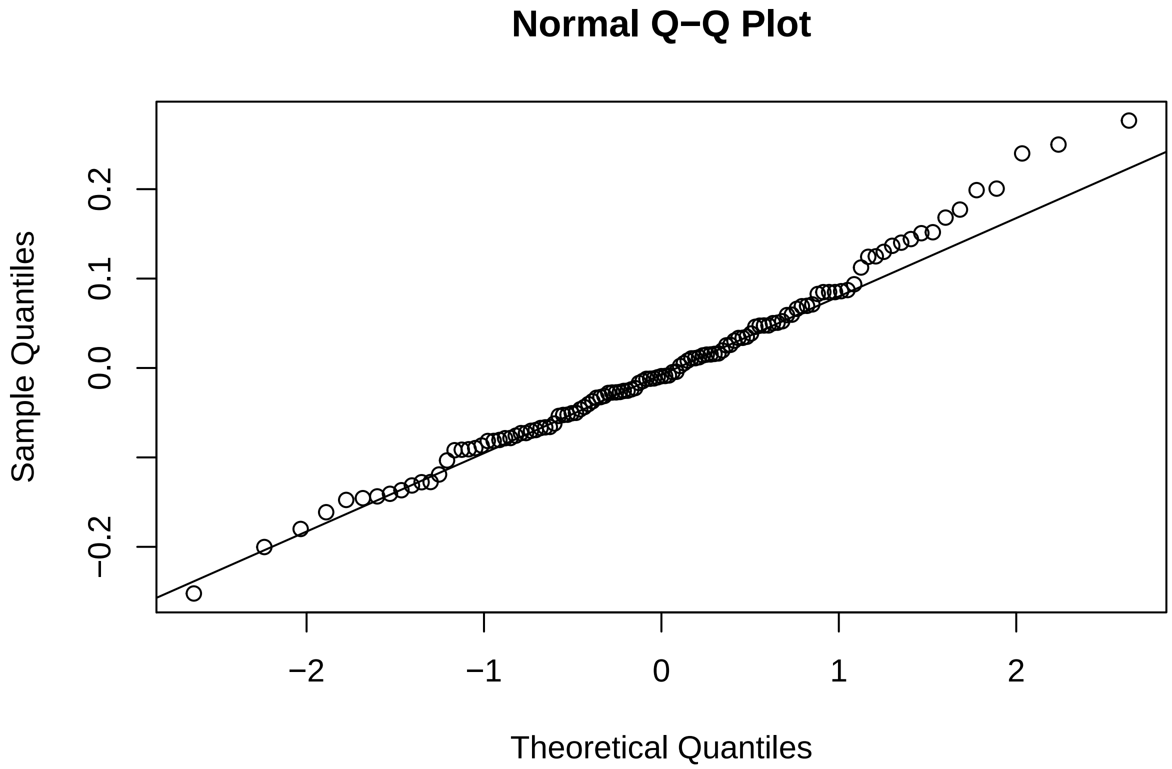

Looks fine.

Model summary

```
summary(m_source)
```

```
##
## Call:
## lm(formula = rel.lagtime ~ source, data = salt)
##
## Residuals:
##       Min        1Q    Median        3Q       Max
## -0.252159 -0.066751 -0.009115  0.051461  0.276681
##
## Coefficients:
##             Estimate Std. Error t value Pr(>|t|)
## (Intercept)  1.34733    0.01352  99.652   <2e-16 ***
## sourceFood   0.01847    0.01789   1.032    0.304
## ---
## Signif. codes:  0 '***' 0.001 '**' 0.01 '*' 0.05 '.' 0.1 ' ' 1
##
## Residual standard error: 0.09655 on 117 degrees of freedom
## Multiple R-squared:  0.009028,	Adjusted R-squared:  0.0005581
## F-statistic: 1.066 on 1 and 117 DF,  p-value: 0.304
```

ANOVA

```
anova(m_source)
```

```
## Analysis of Variance Table
##
## Response: rel.lagtime
##            Df  Sum Sq   Mean Sq F value Pr(>F)
## source      1 0.00994 0.0099370  1.0659  0.304
## Residuals 117 1.09075 0.0093227
```

The conclusion here is that `source` does *not* explain a significant amount of the variability in relative lagtime, since the overall p-value in the summary/ANOVA is large.

Relative Vmax

Plots

Here are plots by Strain, Serotype, CC, Lineage, and Source:

```
salt %>%
  ggplot(aes(x=NENT.Nr., y=rel.Vmax)) +
  geom_boxplot()+
  theme(axis.text.x=element_text(angle=90,hjust=1))
```

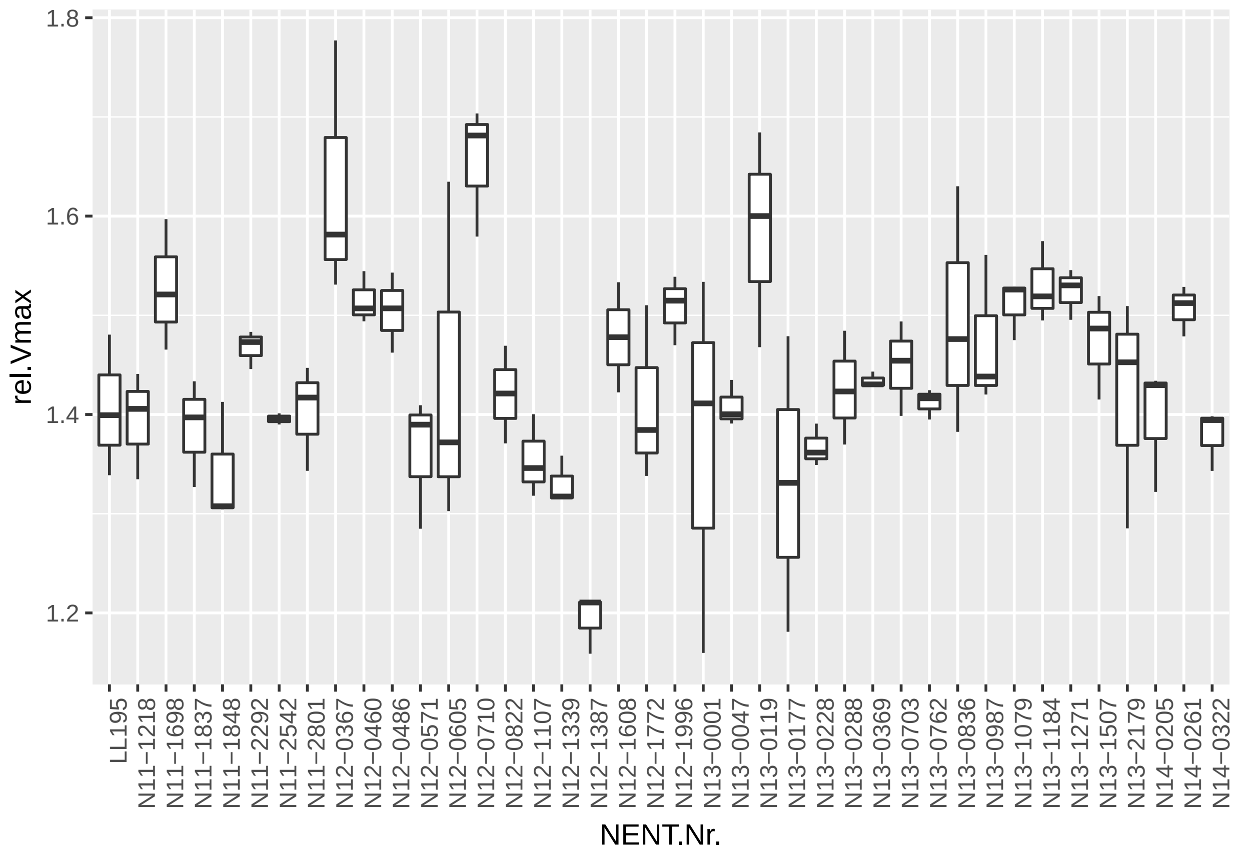


```
salt %>%
  ggplot(aes(x=serotype, y=rel.Vmax)) +
  geom_boxplot()
```

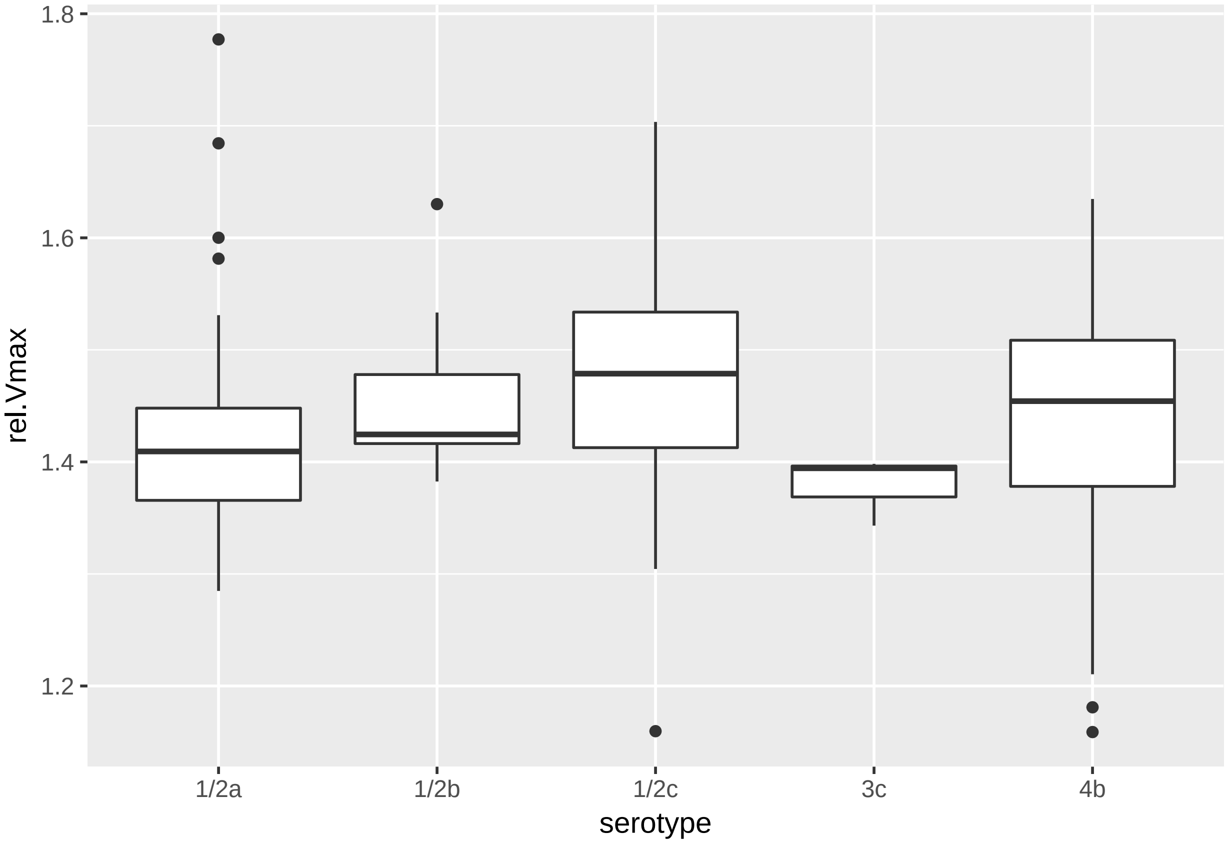


```
salt %>%
  ggplot(aes(x=clonal.complex, y=rel.Vmax)) +
  geom_boxplot()
```

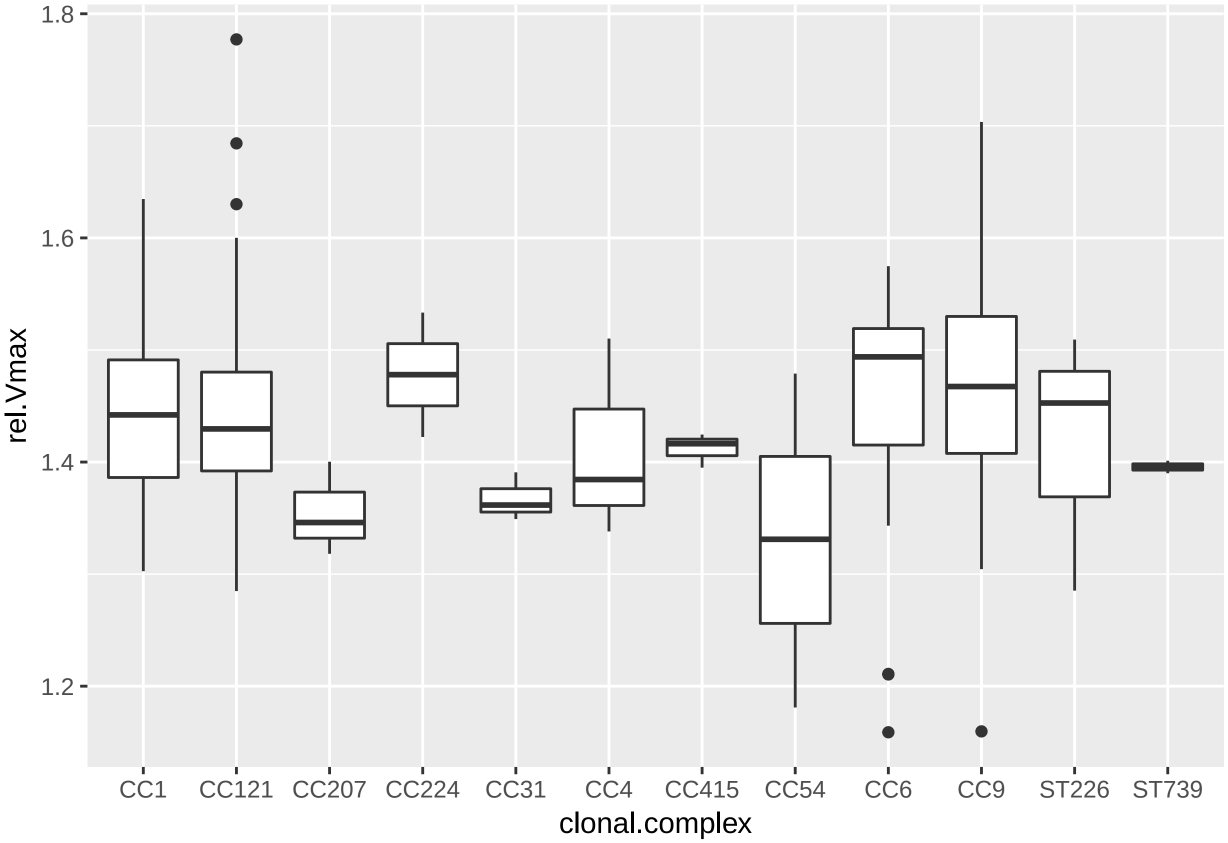


```
salt %>%
  ggplot(aes(x=lineage, y=rel.Vmax)) +
  geom_boxplot()
```

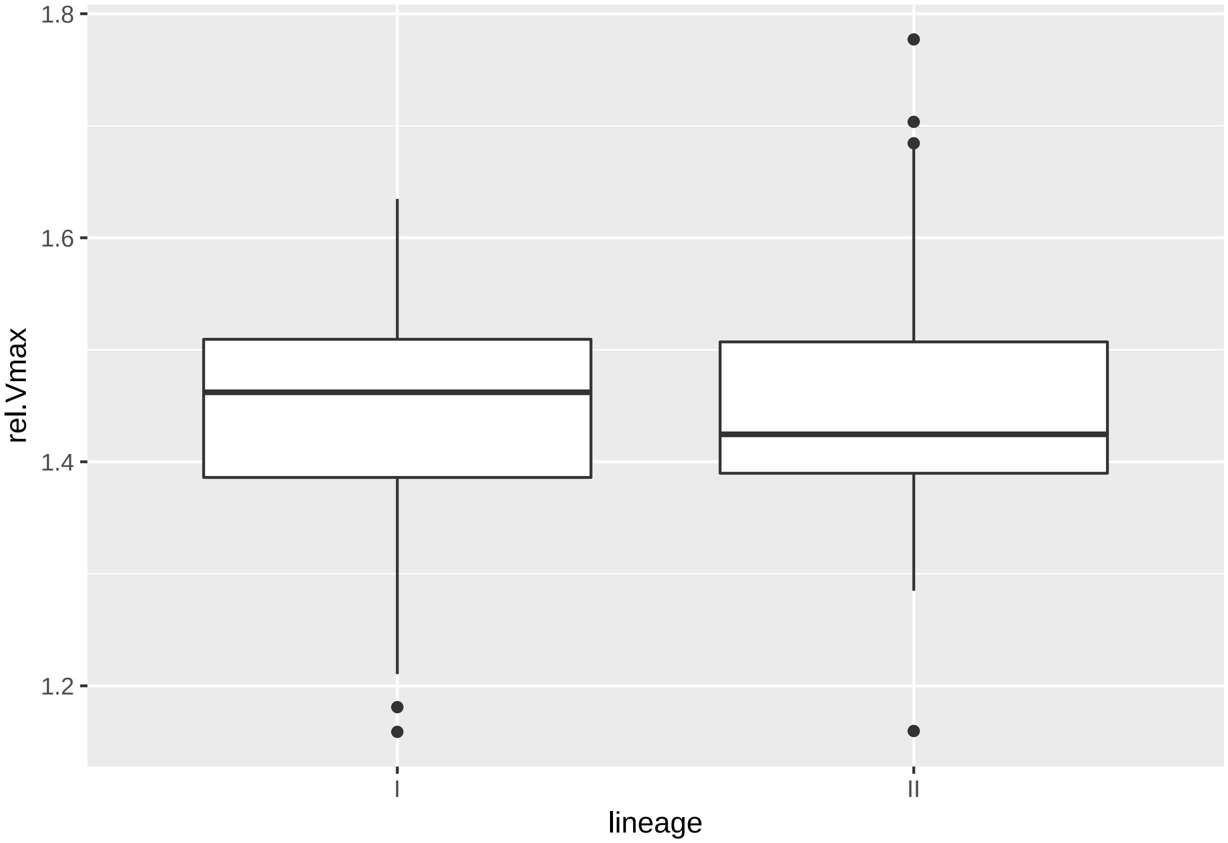


```
salt %>%
  ggplot(aes(x=source, y=rel.Vmax)) +
  geom_boxplot()
```

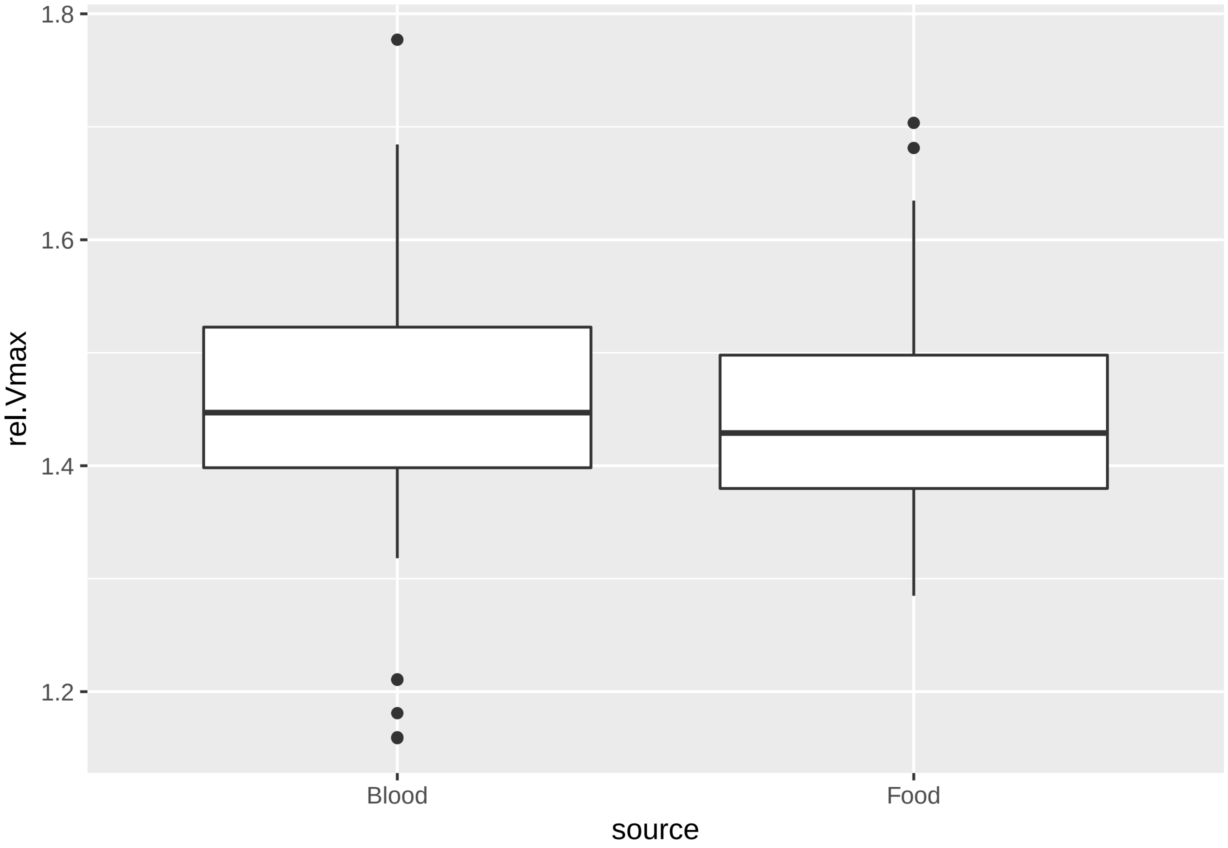

Clonal Complex

Diagnostic plots

```
m_cc <- lm(rel.Vmax ~ clonal.complex, data=salt)
plot(predict(m_cc),resid(m_cc))
```

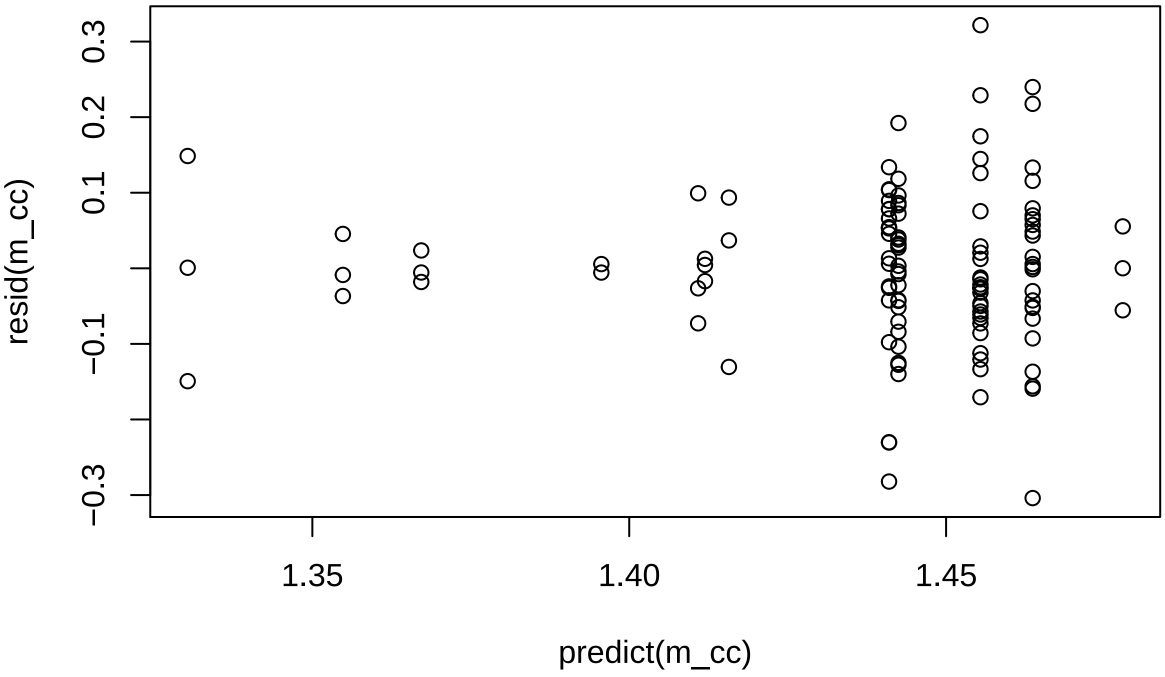


```r
qqnorm(resid(m_cc))
qqline(resid(m_cc))
```

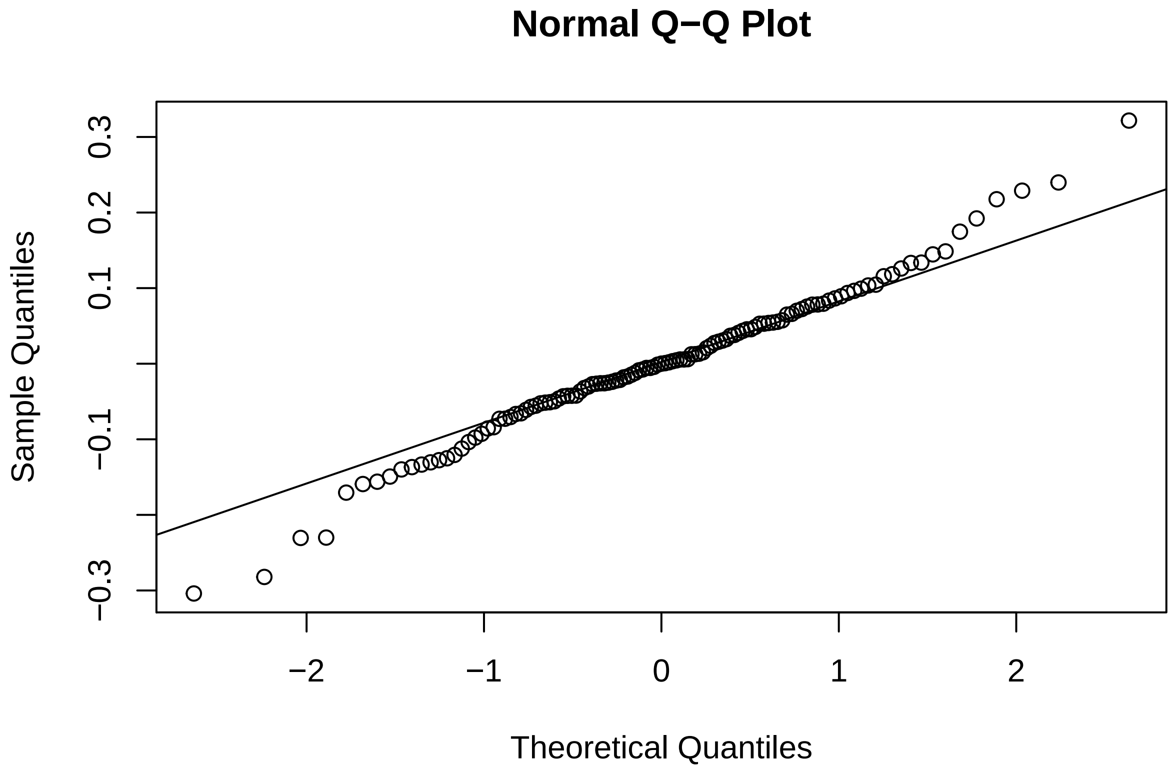

Constant variance looks a little questionable – observations with lower relative Vmax appear to have lower error variance as well. Doesn't look horrible though, and most observations are >1.40. Normality of the errors looks OK.

Model summary

```r
summary(m_cc)
```

```
##
## Call:
## lm(formula = rel.Vmax ~ clonal.complex, data = salt)
##
## Residuals:
##      Min       1Q   Median       3Q      Max
## -0.30399 -0.05197  0.00008  0.05641  0.32168
##
## Coefficients:
##                       Estimate Std. Error t value Pr(>|t|)
## (Intercept)           1.442484   0.021792  66.193   <2e-16 ***
## clonal.complexCC121  0.012938   0.029950   0.432   0.6666
## clonal.complexCC207 -0.087663   0.065376  -1.341   0.1828
## clonal.complexCC224  0.035396   0.065376   0.541   0.5893
## clonal.complexCC31  -0.075298   0.065376  -1.152   0.2520
## clonal.complexCC4   -0.031609   0.065376  -0.483   0.6297
## clonal.complexCC415 -0.030528   0.065376  -0.467   0.6415
## clonal.complexCC54  -0.112154   0.065376  -1.716   0.0891 .
## clonal.complexCC6   -0.001486   0.031900  -0.047   0.9629
## clonal.complexCC9    0.021178   0.030819   0.687   0.4935
## clonal.complexST226 -0.026753   0.065376  -0.409   0.6832
## clonal.complexST739 -0.046882   0.078573  -0.597   0.5520
## ---
## Signif. codes:  0 '***' 0.001 '**' 0.01 '*' 0.05 '.' 0.1 ' ' 1
##
## Residual standard error: 0.1068 on 107 degrees of freedom
## Multiple R-squared:  0.08183,    Adjusted R-squared:  -0.01256
## F-statistic: 0.8669 on 11 and 107 DF,  p-value: 0.5747
```

ANOVA

```r
anova(m_cc)
```

```
## Analysis of Variance Table
##
## Response: rel.Vmax
##                 Df  Sum Sq   Mean Sq F value Pr(>F)
## clonal.complex  11 0.10869 0.0098805  0.8669 0.5747
## Residuals      107 1.21954 0.0113975
```

Clonal complex does not explain a significant amount of the variance in relative Vmax.

Serotype

Diagnostic plots

```r
m_s <- lm(rel.Vmax ~ serotype,data=salt)
plot(predict(m_s),resid(m_s))
```

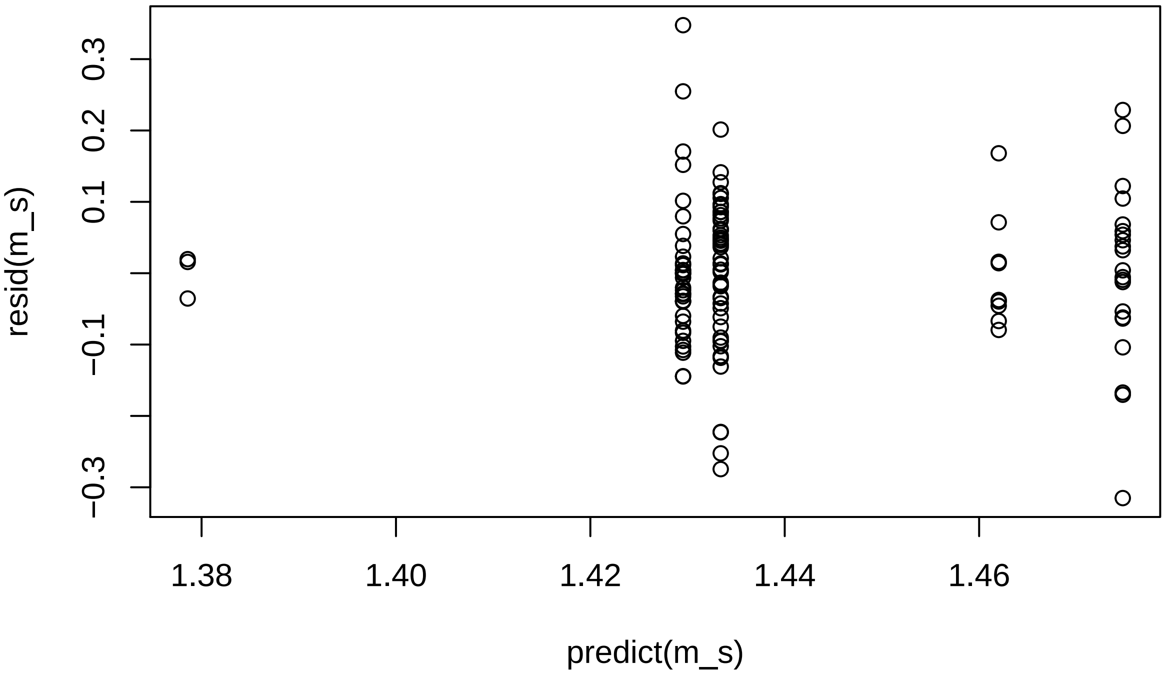


```r
qqnorm(resid(m_s))
qqline(resid(m_s))
```

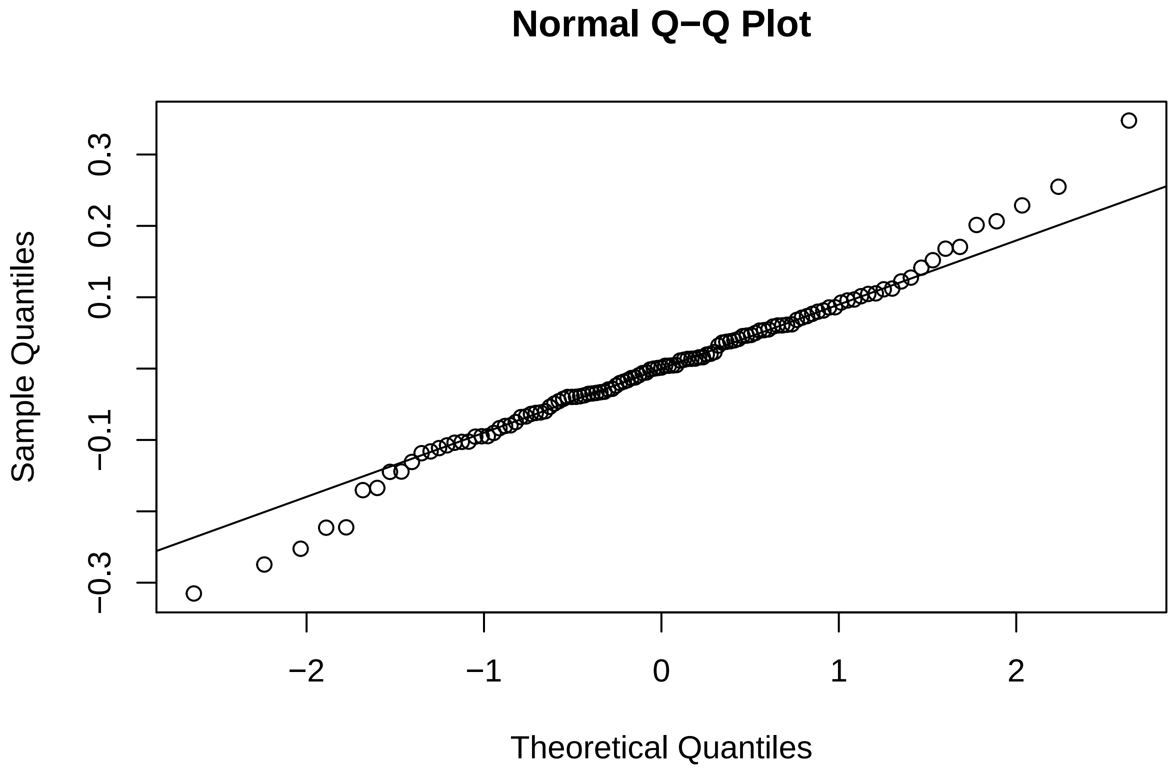

Looks fine.

Model summary

```r
summary(m_s)
```

```
##
## Call:
## lm(formula = rel.Vmax ~ serotype, data = salt)
##
## Residuals:
##      Min       1Q   Median       3Q      Max
## -0.31511 -0.06063  0.00144  0.06050  0.34756
##
## Coefficients:
##               Estimate Std. Error t value Pr(>|t|)
## (Intercept)   1.429541   0.017919  79.779   <2e-16 ***
## serotype1/2b  0.032476   0.039620   0.820    0.414
## serotype1/2c  0.045241   0.029261   1.546    0.125
## serotype3c   -0.050972   0.063773  -0.799    0.426
## serotype4b    0.003875   0.023269   0.167    0.868
## ---
## Signif. codes:  0 '***' 0.001 '**' 0.01 '*' 0.05 '.' 0.1 ' ' 1
##
## Residual standard error: 0.106 on 114 degrees of freedom
## Multiple R-squared:  0.03547,    Adjusted R-squared:  0.001629
## F-statistic: 1.048 on 4 and 114 DF,  p-value: 0.3857
```

ANOVA

```r
anova(m_s)
```

```
## Analysis of Variance Table
##
## Response: rel.Vmax
##            Df  Sum Sq  Mean Sq F value Pr(>F)
## serotype    4 0.04711 0.011779  1.0481 0.3857
## Residuals 114 1.28111 0.011238
```

Nor does serotype.

Lineage

Diagnostic plots

```r
m_l <- lm(rel.Vmax ~ lineage,data=salt)
plot(predict(m_l),resid(m_l))
```

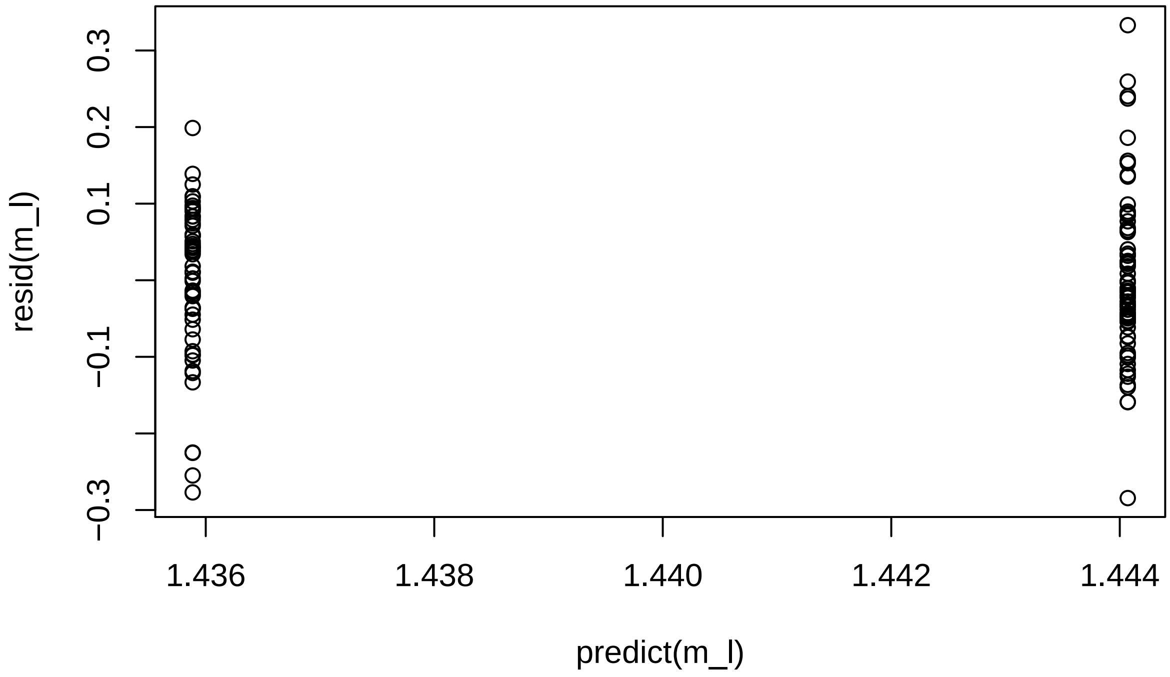


```r
qqnorm(resid(m_l))
qqline(resid(m_l))
```

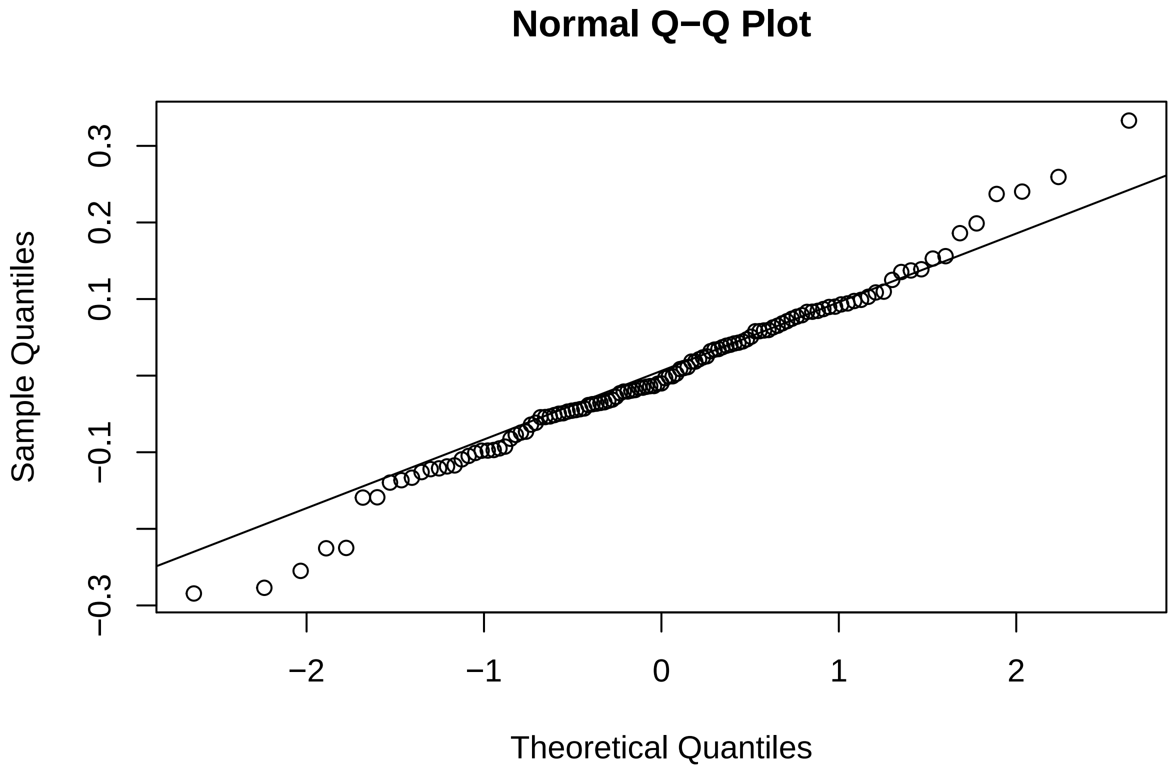

Looks fine.

Model summary

```r
summary(m_l)
```

```
##
## Call:
## lm(formula = rel.Vmax ~ lineage, data = salt)
##
## Residuals:
##      Min       1Q   Median       3Q      Max
## -0.28440 -0.05419 -0.01007  0.06672  0.33303
##
## Coefficients:
##             Estimate Std. Error t value Pr(>|t|)
## (Intercept) 1.435885   0.014488  99.105   <2e-16 ***
## lineageII   0.008185   0.019604   0.418    0.677
## ---
## Signif. codes:  0 '***' 0.001 '**' 0.01 '*' 0.05 '.' 0.1 ' ' 1
##
## Residual standard error: 0.1065 on 117 degrees of freedom
## Multiple R-squared:  0.001488,   Adjusted R-squared:  -0.007047
## F-statistic: 0.1743 on 1 and 117 DF,  p-value: 0.6771
```

ANOVA

```r
anova(m_l)
```

```
## Analysis of Variance Table
##
## Response: rel.Vmax
##            Df  Sum Sq   Mean Sq F value Pr(>F)
## lineage     1 0.00198 0.0019759  0.1743 0.6771
## Residuals 117 1.32625 0.0113354
```

Nor does lineage.

Source

Diagnostic plots

```r
m_source <- lm(rel.Vmax ~ source,data=salt)
plot(predict(m_source),resid(m_source))
```

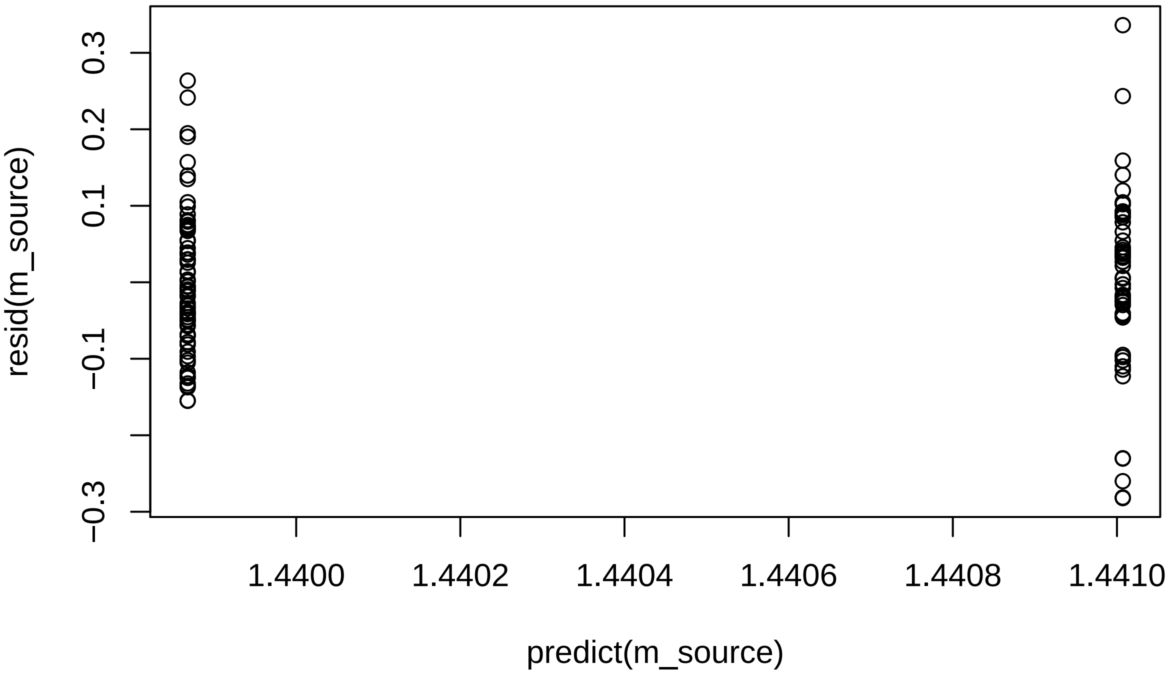


```
qqnorm(resid(m_source))
qqline(resid(m_source))
```

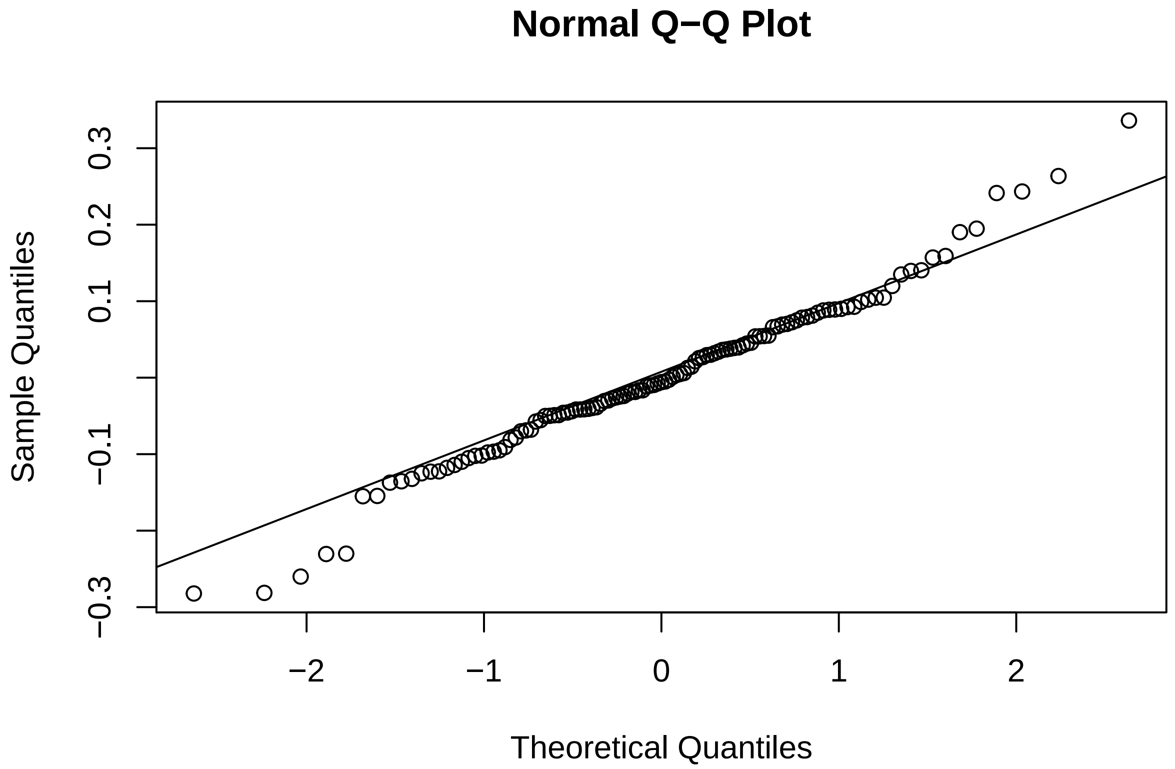

Looks fine.

Model summary

```r
summary(m_source)
```

```
##
## Call:
## lm(formula = rel.Vmax ~ source, data = salt)
##
## Residuals:
##      Min       1Q   Median       3Q      Max
## -0.28213 -0.05278 -0.00587  0.06826  0.33609
##
## Coefficients:
##              Estimate Std. Error t value Pr(>|t|)
## (Intercept)  1.441007   0.014919  96.586   <2e-16 ***
## sourceFood  -0.001139   0.019737  -0.058    0.954
## ---
## Signif. codes:  0 '***' 0.001 '**' 0.01 '*' 0.05 '.' 0.1 ' ' 1
##
## Residual standard error: 0.1065 on 117 degrees of freedom
## Multiple R-squared:  2.848e-05,  Adjusted R-squared:  -0.008518
## F-statistic: 0.003332 on 1 and 117 DF,  p-value: 0.9541
```

ANOVA

```r
anova(m_source)
```

```
## Analysis of Variance Table
##
## Response: rel.Vmax
##            Df  Sum Sq   Mean Sq F value Pr(>F)
## source      1 0.00004 0.0000378  0.0033 0.9541
## Residuals 117 1.32818 0.0113520
```

Nor does source.

Acid

David Kent (dk657)

11/21/2017

Contents

Method: 1
Questions: 1

Check out the data 1
Can we do a linear model? 16
Classifying into two groups? 18

K-means? 19
Adapted or not 33
Clonal Complex 36
Serotype 38
Lineage 39
Source 41
Tables of clusters 42

Method:

Define the survival under 1h acid stress. The strains were either adapted (adaption = 1h pH 5.5) prior to the acid stress at final pH, or not adapted= acid shock at the final pH (for HCl pH3.0 and for LA pH3.5). Three independent replicates, no technical replicates per run. The column "acid" defines the type of acid that was used (HCl or LA). The results are "CFU/ml".

Questions:

Does the adaption step lead to better survival? Is there a correlation between survival and CC? Is there a correlation between survival and source? Is there a correlation between survival and lineage? Is there a correlation between survival and serotype? Since there is such a clear pattern of sensitive/resistant, we would like to assign groups.

Check out the data

```
library(lme4)
```

```
## Loading required package: Matrix
```

```
library(lsmeans)
```

```
## The 'lsmeans' package is being deprecated.
## Users are encouraged to switch to 'emmeans'.
## See help('transition') for more information, including how
## to convert 'lsmeans' objects and scripts to work with 'emmeans'.
```

```r
library(ggplot2)
library(knitr)
library(kableExtra)
library(dplyr)
```

```
##
## Attaching package: 'dplyr'

## The following objects are masked from 'package:stats':
##
##     filter, lag

## The following objects are masked from 'package:base':
##
##     intersect, setdiff, setequal, union
```

```r
library(tidyr)
```

```
##
## Attaching package: 'tidyr'

## The following object is masked from 'package:Matrix':
##
##     expand
```

```r
library(scales)

# For plotting
inverse_logit_trans <- trans_new("inverse_logit",
                                 transform = plogis,
                                 inverse = qlogis)

# where there was 0 cfu/ml at t=60, set them to the limit of detection (100cfu/ml)
raw_acid <- read.csv("acid_Claudia.csv") %>%
  mutate(colony.count=ifelse(colony.count<1,0,colony.count)) %>%
  mutate(CFU.ml=100*colony.count/10^(dillution))

nrow(raw_acid)
```

```
## [1] 1046
```

```r
# calculate log-reduction
acid <- raw_acid %>%
  select(-colony.count,-dillution) %>%
  spread(key=time,value=CFU.ml) %>%
  mutate(logred=ifelse(t60==0,log10(t0/100),log10(t0/t60)),
         censored=t60==0) %>%
  na.exclude()

read.csv("hemolysis.csv") %>%
  select(clonal.complex,serotype,lineage,source,NENT.Nr.) %>%
  rename(NENT.Nr=NENT.Nr.) %>%
  distinct() -> strain_info

acid <- left_join(acid,strain_info) %>%
  filter(NENT.Nr!="10403s",
         NENT.Nr!="dSigB")
```

```
## Joining, by = c("clonal.complex", "serotype", "source", "lineage", "NENT.Nr")
```

```
acid %>%
  kable("latex", longtable = T, booktabs = T) %>%
  kable_styling(latex_options = c("repeat_header"), font_size = 7) %>%
  landscape()
```

replicate	date	adapted.not	acid	strain	clonal.complex	serotype	source	lineage	NENT.Nr	t0	t60	logred	censored
1	07.02.18	a	HCL	25	CC121	1/2a	Food	II	N11-1218	2.50e+09	1.60e+08	1.1938200	FALSE
1	07.02.18	a	HCL	26	CC121	1/2a	Food	II	N12-0571	3.50e+09	3.40e+08	1.0125891	FALSE
1	07.02.18	a	HCL	27	CC121	1/2a	Food	II	N13-0369	3.70e+09	0.00e+00	7.5682017	TRUE
1	07.02.18	a	HCL	28	CC121	1/2b	Food	II	N13-0836	2.60e+09	2.40e+08	1.0347621	FALSE
1	07.02.18	a	HCL	29	CC121	1/2a	Food	II	N14-0205	2.80e+09	1.70e+08	1.2167091	FALSE
1	07.02.18	a	HCL	30	CC121	3c	Food	II	N14-0322	2.10e+09	2.10e+08	1.0000000	FALSE
1	07.02.18	a	HCL	31	CC121	1/2a	Blood	II	N12-0367	1.50e+09	1.30e+04	5.0621479	FALSE
1	07.02.18	a	HCL	32	CC121	1/2a	Blood	II	N13-0119	3.10e+09	2.10e+08	1.1691424	FALSE
1	07.02.18	a	HCL	33	ST739	1/2a	Food	II	N11-2542	2.30e+09	1.80e+08	1.1064553	FALSE
1	07.02.18	a	HCL	34	ST28	1/2a	Food	II	N13-0288	2.70e+09	9.00e+03	5.4771213	FALSE
1	07.02.18	a	HCL	35	ST226	1/2a	Food	II	N13-2179	6.00e+09	1.60e+08	1.5740313	FALSE
1	07.02.18	a	HCL	36	CC31	1/2a	Food	II	N13-0228	5.00e+09	2.60e+05	4.2839967	FALSE
1	07.02.18	a	HCL	37	CC207	1/2a	Blood	II	N12-1107	2.60e+09	3.00e+04	4.9378521	FALSE
1	07.02.18	a	LA	25	CC121	1/2a	Food	II	N11-1218	2.10e+09	5.00e+08	0.6232493	FALSE
1	07.02.18	a	LA	26	CC121	1/2a	Food	II	N12-0571	2.90e+09	6.00e+08	0.6842467	FALSE
1	07.02.18	a	LA	27	CC121	1/2a	Food	II	N13-0369	2.10e+09	7.00e+02	6.4771213	FALSE
1	07.02.18	a	LA	28	CC121	1/2b	Food	II	N13-0836	2.70e+09	5.00e+08	0.7323938	FALSE
1	07.02.18	a	LA	29	CC121	1/2a	Food	II	N14-0205	3.80e+09	1.20e+09	0.5006024	FALSE
1	07.02.18	a	LA	30	CC121	3c	Food	II	N14-0322	2.10e+09	8.00e+08	0.4191293	FALSE
1	07.02.18	a	LA	31	CC121	1/2a	Blood	II	N12-0367	2.20e+09	1.50e+03	6.1663314	FALSE
1	07.02.18	a	LA	32	CC121	1/2a	Blood	II	N13-0119	1.08e+10	5.00e+08	1.3344538	FALSE
1	07.02.18	a	LA	33	ST739	1/2a	Food	II	N11-2542	3.50e+09	6.00e+08	0.7659168	FALSE
1	07.02.18	a	LA	34	ST28	1/2a	Food	II	N13-0288	3.40e+09	1.30e+05	4.4175356	FALSE
1	07.02.18	a	LA	35	ST226	1/2a	Food	II	N13-2179	2.60e+09	7.00e+08	0.5698753	FALSE
1	07.02.18	a	LA	36	CC31	1/2a	Food	II	N13-0228	3.80e+09	1.30e+06	3.4658402	FALSE
1	07.02.18	a	LA	37	CC207	1/2a	Blood	II	N12-1107	3.60e+09	2.20e+04	5.2138798	FALSE
1	07.02.18	n	HCL	25	CC121	1/2a	Food	II	N11-1218	4.00e+09	8.00e+08	0.6989700	FALSE
1	07.02.18	n	HCL	26	CC121	1/2a	Food	II	N12-0571	4.20e+09	6.00e+08	0.8450980	FALSE
1	07.02.18	n	HCL	27	CC121	1/2a	Food	II	N13-0369	3.40e+09	1.60e+04	5.3273589	FALSE
1	07.02.18	n	HCL	28	CC121	1/2b	Food	II	N13-0836	3.10e+09	9.00e+08	0.5371192	FALSE
1	07.02.18	n	HCL	29	CC121	1/2a	Food	II	N14-0205	2.40e+09	1.20e+09	0.3010300	FALSE
1	07.02.18	n	HCL	30	CC121	3c	Food	II	N14-0322	2.10e+09	6.00e+08	0.5440680	FALSE
1	07.02.18	n	HCL	31	CC121	1/2a	Blood	II	N12-0367	2.70e+09	1.40e+05	4.2852357	FALSE
1	07.02.18	n	HCL	32	CC121	1/2a	Blood	II	N13-0119	3.30e+09	6.00e+08	0.7403627	FALSE
1	07.02.18	n	HCL	33	ST739	1/2a	Food	II	N11-2542	3.30e+09	1.10e+09	0.4771213	FALSE
1	07.02.18	n	HCL	34	ST28	1/2a	Food	II	N13-0288	4.20e+09	0.00e+00	7.6232493	TRUE
1	07.02.18	n	HCL	35	ST226	1/2a	Food	II	N13-2179	7.10e+09	4.10e+08	1.2384745	FALSE
1	07.02.18	n	HCL	36	CC31	1/2a	Food	II	N13-0228	5.30e+09	0.00e+00	7.7242759	TRUE
1	07.02.18	n	HCL	37	CC207	1/2a	Blood	II	N12-1107	3.90e+09	1.00e+03	6.5910646	FALSE
1	07.02.18	n	LA	25	CC121	1/2a	Food	II	N11-1218	4.00e+09	1.40e+08	1.4559320	FALSE
1	07.02.18	n	LA	26	CC121	1/2a	Food	II	N12-0571	4.20e+09	2.80e+07	2.1760913	FALSE
1	07.02.18	n	LA	27	CC121	1/2a	Food	II	N13-0369	3.40e+09	2.00e+02	7.2304489	FALSE
1	07.02.18	n	LA	28	CC121	1/2b	Food	II	N13-0836	3.10e+09	8.00e+07	1.5882717	FALSE
1	07.02.18	n	LA	29	CC121	1/2a	Food	II	N14-0205	2.40e+09	2.13e+08	1.0518316	FALSE
1	07.02.18	n	LA	30	CC121	3c	Food	II	N14-0322	2.10e+09	1.81e+08	1.0645407	FALSE

(continued)

replicate	date	adapted.not	acid	strain	clonal.complex	serotype	source	lineage	NENT.Nr	t0	t60	logred	censored
1	07.02.18	n	LA	31	CC121	1/2a	Blood	II	N12-0367	2.70e+09	1.30e+03	6.3174204	FALSE
1	07.02.18	n	LA	32	CC121	1/2a	Blood	II	N13-0119	3.30e+09	8.00e+07	1.6154240	FALSE
1	07.02.18	n	LA	33	ST739	1/2a	Food	II	N11-2542	3.30e+09	1.50e+08	1.3424227	FALSE
1	07.02.18	n	LA	34	ST28	1/2a	Food	II	N13-0288	4.20e+09	3.00e+02	7.1461280	FALSE
1	07.02.18	n	LA	35	ST226	1/2a	Food	II	N13-2179	7.10e+09	8.00e+07	1.9481684	FALSE
1	07.02.18	n	LA	36	CC31	1/2a	Food	II	N13-0228	5.30e+09	3.00e+02	7.2471546	FALSE
1	07.02.18	n	LA	37	CC207	1/2a	Blood	II	N12-1107	3.90e+09	1.00e+02	7.5910646	FALSE
1	13.02.18	a	HCL	11	CC6	4b	Food	I	N13-0703	2.90e+09	0.00e+00	7.4623980	TRUE
1	13.02.18	a	HCL	12	CC6	4b	Food	I	N13-1184	1.80e+09	1.20e+04	5.1760913	FALSE
1	13.02.18	a	HCL	13	CC6	4b	Blood	I	N11-2801	4.50e+09	1.50e+03	6.4771213	FALSE
1	13.02.18	a	HCL	15	CC6	4b	Blood	I	N13-1271	2.30e+09	0.00e+00	7.3617278	TRUE
1	13.02.18	a	HCL	16	CC6	4b	Blood	I	N13-1507	4.70e+09	6.20e+07	1.8797062	FALSE
1	13.02.18	a	HCL	17	CC9	1/2c	Food	II	N11-1698	2.10e+09	0.00e+00	7.3222193	TRUE
1	13.02.18	a	HCL	19	CC9	1/2c	Food	II	N12-0822	3.70e+09	1.70e+08	1.3377528	FALSE
1	13.02.18	a	HCL	20	CC9	1/2c	Food	II	N11-1848	3.10e+09	1.30e+08	1.3774183	FALSE
1	13.02.18	a	HCL	21	CC9	1/2c	Food	II	N14-0261	3.20e+09	8.00e+08	0.6020600	FALSE
1	13.02.18	a	HCL	22	CC9	1/2a	Blood	II	N11-1837	3.00e+09	1.00e+02	7.4771213	FALSE
1	13.02.18	a	HCL	23	CC9	1/2c	Blood	II	N12-0486	2.90e+09	2.00e+02	7.1613680	FALSE
1	13.02.18	a	HCL	24	CC9	1/2c	Blood	II	N13-0001	4.50e+09	5.00e+02	6.9542425	FALSE
1	13.02.18	a	LA	11	CC6	4b	Food	I	N13-0703	2.50e+09	0.00e+00	7.3979400	TRUE
1	13.02.18	a	LA	12	CC6	4b	Food	I	N13-1184	2.00e+09	1.00e+02	7.3010300	FALSE
1	13.02.18	a	LA	13	CC6	4b	Blood	I	N11-2801	2.80e+09	0.00e+00	7.4471580	TRUE
1	13.02.18	a	LA	15	CC6	4b	Blood	I	N13-1271	2.10e+09	0.00e+00	7.3222193	TRUE
1	13.02.18	a	LA	16	CC6	4b	Blood	I	N13-1507	3.80e+09	1.10e+09	0.5383909	FALSE
1	13.02.18	a	LA	17	CC9	1/2c	Food	II	N11-1698	3.70e+09	3.00e+02	7.0910805	FALSE
1	13.02.18	a	LA	18	CC9	1/2c	Food	II	N12-0710	2.50e+09	1.80e+07	2.1426675	FALSE
1	13.02.18	a	LA	19	CC9	1/2c	Food	II	N12-0822	3.40e+09	8.00e+08	0.6283889	FALSE
1	13.02.18	a	LA	20	CC9	1/2c	Food	II	N11-1848	2.80e+09	1.00e+09	0.4471580	FALSE
1	13.02.18	a	LA	21	CC9	1/2c	Food	II	N14-0261	2.50e+09	1.50e+09	0.2218487	FALSE
1	13.02.18	a	LA	22	CC9	1/2a	Blood	II	N11-1837	3.00e+09	9.00e+02	6.5228787	FALSE
1	13.02.18	a	LA	23	CC9	1/2c	Blood	II	N12-0486	2.50e+09	1.90e+04	5.1191864	FALSE
1	13.02.18	a	LA	24	CC9	1/2c	Blood	II	N13-0001	3.40e+09	1.00e+02	7.5314789	FALSE
1	13.02.18	n	HCL	11	CC6	4b	Food	I	N13-0703	3.50e+09	1.30e+03	6.4301247	FALSE
1	13.02.18	n	HCL	12	CC6	4b	Food	I	N13-1184	3.10e+09	2.00e+03	6.1903317	FALSE
1	13.02.18	n	HCL	13	CC6	4b	Blood	I	N11-2801	4.80e+09	4.00e+02	7.0791812	FALSE
1	13.02.18	n	HCL	15	CC6	4b	Blood	I	N13-1271	5.10e+09	1.70e+03	6.4771213	FALSE
1	13.02.18	n	HCL	16	CC6	4b	Blood	I	N13-1507	4.50e+09	1.30e+08	1.5392692	FALSE
1	13.02.18	n	HCL	17	CC9	1/2c	Food	II	N11-1698	2.40e+09	1.30e+03	6.2662679	FALSE
1	13.02.18	n	HCL	19	CC9	1/2c	Food	II	N12-0822	3.70e+09	3.80e+08	0.9884181	FALSE
1	13.02.18	n	HCL	20	CC9	1/2c	Food	II	N11-1848	4.20e+09	3.50e+08	1.0791812	FALSE
1	13.02.18	n	HCL	21	CC9	1/2c	Food	II	N14-0261	2.90e+09	3.30e+08	0.9438841	FALSE
1	13.02.18	n	HCL	22	CC9	1/2a	Blood	II	N11-1837	3.70e+09	1.00e+02	7.5682017	FALSE
1	13.02.18	n	HCL	23	CC9	1/2c	Blood	II	N12-0486	2.90e+09	2.00e+02	7.1613680	FALSE

(continued)

replicate	date	adapted.not	acid	strain	clonal.complex	serotype	source	lineage	NENT.Nr	t0	t60	logred	censored
1	13.02.18	n	HCL	24	CC9	1/2c	Blood	II	N13-0001	3.80e+09	0.00e+00	7.5797836	TRUE
1	13.02.18	n	LA	11	CC6	4b	Food	I	N13-0703	3.50e+09	0.00e+00	7.5440680	TRUE
1	13.02.18	n	LA	12	CC6	4b	Food	I	N13-1184	3.10e+09	0.00e+00	7.4913617	TRUE
1	13.02.18	n	LA	13	CC6	4b	Blood	I	N11-2801	4.80e+09	0.00e+00	7.6812412	TRUE
1	13.02.18	n	LA	15	CC6	4b	Blood	I	N13-1271	5.10e+09	0.00e+00	7.7075702	TRUE
1	13.02.18	n	LA	16	CC6	4b	Blood	I	N13-1507	4.50e+09	4.20e+07	2.0299632	FALSE
1	13.02.18	n	LA	17	CC9	1/2c	Food	II	N11-1698	2.40e+09	0.00e+00	7.3802112	TRUE
1	13.02.18	n	LA	19	CC9	1/2c	Food	II	N12-0822	3.70e+09	2.40e+09	0.1879905	FALSE
1	13.02.18	n	LA	20	CC9	1/2c	Food	II	N11-1848	4.20e+09	2.00e+07	2.3222193	FALSE
1	13.02.18	n	LA	21	CC9	1/2c	Food	II	N14-0261	2.90e+09	5.30e+08	0.7381221	FALSE
1	13.02.18	n	LA	22	CC9	1/2a	Blood	II	N11-1837	3.70e+09	0.00e+00	7.5682017	TRUE
1	13.02.18	n	LA	23	CC9	1/2c	Blood	II	N12-0486	2.90e+09	1.00e+02	7.4623980	FALSE
1	13.02.18	n	LA	24	CC9	1/2c	Blood	II	N13-0001	3.80e+09	0.00e+00	7.5797836	TRUE
1	14.02.18	a	HCL	1	CC1	4b	Food	I	N12-0605	3.80e+09	6.00e+07	1.8016323	FALSE
1	14.02.18	a	HCL	10	CC4	4b	Food	I	N12-1772	3.30e+09	2.00e+02	7.2174839	FALSE
1	14.02.18	a	HCL	14	CC6	4b	Blood	I	N12-1387	5.00e+08	3.10e+04	4.2076083	FALSE
1	14.02.18	a	HCL	2	CC1	4b	Food	I	N12-1339	4.40e+09	6.00e+07	1.8653014	FALSE
1	14.02.18	a	HCL	3	CC1	4b	Food	I	N12-1996	3.70e+09	1.30e+07	2.4542584	FALSE
1	14.02.18	a	HCL	38	CC415	1/2b	Blood	II	N13-0762	3.90e+09	2.60e+05	4.1760913	FALSE
1	14.02.18	a	HCL	39	CC54	4b	Blood	I	N13-0177	3.80e+09	2.70e+07	2.1484198	FALSE
1	14.02.18	a	HCL	4	CC1	4b	Food	I	N13-0047	4.40e+09	2.00e+03	6.3424227	FALSE
1	14.02.18	a	HCL	40	CC224	1/2b	Blood	I	N12-1608	3.50e+09	6.00e+06	2.7659168	FALSE
1	14.02.18	a	HCL	5	CC1	4b	Blood	I	N11-2292	4.40e+09	4.50e+07	1.9902402	FALSE
1	14.02.18	a	HCL	6	CC1	4b	Blood	I	LL195	2.90e+09	5.60e+07	1.7142100	FALSE
1	14.02.18	a	HCL	7	CC1	4b	Blood	I	N13-0987	3.30e+09	8.00e+02	6.6154240	FALSE
1	14.02.18	a	HCL	8	CC1	4b	Blood	I	N13-1079	3.90e+09	2.10e+03	6.2688453	FALSE
1	14.02.18	a	HCL	9	CC6	4b	Food	I	N12-0460	4.00e+09	1.90e+07	2.3233064	FALSE
1	14.02.18	a	LA	1	CC1	4b	Food	I	N12-0605	3.60e+09	1.00e+08	1.5563025	FALSE
1	14.02.18	a	LA	10	CC4	4b	Food	I	N12-1772	4.00e+09	0.00e+00	7.6020600	TRUE
1	14.02.18	a	LA	14	CC6	4b	Blood	I	N12-1387	6.00e+08	0.00e+00	6.7781513	TRUE
1	14.02.18	a	LA	2	CC1	4b	Food	I	N12-1339	1.10e+10	2.80e+08	1.5942347	FALSE
1	14.02.18	a	LA	3	CC1	4b	Food	I	N12-1996	3.60e+09	2.30e+08	1.1945747	FALSE
1	14.02.18	a	LA	38	CC415	1/2b	Blood	II	N13-0762	3.30e+09	0.00e+00	7.5185139	TRUE
1	14.02.18	a	LA	39	CC54	4b	Blood	I	N13-0177	3.50e+09	8.00e+08	0.6409781	FALSE
1	14.02.18	a	LA	4	CC1	4b	Food	I	N13-0047	3.20e+09	7.00e+02	6.6600519	FALSE
1	14.02.18	a	LA	40	CC224	1/2b	Blood	I	N12-1608	3.80e+09	2.90e+08	1.1173856	FALSE
1	14.02.18	a	LA	5	CC1	4b	Blood	I	N11-2292	4.10e+09	2.30e+08	1.2510560	FALSE
1	14.02.18	a	LA	6	CC1	4b	Blood	I	LL195	3.70e+09	1.60e+08	1.3640817	FALSE
1	14.02.18	a	LA	7	CC1	4b	Blood	I	N13-0987	4.40e+09	0.00e+00	7.6434527	TRUE
1	14.02.18	a	LA	8	CC1	4b	Blood	I	N13-1079	3.10e+09	0.00e+00	7.4913617	TRUE
1	14.02.18	a	LA	9	CC6	4b	Food	I	N12-0460	3.20e+09	4.90e+07	1.8149539	FALSE
1	14.02.18	n	HCL	1	CC1	4b	Food	I	N12-0605	3.90e+09	2.20e+08	1.2486419	FALSE
1	14.02.18	n	HCL	10	CC4	4b	Food	I	N12-1772	3.30e+09	0.00e+00	7.5185139	TRUE

(continued)

replicate	date	adapted.not	acid	strain	clonal.complex	serotype	source	lineage	NENT.Nr	t0	t60	logred	censored
1	14.02.18	n	HCL	14	CC6	4b	Blood	I	N12-1387	8.00e+08	0.00e+00	6.9030900	TRUE
1	14.02.18	n	HCL	2	CC1	4b	Food	I	N12-1339	3.40e+09	2.90e+08	1.0690809	FALSE
1	14.02.18	n	HCL	3	CC1	4b	Food	I	N12-1996	3.50e+09	1.00e+08	1.5440680	FALSE
1	14.02.18	n	HCL	38	CC415	1/2b	Blood	II	N13-0762	4.10e+09	4.00e+02	7.0107239	FALSE
1	14.02.18	n	HCL	39	CC54	4b	Blood	I	N13-0177	3.80e+09	2.30e+08	1.2180558	FALSE
1	14.02.18	n	HCL	4	CC1	4b	Food	I	N13-0047	3.50e+09	2.80e+03	6.0969100	FALSE
1	14.02.18	n	HCL	40	CC224	1/2b	Blood	I	N12-1608	4.30e+09	6.00e+06	2.8553172	FALSE
1	14.02.18	n	HCL	5	CC1	4b	Blood	I	N11-2292	3.30e+09	3.20e+08	1.0133640	FALSE
1	14.02.18	n	HCL	6	CC1	4b	Blood	I	LL195	1.80e+09	3.80e+08	0.6754889	FALSE
1	14.02.18	n	HCL	7	CC1	4b	Blood	I	N13-0987	1.10e+09	1.00e+03	6.0413927	FALSE
1	14.02.18	n	HCL	8	CC1	4b	Blood	I	N13-1079	2.10e+09	3.00e+03	5.8450980	FALSE
1	14.02.18	n	HCL	9	CC6	4b	Food	I	N12-0460	3.40e+09	8.00e+07	1.6283889	FALSE
1	14.02.18	n	LA	1	CC1	4b	Food	I	N12-0605	3.90e+09	5.00e+07	1.8920946	FALSE
1	14.02.18	n	LA	10	CC4	4b	Food	I	N12-1772	3.30e+09	0.00e+00	7.5185139	TRUE
1	14.02.18	n	LA	14	CC6	4b	Blood	I	N12-1387	8.00e+08	0.00e+00	6.9030900	TRUE
1	14.02.18	n	LA	2	CC1	4b	Food	I	N12-1339	3.40e+09	9.00e+07	1.5772364	FALSE
1	14.02.18	n	LA	3	CC1	4b	Food	I	N12-1996	3.50e+09	2.30e+07	2.1823402	FALSE
1	14.02.18	n	LA	38	CC415	1/2b	Blood	II	N13-0762	4.10e+09	0.00e+00	7.6127839	TRUE
1	14.02.18	n	LA	39	CC54	4b	Blood	I	N13-0177	3.80e+09	4.30e+08	0.9463151	FALSE
1	14.02.18	n	LA	4	CC1	4b	Food	I	N13-0047	3.50e+09	8.00e+02	6.6409781	FALSE
1	14.02.18	n	LA	40	CC224	1/2b	Blood	I	N12-1608	4.30e+09	2.40e+07	2.2532572	FALSE
1	14.02.18	n	LA	5	CC1	4b	Blood	I	N11-2292	3.30e+09	2.30e+08	1.1567861	FALSE
1	14.02.18	n	LA	6	CC1	4b	Blood	I	LL195	1.80e+09	2.90e+08	0.7928745	FALSE
1	14.02.18	n	LA	7	CC1	4b	Blood	I	N13-0987	1.10e+09	0.00e+00	7.0413927	TRUE
1	14.02.18	n	LA	8	CC1	4b	Blood	I	N13-1079	2.10e+09	0.00e+00	7.3222193	TRUE
1	14.02.18	n	LA	9	CC6	4b	Food	I	N12-0460	3.40e+09	4.60e+06	2.8687211	FALSE
2	08.02.18	a	HCL	25	CC121	1/2a	Food	II	N11-1218	2.50e+09	1.60e+08	1.1938200	FALSE
2	08.02.18	a	HCL	26	CC121	1/2a	Food	II	N12-0571	4.20e+09	1.90e+08	1.3444957	FALSE
2	08.02.18	a	HCL	27	CC121	1/2a	Food	II	N13-0369	4.80e+09	6.00e+02	6.9030900	FALSE
2	08.02.18	a	HCL	29	CC121	1/2a	Food	II	N14-0205	3.80e+09	1.40e+08	1.4336556	FALSE
2	08.02.18	a	HCL	30	CC121	3c	Food	II	N14-0322	2.90e+09	2.00e+08	1.1613680	FALSE
2	08.02.18	a	HCL	31	CC121	1/2a	Blood	II	N12-0367	3.20e+09	1.10e+03	6.4637573	FALSE
2	08.02.18	a	HCL	32	CC121	1/2a	Blood	II	N13-0119	3.70e+09	7.00e+07	1.7231037	FALSE
2	08.02.18	a	HCL	33	ST739	1/2a	Food	II	N11-2542	2.70e+09	7.00e+07	1.5862657	FALSE
2	08.02.18	a	HCL	34	ST28	1/2a	Food	II	N13-0288	2.50e+09	1.80e+06	3.1426675	FALSE
2	08.02.18	a	HCL	35	ST226	1/2a	Food	II	N13-2179	2.70e+09	1.10e+08	1.3899711	FALSE
2	08.02.18	a	HCL	36	CC31	1/2a	Food	II	N13-0228	2.20e+09	1.00e+06	3.3424227	FALSE
2	08.02.18	a	HCL	37	CC207	1/2a	Blood	II	N12-1107	3.00e+09	1.80e+05	4.2218487	FALSE
2	08.02.18	a	LA	25	CC121	1/2a	Food	II	N11-1218	3.00e+09	9.00e+07	1.5228787	FALSE
2	08.02.18	a	LA	26	CC121	1/2a	Food	II	N12-0571	4.50e+09	1.10e+08	1.6118198	FALSE
2	08.02.18	a	LA	27	CC121	1/2a	Food	II	N13-0369	3.00e+09	3.20e+03	5.9719713	FALSE
2	08.02.18	a	LA	29	CC121	1/2a	Food	II	N14-0205	3.60e+09	3.20e+08	1.0511525	FALSE
2	08.02.18	a	LA	30	CC121	3c	Food	II	N14-0322	4.40e+09	2.20e+08	1.3010300	FALSE
2	08.02.18	a	LA	31	CC121	1/2a	Blood	II	N12-0367	3.70e+09	0.00e+00	7.5682017	TRUE

(continued)

replicate	date	adapted.not	acid	strain	clonal.complex	serotype	source	lineage	NENT.Nr	t0	t60	logred	censored
2	08.02.18	a	LA	32	CC121	1/2a	Blood	II	N13-0119	2.90e+09	2.50e+08	1.0644580	FALSE
2	08.02.18	a	LA	33	ST739	1/2a	Food	II	N11-2542	3.60e+09	8.00e+07	1.6532125	FALSE
2	08.02.18	a	LA	34	ST28	1/2a	Food	II	N13-0288	3.80e+09	1.00e+02	7.5797836	FALSE
2	08.02.18	a	LA	35	ST226	1/2a	Food	II	N13-2179	4.10e+09	3.30e+07	2.0942699	FALSE
2	08.02.18	a	LA	36	CC31	1/2a	Food	II	N13-0228	2.10e+09	0.00e+00	7.3222193	TRUE
2	08.02.18	a	LA	37	CC207	1/2a	Blood	II	N12-1107	1.60e+09	0.00e+00	7.2041200	TRUE
2	08.02.18	n	HCL	25	CC121	1/2a	Food	II	N11-1218	3.50e+09	4.10e+08	0.9312842	FALSE
2	08.02.18	n	HCL	26	CC121	1/2a	Food	II	N12-0571	3.60e+09	6.00e+07	1.7781513	FALSE
2	08.02.18	n	HCL	27	CC121	1/2a	Food	II	N13-0369	4.00e+09	0.00e+00	7.6020600	TRUE
2	08.02.18	n	HCL	29	CC121	1/2a	Food	II	N14-0205	3.20e+09	4.00e+08	0.9030900	FALSE
2	08.02.18	n	HCL	30	CC121	3c	Food	II	N14-0322	4.40e+09	2.20e+08	1.3010300	FALSE
2	08.02.18	n	HCL	31	CC121	1/2a	Blood	II	N12-0367	3.80e+09	0.00e+00	7.5797836	TRUE
2	08.02.18	n	HCL	32	CC121	1/2a	Blood	II	N13-0119	4.90e+09	3.20e+07	2.1850461	FALSE
2	08.02.18	n	HCL	33	ST739	1/2a	Food	II	N11-2542	4.60e+09	6.00e+07	1.8846066	FALSE
2	08.02.18	n	HCL	34	ST28	1/2a	Food	II	N13-0288	4.20e+09	0.00e+00	7.6232493	TRUE
2	08.02.18	n	HCL	35	ST226	1/2a	Food	II	N13-2179	4.30e+09	3.00e+07	2.1563472	FALSE
2	08.02.18	n	HCL	36	CC31	1/2a	Food	II	N13-0228	3.40e+09	0.00e+00	7.5314789	TRUE
2	08.02.18	n	HCL	37	CC207	1/2a	Blood	II	N12-1107	2.80e+09	0.00e+00	7.4471580	TRUE
2	08.02.18	n	LA	25	CC121	1/2a	Food	II	N11-1218	3.50e+09	2.20e+05	4.2016454	FALSE
2	08.02.18	n	LA	26	CC121	1/2a	Food	II	N12-0571	3.60e+09	3.70e+05	3.9881008	FALSE
2	08.02.18	n	LA	27	CC121	1/2a	Food	II	N13-0369	4.00e+09	0.00e+00	7.6020600	TRUE
2	08.02.18	n	LA	29	CC121	1/2a	Food	II	N14-0205	3.20e+09	2.20e+07	2.1627273	FALSE
2	08.02.18	n	LA	30	CC121	3c	Food	II	N14-0322	4.40e+09	7.00e+06	2.7983546	FALSE
2	08.02.18	n	LA	31	CC121	1/2a	Blood	II	N12-0367	3.80e+09	0.00e+00	7.5797836	TRUE
2	08.02.18	n	LA	32	CC121	1/2a	Blood	II	N13-0119	4.90e+09	2.10e+05	4.3679768	FALSE
2	08.02.18	n	LA	34	ST28	1/2a	Food	II	N13-0288	4.20e+09	0.00e+00	7.6232493	TRUE
2	08.02.18	n	LA	36	CC31	1/2a	Food	II	N13-0228	3.40e+09	0.00e+00	7.5314789	TRUE
2	08.02.18	n	LA	37	CC207	1/2a	Blood	II	N12-1107	2.80e+09	0.00e+00	7.4471580	TRUE
2	15.02.18	a	HCL	1	CC1	4b	Food	I	N12-0605	2.90e+09	1.20e+08	1.3832168	FALSE
2	15.02.18	a	HCL	10	CC4	4b	Food	I	N12-1772	3.60e+09	0.00e+00	7.5563025	TRUE
2	15.02.18	a	HCL	14	CC6	4b	Blood	I	N12-1387	1.60e+09	0.00e+00	7.2041200	TRUE
2	15.02.18	a	HCL	2	CC1	4b	Food	I	N12-1339	4.20e+09	3.50e+08	1.0791812	FALSE
2	15.02.18	a	HCL	3	CC1	4b	Food	I	N12-1996	2.20e+09	9.00e+07	1.3881802	FALSE
2	15.02.18	a	HCL	38	CC415	1/2b	Blood	II	N13-0762	1.70e+09	6.00e+02	6.4522977	FALSE
2	15.02.18	a	HCL	39	CC54	4b	Blood	I	N13-0177	3.50e+09	5.00e+07	1.8450980	FALSE
2	15.02.18	a	HCL	4	CC1	4b	Food	I	N13-0047	3.60e+09	1.60e+04	5.3521825	FALSE
2	15.02.18	a	HCL	40	CC224	1/2b	Blood	I	N12-1608	1.00e+09	2.30e+08	0.6382722	FALSE
2	15.02.18	a	HCL	5	CC1	4b	Blood	I	N11-2292	5.20e+09	1.50e+08	1.5399121	FALSE
2	15.02.18	a	HCL	6	CC1	4b	Blood	I	LL195	4.10e+09	1.80e+08	1.3575114	FALSE
2	15.02.18	a	HCL	7	CC1	4b	Blood	I	N13-0987	3.50e+09	3.00e+02	7.0669468	FALSE
2	15.02.18	a	HCL	8	CC1	4b	Blood	I	N13-1079	3.20e+09	7.00e+02	6.6600519	FALSE
2	15.02.18	a	HCL	9	CC6	4b	Food	I	N12-0460	3.40e+09	1.80e+07	2.2762064	FALSE
2	15.02.18	a	LA	1	CC1	4b	Food	I	N12-0605	3.90e+09	2.20e+09	0.2486419	FALSE

(continued)

replicate	date	adapted.not	acid	strain	clonal.complex	serotype	source	lineage	NENT.Nr	t0	t60	logred	censored
2	15.02.18	a	LA	10	CC4	4b	Food	I	N12-1772	1.80e+09	1.00e+03	6.2552725	FALSE
2	15.02.18	a	LA	14	CC6	4b	Blood	I	N12-1387	1.20e+09	0.00e+00	7.0791812	TRUE
2	15.02.18	a	LA	2	CC1	4b	Food	I	N12-1339	4.10e+09	1.60e+09	0.4086639	FALSE
2	15.02.18	a	LA	3	CC1	4b	Food	I	N12-1996	3.30e+09	9.00e+08	0.5642714	FALSE
2	15.02.18	a	LA	38	CC415	1/2b	Blood	II	N13-0762	2.50e+09	9.00e+03	5.4436975	FALSE
2	15.02.18	a	LA	39	CC54	4b	Blood	I	N13-0177	2.50e+09	7.60e+08	0.5171264	FALSE
2	15.02.18	a	LA	4	CC1	4b	Food	I	N13-0047	3.00e+09	2.20e+04	5.1346986	FALSE
2	15.02.18	a	LA	40	CC224	1/2b	Blood	I	N12-1608	1.40e+09	4.00e+08	0.5440680	FALSE
2	15.02.18	a	LA	5	CC1	4b	Blood	I	N11-2292	2.30e+09	1.60e+09	0.1576079	FALSE
2	15.02.18	a	LA	6	CC1	4b	Blood	I	LL195	3.20e+09	1.30e+09	0.3912066	FALSE
2	15.02.18	a	LA	7	CC1	4b	Blood	I	N13-0987	2.60e+09	7.00e+02	6.5698753	FALSE
2	15.02.18	a	LA	8	CC1	4b	Blood	I	N13-1079	4.30e+09	1.20e+04	5.5542872	FALSE
2	15.02.18	a	LA	9	CC6	4b	Food	I	N12-0460	3.30e+09	1.00e+09	0.5185139	FALSE
2	15.02.18	n	HCL	1	CC1	4b	Food	I	N12-0605	3.10e+09	2.90e+08	1.0289637	FALSE
2	15.02.18	n	HCL	10	CC4	4b	Food	I	N12-1772	2.70e+09	1.00e+02	7.4313638	FALSE
2	15.02.18	n	HCL	14	CC6	4b	Blood	I	N12-1387	1.50e+09	4.00e+02	6.5740313	FALSE
2	15.02.18	n	HCL	2	CC1	4b	Food	I	N12-1339	2.70e+09	6.00e+08	0.6532125	FALSE
2	15.02.18	n	HCL	3	CC1	4b	Food	I	N12-1996	3.10e+09	1.70e+08	1.2609128	FALSE
2	15.02.18	n	HCL	38	CC415	1/2b	Blood	II	N13-0762	4.90e+09	0.00e+00	7.6901961	TRUE
2	15.02.18	n	HCL	39	CC54	4b	Blood	I	N13-0177	7.50e+09	1.10e+08	1.8336686	FALSE
2	15.02.18	n	HCL	4	CC1	4b	Food	I	N13-0047	2.80e+09	9.00e+02	6.4929155	FALSE
2	15.02.18	n	HCL	40	CC224	1/2b	Blood	I	N12-1608	1.40e+09	1.10e+08	1.1047354	FALSE
2	15.02.18	n	HCL	5	CC1	4b	Blood	I	N11-2292	4.00e+09	2.50e+08	1.2041200	FALSE
2	15.02.18	n	HCL	6	CC1	4b	Blood	I	LL195	2.20e+09	7.00e+08	0.4973246	FALSE
2	15.02.18	n	HCL	7	CC1	4b	Blood	I	N13-0987	2.60e+09	0.00e+00	7.4149733	TRUE
2	15.02.18	n	HCL	8	CC1	4b	Blood	I	N13-1079	3.60e+09	3.30e+03	6.0377886	FALSE
2	15.02.18	n	HCL	9	CC6	4b	Food	I	N12-0460	3.20e+09	1.60e+07	2.3010300	FALSE
2	15.02.18	n	LA	1	CC1	4b	Food	I	N12-0605	3.10e+09	3.00e+08	1.0142404	FALSE
2	15.02.18	n	LA	10	CC4	4b	Food	I	N12-1772	2.70e+09	0.00e+00	7.4313638	TRUE
2	15.02.18	n	LA	14	CC6	4b	Blood	I	N12-1387	1.50e+09	0.00e+00	7.1760913	TRUE
2	15.02.18	n	LA	2	CC1	4b	Food	I	N12-1339	2.70e+09	1.10e+09	0.3899711	FALSE
2	15.02.18	n	LA	3	CC1	4b	Food	I	N12-1996	3.10e+09	7.00e+08	0.6462637	FALSE
2	15.02.18	n	LA	38	CC415	1/2b	Blood	II	N13-0762	4.90e+09	1.00e+03	6.6901961	FALSE
2	15.02.18	n	LA	39	CC54	4b	Blood	I	N13-0177	7.50e+09	8.00e+08	0.9719713	FALSE
2	15.02.18	n	LA	4	CC1	4b	Food	I	N13-0047	2.80e+09	0.00e+00	7.4471580	TRUE
2	15.02.18	n	LA	40	CC224	1/2b	Blood	I	N12-1608	1.40e+09	2.40e+08	0.7659168	FALSE
2	15.02.18	n	LA	5	CC1	4b	Blood	I	N11-2292	4.00e+09	1.00e+09	0.6020600	FALSE
2	15.02.18	n	LA	6	CC1	4b	Blood	I	LL195	2.20e+09	9.00e+08	0.3881802	FALSE
2	15.02.18	n	LA	7	CC1	4b	Blood	I	N13-0987	2.60e+09	4.00e+02	6.8129134	FALSE
2	15.02.18	n	LA	8	CC1	4b	Blood	I	N13-1079	3.60e+09	9.00e+03	5.6020600	FALSE
2	15.02.18	n	LA	9	CC6	4b	Food	I	N12-0460	3.20e+09	1.30e+08	1.3912066	FALSE
2	31.01.18	a	HCL	11	CC6	4b	Food	I	N13-0703	2.60e+09	8.00e+03	5.5118834	FALSE
2	31.01.18	a	HCL	12	CC6	4b	Food	I	N13-1184	3.70e+09	1.40e+04	5.4220737	FALSE

(continued)

replicate	date	adapted.not	acid	strain	clonal.complex	serotype	source	lineage	NENT.Nr	t0	t60	logred	censored
2	31.01.18	a	HCL	13	CC6	4b	Blood	I	N11-2801	4.10e+09	1.20e+04	5.5336026	FALSE
2	31.01.18	a	HCL	15	CC6	4b	Blood	I	N13-1271	3.20e+09	1.50e+04	5.3290587	FALSE
2	31.01.18	a	HCL	16	CC6	4b	Blood	I	N13-1507	4.20e+09	5.50e+07	1.8828866	FALSE
2	31.01.18	a	HCL	17	CC9	1/2c	Food	II	N11-1698	3.10e+09	2.30e+04	5.1296339	FALSE
2	31.01.18	a	HCL	18	CC9	1/2c	Food	II	N12-0710	2.20e+09	2.90e+07	1.8800247	FALSE
2	31.01.18	a	HCL	19	CC9	1/2c	Food	II	N12-0822	2.50e+09	2.30e+08	1.0362122	FALSE
2	31.01.18	a	HCL	20	CC9	1/2c	Food	II	N11-1848	3.00e+09	1.60e+08	1.2730013	FALSE
2	31.01.18	a	HCL	21	CC9	1/2c	Food	II	N14-0261	1.50e+09	3.30e+08	0.6575773	FALSE
2	31.01.18	a	HCL	23	CC9	1/2c	Blood	II	N12-0486	2.40e+09	4.50e+05	3.7269987	FALSE
2	31.01.18	a	HCL	24	CC9	1/2c	Blood	II	N13-0001	4.90e+09	1.10e+06	3.6488034	FALSE
2	31.01.18	a	LA	11	CC6	4b	Food	I	N13-0703	1.10e+09	4.00e+03	5.4393327	FALSE
2	31.01.18	a	LA	12	CC6	4b	Food	I	N13-1184	3.60e+09	3.30e+03	6.0377886	FALSE
2	31.01.18	a	LA	13	CC6	4b	Blood	I	N11-2801	1.80e+09	1.80e+03	6.0000000	FALSE
2	31.01.18	a	LA	15	CC6	4b	Blood	I	N13-1271	1.90e+09	1.50e+03	6.1026623	FALSE
2	31.01.18	a	LA	16	CC6	4b	Blood	I	N13-1507	3.20e+09	6.40e+08	0.6989700	FALSE
2	31.01.18	a	LA	17	CC9	1/2c	Food	II	N11-1698	1.40e+09	3.60e+03	5.5898255	FALSE
2	31.01.18	a	LA	18	CC9	1/2c	Food	II	N12-0710	2.80e+09	5.80e+08	0.6837300	FALSE
2	31.01.18	a	LA	19	CC9	1/2c	Food	II	N12-0822	3.40e+09	5.30e+08	0.8072030	FALSE
2	31.01.18	a	LA	20	CC9	1/2c	Food	II	N11-1848	3.00e+09	1.70e+09	0.2466723	FALSE
2	31.01.18	a	LA	21	CC9	1/2c	Food	II	N14-0261	3.00e+09	6.00e+08	0.6989700	FALSE
2	31.01.18	a	LA	22	CC9	1/2a	Blood	II	N11-1837	3.50e+09	0.00e+00	7.5440680	TRUE
2	31.01.18	a	LA	23	CC9	1/2c	Blood	II	N12-0486	3.00e+09	2.10e+04	5.1549020	FALSE
2	31.01.18	a	LA	24	CC9	1/2c	Blood	II	N13-0001	2.50e+09	2.80e+03	5.9507820	FALSE
2	31.01.18	n	HCL	11	CC6	4b	Food	I	N13-0703	2.60e+09	9.00e+04	4.4607308	FALSE
2	31.01.18	n	HCL	12	CC6	4b	Food	I	N13-1184	3.80e+09	0.00e+00	7.5797836	TRUE
2	31.01.18	n	HCL	13	CC6	4b	Blood	I	N11-2801	3.80e+09	2.00e+03	6.2787536	FALSE
2	31.01.18	n	HCL	15	CC6	4b	Blood	I	N13-1271	5.50e+09	0.00e+00	7.7403627	TRUE
2	31.01.18	n	HCL	16	CC6	4b	Blood	I	N13-1507	2.60e+09	2.00e+08	1.1139434	FALSE
2	31.01.18	n	HCL	17	CC9	1/2c	Food	II	N11-1698	3.30e+09	0.00e+00	7.5185139	TRUE
2	31.01.18	n	HCL	18	CC9	1/2c	Food	II	N12-0710	2.50e+09	2.30e+05	4.0362122	FALSE
2	31.01.18	n	HCL	19	CC9	1/2c	Food	II	N12-0822	2.00e+09	2.30e+08	0.9393022	FALSE
2	31.01.18	n	HCL	20	CC9	1/2c	Food	II	N11-1848	3.20e+09	1.40e+08	1.3590219	FALSE
2	31.01.18	n	HCL	21	CC9	1/2c	Food	II	N14-0261	2.20e+09	1.90e+08	1.0636691	FALSE
2	31.01.18	n	HCL	22	CC9	1/2a	Blood	II	N11-1837	2.40e+09	0.00e+00	7.3802112	TRUE
2	31.01.18	n	HCL	23	CC9	1/2c	Blood	II	N12-0486	2.10e+09	0.00e+00	7.3222193	TRUE
2	31.01.18	n	HCL	24	CC9	1/2c	Blood	II	N13-0001	3.10e+09	0.00e+00	7.4913617	TRUE
2	31.01.18	n	LA	11	CC6	4b	Food	I	N13-0703	2.60e+09	0.00e+00	7.4149733	TRUE
2	31.01.18	n	LA	12	CC6	4b	Food	I	N13-1184	3.80e+09	2.70e+03	6.1484198	FALSE
2	31.01.18	n	LA	13	CC6	4b	Blood	I	N11-2801	3.80e+09	1.00e+04	5.5797836	FALSE
2	31.01.18	n	LA	15	CC6	4b	Blood	I	N13-1271	5.50e+09	1.10e+03	6.6989700	FALSE
2	31.01.18	n	LA	16	CC6	4b	Blood	I	N13-1507	2.60e+09	5.90e+08	0.6441213	FALSE
2	31.01.18	n	LA	17	CC9	1/2c	Food	II	N11-1698	3.30e+09	8.00e+03	5.6154240	FALSE
2	31.01.18	n	LA	18	CC9	1/2c	Food	II	N12-0710	2.50e+09	5.40e+08	0.6655462	FALSE
2	31.01.18	n	LA	19	CC9	1/2c	Food	II	N12-0822	2.00e+09	1.70e+09	0.0705811	FALSE

(continued)

replicate	date	adapted.not	acid	strain	clonal.complex	serotype	source	lineage	NENT.Nr	t0	t60	logred	censored
2	31.01.18	n	LA	20	CC9	1/2c	Food	II	N11-1848	3.20e+09	1.10e+09	0.4637573	FALSE
2	31.01.18	n	LA	21	CC9	1/2c	Food	II	N14-0261	2.20e+09	2.50e+09	-0.0555173	FALSE
2	31.01.18	n	LA	23	CC9	1/2c	Blood	II	N12-0486	2.10e+09	3.10e+05	3.8308576	FALSE
2	31.01.18	n	LA	24	CC9	1/2c	Blood	II	N13-0001	3.10e+09	2.10e+04	5.1691424	FALSE
3	06.02.18	a	HCL	11	CC6	4b	Food	I	N13-0703	3.60e+09	0.00e+00	7.5563025	TRUE
3	06.02.18	a	HCL	12	CC6	4b	Food	I	N13-1184	1.80e+09	4.50e+06	2.6020600	FALSE
3	06.02.18	a	HCL	13	CC6	4b	Blood	I	N11-2801	3.30e+09	0.00e+00	7.5185139	TRUE
3	06.02.18	a	HCL	15	CC6	4b	Blood	I	N13-1271	4.80e+09	1.00e+02	7.6812412	FALSE
3	06.02.18	a	HCL	16	CC6	4b	Blood	I	N13-1507	2.80e+09	2.00e+07	2.1461280	FALSE
3	06.02.18	a	HCL	17	CC9	1/2c	Food	II	N11-1698	2.00e+09	0.00e+00	7.3010300	TRUE
3	06.02.18	a	HCL	18	CC9	1/2c	Food	II	N12-0710	2.20e+09	3.10e+07	1.8510610	FALSE
3	06.02.18	a	HCL	19	CC9	1/2c	Food	II	N12-0822	2.20e+09	3.00e+07	1.8653014	FALSE
3	06.02.18	a	HCL	20	CC9	1/2c	Food	II	N11-1848	3.60e+09	1.50e+08	1.3802112	FALSE
3	06.02.18	a	HCL	21	CC9	1/2c	Food	II	N14-0261	2.20e+09	2.10e+08	1.0202034	FALSE
3	06.02.18	a	HCL	22	CC9	1/2a	Blood	II	N11-1837	4.20e+09	0.00e+00	7.6232493	TRUE
3	06.02.18	a	HCL	23	CC9	1/2c	Blood	II	N12-0486	2.80e+09	0.00e+00	7.4471580	TRUE
3	06.02.18	a	HCL	24	CC9	1/2c	Blood	II	N13-0001	1.57e+10	6.00e+02	7.4177484	FALSE
3	06.02.18	a	LA	11	CC6	4b	Food	I	N13-0703	3.20e+09	0.00e+00	7.5051500	TRUE
3	06.02.18	a	LA	12	CC6	4b	Food	I	N13-1184	4.30e+09	0.00e+00	7.6334685	TRUE
3	06.02.18	a	LA	13	CC6	4b	Blood	I	N11-2801	8.00e+08	0.00e+00	6.9030900	TRUE
3	06.02.18	a	LA	15	CC6	4b	Blood	I	N13-1271	3.30e+09	2.10e+04	5.1962946	FALSE
3	06.02.18	a	LA	16	CC6	4b	Blood	I	N13-1507	3.90e+09	1.10e+08	1.5496719	FALSE
3	06.02.18	a	LA	17	CC9	1/2c	Food	II	N11-1698	2.20e+09	0.00e+00	7.3424227	TRUE
3	06.02.18	a	LA	18	CC9	1/2c	Food	II	N12-0710	1.60e+09	3.30e+08	0.6856060	FALSE
3	06.02.18	a	LA	19	CC9	1/2c	Food	II	N12-0822	3.60e+09	1.10e+09	0.5149098	FALSE
3	06.02.18	a	LA	20	CC9	1/2c	Food	II	N11-1848	3.90e+09	2.50e+08	1.1931246	FALSE
3	06.02.18	a	LA	21	CC9	1/2c	Food	II	N14-0261	2.10e+09	2.00e+09	0.0211893	FALSE
3	06.02.18	a	LA	22	CC9	1/2a	Blood	II	N11-1837	4.00e+09	7.00e+02	6.7569620	FALSE
3	06.02.18	a	LA	23	CC9	1/2c	Blood	II	N12-0486	2.10e+09	0.00e+00	7.3222193	TRUE
3	06.02.18	a	LA	24	CC9	1/2c	Blood	II	N13-0001	2.40e+09	0.00e+00	7.3802112	TRUE
3	06.02.18	n	HCL	11	CC6	4b	Food	I	N13-0703	3.40e+09	0.00e+00	7.5314789	TRUE
3	06.02.18	n	HCL	12	CC6	4b	Food	I	N13-1184	4.10e+09	0.00e+00	7.6127839	TRUE
3	06.02.18	n	HCL	13	CC6	4b	Blood	I	N11-2801	3.00e+09	0.00e+00	7.4771213	TRUE
3	06.02.18	n	HCL	15	CC6	4b	Blood	I	N13-1271	3.20e+09	0.00e+00	7.5051500	TRUE
3	06.02.18	n	HCL	16	CC6	4b	Blood	I	N13-1507	3.60e+09	2.10e+08	1.2340832	FALSE
3	06.02.18	n	HCL	17	CC9	1/2c	Food	II	N11-1698	1.80e+09	0.00e+00	7.2552725	TRUE
3	06.02.18	n	HCL	18	CC9	1/2c	Food	II	N12-0710	2.90e+09	3.80e+06	2.8826144	FALSE
3	06.02.18	n	HCL	19	CC9	1/2c	Food	II	N12-0822	2.90e+09	2.80e+08	1.0152400	FALSE
3	06.02.18	n	HCL	20	CC9	1/2c	Food	II	N11-1848	2.20e+09	1.30e+08	1.2284793	FALSE
3	06.02.18	n	HCL	21	CC9	1/2c	Food	II	N14-0261	3.00e+09	2.20e+08	1.1346986	FALSE
3	06.02.18	n	HCL	22	CC9	1/2a	Blood	II	N11-1837	2.20e+09	0.00e+00	7.3424227	TRUE
3	06.02.18	n	HCL	23	CC9	1/2c	Blood	II	N12-0486	2.60e+09	0.00e+00	7.4149733	TRUE
3	06.02.18	n	HCL	24	CC9	1/2c	Blood	II	N13-0001	4.40e+09	0.00e+00	7.6434527	TRUE

(continued)

replicate	date	adapted.not	acid	strain	clonal.complex	serotype	source	lineage	NENT.Nr	t0	t60	logred	censored
3	06.02.18	n	LA	11	CC6	4b	Food	I	N13-0703	3.40e+09	0.00e+00	7.5314789	TRUE
3	06.02.18	n	LA	12	CC6	4b	Food	I	N13-1184	4.10e+09	2.80e+05	4.1656258	FALSE
3	06.02.18	n	LA	13	CC6	4b	Blood	I	N11-2801	3.00e+09	0.00e+00	7.4771213	TRUE
3	06.02.18	n	LA	15	CC6	4b	Blood	I	N13-1271	3.20e+09	0.00e+00	7.5051500	TRUE
3	06.02.18	n	LA	16	CC6	4b	Blood	I	N13-1507	3.60e+09	3.80e+07	1.9765189	FALSE
3	06.02.18	n	LA	17	CC9	1/2c	Food	II	N11-1698	1.80e+09	0.00e+00	7.2552725	TRUE
3	06.02.18	n	LA	18	CC9	1/2c	Food	II	N12-0710	2.90e+09	1.50e+07	2.2863067	FALSE
3	06.02.18	n	LA	19	CC9	1/2c	Food	II	N12-0822	2.90e+09	7.00e+08	0.6173000	FALSE
3	06.02.18	n	LA	20	CC9	1/2c	Food	II	N11-1848	2.20e+09	2.50e+07	1.9444827	FALSE
3	06.02.18	n	LA	21	CC9	1/2c	Food	II	N14-0261	3.00e+09	5.50e+08	0.7367586	FALSE
3	06.02.18	n	LA	22	CC9	1/2a	Blood	II	N11-1837	2.20e+09	0.00e+00	7.3424227	TRUE
3	06.02.18	n	LA	23	CC9	1/2c	Blood	II	N12-0486	2.60e+09	0.00e+00	7.4149733	TRUE
3	06.02.18	n	LA	24	CC9	1/2c	Blood	II	N13-0001	4.40e+09	0.00e+00	7.6434527	TRUE
3	09.02.18	a	HCL	25	CC121	1/2a	Food	II	N11-1218	1.80e+09	3.80e+08	0.6754889	FALSE
3	09.02.18	a	HCL	26	CC121	1/2a	Food	II	N12-0571	3.20e+09	4.80e+08	0.8239087	FALSE
3	09.02.18	a	HCL	27	CC121	1/2a	Food	II	N13-0369	3.00e+09	9.00e+02	6.5228787	FALSE
3	09.02.18	a	HCL	28	CC121	1/2b	Food	II	N13-0836	1.70e+09	4.60e+08	0.5676911	FALSE
3	09.02.18	a	HCL	29	CC121	1/2a	Food	II	N14-0205	2.60e+09	3.30e+08	0.8964594	FALSE
3	09.02.18	a	HCL	30	CC121	3c	Food	II	N14-0322	2.20e+09	3.20e+08	0.8372727	FALSE
3	09.02.18	a	HCL	31	CC121	1/2a	Blood	II	N12-0367	3.00e+09	1.80e+03	6.2218487	FALSE
3	09.02.18	a	HCL	32	CC121	1/2a	Blood	II	N13-0119	2.80e+09	2.20e+08	1.1047354	FALSE
3	09.02.18	a	HCL	33	ST739	1/2a	Food	II	N11-2542	2.10e+09	2.70e+08	0.8908555	FALSE
3	09.02.18	a	HCL	34	ST28	1/2a	Food	II	N13-0288	3.50e+09	7.00e+04	4.6989700	FALSE
3	09.02.18	a	HCL	35	ST226	1/2a	Food	II	N13-2179	2.20e+09	2.80e+08	0.8952646	FALSE
3	09.02.18	a	HCL	36	CC31	1/2a	Food	II	N13-0228	2.10e+09	1.00e+05	4.3222193	FALSE
3	09.02.18	a	HCL	37	CC207	1/2a	Blood	II	N12-1107	3.80e+09	3.00e+02	7.1026623	FALSE
3	09.02.18	n	HCL	25	CC121	1/2a	Food	II	N11-1218	2.80e+09	1.70e+09	0.2167091	FALSE
3	09.02.18	n	HCL	26	CC121	1/2a	Food	II	N12-0571	3.40e+09	1.70e+09	0.3010300	FALSE
3	09.02.18	n	HCL	27	CC121	1/2a	Food	II	N13-0369	3.90e+09	1.40e+03	6.4449366	FALSE
3	09.02.18	n	HCL	28	CC121	1/2b	Food	II	N13-0836	1.70e+09	1.30e+09	0.1165056	FALSE
3	09.02.18	n	HCL	29	CC121	1/2a	Food	II	N14-0205	3.60e+09	1.40e+09	0.4101745	FALSE
3	09.02.18	n	HCL	30	CC121	3c	Food	II	N14-0322	3.80e+09	1.50e+09	0.4036923	FALSE
3	09.02.18	n	HCL	31	CC121	1/2a	Blood	II	N12-0367	2.30e+09	1.90e+04	5.0829742	FALSE
3	09.02.18	n	HCL	32	CC121	1/2a	Blood	II	N13-0119	3.10e+09	8.00e+08	0.5882717	FALSE
3	09.02.18	n	HCL	33	ST739	1/2a	Food	II	N11-2542	3.20e+09	1.90e+09	0.2263964	FALSE
3	09.02.18	n	HCL	34	ST28	1/2a	Food	II	N13-0288	4.00e+09	3.00e+02	7.1249387	FALSE
3	09.02.18	n	HCL	35	ST226	1/2a	Food	II	N13-2179	2.60e+09	6.00e+08	0.6368221	FALSE
3	09.02.18	n	HCL	36	CC31	1/2a	Food	II	N13-0228	4.90e+09	3.00e+08	1.2130748	FALSE
3	09.02.18	n	HCL	37	CC207	1/2a	Blood	II	N12-1107	2.80e+09	7.00e+02	6.6020600	FALSE
3	09.02.18	n	LA	25	CC121	1/2a	Food	II	N11-1218	2.80e+09	1.20e+09	0.3679768	FALSE
3	09.02.18	n	LA	26	CC121	1/2a	Food	II	N12-0571	3.40e+09	3.40e+09	0.0000000	FALSE
3	09.02.18	n	LA	28	CC121	1/2b	Food	II	N13-0836	1.70e+09	1.30e+09	0.1165056	FALSE
3	09.02.18	n	LA	29	CC121	1/2a	Food	II	N14-0205	3.60e+09	1.30e+09	0.4423591	FALSE

(continued)

replicate	date	adapted.not	acid	strain	clonal.complex	serotype	source	lineage	NENT.Nr	t0	t60	logred	censored
3	09.02.18	n	LA	30	CC121	3c	Food	II	N14-0322	3.80e+09	1.80e+09	0.3245111	FALSE
3	09.02.18	n	LA	31	CC121	1/2a	Blood	II	N12-0367	2.30e+09	6.00e+04	4.5835766	FALSE
3	09.02.18	n	LA	32	CC121	1/2a	Blood	II	N13-0119	3.10e+09	1.70e+09	0.2609128	FALSE
3	09.02.18	n	LA	33	ST739	1/2a	Food	II	N11-2542	3.20e+09	1.10e+09	0.4637573	FALSE
3	09.02.18	n	LA	34	ST28	1/2a	Food	II	N13-0288	4.00e+09	2.40e+05	4.2218487	FALSE
3	09.02.18	n	LA	35	ST226	1/2a	Food	II	N13-2179	2.60e+09	1.10e+09	0.3735807	FALSE
3	09.02.18	n	LA	36	CC31	1/2a	Food	II	N13-0228	4.90e+09	2.30e+06	3.3284682	FALSE
3	09.02.18	n	LA	37	CC207	1/2a	Blood	II	N12-1107	2.80e+09	7.00e+03	5.6020600	FALSE
3	16.02.18	a	HCL	1	CC1	4b	Food	I	N12-0605	2.70e+09	1.10e+08	1.3899711	FALSE
3	16.02.18	a	HCL	10	CC4	4b	Food	I	N12-1772	5.60e+09	1.50e+03	6.5720968	FALSE
3	16.02.18	a	HCL	14	CC6	4b	Blood	I	N12-1387	1.30e+09	8.00e+04	4.2108534	FALSE
3	16.02.18	a	HCL	2	CC1	4b	Food	I	N12-1339	3.70e+09	2.70e+08	1.1368380	FALSE
3	16.02.18	a	HCL	3	CC1	4b	Food	I	N12-1996	2.10e+09	5.00e+07	1.6232493	FALSE
3	16.02.18	a	HCL	38	CC415	1/2b	Blood	II	N13-0762	3.00e+09	6.00e+04	4.6989700	FALSE
3	16.02.18	a	HCL	39	CC54	4b	Blood	I	N13-0177	4.00e+09	7.00e+07	1.7569620	FALSE
3	16.02.18	a	HCL	4	CC1	4b	Food	I	N13-0047	3.60e+09	1.00e+02	7.5563025	FALSE
3	16.02.18	a	HCL	40	CC224	1/2b	Blood	I	N12-1608	3.60e+09	3.00e+07	2.0791812	FALSE
3	16.02.18	a	HCL	5	CC1	4b	Blood	I	N11-2292	4.30e+09	2.40e+08	1.2532572	FALSE
3	16.02.18	a	HCL	6	CC1	4b	Blood	I	LL195	3.70e+09	1.70e+08	1.3377528	FALSE
3	16.02.18	a	HCL	7	CC1	4b	Blood	I	N13-0987	1.80e+09	1.70e+04	5.0248236	FALSE
3	16.02.18	a	HCL	8	CC1	4b	Blood	I	N13-1079	4.70e+09	3.70e+05	4.1038961	FALSE
3	16.02.18	a	HCL	9	CC6	4b	Food	I	N12-0460	2.90e+09	4.40e+07	1.8189453	FALSE
3	16.02.18	a	LA	1	CC1	4b	Food	I	N12-0605	4.00e+09	6.00e+08	0.8239087	FALSE
3	16.02.18	a	LA	10	CC4	4b	Food	I	N12-1772	2.70e+09	0.00e+00	7.4313638	TRUE
3	16.02.18	a	LA	14	CC6	4b	Blood	I	N12-1387	8.00e+08	0.00e+00	6.9030900	TRUE
3	16.02.18	a	LA	2	CC1	4b	Food	I	N12-1339	3.50e+09	9.00e+08	0.5898255	FALSE
3	16.02.18	a	LA	3	CC1	4b	Food	I	N12-1996	4.90e+09	9.00e+08	0.7359536	FALSE
3	16.02.18	a	LA	38	CC415	1/2b	Blood	II	N13-0762	3.00e+09	2.00e+02	7.1760913	FALSE
3	16.02.18	a	LA	39	CC54	4b	Blood	I	N13-0177	3.20e+09	1.70e+09	0.2747011	FALSE
3	16.02.18	a	LA	4	CC1	4b	Food	I	N13-0047	3.80e+09	1.00e+02	7.5797836	FALSE
3	16.02.18	a	LA	40	CC224	1/2b	Blood	I	N12-1608	2.50e+09	5.00e+08	0.6989700	FALSE
3	16.02.18	a	LA	5	CC1	4b	Blood	I	N11-2292	4.30e+09	7.00e+08	0.7883704	FALSE
3	16.02.18	a	LA	6	CC1	4b	Blood	I	LL195	4.30e+09	7.00e+08	0.7883704	FALSE
3	16.02.18	a	LA	7	CC1	4b	Blood	I	N13-0987	3.70e+09	0.00e+00	7.5682017	TRUE
3	16.02.18	a	LA	8	CC1	4b	Blood	I	N13-1079	4.70e+09	1.00e+02	7.6720979	FALSE
3	16.02.18	a	LA	9	CC6	4b	Food	I	N12-0460	9.00e+08	5.00e+08	0.2552725	FALSE
3	16.02.18	n	HCL	1	CC1	4b	Food	I	N12-0605	3.70e+09	3.70e+08	1.0000000	FALSE
3	16.02.18	n	HCL	10	CC4	4b	Food	I	N12-1772	4.40e+09	4.30e+05	4.0099842	FALSE
3	16.02.18	n	HCL	14	CC6	4b	Blood	I	N12-1387	1.30e+09	0.00e+00	7.1139434	TRUE
3	16.02.18	n	HCL	2	CC1	4b	Food	I	N12-1339	3.60e+09	9.00e+08	0.6020600	FALSE
3	16.02.18	n	HCL	3	CC1	4b	Food	I	N12-1996	3.50e+09	3.30e+08	1.0255541	FALSE
3	16.02.18	n	HCL	38	CC415	1/2b	Blood	II	N13-0762	3.90e+09	0.00e+00	7.5910646	TRUE
3	16.02.18	n	HCL	39	CC54	4b	Blood	I	N13-0177	2.90e+09	4.40e+08	0.8189453	FALSE
3	16.02.18	n	HCL	4	CC1	4b	Food	I	N13-0047	4.90e+09	4.00e+02	7.0881361	FALSE

(continued)

replicate	date	adapted.not	acid	strain	clonal.complex	serotype	source	lineage	NENT.Nr	t0	t60	logred	censored
3	16.02.18	n	HCL	40	CC224	1/2b	Blood	I	N12-1608	4.00e+09	4.70e+07	1.9299621	FALSE
3	16.02.18	n	HCL	5	CC1	4b	Blood	I	N11-2292	3.60e+09	5.00e+08	0.8573325	FALSE
3	16.02.18	n	HCL	6	CC1	4b	Blood	I	LL195	3.10e+09	7.00e+08	0.6462637	FALSE
3	16.02.18	n	HCL	7	CC1	4b	Blood	I	N13-0987	2.60e+09	0.00e+00	7.4149733	TRUE
3	16.02.18	n	HCL	8	CC1	4b	Blood	I	N13-1079	3.60e+09	2.00e+02	7.2552725	FALSE
3	16.02.18	n	HCL	9	CC6	4b	Food	I	N12-0460	2.10e+09	1.20e+08	1.2430380	FALSE
3	16.02.18	n	LA	1	CC1	4b	Food	I	N12-0605	3.70e+09	5.30e+08	0.8439259	FALSE
3	16.02.18	n	LA	10	CC4	4b	Food	I	N12-1772	4.40e+09	0.00e+00	7.6434527	TRUE
3	16.02.18	n	LA	14	CC6	4b	Blood	I	N12-1387	1.30e+09	0.00e+00	7.1139434	TRUE
3	16.02.18	n	LA	2	CC1	4b	Food	I	N12-1339	3.60e+09	2.20e+07	2.2138798	FALSE
3	16.02.18	n	LA	3	CC1	4b	Food	I	N12-1996	3.50e+09	1.50e+08	1.3679768	FALSE
3	16.02.18	n	LA	38	CC415	1/2b	Blood	II	N13-0762	3.90e+09	0.00e+00	7.5910646	TRUE
3	16.02.18	n	LA	39	CC54	4b	Blood	I	N13-0177	2.90e+09	3.20e+08	0.9572480	FALSE
3	16.02.18	n	LA	4	CC1	4b	Food	I	N13-0047	4.90e+09	0.00e+00	7.6901961	TRUE
3	16.02.18	n	LA	40	CC224	1/2b	Blood	I	N12-1608	4.00e+09	6.00e+06	2.8239087	FALSE
3	16.02.18	n	LA	5	CC1	4b	Blood	I	N11-2292	3.60e+09	2.20e+08	1.2138798	FALSE
3	16.02.18	n	LA	6	CC1	4b	Blood	I	LL195	3.10e+09	2.60e+08	1.0763883	FALSE
3	16.02.18	n	LA	7	CC1	4b	Blood	I	N13-0987	2.60e+09	0.00e+00	7.4149733	TRUE
3	16.02.18	n	LA	8	CC1	4b	Blood	I	N13-1079	3.60e+09	0.00e+00	7.5563025	TRUE
3	16.02.18	n	LA	9	CC6	4b	Food	I	N12-0460	2.10e+09	1.70e+07	2.0917704	FALSE
3	20.02.18	a	LA	25	CC121	1/2a	Food	II	N11-1218	2.00e+09	1.80e+09	0.0457575	FALSE
3	20.02.18	a	LA	26	CC121	1/2a	Food	II	N12-0571	2.70e+09	1.20e+09	0.3521825	FALSE
3	20.02.18	a	LA	27	CC121	1/2a	Food	II	N13-0369	3.70e+09	3.20e+03	6.0630517	FALSE
3	20.02.18	a	LA	28	CC121	1/2b	Food	II	N13-0836	1.30e+09	1.50e+09	-0.0621479	FALSE
3	20.02.18	a	LA	29	CC121	1/2a	Food	II	N14-0205	1.30e+09	9.00e+08	0.1597008	FALSE
3	20.02.18	a	LA	30	CC121	3c	Food	II	N14-0322	1.80e+09	1.20e+09	0.1760913	FALSE
3	20.02.18	a	LA	31	CC121	1/2a	Blood	II	N12-0367	2.80e+09	4.90e+03	5.7569620	FALSE
3	20.02.18	a	LA	32	CC121	1/2a	Blood	II	N13-0119	2.60e+09	1.40e+09	0.2688453	FALSE
3	20.02.18	a	LA	33	ST739	1/2a	Food	II	N11-2542	1.80e+09	1.80e+09	0.0000000	FALSE
3	20.02.18	a	LA	34	ST28	1/2a	Food	II	N13-0288	2.80e+09	3.30e+04	4.9286441	FALSE
3	20.02.18	a	LA	35	ST226	1/2a	Food	II	N13-2179	3.00e+09	8.00e+08	0.5740313	FALSE
3	20.02.18	a	LA	36	CC31	1/2a	Food	II	N13-0228	2.30e+09	1.80e+07	2.1064553	FALSE
3	20.02.18	a	LA	37	CC207	1/2a	Blood	II	N12-1107	3.40e+09	1.70e+03	6.3010300	FALSE
4	20.02.18	a	HCL	18	CC9	1/2c	Food	II	N12-0710	2.90e+09	6.00e+07	1.6842467	FALSE
4	20.02.18	a	HCL	22	CC9	1/2a	Blood	II	N11-1837	1.90e+09	0.00e+00	7.2787536	TRUE
4	20.02.18	a	LA	28	CC121	1/2b	Food	II	N13-0836	2.00e+09	7.00e+08	0.4559320	FALSE
4	20.02.18	n	HCL	18	CC9	1/2c	Food	II	N12-0710	2.20e+09	3.20e+07	1.8372727	FALSE
4	20.02.18	n	HCL	22	CC9	1/2a	Blood	II	N11-1837	2.60e+09	1.20e+03	6.3357921	FALSE
4	20.02.18	n	HCL	28	CC121	1/2b	Food	II	N13-0836	2.00e+09	9.00e+08	0.3467875	FALSE
4	20.02.18	n	HCL	32	CC121	1/2a	Blood	II	N13-0119	3.00e+09	5.00e+08	0.7781513	FALSE
4	20.02.18	n	HCL	36	CC31	1/2a	Food	II	N13-0228	1.80e+09	8.00e+02	6.3521825	FALSE
4	20.02.18	n	LA	18	CC9	1/2c	Food	II	N12-0710	3.20e+09	2.70e+07	2.0737862	FALSE
4	20.02.18	n	LA	22	CC9	1/2a	Blood	II	N11-1837	2.10e+09	0.00e+00	7.3222193	TRUE

(continued)

replicate	date	adapted.not	acid	strain	clonal.complex	serotype	source	lineage	NENT.Nr	t0	t60	logred	censored
4	20.02.18	n	LA	25	CC121	1/2a	Food	II	N11-1218	2.20e+09	1.30e+09	0.2284793	FALSE
4	20.02.18	n	LA	26	CC121	1/2a	Food	II	N12-0571	3.10e+09	1.60e+09	0.2872417	FALSE
4	20.02.18	n	LA	27	CC121	1/2a	Food	II	N13-0369	2.20e+09	2.90e+03	5.8800247	FALSE
4	20.02.18	n	LA	28	CC121	1/2b	Food	II	N13-0836	2.00e+09	8.00e+08	0.3979400	FALSE
4	20.02.18	n	LA	29	CC121	1/2a	Food	II	N14-0205	3.00e+09	1.70e+09	0.2466723	FALSE
4	20.02.18	n	LA	3	CC1	4b	Food	I	N12-1996	2.30e+09	1.30e+09	0.2477845	FALSE
4	20.02.18	n	LA	30	CC121	1/2a	Food	II	N14-0205	2.20e+09	1.70e+09	0.1119738	FALSE
4	20.02.18	n	LA	32	CC121	3c	Food	II	N14-0322	3.00e+09	1.10e+09	0.4357286	FALSE
4	20.02.18	n	LA	33	ST739	1/2a	Food	II	N11-2542	3.30e+09	1.40e+09	0.3723859	FALSE
4	20.02.18	n	LA	35	ST226	1/2a	Food	II	N13-2179	3.90e+09	1.10e+09	0.5496719	FALSE
5	20.02.18	a	HCL	28	CC121	1/2b	Food	II	N13-0836	3.70e+09	3.00e+08	1.0910805	FALSE
5	20.02.18	a	HCL	36	CC31	1/2a	Food	II	N13-0228	2.60e+09	2.30e+05	4.0532455	FALSE

The second chunk here calculates the log-reduction, substituting in the detection limit of 100 CFU/mL if the t60 value was zero. This isn't great, but it doesn't stretch the data as much as a very small substitution. I also added a column noting whether the observation is "censored" or not, since that's not clear in the logred column.

The third chunk grabs the CC/serotype/lineage/source info from the `hemolysis.csv` data sheet, since this raw data doesn't have those items.

```
acid$NENT.Nr <- reorder(acid$NENT.Nr, acid$logred)
ggplot(acid, aes(x=NENT.Nr,y=logred)) +
  theme_bw()+
  geom_boxplot() +
  theme(axis.text.x=element_text(angle=90,hjust=1))+
  facet_grid(adapted.not~acid)
```

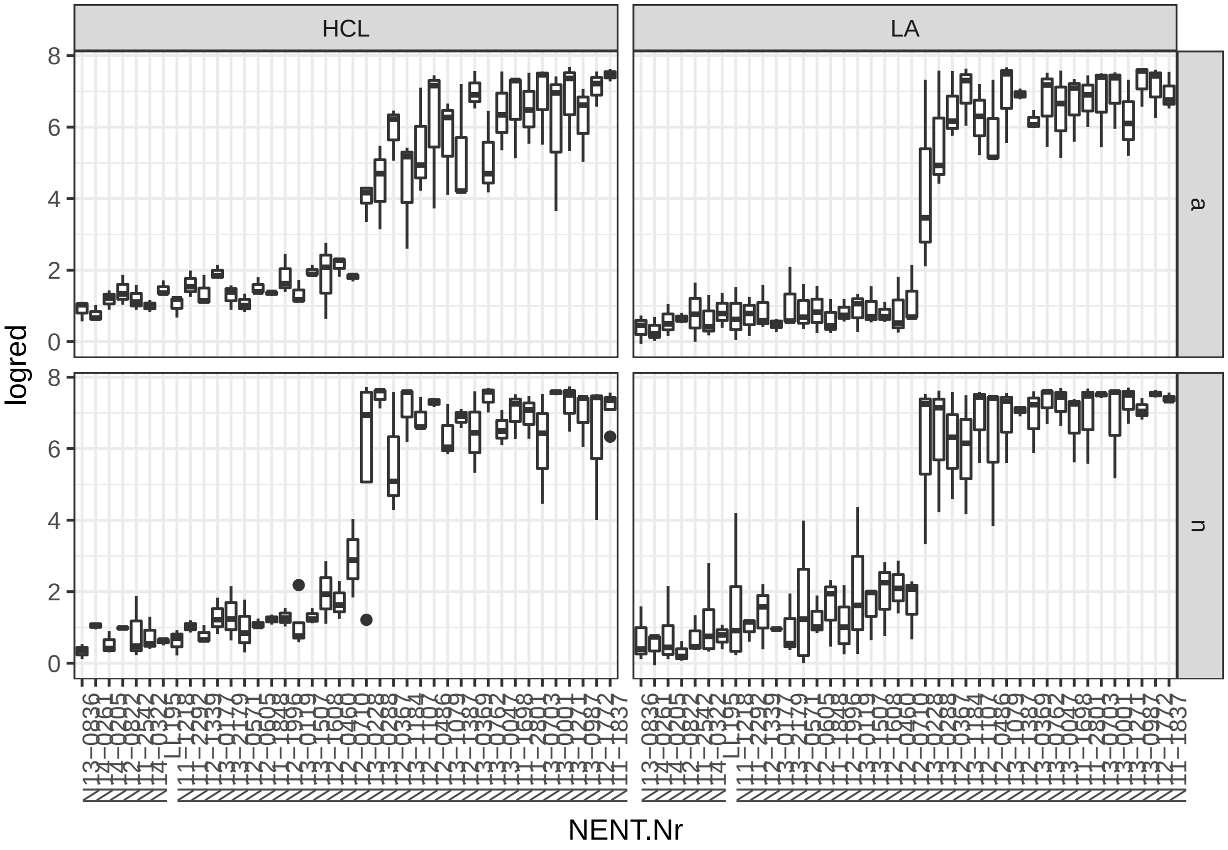

It looks like there's a yes/no kind of response.

Can we do a linear model?

```
m <- lm(logred~NENT.Nr*adapted.not*acid, data=acid)
plot(predict(m),resid(m))
```

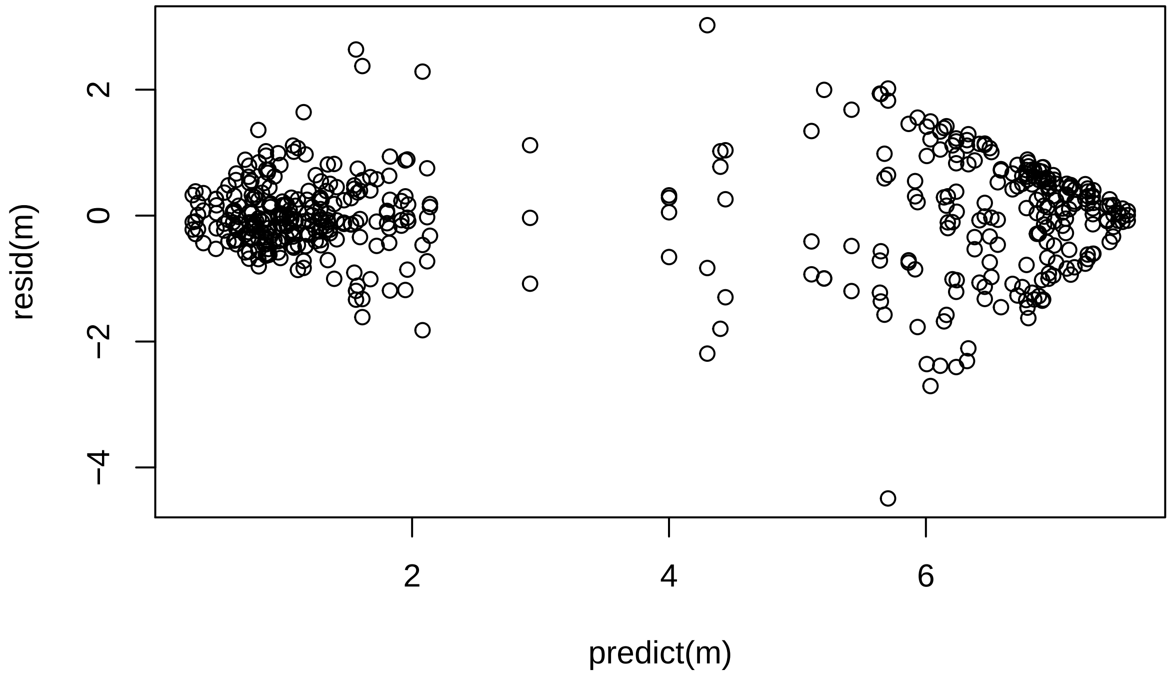


```r
qqnorm(resid(m))
qqline(resid(m))
```

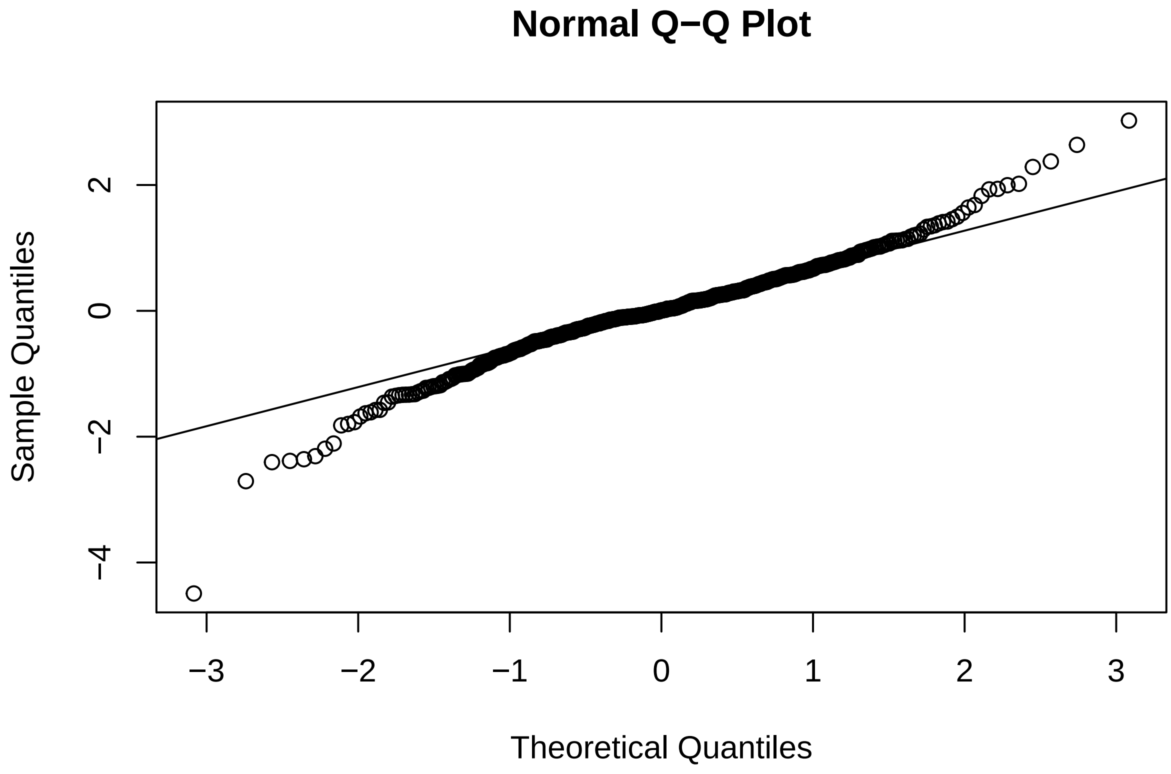

Unfortunately, this data does not appear to satisfy the assumptions of a linear model - the variance doesn't look constant, and the residuals look distinctly non-normal.

Classifying into two groups?

```
hist(acid$logred)
```

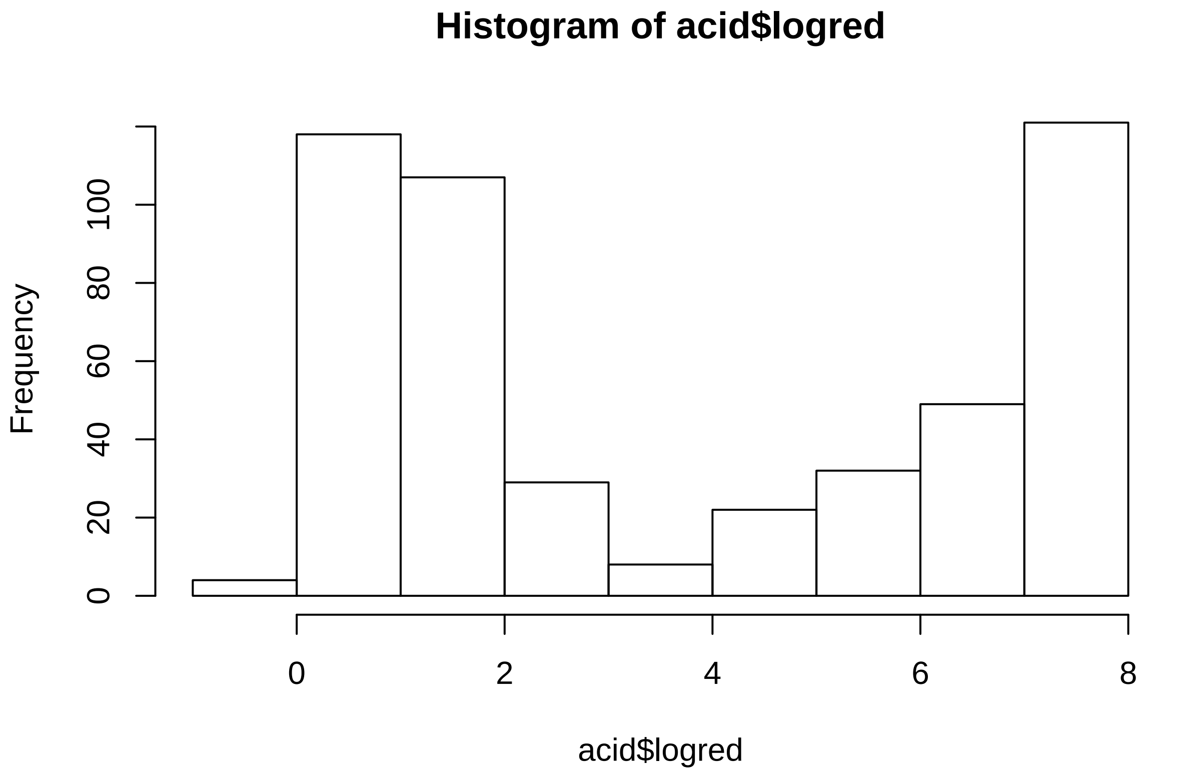

Histogram of the log-reduction.

```
acid %>%
  ggplot(aes(x=logred)) +
  geom_histogram() +
  facet_grid(acid~adapted.not)
```

```
## `stat_bin()` using `bins = 30`. Pick better value with `binwidth`.
```

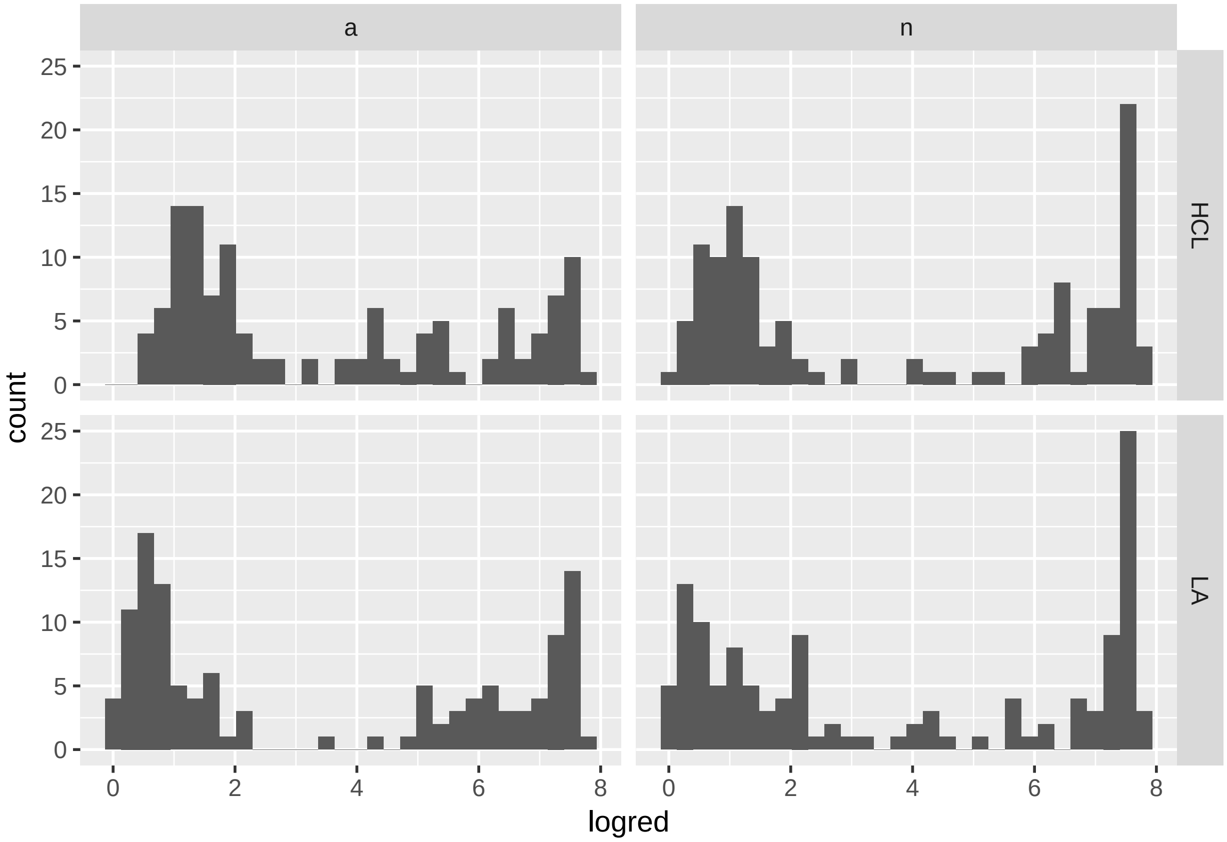

Here's another glance at the bimodal nature of the data - just as a histogram by acid/adaptation.

K-means?

If we believe that an observation is either susceptible (high log-reduction) or not (low log-reduction), then it would be reasonable to use something like k-means clustering to group the observations into two clusters. Then we could see if there's any trend along the lines of interest.

```
acid$cluster <- as.factor(kmeans(acid$logred,centers = c(1,7))$cluster)
acid %>%
  ggplot(aes(x=logred,fill=cluster)) +
  geom_histogram() +
  facet_grid(acid~adapted.not)
```

```
## `stat_bin()` using `bins = 30`. Pick better value with `binwidth`.
```

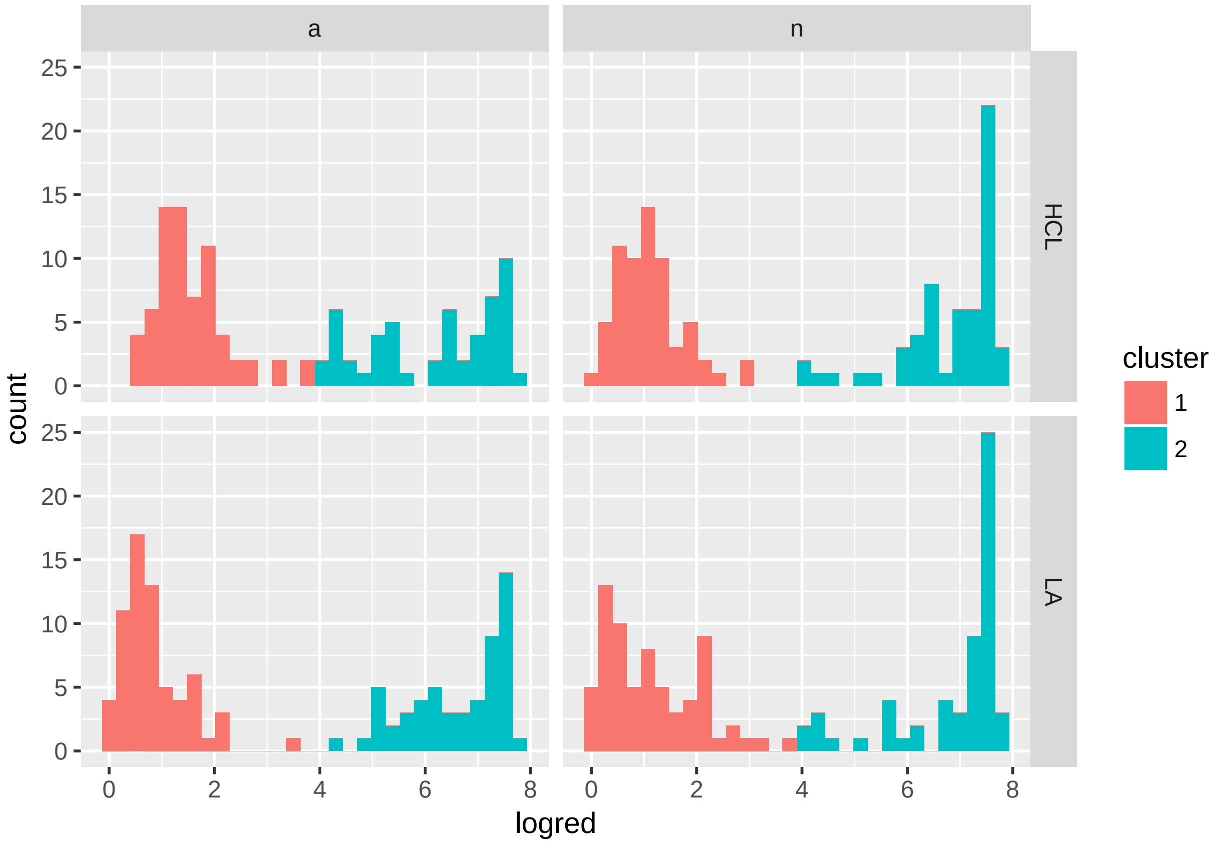


```r
acid %>%
  kable("latex", longtable = T, booktabs = T) %>%
  kable_styling(latex_options = c("repeat_header"), font_size = 7) %>%
  landscape()
```

replicate	date	adapted.not	acid	strain	clonal.complex	serotype	source	lineage	NENT.Nr	t0	t60	logred	censored	cluster
1	07.02.18	a	HCL	25	CC121	1/2a	Food	II	N11-1218	2.50e+09	1.60e+08	1.1938200	FALSE	1
1	07.02.18	a	HCL	26	CC121	1/2a	Food	II	N12-0571	3.50e+09	3.40e+08	1.0125891	FALSE	1
1	07.02.18	a	HCL	27	CC121	1/2a	Food	II	N13-0369	3.70e+09	0.00e+00	7.5682017	TRUE	2
1	07.02.18	a	HCL	28	CC121	1/2b	Food	II	N13-0836	2.60e+09	2.40e+08	1.0347621	FALSE	1
1	07.02.18	a	HCL	29	CC121	1/2a	Food	II	N14-0205	2.80e+09	1.70e+08	1.2167091	FALSE	1
1	07.02.18	a	HCL	30	CC121	3c	Food	II	N14-0322	2.10e+09	2.10e+08	1.0000000	FALSE	1
1	07.02.18	a	HCL	31	CC121	1/2a	Blood	II	N12-0367	1.50e+09	1.30e+04	5.0621479	FALSE	2
1	07.02.18	a	HCL	32	CC121	1/2a	Blood	II	N13-0119	3.10e+09	2.10e+08	1.1691424	FALSE	1
1	07.02.18	a	HCL	33	ST739	1/2a	Food	II	N11-2542	2.30e+09	1.80e+08	1.1064553	FALSE	1
1	07.02.18	a	HCL	34	ST28	1/2a	Food	II	N13-0288	2.70e+09	9.00e+03	5.4771213	FALSE	2
1	07.02.18	a	HCL	35	ST226	1/2a	Food	II	N13-2179	6.00e+09	1.60e+08	1.5740313	FALSE	1
1	07.02.18	a	HCL	36	CC31	1/2a	Food	II	N13-0228	5.00e+09	2.60e+05	4.2839967	FALSE	2
1	07.02.18	a	HCL	37	CC207	1/2a	Blood	II	N12-1107	2.60e+09	3.00e+04	4.9378521	FALSE	2
1	07.02.18	a	LA	25	CC121	1/2a	Food	II	N11-1218	2.10e+09	5.00e+08	0.6232493	FALSE	1
1	07.02.18	a	LA	26	CC121	1/2a	Food	II	N12-0571	2.90e+09	6.00e+08	0.6842467	FALSE	1
1	07.02.18	a	LA	27	CC121	1/2a	Food	II	N13-0369	2.10e+09	7.00e+02	6.4771213	FALSE	2
1	07.02.18	a	LA	28	CC121	1/2b	Food	II	N13-0836	2.70e+09	5.00e+08	0.7323938	FALSE	1
1	07.02.18	a	LA	29	CC121	1/2a	Food	II	N14-0205	3.80e+09	1.20e+09	0.5006024	FALSE	1
1	07.02.18	a	LA	30	CC121	3c	Food	II	N14-0322	2.10e+09	8.00e+08	0.4191293	FALSE	1
1	07.02.18	a	LA	31	CC121	1/2a	Blood	II	N12-0367	2.20e+09	1.50e+03	6.1663314	FALSE	2
1	07.02.18	a	LA	32	CC121	1/2a	Blood	II	N13-0119	1.08e+10	5.00e+08	1.3344538	FALSE	1
1	07.02.18	a	LA	33	ST739	1/2a	Food	II	N11-2542	3.50e+09	6.00e+08	0.7659168	FALSE	1
1	07.02.18	a	LA	34	ST28	1/2a	Food	II	N13-0288	3.40e+09	1.30e+05	4.4175356	FALSE	2
1	07.02.18	a	LA	35	ST226	1/2a	Food	II	N13-2179	2.60e+09	7.00e+08	0.5698753	FALSE	1
1	07.02.18	a	LA	36	CC31	1/2a	Food	II	N13-0228	3.80e+09	1.30e+06	3.4658402	FALSE	1
1	07.02.18	a	LA	37	CC207	1/2a	Blood	II	N12-1107	3.60e+09	2.20e+04	5.2138798	FALSE	2
1	07.02.18	n	HCL	25	CC121	1/2a	Food	II	N11-1218	4.00e+09	8.00e+08	0.6989700	FALSE	1
1	07.02.18	n	HCL	26	CC121	1/2a	Food	II	N12-0571	4.20e+09	6.00e+08	0.8450980	FALSE	1
1	07.02.18	n	HCL	27	CC121	1/2a	Food	II	N13-0369	3.40e+09	1.60e+04	5.3273589	FALSE	2
1	07.02.18	n	HCL	28	CC121	1/2b	Food	II	N13-0836	3.10e+09	9.00e+08	0.5371192	FALSE	1
1	07.02.18	n	HCL	29	CC121	1/2a	Food	II	N14-0205	2.40e+09	1.20e+09	0.3010300	FALSE	1
1	07.02.18	n	HCL	30	CC121	3c	Food	II	N14-0322	2.10e+09	6.00e+08	0.5440680	FALSE	1
1	07.02.18	n	HCL	31	CC121	1/2a	Blood	II	N12-0367	2.70e+09	1.40e+05	4.2852357	FALSE	2
1	07.02.18	n	HCL	32	CC121	1/2a	Blood	II	N13-0119	3.30e+09	6.00e+08	0.7403627	FALSE	1
1	07.02.18	n	HCL	33	ST739	1/2a	Food	II	N11-2542	3.30e+09	1.10e+09	0.4771213	FALSE	1
1	07.02.18	n	HCL	34	ST28	1/2a	Food	II	N13-0288	4.20e+09	0.00e+00	7.6232493	TRUE	2
1	07.02.18	n	HCL	35	ST226	1/2a	Food	II	N13-2179	7.10e+09	4.10e+08	1.2384745	FALSE	1
1	07.02.18	n	HCL	36	CC31	1/2a	Food	II	N13-0228	5.30e+09	0.00e+00	7.7242759	TRUE	2
1	07.02.18	n	HCL	37	CC207	1/2a	Blood	II	N12-1107	3.90e+09	1.00e+03	6.5910646	FALSE	2
1	07.02.18	n	LA	25	CC121	1/2a	Food	II	N11-1218	4.00e+09	1.40e+08	1.4559320	FALSE	1
1	07.02.18	n	LA	26	CC121	1/2a	Food	II	N12-0571	4.20e+09	2.80e+07	2.1760913	FALSE	1
1	07.02.18	n	LA	27	CC121	1/2a	Food	II	N13-0369	3.40e+09	2.00e+02	7.2304489	FALSE	2
1	07.02.18	n	LA	28	CC121	1/2b	Food	II	N13-0836	3.10e+09	8.00e+07	1.5882717	FALSE	1
1	07.02.18	n	LA	29	CC121	1/2a	Food	II	N14-0205	2.40e+09	2.13e+08	1.0518316	FALSE	1
1	07.02.18	n	LA	30	CC121	3c	Food	II	N14-0322	2.10e+09	1.81e+08	1.0645407	FALSE	1

(continued)

replicate	date	adapted.not	acid	strain	clonal.complex	serotype	source	lineage	NENT.Nr	t0	t60	logred	censored	cluster
1	07.02.18	n	LA	31	CC121	1/2a	Blood	II	N12-0367	2.70e+09	1.30e+03	6.3174204	FALSE	2
1	07.02.18	n	LA	32	CC121	1/2a	Blood	II	N13-0119	3.30e+09	8.00e+07	1.6154240	FALSE	1
1	07.02.18	n	LA	33	ST739	1/2a	Food	II	N11-2542	3.30e+09	1.50e+08	1.3424227	FALSE	1
1	07.02.18	n	LA	34	ST28	1/2a	Food	II	N13-0288	4.20e+09	3.00e+02	7.1461280	FALSE	2
1	07.02.18	n	LA	35	ST226	1/2a	Food	II	N13-2179	7.10e+09	8.00e+07	1.9481684	FALSE	1
1	07.02.18	n	LA	36	CC31	1/2a	Food	II	N13-0228	5.30e+09	3.00e+02	7.2471546	FALSE	2
1	07.02.18	n	LA	37	CC207	1/2a	Blood	II	N12-1107	3.90e+09	1.00e+02	7.5910646	FALSE	2
1	13.02.18	a	HCL	11	CC6	4b	Food	I	N13-0703	2.90e+09	0.00e+00	7.4623980	TRUE	2
1	13.02.18	a	HCL	12	CC6	4b	Food	I	N13-1184	1.80e+09	1.20e+04	5.1760913	FALSE	2
1	13.02.18	a	HCL	13	CC6	4b	Blood	I	N11-2801	4.50e+09	1.50e+03	6.4771213	FALSE	2
1	13.02.18	a	HCL	15	CC6	4b	Blood	I	N13-1271	2.30e+09	0.00e+00	7.3617278	TRUE	2
1	13.02.18	a	HCL	16	CC6	4b	Blood	I	N13-1507	4.70e+09	6.20e+07	1.8797062	FALSE	1
1	13.02.18	a	HCL	17	CC9	1/2c	Food	II	N11-1698	2.10e+09	0.00e+00	7.3222193	TRUE	2
1	13.02.18	a	HCL	19	CC9	1/2c	Food	II	N12-0822	3.70e+09	1.70e+08	1.3377528	FALSE	1
1	13.02.18	a	HCL	20	CC9	1/2c	Food	II	N11-1848	3.10e+09	1.30e+08	1.3774183	FALSE	1
1	13.02.18	a	HCL	21	CC9	1/2c	Food	II	N14-0261	3.20e+09	8.00e+08	0.6020600	FALSE	1
1	13.02.18	a	HCL	22	CC9	1/2a	Blood	II	N11-1837	3.00e+09	1.00e+02	7.4771213	FALSE	2
1	13.02.18	a	HCL	23	CC9	1/2c	Blood	II	N12-0486	2.90e+09	2.00e+02	7.1613680	FALSE	2
1	13.02.18	a	HCL	24	CC9	1/2c	Blood	II	N13-0001	4.50e+09	5.00e+02	6.9542425	FALSE	2
1	13.02.18	a	LA	11	CC6	4b	Food	I	N13-0703	2.50e+09	0.00e+00	7.3979400	TRUE	2
1	13.02.18	a	LA	12	CC6	4b	Food	I	N13-1184	2.00e+09	1.00e+02	7.3010300	FALSE	2
1	13.02.18	a	LA	13	CC6	4b	Blood	I	N11-2801	2.80e+09	0.00e+00	7.4471580	TRUE	2
1	13.02.18	a	LA	15	CC6	4b	Blood	I	N13-1271	2.10e+09	0.00e+00	7.3222193	TRUE	2
1	13.02.18	a	LA	16	CC6	4b	Blood	I	N13-1507	3.80e+09	1.10e+09	0.5383909	FALSE	1
1	13.02.18	a	LA	17	CC9	1/2c	Food	II	N11-1698	3.70e+09	3.00e+02	7.0910805	FALSE	2
1	13.02.18	a	LA	18	CC9	1/2c	Food	II	N12-0710	2.50e+09	1.80e+07	2.1426675	FALSE	1
1	13.02.18	a	LA	19	CC9	1/2c	Food	II	N12-0822	3.40e+09	8.00e+08	0.6283889	FALSE	1
1	13.02.18	a	LA	20	CC9	1/2c	Food	II	N11-1848	2.80e+09	1.00e+09	0.4471580	FALSE	1
1	13.02.18	a	LA	21	CC9	1/2c	Food	II	N14-0261	2.50e+09	1.50e+09	0.2218487	FALSE	1
1	13.02.18	a	LA	22	CC9	1/2a	Blood	II	N11-1837	3.00e+09	9.00e+02	6.5228787	FALSE	2
1	13.02.18	a	LA	23	CC9	1/2c	Blood	II	N12-0486	2.50e+09	1.90e+04	5.1191864	FALSE	2
1	13.02.18	a	LA	24	CC9	1/2c	Blood	II	N13-0001	3.40e+09	1.00e+02	7.5314789	FALSE	2
1	13.02.18	n	HCL	11	CC6	4b	Food	I	N13-0703	3.50e+09	1.30e+03	6.4301247	FALSE	2
1	13.02.18	n	HCL	12	CC6	4b	Food	I	N13-1184	3.10e+09	2.00e+03	6.1903317	FALSE	2
1	13.02.18	n	HCL	13	CC6	4b	Blood	I	N11-2801	4.80e+09	4.00e+02	7.0791812	FALSE	2
1	13.02.18	n	HCL	15	CC6	4b	Blood	I	N13-1271	5.10e+09	1.70e+03	6.4771213	FALSE	2
1	13.02.18	n	HCL	16	CC6	4b	Blood	I	N13-1507	4.50e+09	1.30e+08	1.5392692	FALSE	1
1	13.02.18	n	HCL	17	CC9	1/2c	Food	II	N11-1698	2.40e+09	1.30e+03	6.2662679	FALSE	2
1	13.02.18	n	HCL	19	CC9	1/2c	Food	II	N12-0822	3.70e+09	3.80e+08	0.9884181	FALSE	1
1	13.02.18	n	HCL	20	CC9	1/2c	Food	II	N11-1848	4.20e+09	3.50e+08	1.0791812	FALSE	1
1	13.02.18	n	HCL	21	CC9	1/2c	Food	II	N14-0261	2.90e+09	3.30e+08	0.9438841	FALSE	1
1	13.02.18	n	HCL	22	CC9	1/2a	Blood	II	N11-1837	3.70e+09	1.00e+02	7.5682017	FALSE	2
1	13.02.18	n	HCL	23	CC9	1/2c	Blood	II	N12-0486	2.90e+09	2.00e+02	7.1613680	FALSE	2

(continued)

replicate	date	adapted.not	acid	strain	clonal.complex	serotype	source	lineage	NENT.Nr	t0	t60	logred	censored	cluster
1	13.02.18	n	HCL	24	CC9	1/2c	Blood	II	N13-0001	3.80e+09	0.00e+00	7.5797836	TRUE	2
1	13.02.18	n	LA	11	CC6	4b	Food	I	N13-0703	3.50e+09	0.00e+00	7.5440680	TRUE	2
1	13.02.18	n	LA	12	CC6	4b	Food	I	N13-1184	3.10e+09	0.00e+00	7.4913617	TRUE	2
1	13.02.18	n	LA	13	CC6	4b	Blood	I	N11-2801	4.80e+09	0.00e+00	7.6812412	TRUE	2
1	13.02.18	n	LA	15	CC6	4b	Blood	I	N13-1271	5.10e+09	0.00e+00	7.7075702	TRUE	2
1	13.02.18	n	LA	16	CC6	4b	Blood	I	N13-1507	4.50e+09	4.20e+07	2.0299632	FALSE	1
1	13.02.18	n	LA	17	CC9	1/2c	Food	II	N11-1698	2.40e+09	0.00e+00	7.3802112	TRUE	2
1	13.02.18	n	LA	19	CC9	1/2c	Food	II	N12-0822	3.70e+09	2.40e+09	0.1879905	FALSE	1
1	13.02.18	n	LA	20	CC9	1/2c	Food	II	N11-1848	4.20e+09	2.00e+07	2.3222193	FALSE	1
1	13.02.18	n	LA	21	CC9	1/2c	Food	II	N14-0261	2.90e+09	5.30e+08	0.7381221	FALSE	1
1	13.02.18	n	LA	22	CC9	1/2a	Blood	II	N11-1837	3.70e+09	0.00e+00	7.5682017	TRUE	2
1	13.02.18	n	LA	23	CC9	1/2c	Blood	II	N12-0486	2.90e+09	1.00e+02	7.4623980	FALSE	2
1	13.02.18	n	LA	24	CC9	1/2c	Blood	II	N13-0001	3.80e+09	0.00e+00	7.5797836	TRUE	2
1	14.02.18	a	HCL	1	CC1	4b	Food	I	N12-0605	3.80e+09	6.00e+07	1.8016323	FALSE	1
1	14.02.18	a	HCL	10	CC4	4b	Food	I	N12-1772	3.30e+09	2.00e+02	7.2174839	FALSE	2
1	14.02.18	a	HCL	14	CC6	4b	Blood	I	N12-1387	5.00e+08	3.10e+04	4.2076083	FALSE	2
1	14.02.18	a	HCL	2	CC1	4b	Food	I	N12-1339	4.40e+09	6.00e+07	1.8653014	FALSE	1
1	14.02.18	a	HCL	3	CC1	4b	Food	I	N12-1996	3.70e+09	1.30e+07	2.4542584	FALSE	1
1	14.02.18	a	HCL	38	CC415	1/2b	Blood	II	N13-0762	3.90e+09	2.60e+05	4.1760913	FALSE	2
1	14.02.18	a	HCL	39	CC54	4b	Blood	I	N13-0177	3.80e+09	2.70e+07	2.1484198	FALSE	1
1	14.02.18	a	HCL	4	CC1	4b	Food	I	N13-0047	4.40e+09	2.00e+03	6.3424227	FALSE	2
1	14.02.18	a	HCL	40	CC224	1/2b	Blood	I	N12-1608	3.50e+09	6.00e+06	2.7659168	FALSE	1
1	14.02.18	a	HCL	5	CC1	4b	Blood	I	N11-2292	4.40e+09	4.50e+07	1.9902402	FALSE	1
1	14.02.18	a	HCL	6	CC1	4b	Blood	I	LL195	2.90e+09	5.60e+07	1.7142100	FALSE	1
1	14.02.18	a	HCL	7	CC1	4b	Blood	I	N13-0987	3.30e+09	8.00e+02	6.6154240	FALSE	2
1	14.02.18	a	HCL	8	CC1	4b	Blood	I	N13-1079	3.90e+09	2.10e+03	6.2688453	FALSE	2
1	14.02.18	a	HCL	9	CC6	4b	Food	I	N12-0460	4.00e+09	1.90e+07	2.3233064	FALSE	1
1	14.02.18	a	LA	1	CC1	4b	Food	I	N12-0605	3.60e+09	1.00e+08	1.5563025	FALSE	1
1	14.02.18	a	LA	10	CC4	4b	Food	I	N12-1772	4.00e+09	0.00e+00	7.6020600	TRUE	2
1	14.02.18	a	LA	14	CC6	4b	Blood	I	N12-1387	6.00e+08	0.00e+00	6.7781513	TRUE	2
1	14.02.18	a	LA	2	CC1	4b	Food	I	N12-1339	1.10e+10	2.80e+08	1.5942347	FALSE	1
1	14.02.18	a	LA	3	CC1	4b	Food	I	N12-1996	3.60e+09	2.30e+08	1.1945747	FALSE	1
1	14.02.18	a	LA	38	CC415	1/2b	Blood	II	N13-0762	3.30e+09	0.00e+00	7.5185139	TRUE	2
1	14.02.18	a	LA	39	CC54	4b	Blood	I	N13-0177	3.50e+09	8.00e+08	0.6409781	FALSE	1
1	14.02.18	a	LA	4	CC1	4b	Food	I	N13-0047	3.20e+09	7.00e+02	6.6600519	FALSE	2
1	14.02.18	a	LA	40	CC224	1/2b	Blood	I	N12-1608	3.80e+09	2.90e+08	1.1173856	FALSE	1
1	14.02.18	a	LA	5	CC1	4b	Blood	I	N11-2292	4.10e+09	2.30e+08	1.2510560	FALSE	1
1	14.02.18	a	LA	6	CC1	4b	Blood	I	LL195	3.70e+09	1.60e+08	1.3640817	FALSE	1
1	14.02.18	a	LA	7	CC1	4b	Blood	I	N13-0987	4.40e+09	0.00e+00	7.6434527	TRUE	2
1	14.02.18	a	LA	8	CC1	4b	Blood	I	N13-1079	3.10e+09	0.00e+00	7.4913617	TRUE	2
1	14.02.18	a	LA	9	CC6	4b	Food	I	N12-0460	3.20e+09	4.90e+07	1.8149539	FALSE	1
1	14.02.18	n	HCL	1	CC1	4b	Food	I	N12-0605	3.90e+09	2.20e+08	1.2486419	FALSE	1
1	14.02.18	n	HCL	10	CC4	4b	Food	I	N12-1772	3.30e+09	0.00e+00	7.5185139	TRUE	2

(continued)

replicate	date	adapted.not	acid	strain	clonal.complex	serotype	source	lineage	NENT.Nr	t0	t60	logred	censored	cluster
1	14.02.18	n	HCL	14	CC6	4b	Blood	I	N12-1387	8.00e+08	0.00e+00	6.9030900	TRUE	2
1	14.02.18	n	HCL	2	CC1	4b	Food	I	N12-1339	3.40e+09	2.90e+08	1.0690809	FALSE	1
1	14.02.18	n	HCL	3	CC1	4b	Food	I	N12-1996	3.50e+09	1.00e+08	1.5440680	FALSE	1
1	14.02.18	n	HCL	38	CC415	1/2b	Blood	II	N13-0762	4.10e+09	4.00e+02	7.0107239	FALSE	2
1	14.02.18	n	HCL	39	CC54	4b	Blood	I	N13-0177	3.80e+09	2.30e+08	1.2180558	FALSE	1
1	14.02.18	n	HCL	4	CC1	4b	Food	I	N13-0047	3.50e+09	2.80e+03	6.0969100	FALSE	2
1	14.02.18	n	HCL	40	CC224	1/2b	Blood	I	N12-1608	4.30e+09	6.00e+06	2.8553172	FALSE	1
1	14.02.18	n	HCL	5	CC1	4b	Blood	I	N11-2292	3.30e+09	3.20e+08	1.0133640	FALSE	1
1	14.02.18	n	HCL	6	CC1	4b	Blood	I	LL195	1.80e+09	3.80e+08	0.6754889	FALSE	1
1	14.02.18	n	HCL	7	CC1	4b	Blood	I	N13-0987	1.10e+09	1.00e+03	6.0413927	FALSE	2
1	14.02.18	n	HCL	8	CC1	4b	Blood	I	N13-1079	2.10e+09	3.00e+03	5.8450980	FALSE	2
1	14.02.18	n	HCL	9	CC6	4b	Food	I	N12-0460	3.40e+09	8.00e+07	1.6283889	FALSE	1
1	14.02.18	n	LA	1	CC1	4b	Food	I	N12-0605	3.90e+09	5.00e+07	1.8920946	FALSE	1
1	14.02.18	n	LA	10	CC4	4b	Food	I	N12-1772	3.30e+09	0.00e+00	7.5185139	TRUE	2
1	14.02.18	n	LA	14	CC6	4b	Blood	I	N12-1387	8.00e+08	0.00e+00	6.9030900	TRUE	2
1	14.02.18	n	LA	2	CC1	4b	Food	I	N12-1339	3.40e+09	9.00e+07	1.5772364	FALSE	1
1	14.02.18	n	LA	3	CC1	4b	Food	I	N12-1996	3.50e+09	2.30e+07	2.1823402	FALSE	1
1	14.02.18	n	LA	38	CC415	1/2b	Blood	II	N13-0762	4.10e+09	0.00e+00	7.6127839	TRUE	2
1	14.02.18	n	LA	39	CC54	4b	Blood	I	N13-0177	3.80e+09	4.30e+08	0.9463151	FALSE	1
1	14.02.18	n	LA	4	CC1	4b	Food	I	N13-0047	3.50e+09	8.00e+02	6.6409781	FALSE	2
1	14.02.18	n	LA	40	CC224	1/2b	Blood	I	N12-1608	4.30e+09	2.40e+07	2.2532572	FALSE	1
1	14.02.18	n	LA	5	CC1	4b	Blood	I	N11-2292	3.30e+09	2.30e+08	1.1567861	FALSE	1
1	14.02.18	n	LA	6	CC1	4b	Blood	I	LL195	1.80e+09	2.90e+08	0.7928745	FALSE	1
1	14.02.18	n	LA	7	CC1	4b	Blood	I	N13-0987	1.10e+09	0.00e+00	7.0413927	TRUE	2
1	14.02.18	n	LA	8	CC1	4b	Blood	I	N13-1079	2.10e+09	0.00e+00	7.3222193	TRUE	2
1	14.02.18	n	LA	9	CC6	4b	Food	I	N12-0460	3.40e+09	4.60e+06	2.8687211	FALSE	1
2	08.02.18	a	HCL	25	CC121	1/2a	Food	II	N11-1218	2.50e+09	1.60e+08	1.1938200	FALSE	1
2	08.02.18	a	HCL	26	CC121	1/2a	Food	II	N12-0571	4.20e+09	1.90e+08	1.3444957	FALSE	1
2	08.02.18	a	HCL	27	CC121	1/2a	Food	II	N13-0369	4.80e+09	6.00e+02	6.9030900	FALSE	2
2	08.02.18	a	HCL	29	CC121	1/2a	Food	II	N14-0205	3.80e+09	1.40e+08	1.4336556	FALSE	1
2	08.02.18	a	HCL	30	CC121	3c	Food	II	N14-0322	2.90e+09	2.00e+08	1.1613680	FALSE	1
2	08.02.18	a	HCL	31	CC121	1/2a	Blood	II	N12-0367	3.20e+09	1.10e+03	6.4637573	FALSE	2
2	08.02.18	a	HCL	32	CC121	1/2a	Blood	II	N13-0119	3.70e+09	7.00e+07	1.7231037	FALSE	1
2	08.02.18	a	HCL	33	ST739	1/2a	Food	II	N11-2542	2.70e+09	7.00e+07	1.5862657	FALSE	1
2	08.02.18	a	HCL	34	ST28	1/2a	Food	II	N13-0288	2.50e+09	1.80e+06	3.1426675	FALSE	1
2	08.02.18	a	HCL	35	ST226	1/2a	Food	II	N13-2179	2.70e+09	1.10e+08	1.3899711	FALSE	1
2	08.02.18	a	HCL	36	CC31	1/2a	Food	II	N13-0228	2.20e+09	1.00e+06	3.3424227	FALSE	1
2	08.02.18	a	HCL	37	CC207	1/2a	Blood	II	N12-1107	3.00e+09	1.80e+05	4.2218487	FALSE	2
2	08.02.18	a	LA	25	CC121	1/2a	Food	II	N11-1218	3.00e+09	9.00e+07	1.5228787	FALSE	1
2	08.02.18	a	LA	26	CC121	1/2a	Food	II	N12-0571	4.50e+09	1.10e+08	1.6118198	FALSE	1
2	08.02.18	a	LA	27	CC121	1/2a	Food	II	N13-0369	3.00e+09	3.20e+03	5.9719713	FALSE	2
2	08.02.18	a	LA	29	CC121	1/2a	Food	II	N14-0205	3.60e+09	3.20e+08	1.0511525	FALSE	1
2	08.02.18	a	LA	30	CC121	3c	Food	II	N14-0322	4.40e+09	2.20e+08	1.3010300	FALSE	1
2	08.02.18	a	LA	31	CC121	1/2a	Blood	II	N12-0367	3.70e+09	0.00e+00	7.5682017	TRUE	2

(continued)

replicate	date	adapted.not	acid	strain	clonal.complex	serotype	source	lineage	NENT.Nr	t0	t60	logred	censored	cluster
2	08.02.18	a	LA	32	CC121	1/2a	Blood	II	N13-0119	2.90e+09	2.50e+08	1.0644580	FALSE	1
2	08.02.18	a	LA	33	ST739	1/2a	Food	II	N11-2542	3.60e+09	8.00e+07	1.6532125	FALSE	1
2	08.02.18	a	LA	34	ST28	1/2a	Food	II	N13-0288	3.80e+09	1.00e+02	7.5797836	FALSE	2
2	08.02.18	a	LA	35	ST226	1/2a	Food	II	N13-2179	4.10e+09	3.30e+07	2.0942699	FALSE	1
2	08.02.18	a	LA	36	CC31	1/2a	Food	II	N13-0228	2.10e+09	0.00e+00	7.3222193	TRUE	2
2	08.02.18	a	LA	37	CC207	1/2a	Blood	II	N12-1107	1.60e+09	0.00e+00	7.2041200	TRUE	2
2	08.02.18	n	HCL	25	CC121	1/2a	Food	II	N11-1218	3.50e+09	4.10e+08	0.9312842	FALSE	1
2	08.02.18	n	HCL	26	CC121	1/2a	Food	II	N12-0571	3.60e+09	6.00e+07	1.7781513	FALSE	1
2	08.02.18	n	HCL	27	CC121	1/2a	Food	II	N13-0369	4.00e+09	0.00e+00	7.6020600	TRUE	2
2	08.02.18	n	HCL	29	CC121	1/2a	Food	II	N14-0205	3.20e+09	4.00e+08	0.9030900	FALSE	1
2	08.02.18	n	HCL	30	CC121	3c	Food	II	N14-0322	4.40e+09	2.20e+08	1.3010300	FALSE	1
2	08.02.18	n	HCL	31	CC121	1/2a	Blood	II	N12-0367	3.80e+09	0.00e+00	7.5797836	TRUE	2
2	08.02.18	n	HCL	32	CC121	1/2a	Blood	II	N13-0119	4.90e+09	3.20e+07	2.1850461	FALSE	1
2	08.02.18	n	HCL	33	ST739	1/2a	Food	II	N11-2542	4.60e+09	6.00e+07	1.8846066	FALSE	1
2	08.02.18	n	HCL	34	ST28	1/2a	Food	II	N13-0288	4.20e+09	0.00e+00	7.6232493	TRUE	2
2	08.02.18	n	HCL	35	ST226	1/2a	Food	II	N13-2179	4.30e+09	3.00e+07	2.1563472	FALSE	1
2	08.02.18	n	HCL	36	CC31	1/2a	Food	II	N13-0228	3.40e+09	0.00e+00	7.5314789	TRUE	2
2	08.02.18	n	HCL	37	CC207	1/2a	Blood	II	N12-1107	2.80e+09	0.00e+00	7.4471580	TRUE	2
2	08.02.18	n	LA	25	CC121	1/2a	Food	II	N11-1218	3.50e+09	2.20e+05	4.2016454	FALSE	2
2	08.02.18	n	LA	26	CC121	1/2a	Food	II	N12-0571	3.60e+09	3.70e+05	3.9881008	FALSE	2
2	08.02.18	n	LA	27	CC121	1/2a	Food	II	N13-0369	4.00e+09	0.00e+00	7.6020600	TRUE	2
2	08.02.18	n	LA	29	CC121	1/2a	Food	II	N14-0205	3.20e+09	2.20e+07	2.1627273	FALSE	1
2	08.02.18	n	LA	30	CC121	3c	Food	II	N14-0322	4.40e+09	7.00e+06	2.7983546	FALSE	1
2	08.02.18	n	LA	31	CC121	1/2a	Blood	II	N12-0367	3.80e+09	0.00e+00	7.5797836	TRUE	2
2	08.02.18	n	LA	32	CC121	1/2a	Blood	II	N13-0119	4.90e+09	2.10e+05	4.3679768	FALSE	2
2	08.02.18	n	LA	34	ST28	1/2a	Food	II	N13-0288	4.20e+09	0.00e+00	7.6232493	TRUE	2
2	08.02.18	n	LA	36	CC31	1/2a	Food	II	N13-0228	3.40e+09	0.00e+00	7.5314789	TRUE	2
2	08.02.18	n	LA	37	CC207	1/2a	Blood	II	N12-1107	2.80e+09	0.00e+00	7.4471580	TRUE	2
2	15.02.18	a	HCL	1	CC1	4b	Food	I	N12-0605	2.90e+09	1.20e+08	1.3832168	FALSE	1
2	15.02.18	a	HCL	10	CC4	4b	Food	I	N12-1772	3.60e+09	0.00e+00	7.5563025	TRUE	2
2	15.02.18	a	HCL	14	CC6	4b	Blood	I	N12-1387	1.60e+09	0.00e+00	7.2041200	TRUE	2
2	15.02.18	a	HCL	2	CC1	4b	Food	I	N12-1339	4.20e+09	3.50e+08	1.0791812	FALSE	1
2	15.02.18	a	HCL	3	CC1	4b	Food	I	N12-1996	2.20e+09	9.00e+07	1.3881802	FALSE	1
2	15.02.18	a	HCL	38	CC415	1/2b	Blood	II	N13-0762	1.70e+09	6.00e+02	6.4522977	FALSE	2
2	15.02.18	a	HCL	39	CC54	4b	Blood	I	N13-0177	3.50e+09	5.00e+07	1.8450980	FALSE	1
2	15.02.18	a	HCL	4	CC1	4b	Food	I	N13-0047	3.60e+09	1.60e+04	5.3521825	FALSE	2
2	15.02.18	a	HCL	40	CC224	1/2b	Blood	I	N12-1608	1.00e+09	2.30e+08	0.6382722	FALSE	1
2	15.02.18	a	HCL	5	CC1	4b	Blood	I	N11-2292	5.20e+09	1.50e+08	1.5399121	FALSE	1
2	15.02.18	a	HCL	6	CC1	4b	Blood	I	LL195	4.10e+09	1.80e+08	1.3575114	FALSE	1
2	15.02.18	a	HCL	7	CC1	4b	Blood	I	N13-0987	3.50e+09	3.00e+02	7.0669468	FALSE	2
2	15.02.18	a	HCL	8	CC1	4b	Blood	I	N13-1079	3.20e+09	7.00e+02	6.6600519	FALSE	2
2	15.02.18	a	HCL	9	CC6	4b	Food	I	N12-0460	3.40e+09	1.80e+07	2.2762064	FALSE	1
2	15.02.18	a	LA	1	CC1	4b	Food	I	N12-0605	3.90e+09	2.20e+09	0.2486419	FALSE	1

(continued)

replicate	date	adapted.not	acid	strain	clonal.complex	serotype	source	lineage	NENT.Nr	t0	t60	logred	censored	cluster
2	15.02.18	a	LA	10	CC4	4b	Food	I	N12-1772	1.80e+09	1.00e+03	6.2552725	FALSE	2
2	15.02.18	a	LA	14	CC6	4b	Blood	I	N12-1387	1.20e+09	0.00e+00	7.0791812	TRUE	2
2	15.02.18	a	LA	2	CC1	4b	Food	I	N12-1339	4.10e+09	1.60e+09	0.4086639	FALSE	1
2	15.02.18	a	LA	3	CC1	4b	Food	I	N12-1996	3.30e+09	9.00e+08	0.5642714	FALSE	1
2	15.02.18	a	LA	38	CC415	1/2b	Blood	II	N13-0762	2.50e+09	9.00e+03	5.4436975	FALSE	2
2	15.02.18	a	LA	39	CC54	4b	Blood	I	N13-0177	2.50e+09	7.60e+08	0.5171264	FALSE	1
2	15.02.18	a	LA	4	CC1	4b	Food	I	N13-0047	3.00e+09	2.20e+04	5.1346986	FALSE	2
2	15.02.18	a	LA	40	CC224	1/2b	Blood	I	N12-1608	1.40e+09	4.00e+08	0.5440680	FALSE	1
2	15.02.18	a	LA	5	CC1	4b	Blood	I	N11-2292	2.30e+09	1.60e+09	0.1576079	FALSE	1
2	15.02.18	a	LA	6	CC1	4b	Blood	I	LL195	3.20e+09	1.30e+09	0.3912066	FALSE	1
2	15.02.18	a	LA	7	CC1	4b	Blood	I	N13-0987	2.60e+09	7.00e+02	6.5698753	FALSE	2
2	15.02.18	a	LA	8	CC1	4b	Blood	I	N13-1079	4.30e+09	1.20e+04	5.5542872	FALSE	2
2	15.02.18	a	LA	9	CC6	4b	Food	I	N12-0460	3.30e+09	1.00e+09	0.5185139	FALSE	1
2	15.02.18	n	HCL	1	CC1	4b	Food	I	N12-0605	3.10e+09	2.90e+08	1.0289637	FALSE	1
2	15.02.18	n	HCL	10	CC4	4b	Food	I	N12-1772	2.70e+09	1.00e+02	7.4313638	FALSE	2
2	15.02.18	n	HCL	14	CC6	4b	Blood	I	N12-1387	1.50e+09	4.00e+02	6.5740313	FALSE	2
2	15.02.18	n	HCL	2	CC1	4b	Food	I	N12-1339	2.70e+09	6.00e+08	0.6532125	FALSE	1
2	15.02.18	n	HCL	3	CC1	4b	Food	I	N12-1996	3.10e+09	1.70e+08	1.2609128	FALSE	1
2	15.02.18	n	HCL	38	CC415	1/2b	Blood	II	N13-0762	4.90e+09	0.00e+00	7.6901961	TRUE	2
2	15.02.18	n	HCL	39	CC54	4b	Blood	I	N13-0177	7.50e+09	1.10e+08	1.8336686	FALSE	1
2	15.02.18	n	HCL	4	CC1	4b	Food	I	N13-0047	2.80e+09	9.00e+02	6.4929155	FALSE	2
2	15.02.18	n	HCL	40	CC224	1/2b	Blood	I	N12-1608	1.40e+09	1.10e+08	1.1047354	FALSE	1
2	15.02.18	n	HCL	5	CC1	4b	Blood	I	N11-2292	4.00e+09	2.50e+08	1.2041200	FALSE	1
2	15.02.18	n	HCL	6	CC1	4b	Blood	I	LL195	2.20e+09	7.00e+08	0.4973246	FALSE	1
2	15.02.18	n	HCL	7	CC1	4b	Blood	I	N13-0987	2.60e+09	0.00e+00	7.4149733	TRUE	2
2	15.02.18	n	HCL	8	CC1	4b	Blood	I	N13-1079	3.60e+09	3.30e+03	6.0377886	FALSE	2
2	15.02.18	n	HCL	9	CC6	4b	Food	I	N12-0460	3.20e+09	1.60e+07	2.3010300	FALSE	1
2	15.02.18	n	LA	1	CC1	4b	Food	I	N12-0605	3.10e+09	3.00e+08	1.0142404	FALSE	1
2	15.02.18	n	LA	10	CC4	4b	Food	I	N12-1772	2.70e+09	0.00e+00	7.4313638	TRUE	2
2	15.02.18	n	LA	14	CC6	4b	Blood	I	N12-1387	1.50e+09	0.00e+00	7.1760913	TRUE	2
2	15.02.18	n	LA	2	CC1	4b	Food	I	N12-1339	2.70e+09	1.10e+09	0.3899711	FALSE	1
2	15.02.18	n	LA	3	CC1	4b	Food	I	N12-1996	3.10e+09	7.00e+08	0.6462637	FALSE	1
2	15.02.18	n	LA	38	CC415	1/2b	Blood	II	N13-0762	4.90e+09	1.00e+03	6.6901961	FALSE	2
2	15.02.18	n	LA	39	CC54	4b	Blood	I	N13-0177	7.50e+09	8.00e+08	0.9719713	FALSE	1
2	15.02.18	n	LA	4	CC1	4b	Food	I	N13-0047	2.80e+09	0.00e+00	7.4471580	TRUE	2
2	15.02.18	n	LA	40	CC224	1/2b	Blood	I	N12-1608	1.40e+09	2.40e+08	0.7659168	FALSE	1
2	15.02.18	n	LA	5	CC1	4b	Blood	I	N11-2292	4.00e+09	1.00e+09	0.6020600	FALSE	1
2	15.02.18	n	LA	6	CC1	4b	Blood	I	LL195	2.20e+09	9.00e+08	0.3881802	FALSE	1
2	15.02.18	n	LA	7	CC1	4b	Blood	I	N13-0987	2.60e+09	4.00e+02	6.8129134	FALSE	2
2	15.02.18	n	LA	8	CC1	4b	Blood	I	N13-1079	3.60e+09	9.00e+03	5.6020600	FALSE	2
2	15.02.18	n	LA	9	CC6	4b	Food	I	N12-0460	3.20e+09	1.30e+08	1.3912066	FALSE	1
2	31.01.18	a	HCL	11	CC6	4b	Food	I	N13-0703	2.60e+09	8.00e+03	5.5118834	FALSE	2
2	31.01.18	a	HCL	12	CC6	4b	Food	I	N13-1184	3.70e+09	1.40e+04	5.4220737	FALSE	2

(continued)

replicate	date	adapted.not	acid	strain	clonal.complex	serotype	source	lineage	NENT.Nr	t0	t60	logred	censored	cluster
2	31.01.18	a	HCL	13	CC6	4b	Blood	I	N11-2801	4.10e+09	1.20e+04	5.5336026	FALSE	2
2	31.01.18	a	HCL	15	CC6	4b	Blood	I	N13-1271	3.20e+09	1.50e+04	5.3290587	FALSE	2
2	31.01.18	a	HCL	16	CC6	4b	Blood	I	N13-1507	4.20e+09	5.50e+07	1.8828866	FALSE	1
2	31.01.18	a	HCL	17	CC9	1/2c	Food	II	N11-1698	3.10e+09	2.30e+04	5.1296339	FALSE	2
2	31.01.18	a	HCL	18	CC9	1/2c	Food	II	N12-0710	2.20e+09	2.90e+07	1.8800247	FALSE	1
2	31.01.18	a	HCL	19	CC9	1/2c	Food	II	N12-0822	2.50e+09	2.30e+08	1.0362122	FALSE	1
2	31.01.18	a	HCL	20	CC9	1/2c	Food	II	N11-1848	3.00e+09	1.60e+08	1.2730013	FALSE	1
2	31.01.18	a	HCL	21	CC9	1/2c	Food	II	N14-0261	1.50e+09	3.30e+08	0.6575773	FALSE	1
2	31.01.18	a	HCL	23	CC9	1/2c	Blood	II	N12-0486	2.40e+09	4.50e+05	3.7269987	FALSE	1
2	31.01.18	a	HCL	24	CC9	1/2c	Blood	II	N13-0001	4.90e+09	1.10e+06	3.6488034	FALSE	1
2	31.01.18	a	LA	11	CC6	4b	Food	I	N13-0703	1.10e+09	4.00e+03	5.4393327	FALSE	2
2	31.01.18	a	LA	12	CC6	4b	Food	I	N13-1184	3.60e+09	3.30e+03	6.0377886	FALSE	2
2	31.01.18	a	LA	13	CC6	4b	Blood	I	N11-2801	1.80e+09	1.80e+03	6.0000000	FALSE	2
2	31.01.18	a	LA	15	CC6	4b	Blood	I	N13-1271	1.90e+09	1.50e+03	6.1026623	FALSE	2
2	31.01.18	a	LA	16	CC6	4b	Blood	I	N13-1507	3.20e+09	6.40e+08	0.6989700	FALSE	1
2	31.01.18	a	LA	17	CC9	1/2c	Food	II	N11-1698	1.40e+09	3.60e+03	5.5898255	FALSE	2
2	31.01.18	a	LA	18	CC9	1/2c	Food	II	N12-0710	2.80e+09	5.80e+08	0.6837300	FALSE	1
2	31.01.18	a	LA	19	CC9	1/2c	Food	II	N12-0822	3.40e+09	5.30e+08	0.8072030	FALSE	1
2	31.01.18	a	LA	20	CC9	1/2c	Food	II	N11-1848	3.00e+09	1.70e+09	0.2466723	FALSE	1
2	31.01.18	a	LA	21	CC9	1/2c	Food	II	N14-0261	3.00e+09	6.00e+08	0.6989700	FALSE	1
2	31.01.18	a	LA	22	CC9	1/2a	Blood	II	N11-1837	3.50e+09	0.00e+00	7.5440680	TRUE	2
2	31.01.18	a	LA	23	CC9	1/2c	Blood	II	N12-0486	3.00e+09	2.10e+04	5.1549020	FALSE	2
2	31.01.18	a	LA	24	CC9	1/2c	Blood	II	N13-0001	2.50e+09	2.80e+03	5.9507820	FALSE	2
2	31.01.18	n	HCL	11	CC6	4b	Food	I	N13-0703	2.60e+09	9.00e+04	4.4607308	FALSE	2
2	31.01.18	n	HCL	12	CC6	4b	Food	I	N13-1184	3.80e+09	0.00e+00	7.5797836	TRUE	2
2	31.01.18	n	HCL	13	CC6	4b	Blood	I	N11-2801	3.80e+09	2.00e+03	6.2787536	FALSE	2
2	31.01.18	n	HCL	15	CC6	4b	Blood	I	N13-1271	5.50e+09	0.00e+00	7.7403627	TRUE	2
2	31.01.18	n	HCL	16	CC6	4b	Blood	I	N13-1507	2.60e+09	2.00e+08	1.1139434	FALSE	1
2	31.01.18	n	HCL	17	CC9	1/2c	Food	II	N11-1698	3.30e+09	0.00e+00	7.5185139	TRUE	2
2	31.01.18	n	HCL	18	CC9	1/2c	Food	II	N12-0710	2.50e+09	2.30e+05	4.0362122	FALSE	2
2	31.01.18	n	HCL	19	CC9	1/2c	Food	II	N12-0822	2.00e+09	2.30e+08	0.9393022	FALSE	1
2	31.01.18	n	HCL	20	CC9	1/2c	Food	II	N11-1848	3.20e+09	1.40e+08	1.3590219	FALSE	1
2	31.01.18	n	HCL	21	CC9	1/2c	Food	II	N14-0261	2.20e+09	1.90e+08	1.0636691	FALSE	1
2	31.01.18	n	HCL	22	CC9	1/2a	Blood	II	N11-1837	2.40e+09	0.00e+00	7.3802112	TRUE	2
2	31.01.18	n	HCL	23	CC9	1/2c	Blood	II	N12-0486	2.10e+09	0.00e+00	7.3222193	TRUE	2
2	31.01.18	n	HCL	24	CC9	1/2c	Blood	II	N13-0001	3.10e+09	0.00e+00	7.4913617	TRUE	2
2	31.01.18	n	LA	11	CC6	4b	Food	I	N13-0703	2.60e+09	0.00e+00	7.4149733	TRUE	2
2	31.01.18	n	LA	12	CC6	4b	Food	I	N13-1184	3.80e+09	2.70e+03	6.1484198	FALSE	2
2	31.01.18	n	LA	13	CC6	4b	Blood	I	N11-2801	3.80e+09	1.00e+04	5.5797836	FALSE	2
2	31.01.18	n	LA	15	CC6	4b	Blood	I	N13-1271	5.50e+09	1.10e+03	6.6989700	FALSE	2
2	31.01.18	n	LA	16	CC6	4b	Blood	I	N13-1507	2.60e+09	5.90e+08	0.6441213	FALSE	1
2	31.01.18	n	LA	17	CC9	1/2c	Food	II	N11-1698	3.30e+09	8.00e+03	5.6154240	FALSE	2
2	31.01.18	n	LA	18	CC9	1/2c	Food	II	N12-0710	2.50e+09	5.40e+08	0.6655462	FALSE	1
2	31.01.18	n	LA	19	CC9	1/2c	Food	II	N12-0822	2.00e+09	1.70e+09	0.0705811	FALSE	1

(continued)

replicate	date	adapted.not	acid	strain	clonal.complex	serotype	source	lineage	NENT.Nr	t0	t60	logred	censored	cluster
2	31.01.18	n	LA	20	CC9	1/2c	Food	II	N11-1848	3.20e+09	1.10e+09	0.4637573	FALSE	1
2	31.01.18	n	LA	21	CC9	1/2c	Food	II	N14-0261	2.20e+09	2.50e+09	-0.0555173	FALSE	1
2	31.01.18	n	LA	23	CC9	1/2c	Blood	II	N12-0486	2.10e+09	3.10e+05	3.8308576	FALSE	1
2	31.01.18	n	LA	24	CC9	1/2c	Blood	II	N13-0001	3.10e+09	2.10e+04	5.1691424	FALSE	2
3	06.02.18	a	HCL	11	CC6	4b	Food	I	N13-0703	3.60e+09	0.00e+00	7.5563025	TRUE	2
3	06.02.18	a	HCL	12	CC6	4b	Food	I	N13-1184	1.80e+09	4.50e+06	2.6020600	FALSE	1
3	06.02.18	a	HCL	13	CC6	4b	Blood	I	N11-2801	3.30e+09	0.00e+00	7.5185139	TRUE	2
3	06.02.18	a	HCL	15	CC6	4b	Blood	I	N13-1271	4.80e+09	1.00e+02	7.6812412	FALSE	2
3	06.02.18	a	HCL	16	CC6	4b	Blood	I	N13-1507	2.80e+09	2.00e+07	2.1461280	FALSE	1
3	06.02.18	a	HCL	17	CC9	1/2c	Food	II	N11-1698	2.00e+09	0.00e+00	7.3010300	TRUE	2
3	06.02.18	a	HCL	18	CC9	1/2c	Food	II	N12-0710	2.20e+09	3.10e+07	1.8510610	FALSE	1
3	06.02.18	a	HCL	19	CC9	1/2c	Food	II	N12-0822	2.20e+09	3.00e+07	1.8653014	FALSE	1
3	06.02.18	a	HCL	20	CC9	1/2c	Food	II	N11-1848	3.60e+09	1.50e+08	1.3802112	FALSE	1
3	06.02.18	a	HCL	21	CC9	1/2c	Food	II	N14-0261	2.20e+09	2.10e+08	1.0202034	FALSE	1
3	06.02.18	a	HCL	22	CC9	1/2a	Blood	II	N11-1837	4.20e+09	0.00e+00	7.6232493	TRUE	2
3	06.02.18	a	HCL	23	CC9	1/2c	Blood	II	N12-0486	2.80e+09	0.00e+00	7.4471580	TRUE	2
3	06.02.18	a	HCL	24	CC9	1/2c	Blood	II	N13-0001	1.57e+10	6.00e+02	7.4177484	FALSE	2
3	06.02.18	a	LA	11	CC6	4b	Food	I	N13-0703	3.20e+09	0.00e+00	7.5051500	TRUE	2
3	06.02.18	a	LA	12	CC6	4b	Food	I	N13-1184	4.30e+09	0.00e+00	7.6334685	TRUE	2
3	06.02.18	a	LA	13	CC6	4b	Blood	I	N11-2801	8.00e+08	0.00e+00	6.9030900	TRUE	2
3	06.02.18	a	LA	15	CC6	4b	Blood	I	N13-1271	3.30e+09	2.10e+04	5.1962946	FALSE	2
3	06.02.18	a	LA	16	CC6	4b	Blood	I	N13-1507	3.90e+09	1.10e+08	1.5496719	FALSE	1
3	06.02.18	a	LA	17	CC9	1/2c	Food	II	N11-1698	2.20e+09	0.00e+00	7.3424227	TRUE	2
3	06.02.18	a	LA	18	CC9	1/2c	Food	II	N12-0710	1.60e+09	3.30e+08	0.6856060	FALSE	1
3	06.02.18	a	LA	19	CC9	1/2c	Food	II	N12-0822	3.60e+09	1.10e+09	0.5149098	FALSE	1
3	06.02.18	a	LA	20	CC9	1/2c	Food	II	N11-1848	3.90e+09	2.50e+08	1.1931246	FALSE	1
3	06.02.18	a	LA	21	CC9	1/2c	Food	II	N14-0261	2.10e+09	2.00e+09	0.0211893	FALSE	1
3	06.02.18	a	LA	22	CC9	1/2a	Blood	II	N11-1837	4.00e+09	7.00e+02	6.7569620	FALSE	2
3	06.02.18	a	LA	23	CC9	1/2c	Blood	II	N12-0486	2.10e+09	0.00e+00	7.3222193	TRUE	2
3	06.02.18	a	LA	24	CC9	1/2c	Blood	II	N13-0001	2.40e+09	0.00e+00	7.3802112	TRUE	2
3	06.02.18	n	HCL	11	CC6	4b	Food	I	N13-0703	3.40e+09	0.00e+00	7.5314789	TRUE	2
3	06.02.18	n	HCL	12	CC6	4b	Food	I	N13-1184	4.10e+09	0.00e+00	7.6127839	TRUE	2
3	06.02.18	n	HCL	13	CC6	4b	Blood	I	N11-2801	3.00e+09	0.00e+00	7.4771213	TRUE	2
3	06.02.18	n	HCL	15	CC6	4b	Blood	I	N13-1271	3.20e+09	0.00e+00	7.5051500	TRUE	2
3	06.02.18	n	HCL	16	CC6	4b	Blood	I	N13-1507	3.60e+09	2.10e+08	1.2340832	FALSE	1
3	06.02.18	n	HCL	17	CC9	1/2c	Food	II	N11-1698	1.80e+09	0.00e+00	7.2552725	TRUE	2
3	06.02.18	n	HCL	18	CC9	1/2c	Food	II	N12-0710	2.90e+09	3.80e+06	2.8826144	FALSE	1
3	06.02.18	n	HCL	19	CC9	1/2c	Food	II	N12-0822	2.90e+09	2.80e+08	1.0152400	FALSE	1
3	06.02.18	n	HCL	20	CC9	1/2c	Food	II	N11-1848	2.20e+09	1.30e+08	1.2284793	FALSE	1
3	06.02.18	n	HCL	21	CC9	1/2c	Food	II	N14-0261	3.00e+09	2.20e+08	1.1346986	FALSE	1
3	06.02.18	n	HCL	22	CC9	1/2a	Blood	II	N11-1837	2.20e+09	0.00e+00	7.3424227	TRUE	2
3	06.02.18	n	HCL	23	CC9	1/2c	Blood	II	N12-0486	2.60e+09	0.00e+00	7.4149733	TRUE	2
3	06.02.18	n	HCL	24	CC9	1/2c	Blood	II	N13-0001	4.40e+09	0.00e+00	7.6434527	TRUE	2

(continued)

replicate	date	adapted.not	acid	strain	clonal.complex	serotype	source	lineage	NENT.Nr	t0	t60	logred	censored	cluster
3	06.02.18	n	LA	11	CC6	4b	Food	I	N13-0703	3.40e+09	0.00e+00	7.5314789	TRUE	2
3	06.02.18	n	LA	12	CC6	4b	Food	I	N13-1184	4.10e+09	2.80e+05	4.1656258	FALSE	2
3	06.02.18	n	LA	13	CC6	4b	Blood	I	N11-2801	3.00e+09	0.00e+00	7.4771213	TRUE	2
3	06.02.18	n	LA	15	CC6	4b	Blood	I	N13-1271	3.20e+09	0.00e+00	7.5051500	TRUE	2
3	06.02.18	n	LA	16	CC6	4b	Blood	I	N13-1507	3.60e+09	3.80e+07	1.9765189	FALSE	1
3	06.02.18	n	LA	17	CC9	1/2c	Food	II	N11-1698	1.80e+09	0.00e+00	7.2552725	TRUE	2
3	06.02.18	n	LA	18	CC9	1/2c	Food	II	N12-0710	2.90e+09	1.50e+07	2.2863067	FALSE	1
3	06.02.18	n	LA	19	CC9	1/2c	Food	II	N12-0822	2.90e+09	7.00e+08	0.6173000	FALSE	1
3	06.02.18	n	LA	20	CC9	1/2c	Food	II	N11-1848	2.20e+09	2.50e+07	1.9444827	FALSE	1
3	06.02.18	n	LA	21	CC9	1/2c	Food	II	N14-0261	3.00e+09	5.50e+08	0.7367586	FALSE	1
3	06.02.18	n	LA	22	CC9	1/2a	Blood	II	N11-1837	2.20e+09	0.00e+00	7.3424227	TRUE	2
3	06.02.18	n	LA	23	CC9	1/2c	Blood	II	N12-0486	2.60e+09	0.00e+00	7.4149733	TRUE	2
3	06.02.18	n	LA	24	CC9	1/2c	Blood	II	N13-0001	4.40e+09	0.00e+00	7.6434527	TRUE	2
3	09.02.18	a	HCL	25	CC121	1/2a	Food	II	N11-1218	1.80e+09	3.80e+08	0.6754889	FALSE	1
3	09.02.18	a	HCL	26	CC121	1/2a	Food	II	N12-0571	3.20e+09	4.80e+08	0.8239087	FALSE	1
3	09.02.18	a	HCL	27	CC121	1/2a	Food	II	N13-0369	3.00e+09	9.00e+02	6.5228787	FALSE	2
3	09.02.18	a	HCL	28	CC121	1/2b	Food	II	N13-0836	1.70e+09	4.60e+08	0.5676911	FALSE	1
3	09.02.18	a	HCL	29	CC121	1/2a	Food	II	N14-0205	2.60e+09	3.30e+08	0.8964594	FALSE	1
3	09.02.18	a	HCL	30	CC121	3c	Food	II	N14-0322	2.20e+09	3.20e+08	0.8372727	FALSE	1
3	09.02.18	a	HCL	31	CC121	1/2a	Blood	II	N12-0367	3.00e+09	1.80e+03	6.2218487	FALSE	2
3	09.02.18	a	HCL	32	CC121	1/2a	Blood	II	N13-0119	2.80e+09	2.20e+08	1.1047354	FALSE	1
3	09.02.18	a	HCL	33	ST739	1/2a	Food	II	N11-2542	2.10e+09	2.70e+08	0.8908555	FALSE	1
3	09.02.18	a	HCL	34	ST28	1/2a	Food	II	N13-0288	3.50e+09	7.00e+04	4.6989700	FALSE	2
3	09.02.18	a	HCL	35	ST226	1/2a	Food	II	N13-2179	2.20e+09	2.80e+08	0.8952646	FALSE	1
3	09.02.18	a	HCL	36	CC31	1/2a	Food	II	N13-0228	2.10e+09	1.00e+05	4.3222193	FALSE	2
3	09.02.18	a	HCL	37	CC207	1/2a	Blood	II	N12-1107	3.80e+09	3.00e+02	7.1026623	FALSE	2
3	09.02.18	n	HCL	25	CC121	1/2a	Food	II	N11-1218	2.80e+09	1.70e+09	0.2167091	FALSE	1
3	09.02.18	n	HCL	26	CC121	1/2a	Food	II	N12-0571	3.40e+09	1.70e+09	0.3010300	FALSE	1
3	09.02.18	n	HCL	27	CC121	1/2a	Food	II	N13-0369	3.90e+09	1.40e+03	6.4449366	FALSE	2
3	09.02.18	n	HCL	28	CC121	1/2b	Food	II	N13-0836	1.70e+09	1.30e+09	0.1165056	FALSE	1
3	09.02.18	n	HCL	29	CC121	1/2a	Food	II	N14-0205	3.60e+09	1.40e+09	0.4101745	FALSE	1
3	09.02.18	n	HCL	30	CC121	3c	Food	II	N14-0322	3.80e+09	1.50e+09	0.4036923	FALSE	1
3	09.02.18	n	HCL	31	CC121	1/2a	Blood	II	N12-0367	2.30e+09	1.90e+04	5.0829742	FALSE	2
3	09.02.18	n	HCL	32	CC121	1/2a	Blood	II	N13-0119	3.10e+09	8.00e+08	0.5882717	FALSE	1
3	09.02.18	n	HCL	33	ST739	1/2a	Food	II	N11-2542	3.20e+09	1.90e+09	0.2263964	FALSE	1
3	09.02.18	n	HCL	34	ST28	1/2a	Food	II	N13-0288	4.00e+09	3.00e+02	7.1249387	FALSE	2
3	09.02.18	n	HCL	35	ST226	1/2a	Food	II	N13-2179	2.60e+09	6.00e+08	0.6368221	FALSE	1
3	09.02.18	n	HCL	36	CC31	1/2a	Food	II	N13-0228	4.90e+09	3.00e+08	1.2130748	FALSE	1
3	09.02.18	n	HCL	37	CC207	1/2a	Blood	II	N12-1107	2.80e+09	7.00e+02	6.6020600	FALSE	2
3	09.02.18	n	LA	25	CC121	1/2a	Food	II	N11-1218	2.80e+09	1.20e+09	0.3679768	FALSE	1
3	09.02.18	n	LA	26	CC121	1/2a	Food	II	N12-0571	3.40e+09	3.40e+09	0.0000000	FALSE	1
3	09.02.18	n	LA	28	CC121	1/2b	Food	II	N13-0836	1.70e+09	1.30e+09	0.1165056	FALSE	1
3	09.02.18	n	LA	29	CC121	1/2a	Food	II	N14-0205	3.60e+09	1.30e+09	0.4423591	FALSE	1

(continued)

replicate	date	adapted.not	acid	strain	clonal.complex	serotype	source	lineage	NENT.Nr	t0	t60	logred	censored	cluster
3	09.02.18	n	LA	30	CC121	3c	Food	II	N14-0322	3.80e+09	1.80e+09	0.3245111	FALSE	1
3	09.02.18	n	LA	31	CC121	1/2a	Blood	II	N12-0367	2.30e+09	6.00e+04	4.5835766	FALSE	2
3	09.02.18	n	LA	32	CC121	1/2a	Blood	II	N13-0119	3.10e+09	1.70e+09	0.2609128	FALSE	1
3	09.02.18	n	LA	33	ST739	1/2a	Food	II	N11-2542	3.20e+09	1.10e+09	0.4637573	FALSE	1
3	09.02.18	n	LA	34	ST28	1/2a	Food	II	N13-0288	4.00e+09	2.40e+05	4.2218487	FALSE	2
3	09.02.18	n	LA	35	ST226	1/2a	Food	II	N13-2179	2.60e+09	1.10e+09	0.3735807	FALSE	1
3	09.02.18	n	LA	36	CC31	1/2a	Food	II	N13-0228	4.90e+09	2.30e+06	3.3284682	FALSE	1
3	09.02.18	n	LA	37	CC207	1/2a	Blood	II	N12-1107	2.80e+09	7.00e+03	5.6020600	FALSE	2
3	16.02.18	a	HCL	1	CC1	4b	Food	I	N12-0605	2.70e+09	1.10e+08	1.3899711	FALSE	1
3	16.02.18	a	HCL	10	CC4	4b	Food	I	N12-1772	5.60e+09	1.50e+03	6.5720968	FALSE	2
3	16.02.18	a	HCL	14	CC6	4b	Blood	I	N12-1387	1.30e+09	8.00e+04	4.2108534	FALSE	2
3	16.02.18	a	HCL	2	CC1	4b	Food	I	N12-1339	3.70e+09	2.70e+08	1.1368380	FALSE	1
3	16.02.18	a	HCL	3	CC1	4b	Food	I	N12-1996	2.10e+09	5.00e+07	1.6232493	FALSE	1
3	16.02.18	a	HCL	38	CC415	1/2b	Blood	II	N13-0762	3.00e+09	6.00e+04	4.6989700	FALSE	2
3	16.02.18	a	HCL	39	CC54	4b	Blood	I	N13-0177	4.00e+09	7.00e+07	1.7569620	FALSE	1
3	16.02.18	a	HCL	4	CC1	4b	Food	I	N13-0047	3.60e+09	1.00e+02	7.5563025	FALSE	2
3	16.02.18	a	HCL	40	CC224	1/2b	Blood	I	N12-1608	3.60e+09	3.00e+07	2.0791812	FALSE	1
3	16.02.18	a	HCL	5	CC1	4b	Blood	I	N11-2292	4.30e+09	2.40e+08	1.2532572	FALSE	1
3	16.02.18	a	HCL	6	CC1	4b	Blood	I	LL195	3.70e+09	1.70e+08	1.3377528	FALSE	1
3	16.02.18	a	HCL	7	CC1	4b	Blood	I	N13-0987	1.80e+09	1.70e+04	5.0248236	FALSE	2
3	16.02.18	a	HCL	8	CC1	4b	Blood	I	N13-1079	4.70e+09	3.70e+05	4.1038961	FALSE	2
3	16.02.18	a	HCL	9	CC6	4b	Food	I	N12-0460	2.90e+09	4.40e+07	1.8189453	FALSE	1
3	16.02.18	a	LA	1	CC1	4b	Food	I	N12-0605	4.00e+09	6.00e+08	0.8239087	FALSE	1
3	16.02.18	a	LA	10	CC4	4b	Food	I	N12-1772	2.70e+09	0.00e+00	7.4313638	TRUE	2
3	16.02.18	a	LA	14	CC6	4b	Blood	I	N12-1387	8.00e+08	0.00e+00	6.9030900	TRUE	2
3	16.02.18	a	LA	2	CC1	4b	Food	I	N12-1339	3.50e+09	9.00e+08	0.5898255	FALSE	1
3	16.02.18	a	LA	3	CC1	4b	Food	I	N12-1996	4.90e+09	9.00e+08	0.7359536	FALSE	1
3	16.02.18	a	LA	38	CC415	1/2b	Blood	II	N13-0762	3.00e+09	2.00e+02	7.1760913	FALSE	2
3	16.02.18	a	LA	39	CC54	4b	Blood	I	N13-0177	3.20e+09	1.70e+09	0.2747011	FALSE	1
3	16.02.18	a	LA	4	CC1	4b	Food	I	N13-0047	3.80e+09	1.00e+02	7.5797836	FALSE	2
3	16.02.18	a	LA	40	CC224	1/2b	Blood	I	N12-1608	2.50e+09	5.00e+08	0.6989700	FALSE	1
3	16.02.18	a	LA	5	CC1	4b	Blood	I	N11-2292	4.30e+09	7.00e+08	0.7883704	FALSE	1
3	16.02.18	a	LA	6	CC1	4b	Blood	I	LL195	4.30e+09	7.00e+08	0.7883704	FALSE	1
3	16.02.18	a	LA	7	CC1	4b	Blood	I	N13-0987	3.70e+09	0.00e+00	7.5682017	TRUE	2
3	16.02.18	a	LA	8	CC1	4b	Blood	I	N13-1079	4.70e+09	1.00e+02	7.6720979	FALSE	2
3	16.02.18	a	LA	9	CC6	4b	Food	I	N12-0460	9.00e+08	5.00e+08	0.2552725	FALSE	1
3	16.02.18	n	HCL	1	CC1	4b	Food	I	N12-0605	3.70e+09	3.70e+08	1.0000000	FALSE	1
3	16.02.18	n	HCL	10	CC4	4b	Food	I	N12-1772	4.40e+09	4.30e+05	4.0099842	FALSE	2
3	16.02.18	n	HCL	14	CC6	4b	Blood	I	N12-1387	1.30e+09	0.00e+00	7.1139434	TRUE	2
3	16.02.18	n	HCL	2	CC1	4b	Food	I	N12-1339	3.60e+09	9.00e+08	0.6020600	FALSE	1
3	16.02.18	n	HCL	3	CC1	4b	Food	I	N12-1996	3.50e+09	3.30e+08	1.0255541	FALSE	1
3	16.02.18	n	HCL	38	CC415	1/2b	Blood	II	N13-0762	3.90e+09	0.00e+00	7.5910646	TRUE	2
3	16.02.18	n	HCL	39	CC54	4b	Blood	I	N13-0177	2.90e+09	4.40e+08	0.8189453	FALSE	1
3	16.02.18	n	HCL	4	CC1	4b	Food	I	N13-0047	4.90e+09	4.00e+02	7.0881361	FALSE	2

(continued)

replicate	date	adapted.not	acid	strain	clonal.complex	serotype	source	lineage	NENT.Nr	t0	t60	logred	censored	cluster
3	16.02.18	n	HCL	40	CC224	1/2b	Blood	I	N12-1608	4.00e+09	4.70e+07	1.9299621	FALSE	1
3	16.02.18	n	HCL	5	CC1	4b	Blood	I	N11-2292	3.60e+09	5.00e+08	0.8573325	FALSE	1
3	16.02.18	n	HCL	6	CC1	4b	Blood	I	LL195	3.10e+09	7.00e+08	0.6462637	FALSE	1
3	16.02.18	n	HCL	7	CC1	4b	Blood	I	N13-0987	2.60e+09	0.00e+00	7.4149733	TRUE	2
3	16.02.18	n	HCL	8	CC1	4b	Blood	I	N13-1079	3.60e+09	2.00e+02	7.2552725	FALSE	2
3	16.02.18	n	HCL	9	CC6	4b	Food	I	N12-0460	2.10e+09	1.20e+08	1.2430380	FALSE	1
3	16.02.18	n	LA	1	CC1	4b	Food	I	N12-0605	3.70e+09	5.30e+08	0.8439259	FALSE	1
3	16.02.18	n	LA	10	CC4	4b	Food	I	N12-1772	4.40e+09	0.00e+00	7.6434527	TRUE	2
3	16.02.18	n	LA	14	CC6	4b	Blood	I	N12-1387	1.30e+09	0.00e+00	7.1139434	TRUE	2
3	16.02.18	n	LA	2	CC1	4b	Food	I	N12-1339	3.60e+09	2.20e+07	2.2138798	FALSE	1
3	16.02.18	n	LA	3	CC1	4b	Food	I	N12-1996	3.50e+09	1.50e+08	1.3679768	FALSE	1
3	16.02.18	n	LA	38	CC415	1/2b	Blood	II	N13-0762	3.90e+09	0.00e+00	7.5910646	TRUE	2
3	16.02.18	n	LA	39	CC54	4b	Blood	I	N13-0177	2.90e+09	3.20e+08	0.9572480	FALSE	1
3	16.02.18	n	LA	4	CC1	4b	Food	I	N13-0047	4.90e+09	0.00e+00	7.6901961	TRUE	2
3	16.02.18	n	LA	40	CC224	1/2b	Blood	I	N12-1608	4.00e+09	6.00e+06	2.8239087	FALSE	1
3	16.02.18	n	LA	5	CC1	4b	Blood	I	N11-2292	3.60e+09	2.20e+08	1.2138798	FALSE	1
3	16.02.18	n	LA	6	CC1	4b	Blood	I	LL195	3.10e+09	2.60e+08	1.0763883	FALSE	1
3	16.02.18	n	LA	7	CC1	4b	Blood	I	N13-0987	2.60e+09	0.00e+00	7.4149733	TRUE	2
3	16.02.18	n	LA	8	CC1	4b	Blood	I	N13-1079	3.60e+09	0.00e+00	7.5563025	TRUE	2
3	16.02.18	n	LA	9	CC6	4b	Food	I	N12-0460	2.10e+09	1.70e+07	2.0917704	FALSE	1
3	20.02.18	a	LA	25	CC121	1/2a	Food	II	N11-1218	2.00e+09	1.80e+09	0.0457575	FALSE	1
3	20.02.18	a	LA	26	CC121	1/2a	Food	II	N12-0571	2.70e+09	1.20e+09	0.3521825	FALSE	1
3	20.02.18	a	LA	27	CC121	1/2a	Food	II	N13-0369	3.70e+09	3.20e+03	6.0630517	FALSE	2
3	20.02.18	a	LA	28	CC121	1/2b	Food	II	N13-0836	1.30e+09	1.50e+09	-0.0621479	FALSE	1
3	20.02.18	a	LA	29	CC121	1/2a	Food	II	N14-0205	1.30e+09	9.00e+08	0.1597008	FALSE	1
3	20.02.18	a	LA	30	CC121	3c	Food	II	N14-0322	1.80e+09	1.20e+09	0.1760913	FALSE	1
3	20.02.18	a	LA	31	CC121	1/2a	Blood	II	N12-0367	2.80e+09	4.90e+03	5.7569620	FALSE	2
3	20.02.18	a	LA	32	CC121	1/2a	Blood	II	N13-0119	2.60e+09	1.40e+09	0.2688453	FALSE	1
3	20.02.18	a	LA	33	ST739	1/2a	Food	II	N11-2542	1.80e+09	1.80e+09	0.0000000	FALSE	1
3	20.02.18	a	LA	34	ST28	1/2a	Food	II	N13-0288	2.80e+09	3.30e+04	4.9286441	FALSE	2
3	20.02.18	a	LA	35	ST226	1/2a	Food	II	N13-2179	3.00e+09	8.00e+08	0.5740313	FALSE	1
3	20.02.18	a	LA	36	CC31	1/2a	Food	II	N13-0228	2.30e+09	1.80e+07	2.1064553	FALSE	1
3	20.02.18	a	LA	37	CC207	1/2a	Blood	II	N12-1107	3.40e+09	1.70e+03	6.3010300	FALSE	2
4	20.02.18	a	HCL	18	CC9	1/2c	Food	II	N12-0710	2.90e+09	6.00e+07	1.6842467	FALSE	1
4	20.02.18	a	HCL	22	CC9	1/2a	Blood	II	N11-1837	1.90e+09	0.00e+00	7.2787536	TRUE	2
4	20.02.18	a	LA	28	CC121	1/2b	Food	II	N13-0836	2.00e+09	7.00e+08	0.4559320	FALSE	1
4	20.02.18	n	HCL	18	CC9	1/2c	Food	II	N12-0710	2.20e+09	3.20e+07	1.8372727	FALSE	1
4	20.02.18	n	HCL	22	CC9	1/2a	Blood	II	N11-1837	2.60e+09	1.20e+03	6.3357921	FALSE	2
4	20.02.18	n	HCL	28	CC121	1/2b	Food	II	N13-0836	2.00e+09	9.00e+08	0.3467875	FALSE	1
4	20.02.18	n	HCL	32	CC121	1/2a	Blood	II	N13-0119	3.00e+09	5.00e+08	0.7781513	FALSE	1
4	20.02.18	n	HCL	36	CC31	1/2a	Food	II	N13-0228	1.80e+09	8.00e+02	6.3521825	FALSE	2
4	20.02.18	n	LA	18	CC9	1/2c	Food	II	N12-0710	3.20e+09	2.70e+07	2.0737862	FALSE	1
4	20.02.18	n	LA	22	CC9	1/2a	Blood	II	N11-1837	2.10e+09	0.00e+00	7.3222193	TRUE	2

(continued)

replicate	date	adapted.not	acid	strain	clonal.complex	serotype	source	lineage	NENT.Nr	t0	t60	logred	censored	cluster
4	20.02.18	n	LA	25	CC121	1/2a	Food	II	N11-1218	2.20e+09	1.30e+09	0.2284793	FALSE	1
4	20.02.18	n	LA	26	CC121	1/2a	Food	II	N12-0571	3.10e+09	1.60e+09	0.2872417	FALSE	1
4	20.02.18	n	LA	27	CC121	1/2a	Food	II	N13-0369	2.20e+09	2.90e+03	5.8800247	FALSE	2
4	20.02.18	n	LA	28	CC121	1/2b	Food	II	N13-0836	2.00e+09	8.00e+08	0.3979400	FALSE	1
4	20.02.18	n	LA	29	CC121	1/2a	Food	II	N14-0205	3.00e+09	1.70e+09	0.2466723	FALSE	1
4	20.02.18	n	LA	3	CC1	4b	Food	I	N12-1996	2.30e+09	1.30e+09	0.2477845	FALSE	1
4	20.02.18	n	LA	30	CC121	1/2a	Food	II	N14-0205	2.20e+09	1.70e+09	0.1119738	FALSE	1
4	20.02.18	n	LA	32	CC121	3c	Food	II	N14-0322	3.00e+09	1.10e+09	0.4357286	FALSE	1
4	20.02.18	n	LA	33	ST739	1/2a	Food	II	N11-2542	3.30e+09	1.40e+09	0.3723859	FALSE	1
4	20.02.18	n	LA	35	ST226	1/2a	Food	II	N13-2179	3.90e+09	1.10e+09	0.5496719	FALSE	1
5	20.02.18	a	HCL	28	CC121	1/2b	Food	II	N13-0836	3.70e+09	3.00e+08	1.0910805	FALSE	1
5	20.02.18	a	HCL	36	CC31	1/2a	Food	II	N13-0228	2.60e+09	2.30e+05	4.0532455	FALSE	2

OK, those look like reasonable clusters.

Adapted or not

Let's try to answer the questions by modeling which cluster an observation falls into. We'll fit a logistic regression, with the response being a "success" if an observation is in cluster 2 (the "susceptible"/high log-reduction cluster). First, let's glance at the adaptation setp:

```
m_adapt <- glm( I(cluster==2) ~ NENT.Nr + acid + adapted.not,
               data=acid,
               family="binomial" )
m_adapt
```

```
##
## Call:  glm(formula = I(cluster == 2) ~ NENT.Nr + acid + adapted.not,
##     family = "binomial", data = acid)
##
## Coefficients:
##     (Intercept)  NENT.NrN14-0261  NENT.NrN14-0205  NENT.NrN12-0822
##      -2.292e+01       -8.309e-08       -1.551e-01       -8.301e-08
## NENT.NrN11-2542  NENT.NrN14-0322     NENT.NrLL195  NENT.NrN11-1218
##      -8.301e-08       -8.583e-02       -8.301e-08        1.897e+01
## NENT.NrN11-2292  NENT.NrN12-1339  NENT.NrN13-0177  NENT.NrN13-2179
##      -8.301e-08       -8.285e-08       -8.301e-08       -8.296e-08
## NENT.NrN12-0571  NENT.NrN12-0605  NENT.NrN11-1848  NENT.NrN12-1996
##       1.897e+01       -8.304e-08       -8.303e-08       -8.583e-02
## NENT.NrN13-0119  NENT.NrN13-1507  NENT.NrN12-1608  NENT.NrN12-0460
##       1.904e+01       -8.301e-08       -8.301e-08       -8.301e-08
## NENT.NrN12-0710  NENT.NrN13-0228  NENT.NrN13-0288  NENT.NrN12-0367
##       1.915e+01        2.256e+01        2.451e+01        4.365e+01
## NENT.NrN13-1184  NENT.NrN12-1107  NENT.NrN12-0486  NENT.NrN13-1079
##       2.451e+01        4.365e+01        2.367e+01        4.365e+01
## NENT.NrN12-1387  NENT.NrN13-0369  NENT.NrN13-0762  NENT.NrN13-0047
##       4.365e+01        4.365e+01        4.365e+01        4.365e+01
## NENT.NrN11-1698  NENT.NrN11-2801  NENT.NrN13-0703  NENT.NrN13-0001
##       4.365e+01        4.365e+01        4.365e+01        2.451e+01
## NENT.NrN13-1271  NENT.NrN13-0987  NENT.NrN12-1772  NENT.NrN11-1837
##       4.365e+01        4.365e+01        4.365e+01        4.361e+01
##          acidLA     adapted.notn
##       6.167e-01        1.575e+00
##
## Degrees of Freedom: 489 Total (i.e. Null);  448 Residual
## Null Deviance:       676
## Residual Deviance: 71.1  AIC: 155.1
```

```
summary(m_adapt)
```

```
##
## Call:
## glm(formula = I(cluster == 2) ~ NENT.Nr + acid + adapted.not,
##     family = "binomial", data = acid)
##
## Deviance Residuals:
##      Min        1Q    Median        3Q       Max
## -2.44403  -0.00003  -0.00001   0.00003   2.14703
```

```
##
## Coefficients:
##                   Estimate Std. Error z value Pr(>|z|)
## (Intercept)     -2.292e+01  8.155e+03  -0.003   0.9978
## NENT.NrN14-0261 -8.309e-08  1.153e+04   0.000   1.0000
## NENT.NrN14-0205 -1.551e-01  1.112e+04   0.000   1.0000
## NENT.NrN12-0822 -8.301e-08  1.153e+04   0.000   1.0000
## NENT.NrN11-2542 -8.301e-08  1.153e+04   0.000   1.0000
## NENT.NrN14-0322 -8.583e-02  1.131e+04   0.000   1.0000
## NENT.NrLL195    -8.301e-08  1.153e+04   0.000   1.0000
## NENT.NrN11-1218  1.897e+01  8.155e+03   0.002   0.9981
## NENT.NrN11-2292 -8.301e-08  1.153e+04   0.000   1.0000
## NENT.NrN12-1339 -8.285e-08  1.153e+04   0.000   1.0000
## NENT.NrN13-0177 -8.301e-08  1.153e+04   0.000   1.0000
## NENT.NrN13-2179 -8.296e-08  1.153e+04   0.000   1.0000
## NENT.NrN12-0571  1.897e+01  8.155e+03   0.002   0.9981
## NENT.NrN12-0605 -8.304e-08  1.153e+04   0.000   1.0000
## NENT.NrN11-1848 -8.303e-08  1.153e+04   0.000   1.0000
## NENT.NrN12-1996 -8.583e-02  1.131e+04   0.000   1.0000
## NENT.NrN13-0119  1.904e+01  8.155e+03   0.002   0.9981
## NENT.NrN13-1507 -8.301e-08  1.153e+04   0.000   1.0000
## NENT.NrN12-1608 -8.301e-08  1.153e+04   0.000   1.0000
## NENT.NrN12-0460 -8.301e-08  1.153e+04   0.000   1.0000
## NENT.NrN12-0710  1.915e+01  8.155e+03   0.002   0.9981
## NENT.NrN13-0228  2.256e+01  8.155e+03   0.003   0.9978
## NENT.NrN13-0288  2.451e+01  8.155e+03   0.003   0.9976
## NENT.NrN12-0367  4.365e+01  1.153e+04   0.004   0.9970
## NENT.NrN13-1184  2.451e+01  8.155e+03   0.003   0.9976
## NENT.NrN12-1107  4.365e+01  1.153e+04   0.004   0.9970
## NENT.NrN12-0486  2.367e+01  8.155e+03   0.003   0.9977
## NENT.NrN13-1079  4.365e+01  1.153e+04   0.004   0.9970
## NENT.NrN12-1387  4.365e+01  1.153e+04   0.004   0.9970
## NENT.NrN13-0369  4.365e+01  1.153e+04   0.004   0.9970
## NENT.NrN13-0762  4.365e+01  1.153e+04   0.004   0.9970
## NENT.NrN13-0047  4.365e+01  1.153e+04   0.004   0.9970
## NENT.NrN11-1698  4.365e+01  1.153e+04   0.004   0.9970
## NENT.NrN11-2801  4.365e+01  1.153e+04   0.004   0.9970
## NENT.NrN13-0703  4.365e+01  1.153e+04   0.004   0.9970
## NENT.NrN13-0001  2.451e+01  8.155e+03   0.003   0.9976
## NENT.NrN13-1271  4.365e+01  1.153e+04   0.004   0.9970
## NENT.NrN13-0987  4.365e+01  1.153e+04   0.004   0.9970
## NENT.NrN12-1772  4.365e+01  1.153e+04   0.004   0.9970
## NENT.NrN11-1837  4.361e+01  1.131e+04   0.004   0.9969
## acidLA           6.167e-01  6.370e-01   0.968   0.3330
## adapted.notn     1.575e+00  7.234e-01   2.177   0.0295 *
## ---
## Signif. codes:  0 '***' 0.001 '**' 0.01 '*' 0.05 '.' 0.1 ' ' 1
##
## (Dispersion parameter for binomial family taken to be 1)
##
##     Null deviance: 676.015  on 489  degrees of freedom
## Residual deviance:  71.101  on 448  degrees of freedom
## AIC: 155.1
##
```

```
## Number of Fisher Scoring iterations: 20
```

```
anova(m_adapt,update(m_adapt,.~.-adapted.not),test="Chisq")
```

```
## Analysis of Deviance Table
##
## Model 1: I(cluster == 2) ~ NENT.Nr + acid + adapted.not
## Model 2: I(cluster == 2) ~ NENT.Nr + acid
##   Resid. Df Resid. Dev Df Deviance Pr(>Chi)
## 1       448     71.101
## 2       449     76.824 -1  -5.7234  0.01674 *
## ---
## Signif. codes:  0 '***' 0.001 '**' 0.01 '*' 0.05 '.' 0.1 ' ' 1
```

```
lsmeans(m_adapt,~adapted.not) %>%
  summary() %>%
  data.frame() %>%
  mutate(lsmean.p = plogis(lsmean),
         asymp.LCL.p = plogis(asymp.LCL),
         asymp.UCL.p = plogis(asymp.UCL)) %>%
  ggplot(aes(x=adapted.not,y=lsmean.p)) +
  geom_point() +
  ylim(0,1) +
  geom_errorbar(aes(ymin=asymp.LCL.p,ymax=asymp.UCL.p),width=0.5)
```

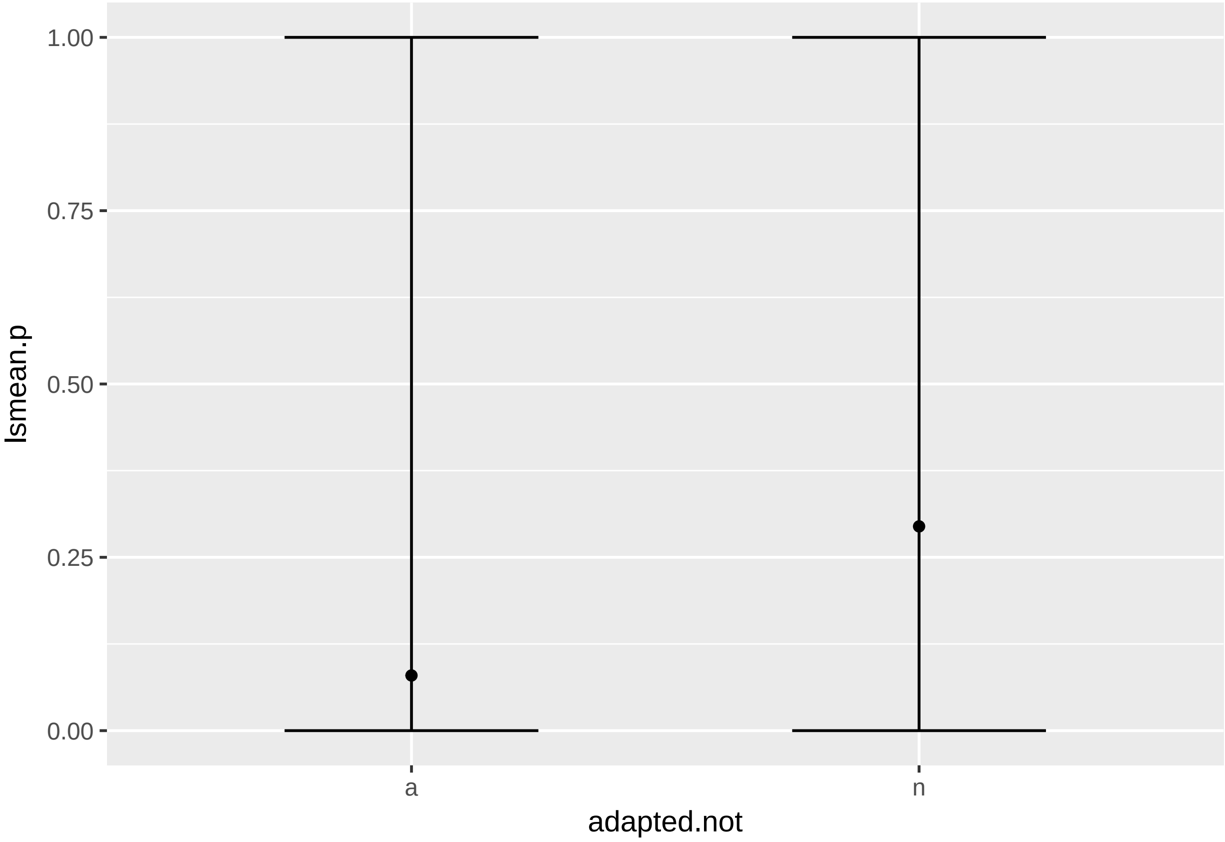

Like we suspected, the model with `NENT.Nr + acid + adapted.not` is not significantly better than the model with just `NENT.Nr + acid`.

The main takeaway from looking at this plot is not the point estimates for the LS-means of

the adaptation groups (which ought to be in the 0.45 ballpark rather than 0.02) – I think the main point here is that the confidence limits span the entire range from 0 to 1, so these are bad estimates and `adapted.not` isn't a good predictor of acid susceptibility

Then we can look at CC, serotype, source, and lineage:

Clonal Complex

```
m_cc <- glm(I(cluster==2)~clonal.complex+acid+adapted.not,data=acid,family="binomial")
summary(m_cc)
```

```
##
## Call:
## glm(formula = I(cluster == 2) ~ clonal.complex + acid + adapted.not,
##     family = "binomial", data = acid)
##
## Deviance Residuals:
##      Min        1Q    Median        3Q       Max
## -2.19363  -0.93973  -0.00013   0.87198   1.67327
##
## Coefficients:
##                       Estimate Std. Error z value Pr(>|z|)
## (Intercept)           -0.61779    0.26029  -2.373  0.01762 *
## clonal.complexCC121   -0.49894    0.30776  -1.621  0.10498
## clonal.complexCC207   19.09646 1881.53155   0.010  0.99190
## clonal.complexCC224  -18.03843 1881.53155  -0.010  0.99235
## clonal.complexCC31     1.11970    0.59672   1.876  0.06060 .
## clonal.complexCC4     19.09646 1881.53155   0.010  0.99190
## clonal.complexCC415   19.09646 1881.53155   0.010  0.99190
## clonal.complexCC54   -18.03843 1881.53155  -0.010  0.99235
## clonal.complexCC6      1.38883    0.31826   4.364 1.28e-05 ***
## clonal.complexCC9      0.46643    0.29253   1.594  0.11083
## clonal.complexST226  -18.03843 1881.53155  -0.010  0.99235
## clonal.complexST28     2.92929    1.06570   2.749  0.00598 **
## clonal.complexST739  -18.03843 1881.53155  -0.010  0.99235
## acidLA                 0.02919    0.21299   0.137  0.89098
## adapted.notn           0.14837    0.21311   0.696  0.48629
## ---
## Signif. codes:  0 '***' 0.001 '**' 0.01 '*' 0.05 '.' 0.1 ' ' 1
##
## (Dispersion parameter for binomial family taken to be 1)
##
##     Null deviance: 676.02  on 489  degrees of freedom
## Residual deviance: 507.14  on 475  degrees of freedom
## AIC: 537.14
##
## Number of Fisher Scoring iterations: 17
```

```
anova(m_cc,update(m_cc,.~.-clonal.complex),test="Chisq")
```

```
## Analysis of Deviance Table
##
## Model 1: I(cluster == 2) ~ clonal.complex + acid + adapted.not
## Model 2: I(cluster == 2) ~ acid + adapted.not
```

```
##   Resid. Df Resid. Dev  Df Deviance  Pr(>Chi)
## 1       475     507.14
## 2       487     675.78 -12  -168.64 < 2.2e-16 ***
## ---
## Signif. codes:  0 '***' 0.001 '**' 0.01 '*' 0.05 '.' 0.1 ' ' 1
```

```
lsmeans(m_cc,~clonal.complex) %>%
  summary() %>%
  data.frame() %>%
  mutate(lsmean.p = plogis(lsmean),
         asymp.LCL.p = plogis(asymp.LCL),
         asymp.UCL.p = plogis(asymp.UCL)) %>%
  mutate(asymp.UCL.p=ifelse(asymp.UCL.p-asymp.LCL.p>.99,lsmean.p,asymp.UCL.p),
         asymp.LCL.p=ifelse(asymp.UCL.p-asymp.LCL.p>.99,lsmean.p,asymp.LCL.p)) %>%
  ggplot(aes(x=clonal.complex,y=lsmean.p)) +
  geom_point() +
  ylim(0,1) +
  geom_errorbar(aes(ymin=asymp.LCL.p,ymax=asymp.UCL.p),width=0.5)
```

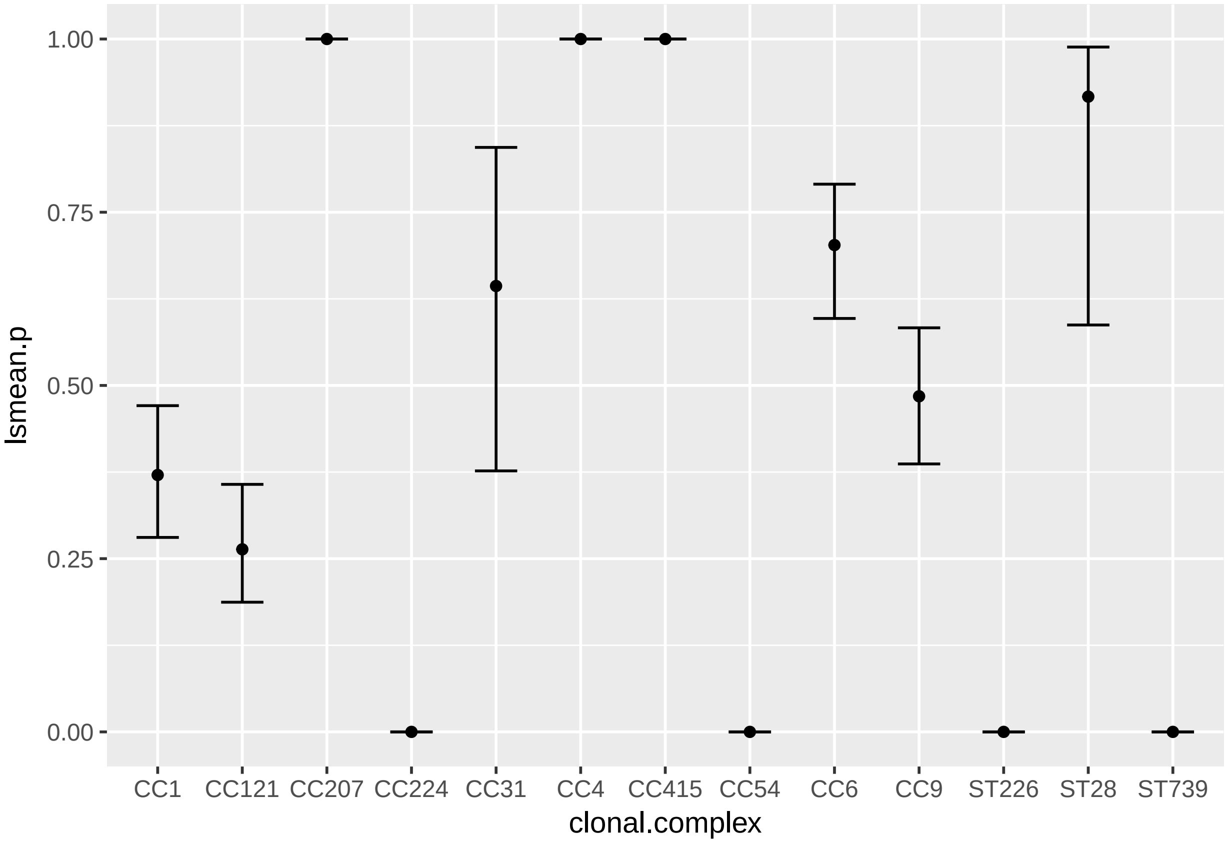

Yes, there is an effect if we look by `clonal.complex`.

Update: If you present this graph, I would put a note that CC207, CC4, and CC415 had only acid-susceptible observations, while CC224 and CC54 had only non-acid-susceptible observations, which is why the error bars are collapsed. In this case I did force them to be collapsed; otherwise they would span from 0 to 1, since it's not possible to get a realistic estimate of the variance of those observations.

Serotype

```
m_s <- glm(I(cluster==2)~serotype+acid+adapted.not,data=acid,family="binomial")
summary(m_s)
```

```
##
## Call:
## glm(formula = I(cluster == 2) ~ serotype + acid + adapted.not,
##     family = "binomial", data = acid)
##
## Deviance Residuals:
##     Min       1Q   Median       3Q      Max
## -1.2372  -1.1508  -0.8841   1.1622   1.5058
##
## Coefficients:
##                Estimate Std. Error z value Pr(>|z|)
## (Intercept)   -0.159158   0.209995  -0.758    0.449
## serotype1/2b  -0.586215   0.389197  -1.506    0.132
## serotype1/2c  -0.278564   0.275414  -1.011    0.312
## serotype3c   -16.462889 665.177287  -0.025    0.980
## serotype4b     0.194979   0.214427   0.909    0.363
## acidLA         0.007544   0.184733   0.041    0.967
## adapted.notn   0.096131   0.184789   0.520    0.603
##
## (Dispersion parameter for binomial family taken to be 1)
##
##     Null deviance: 676.02  on 489  degrees of freedom
## Residual deviance: 653.03  on 483  degrees of freedom
## AIC: 667.03
##
## Number of Fisher Scoring iterations: 15
```

```
anova(m_s,update(m_s,.~.-serotype),test="Chisq")
```

```
## Analysis of Deviance Table
##
## Model 1: I(cluster == 2) ~ serotype + acid + adapted.not
## Model 2: I(cluster == 2) ~ acid + adapted.not
##   Resid. Df Resid. Dev Df Deviance  Pr(>Chi)
## 1       483     653.03
## 2       487     675.78 -4  -22.753 0.0001419 ***
## ---
## Signif. codes:  0 '***' 0.001 '**' 0.01 '*' 0.05 '.' 0.1 ' ' 1
```

```
lsmeans(m_s,~serotype) %>%
  summary() %>%
  data.frame() %>%
  mutate(lsmean.p = plogis(lsmean),
         asymp.LCL.p = plogis(asymp.LCL),
         asymp.UCL.p = plogis(asymp.UCL)) %>%
  ggplot(aes(x=serotype,y=lsmean.p)) +
  geom_point() +
  ylim(0,1) +
  geom_errorbar(aes(ymin=asymp.LCL.p,ymax=asymp.UCL.p),width=0.5)
```

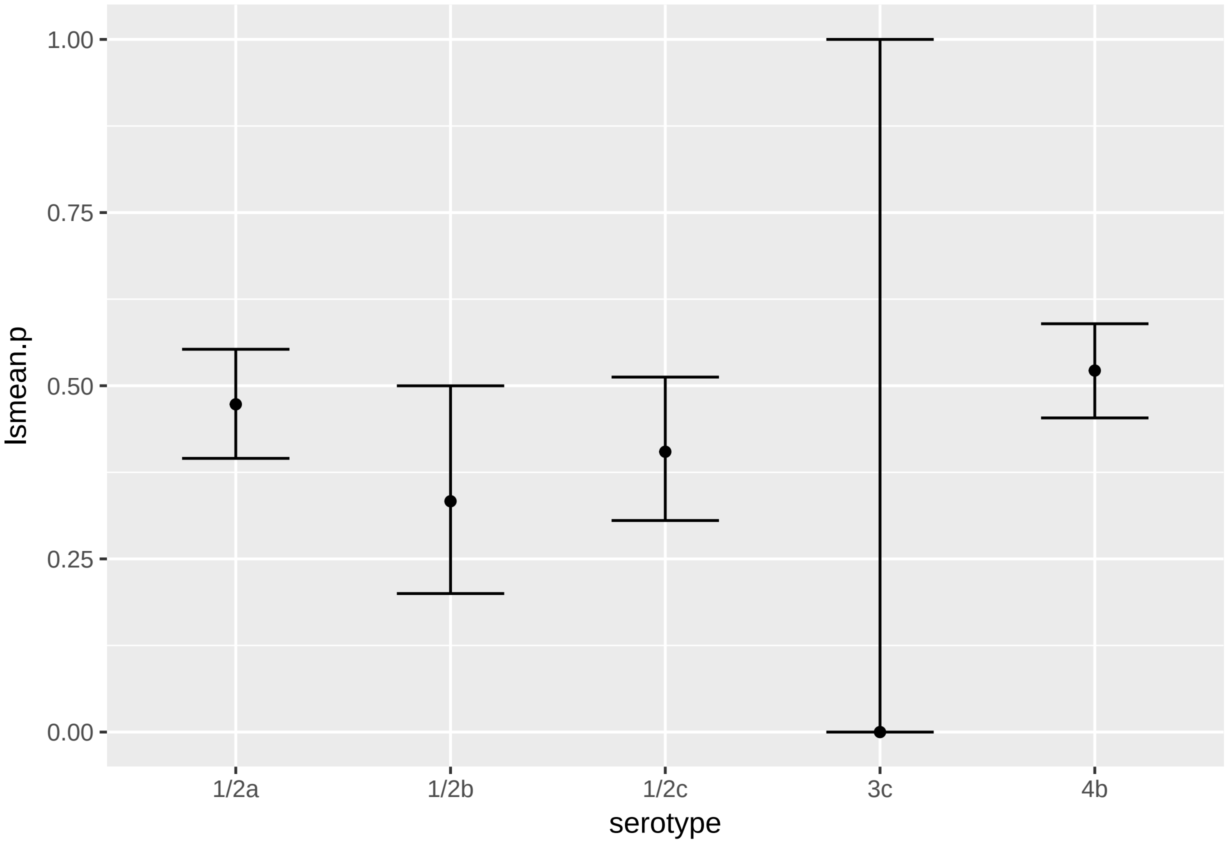

Update: There does perhaps appear to be something going on if we look by `serotype` but it's not quite significant in the classical sense.

Lineage

```
m_l <- glm(I(cluster==2)~lineage+acid+adapted.not,data=acid,family="binomial")
summary(m_l)
```

```
##
## Call:
## glm(formula = I(cluster == 2) ~ lineage + acid + adapted.not,
##     family = "binomial", data = acid)
##
## Deviance Residuals:
##    Min      1Q  Median      3Q     Max
## -1.185  -1.082  -1.045   1.209   1.316
##
## Coefficients:
##               Estimate Std. Error z value Pr(>|z|)
## (Intercept)  -0.073365   0.187044  -0.392    0.695
## lineageII    -0.246165   0.182716  -1.347    0.178
## acidLA        0.000502   0.181693   0.003    0.998
## adapted.notn  0.090477   0.181743   0.498    0.619
##
## (Dispersion parameter for binomial family taken to be 1)
##
```

```
##     Null deviance: 676.02  on 489  degrees of freedom
## Residual deviance: 673.96  on 486  degrees of freedom
## AIC: 681.96
##
## Number of Fisher Scoring iterations: 3
```

```
anova(m_l,update(m_l,.~.-lineage),test="Chisq")
```

```
## Analysis of Deviance Table
##
## Model 1: I(cluster == 2) ~ lineage + acid + adapted.not
## Model 2: I(cluster == 2) ~ acid + adapted.not
##   Resid. Df Resid. Dev Df Deviance Pr(>Chi)
## 1       486     673.96
## 2       487     675.78 -1  -1.8173   0.1776
```

```
lsmeans(m_l,~lineage) %>%
  summary() %>%
  data.frame() %>%
  mutate(lsmean.p = plogis(lsmean),
         asymp.LCL.p = plogis(asymp.LCL),
         asymp.UCL.p = plogis(asymp.UCL)) %>%
  ggplot(aes(x=lineage,y=lsmean.p)) +
  geom_point() +
  ylim(0,1) +
  geom_errorbar(aes(ymin=asymp.LCL.p,ymax=asymp.UCL.p),width=0.5)
```

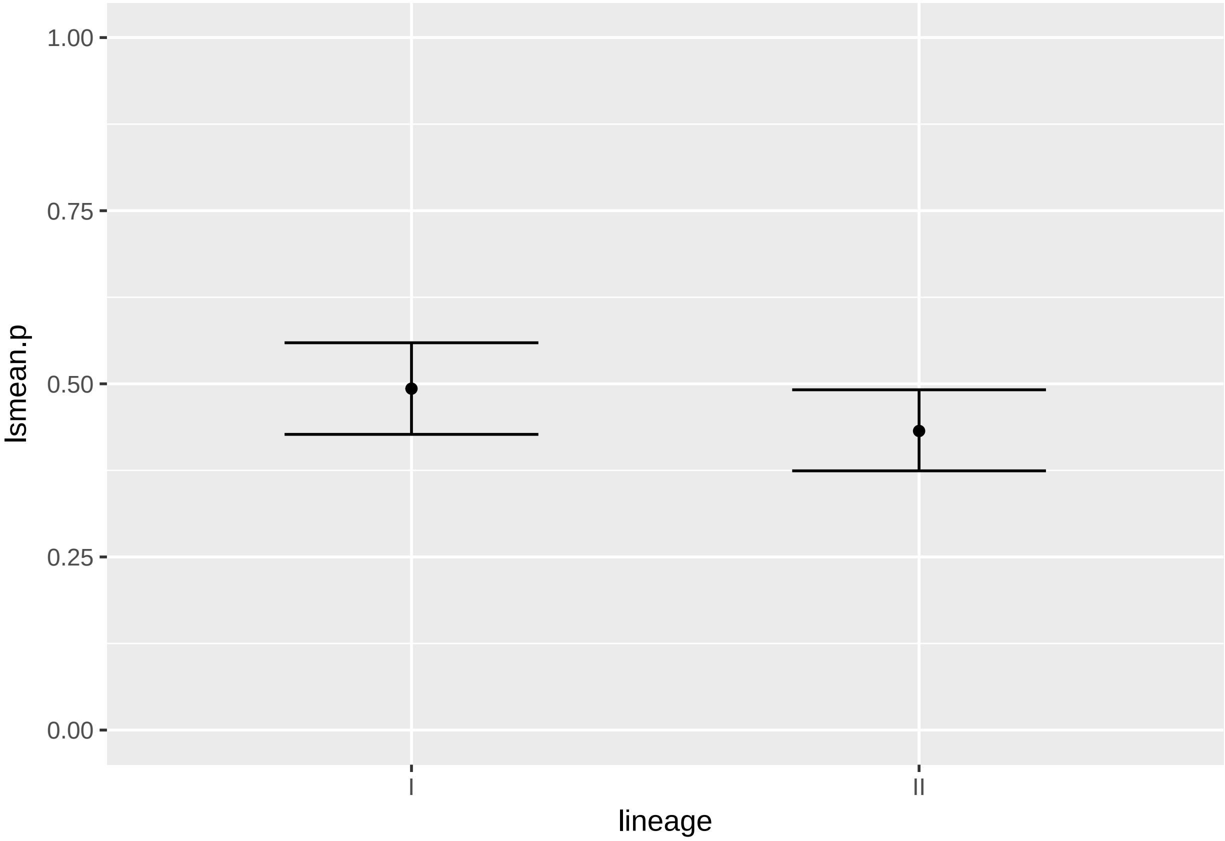

If we look by `lineage`, we don't see a significant effect.

Source

```
m_source <- glm(I(cluster==2)~source+acid+adapted.not,data=acid,family="binomial")
summary(m_source)
```

```
##
## Call:
## glm(formula = I(cluster == 2) ~ source + acid + adapted.not,
##     family = "binomial", data = acid)
##
## Deviance Residuals:
##     Min       1Q   Median       3Q      Max
## -1.4484  -0.9183  -0.8740   0.9747   1.5149
##
## Coefficients:
##              Estimate Std. Error z value Pr(>|z|)
## (Intercept)   0.49758    0.19663   2.531   0.0114 *
## sourceFood   -1.26307    0.19213  -6.574  4.9e-11 ***
## acidLA        0.01636    0.19028   0.086   0.9315
## adapted.notn  0.10381    0.19034   0.545   0.5855
## ---
## Signif. codes:  0 '***' 0.001 '**' 0.01 '*' 0.05 '.' 0.1 ' ' 1
##
## (Dispersion parameter for binomial family taken to be 1)
##
##     Null deviance: 676.02  on 489  degrees of freedom
## Residual deviance: 630.46  on 486  degrees of freedom
## AIC: 638.46
##
## Number of Fisher Scoring iterations: 4
```

```
anova(m_source,update(m_source,.~.-source),test="Chisq")
```

```
## Analysis of Deviance Table
##
## Model 1: I(cluster == 2) ~ source + acid + adapted.not
## Model 2: I(cluster == 2) ~ acid + adapted.not
##   Resid. Df Resid. Dev Df Deviance  Pr(>Chi)
## 1       486     630.46
## 2       487     675.78 -1  -45.319 1.674e-11 ***
## ---
## Signif. codes:  0 '***' 0.001 '**' 0.01 '*' 0.05 '.' 0.1 ' ' 1
```

```
lsmeans(m_source,~source) %>%
  summary() %>%
  data.frame() %>%
  mutate(lsmean.p = plogis(lsmean),
         asymp.LCL.p = plogis(asymp.LCL),
         asymp.UCL.p = plogis(asymp.UCL)) %>%
  ggplot(aes(x=source,y=lsmean.p)) +
  geom_point() +
  ylim(0,1) +
  geom_errorbar(aes(ymin=asymp.LCL.p,ymax=asymp.UCL.p),width=0.5)
```

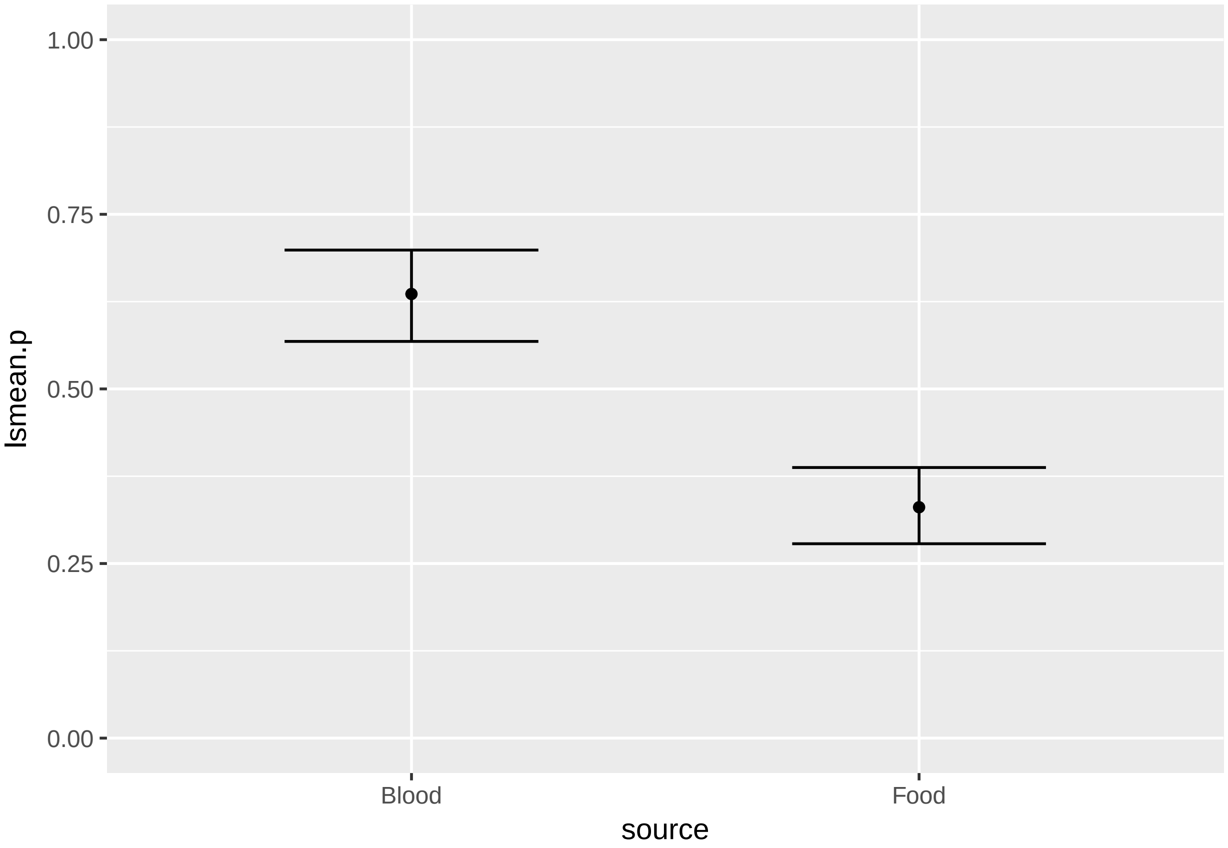

We do see a distinct effect if we look by `source`; food source strains are less likely to be susceptible than blood strains.

Tables of clusters

These include both acids and adapted and non-adapted.

```
acid %>%
  select(adapted.not,cluster) %>%
  table()
```

```
##            cluster
## adapted.not   1   2
##           a 133 108
##           n 132 117
```

```
acid %>%
  select(clonal.complex,cluster) %>%
  table()
```

```
##               cluster
## clonal.complex  1  2
##          CC1   61 36
##          CC121 75 27
##          CC207  0 12
##          CC224 12  0
##          CC31   5  9
##          CC4    0 12
```

```
##           CC415   0  12
##           CC54   12   0
##           CC6    25  59
##           CC9    50  47
##           ST226  12   0
##           ST28    1  11
##           ST739  12   0
```

```
acid %>%
  select(source,cluster) %>%
  table()
```

```
##         cluster
## source     1   2
##   Blood   75 131
##   Food   190  94
```

```
acid %>%
  select(lineage,cluster) %>%
  table()
```

```
##         cluster
## lineage   1   2
##       I  110 107
##      II  155 118
```

```
acid %>%
  select(serotype,cluster) %>%
  table()
```

```
##          cluster
## serotype   1   2
##     1/2a  80  72
##     1/2b  24  12
##     1/2c  50  34
##     3c    13   0
##     4b    98 107
```

```
write.csv(acid,file="acid_with_clusters.csv")
```

BC

David Kent (dk657)

11/26/2017

Contents

Method: . . . 1
Readout: . . . 1
Questions: . . . 1

The data **1**
Huge ugly table . . . 1

Data summaries **5**

Fit the models: **8**
Clonal complex . . . 8
Clonal complex . . . 10
Lineage . . . 11
Source . . . 13

Method:

Inoculate strains on plates with different concentrations of benzalkonium chloride (microgramms/ml)

Readout:

Concentration of BC on the plate with highest BC concentration with confluent growth of bacteria. Three replicates. Definition of the phenotype: 10ug/ml or more => resistant; below =sensitive

Questions:

Is there a correlation between BC resistance and CC? Is there a correlation between BC resistance and source? Is there a correlation between BC resistance and lineage? Is there a correlation between BC resistance and serotype?

The data

Huge ugly table

```
library(car)
```

```
## Warning: package 'car' was built under R version 3.4.3
```

```
library(effects)
```

```
## Loading required package: carData
```

```
##
## Attaching package: 'carData'

## The following objects are masked from 'package:car':
##
##     Guyer, UN, Vocab

## lattice theme set by effectsTheme()
## See ?effectsTheme for details.
```

```
library(MASS)
library(ggplot2)
library(lsmeans)
```

```
## The 'lsmeans' package is being deprecated.
## Users are encouraged to switch to 'emmeans'.
## See help('transition') for more information, including how
## to convert 'lsmeans' objects and scripts to work with 'emmeans'.
```

```
library(knitr)
library(kableExtra)
library(dplyr)
```

```
##
## Attaching package: 'dplyr'

## The following object is masked from 'package:MASS':
##
##     select

## The following object is masked from 'package:car':
##
##     recode

## The following objects are masked from 'package:stats':
##
##     filter, lag

## The following objects are masked from 'package:base':
##
##     intersect, setdiff, setequal, union
```

```
library(tidyr)
bc <- read.csv(file="bc.csv") %>%
  mutate(lineage=as.character(lineage)) %>%
  mutate(lineage=ifelse(clonal.complex=="CC1","I",lineage)) %>%
  as.tbl()

# Make readout an ordered factor
bc$readout <- factor(bc$readout, ordered=T)
bc$lineage <- factor(bc$lineage)

bc %>%
  kable("latex", longtable = T, booktabs = T) %>%
  kable_styling(latex_options = c("repeat_header"), font_size = 7)
```

ID	clonal.complex	serotype	lineage	source	NENT.Nr.	sequencer.ID	replicate	readout
14	CC6	4b	I	Blood	N12-1387	S182	2	0

(continued)

ID	clonal.complex	serotype	lineage	source	NENT.Nr.	sequencer.ID	replicate	readout
14	CC6	4b	I	Blood	N12-1387	S182	3	0
4	CC1	4b	I	Food	N13-0047	S195	1	2.5
5	CC1	4b	I	Blood	N11-2292	S163	1	2.5
7	CC1	4b	I	Blood	N13-0987	21931_2#72	1	2.5
13	CC6	4b	I	Blood	N11-2801	S166	1	2.5
14	CC6	4b	I	Blood	N12-1387	S182	1	2.5
13	CC6	4b	I	Blood	N11-2801	S166	2	2.5
5	CC1	4b	I	Blood	N11-2292	S163	3	2.5
7	CC1	4b	I	Blood	N13-0987	21931_2#72	3	2.5
13	CC6	4b	I	Blood	N11-2801	S166	3	2.5
20	CC9	1/2c	II	Food	N11-1848		3	2.5
22	CC9	1/2a	II	Blood	N11-1837	S82	3	2.5
1	CC1	4b	I	Food	N12-0605	S176	1	5
2	CC1	4b	I	Food	N12-1339	S181	1	5
3	CC1	4b	I	Food	N12-1996	S191	1	5
6	CC1	4b	I	Blood	LL195	LL195_CG	1	5
8	CC1	4b	I	Blood	N13-1079	21931_2#75	1	5
9	CC6	4b	I	Food	N12-0460		1	5
10	CC4	4b	I	Food	N12-1772	S187	1	5
11	CC6	4b	I	Food	N13-0703		1	5
12	CC6	4b	I	Food	N13-1184		1	5
15	CC6	4b	I	Blood	N13-1271	21931_2#78	1	5
16	CC6	4b	I	Blood	N13-1507	21931_2#84	1	5
17	CC9	1/2c	II	Food	N11-1698	21931_2#78	1	5
18	CC9	1/2c	II	Food	N12-0710	S142	1	5
19	CC9	1/2c	II	Food	N12-0822	S143	1	5
20	CC9	1/2c	II	Food	N11-1848		1	5
21	CC9	1/2c	II	Food	N14-0261		1	5
22	CC9	1/2a	II	Blood	N11-1837	S82	1	5
23	CC9	1/2c	II	Blood	N12-0486	S139	1	5
24	CC9	1/2c	II	Blood	N13-0001	S149	1	5
33	ST739	1/2a	II	Food	N11-2542	S30	1	5
35	ST226	1/2a	II	Food	N13-2179	21903_6#61	1	5
36	CC31	1/2a	II	Food	N13-0228	S70	1	5
37	CC207	1/2a	II	Blood	N12-1107	S93	1	5
38	CC415	1/2b	II	Blood	N13-0762	S133	1	5
40	CC224	1/2b	I	Blood	N12-1608	S122	1	5
1	CC1	4b	I	Food	N12-0605	S176	2	5
2	CC1	4b	I	Food	N12-1339	S181	2	5
3	CC1	4b	I	Food	N12-1996	S191	2	5
4	CC1	4b	I	Food	N13-0047	S195	2	5
5	CC1	4b	I	Blood	N11-2292	S163	2	5
6	CC1	4b	I	Blood	LL195	LL195_CG	2	5
7	CC1	4b	I	Blood	N13-0987	21931_2#72	2	5
8	CC1	4b	I	Blood	N13-1079	21931_2#75	2	5
9	CC6	4b	I	Food	N12-0460		2	5
10	CC4	4b	I	Food	N12-1772	S187	2	5
11	CC6	4b	I	Food	N13-0703		2	5
12	CC6	4b	I	Food	N13-1184		2	5
15	CC6	4b	I	Blood	N13-1271	21931_2#78	2	5
16	CC6	4b	I	Blood	N13-1507	21931_2#84	2	5
17	CC9	1/2c	II	Food	N11-1698	21931_2#78	2	5
18	CC9	1/2c	II	Food	N12-0710	S142	2	5
19	CC9	1/2c	II	Food	N12-0822	S143	2	5
20	CC9	1/2c	II	Food	N11-1848		2	5
21	CC9	1/2c	II	Food	N14-0261		2	5
22	CC9	1/2a	II	Blood	N11-1837	S82	2	5
23	CC9	1/2c	II	Blood	N12-0486	S139	2	5
24	CC9	1/2c	II	Blood	N13-0001	S149	2	5
33	ST739	1/2a	II	Food	N11-2542	S30	2	5
35	ST226	1/2a	II	Food	N13-2179	21903_6#61	2	5
36	CC31	1/2a	II	Food	N13-0228	S70	2	5

(continued)

ID	clonal.complex	serotype	lineage	source	NENT.Nr.	sequencer.ID	replicate	readout
37	CC207	1/2a	II	Blood	N12-1107	S93	2	5
38	CC415	1/2b	II	Blood	N13-0762	S133	2	5
39	CC54	4b	I	Blood	N13-0177	S196	2	5
40	CC224	1/2b	I	Blood	N12-1608	S122	2	5
1	CC1	4b	I	Food	N12-0605	S176	3	5
2	CC1	4b	I	Food	N12-1339	S181	3	5
3	CC1	4b	I	Food	N12-1996	S191	3	5
4	CC1	4b	I	Food	N13-0047	S195	3	5
6	CC1	4b	I	Blood	LL195	LL195_CG	3	5
8	CC1	4b	I	Blood	N13-1079	21931_2#75	3	5
9	CC6	4b	I	Food	N12-0460		3	5
10	CC4	4b	I	Food	N12-1772	S187	3	5
11	CC6	4b	I	Food	N13-0703		3	5
12	CC6	4b	I	Food	N13-1184		3	5
15	CC6	4b	I	Blood	N13-1271	21931_2#78	3	5
16	CC6	4b	I	Blood	N13-1507	21931_2#84	3	5
17	CC9	1/2c	II	Food	N11-1698	21931_2#78	3	5
18	CC9	1/2c	II	Food	N12-0710	S142	3	5
19	CC9	1/2c	II	Food	N12-0822	S143	3	5
21	CC9	1/2c	II	Food	N14-0261		3	5
23	CC9	1/2c	II	Blood	N12-0486	S139	3	5
24	CC9	1/2c	II	Blood	N13-0001	S149	3	5
33	ST739	1/2a	II	Food	N11-2542	S30	3	5
35	ST226	1/2a	II	Food	N13-2179	21903_6#61	3	5
36	CC31	1/2a	II	Food	N13-0228	S70	3	5
37	CC207	1/2a	II	Blood	N12-1107	S93	3	5
38	CC415	1/2b	II	Blood	N13-0762	S133	3	5
39	CC54	4b	I	Blood	N13-0177	S196	3	5
40	CC224	1/2b	I	Blood	N12-1608	S122	3	5
28	CC121	1/2b	II	Food	N13-0836		1	7.5
39	CC54	4b	I	Blood	N13-0177	S196	1	7.5
34	CC121	1/2a	II	Food	N13-0288	S74	3	7.5
25	CC121	1/2a	II	Food	N11-1218	S10	2	10
26	CC121	1/2a	II	Food	N12-0571	S44	2	10
27	CC121	1/2a	II	Food	N13-0369	S75	2	10
28	CC121	1/2b	II	Food	N13-0836		2	10
29	CC121	1/2a	II	Food	N14-0205	21903_6#63	2	10
30	CC121	3c	II	Food	N14-0322	21903_6#64	2	10
32	CC121	1/2a	II	Blood	N13-0119	S102	2	10
34	CC121	1/2a	II	Food	N13-0288	S74	2	10
25	CC121	1/2a	II	Food	N11-1218	S10	3	10
28	CC121	1/2b	II	Food	N13-0836		3	10
25	CC121	1/2a	II	Food	N11-1218	S10	1	15
26	CC121	1/2a	II	Food	N12-0571	S44	1	15
27	CC121	1/2a	II	Food	N13-0369	S75	1	15
29	CC121	1/2a	II	Food	N14-0205	21903_6#63	1	15
30	CC121	3c	II	Food	N14-0322	21903_6#64	1	15
31	CC121	1/2a	II	Blood	N12-0367	S109	1	15
32	CC121	1/2a	II	Blood	N13-0119	S102	1	15
34	CC121	1/2a	II	Food	N13-0288	S74	1	15
31	CC121	1/2a	II	Blood	N12-0367	S109	2	15
26	CC121	1/2a	II	Food	N12-0571	S44	3	15
27	CC121	1/2a	II	Food	N13-0369	S75	3	15
29	CC121	1/2a	II	Food	N14-0205	21903_6#63	3	15
30	CC121	3c	II	Food	N14-0322	21903_6#64	3	15
31	CC121	1/2a	II	Blood	N12-0367	S109	3	15
32	CC121	1/2a	II	Blood	N13-0119	S102	3	15

Data summaries

This is a peculiar situation. Since the "readout" values are predetermined to be 0, 2.5, etc., a simple linear model will not work; the residuals are not going to be normal. We could do a logistic regression on readout >= 10, but then we lose a lot of information – that readout = 7.5 is greater than readout = 5, for example.

I think the most appropriate attack would be an ordinal regression. This takes the six categories in order (0 < 2.5 < 5 < 7.5 < 10 < 15) and fits an effect for the probability (actually log odds I think) of a subject to be in the next higher category.

I believe that we could calculate the predicted probability that a group will be in the resistant phenotype (i.e. >= 10) from these models.

First let's check out plots of the raw data. This is the proportion of observations in a category which fall into a readout. They're sort of "upside-down" in the sense that 0 is at the top of the bar, 15 is at the bottom:

```
bc %>%
  group_by(readout,lineage) %>%
  summarize(freq=n()) %>%
  ungroup() %>%
  ggplot(aes(x=lineage,y=freq,fill=readout)) +
  geom_bar(stat="identity",position=position_fill(),color="black")
```

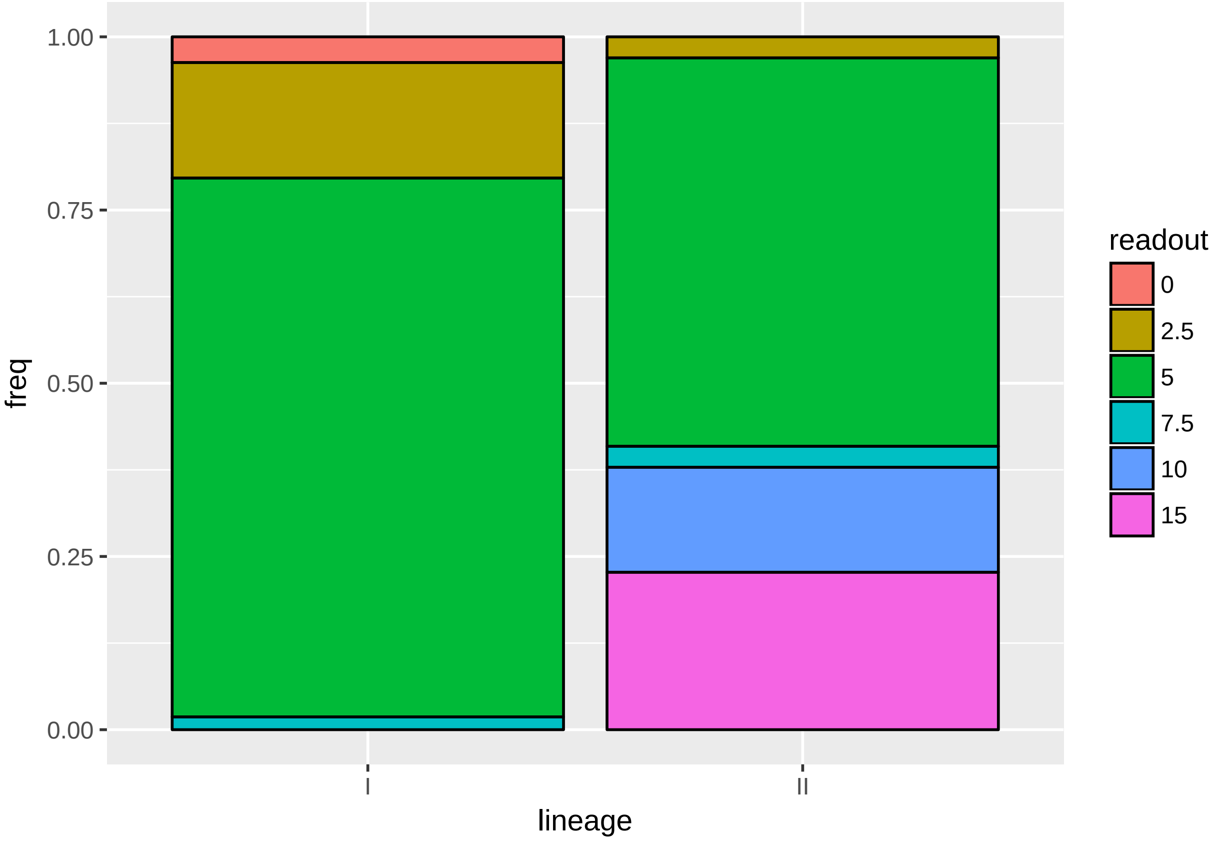


```
bc %>%
  group_by(readout,clonal.complex) %>%
  summarize(freq=n()) %>%
  ungroup() %>%
  ggplot(aes(x=clonal.complex,y=freq,fill=readout)) +
  geom_bar(stat="identity",position=position_fill(),color="black")
```

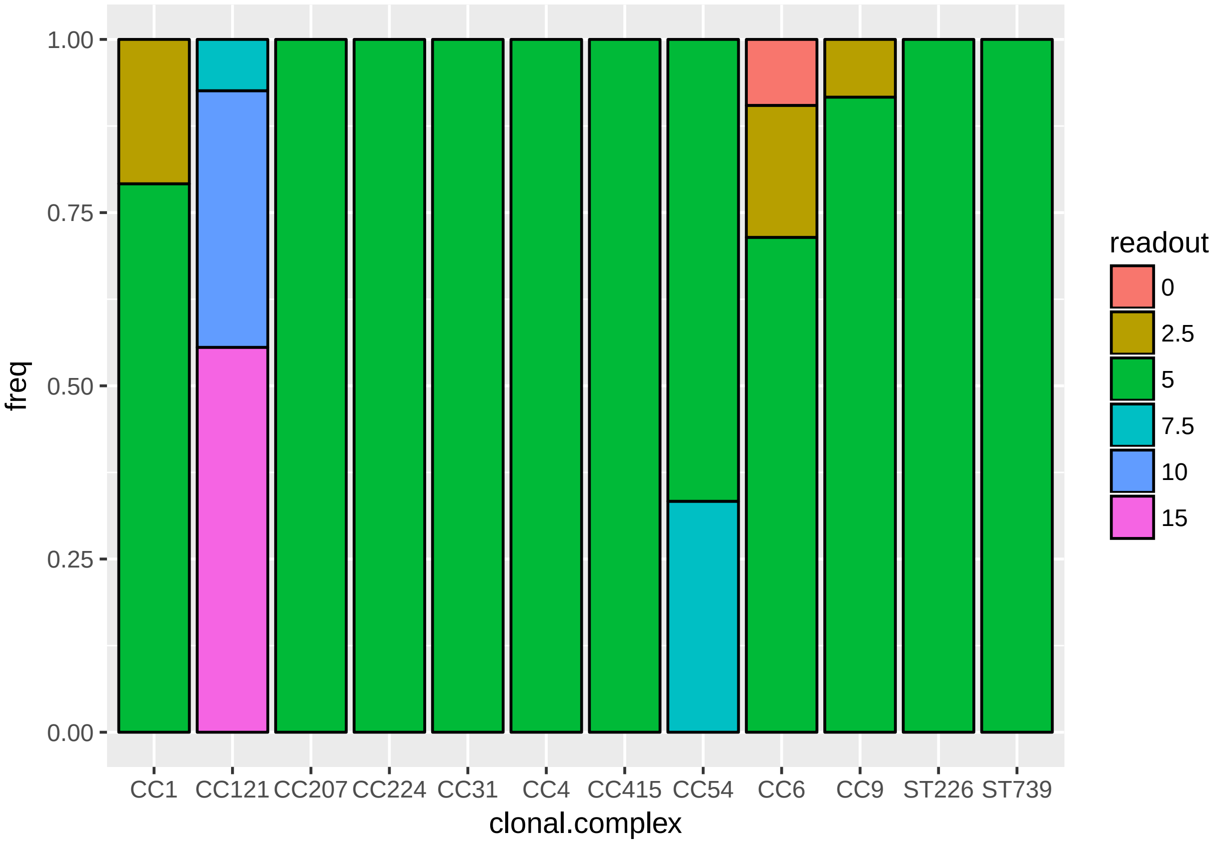


```
bc %>%
  group_by(readout,serotype) %>%
  summarize(freq=n()) %>%
  ungroup() %>%
  ggplot(aes(x=serotype,y=freq,fill=readout)) +
  geom_bar(stat="identity",position=position_fill(),color="black")
```

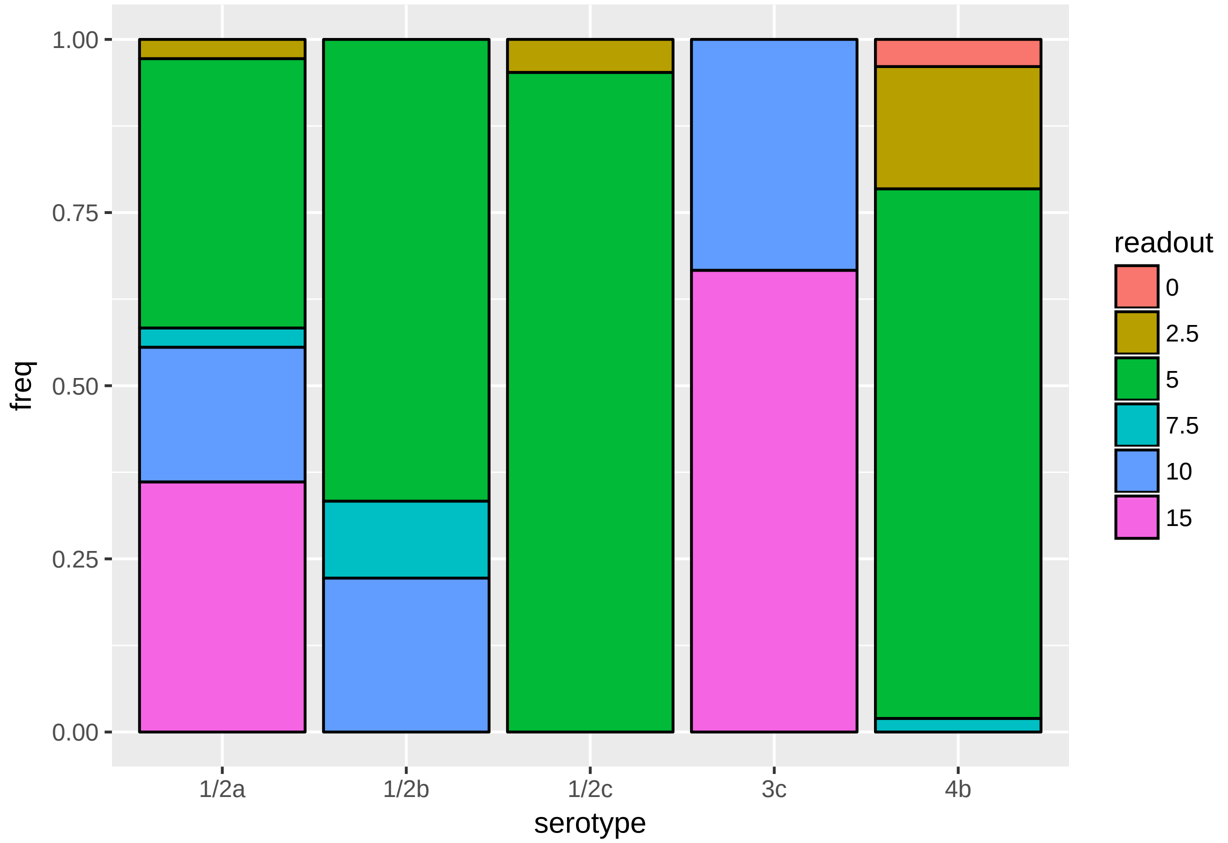


```
bc %>%
  group_by(readout,source) %>%
  summarize(freq=n()) %>%
  ungroup() %>%
  ggplot(aes(x=source,y=freq,fill=readout)) +
  geom_bar(stat="identity",position=position_fill(),color="black")
```

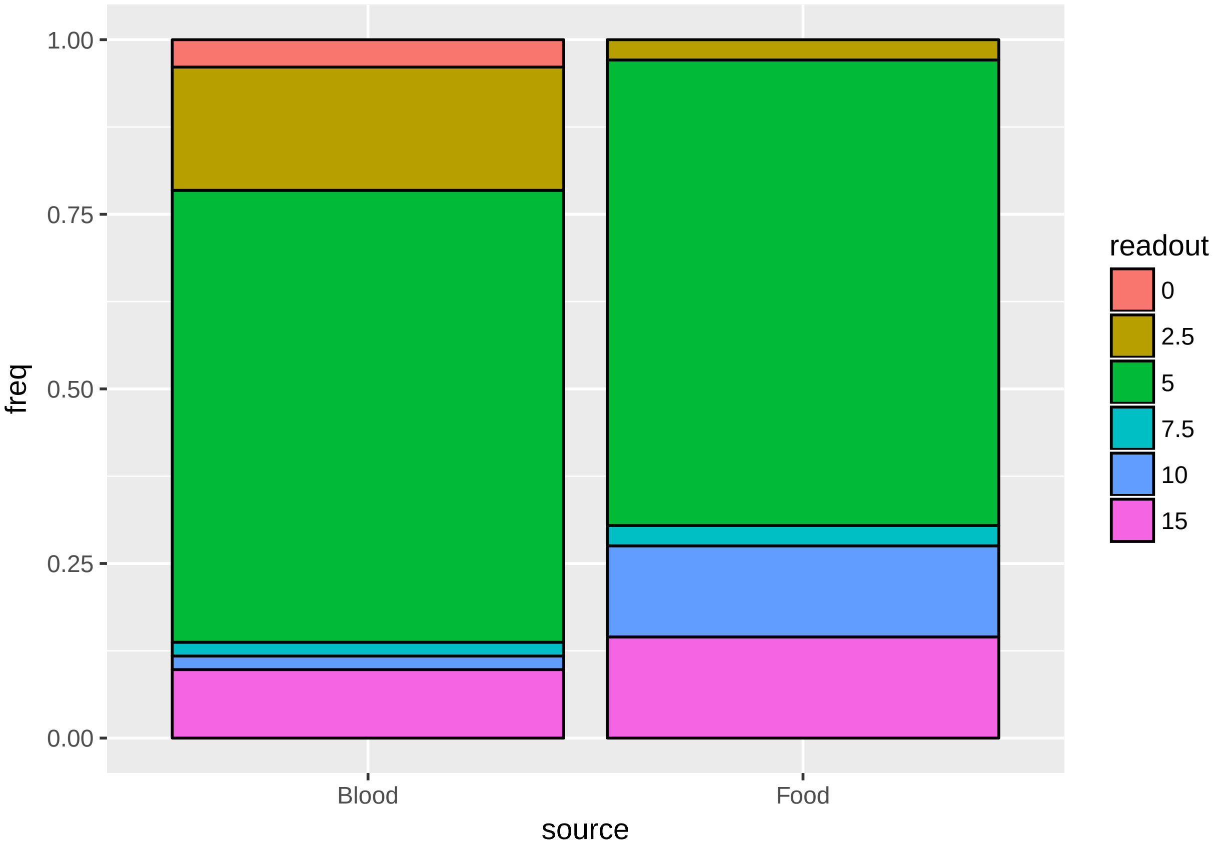

It does appear that lineage II is more resistant than lineage I, CC121 is more resistant than all the others, and meat source is more resistant than blood source, which is more resistant than milk source.

Fit the models:

Clonal complex

```
m_cc <- polr( readout ~ clonal.complex , data=bc)
Anova(m_cc)
```

```
## Analysis of Deviance Table (Type II tests)
##
## Response: readout
##                  LR Chisq Df Pr(>Chisq)
## clonal.complex   143.19 11  < 2.2e-16 ***
## ---
## Signif. codes:  0 '***' 0.001 '**' 0.01 '*' 0.05 '.' 0.1 ' ' 1
```

```
summary(m_cc) %>%
  coef() %>%
  data.frame() %>%
  tibble::rownames_to_column() %>%
  mutate(p=pnorm(abs(t.value),lower.tail=F)*2) %>%
  kable()
```

```
##
```

```
## Re-fitting to get Hessian
```

rowname	Value	Std..Error	t.value	p
clonal.complexCC121	35.0421970	9.0582473	3.8685405	0.0001095
clonal.complexCC207	9.8795165	107.4981562	0.0919041	0.9267743
clonal.complexCC224	9.8795165	107.4981562	0.0919041	0.9267743
clonal.complexCC31	9.8795165	107.4981562	0.0919041	0.9267743
clonal.complexCC4	9.8795165	107.4981562	0.0919041	0.9267743
clonal.complexCC415	9.8795165	107.4981562	0.0919041	0.9267743
clonal.complexCC54	20.5308059	48.8628626	0.4201720	0.6743598
clonal.complexCC6	-0.5264822	0.6952737	-0.7572302	0.4489119
clonal.complexCC9	1.0364598	0.8909405	1.1633322	0.2446948
clonal.complexST226	9.8795165	107.4981562	0.0919041	0.9267743
clonal.complexST739	9.8795165	107.4981562	0.0919041	0.9267743
0|2.5	-3.4763543	0.8229846	-4.2240819	0.0000240
2.5|5	-1.3733532	0.4996348	-2.7487137	0.0059830
5|7.5	21.2241897	48.8526081	0.4344536	0.6639591
7.5|10	32.5165270	9.0858762	3.5787993	0.0003452
10|15	34.8190756	9.0655908	3.8407950	0.0001226

The ANOVA table here tells us that `clonal.complex` does have a significant effect on BC resistance.

The table below that tells us that CC121 is significantly more likely to be more highly resistant than the reference CC, CC1, just as you suspected. You can ignore then rows that say `0|2.5`or similar; they are intercepts.

I'm not sure how to generate pairwise comparisons in this context, but if you need them, let me know and I can try.

```
plot(Effect(mod=m_cc,focal.predictors = "clonal.complex"),style="stacked")
```

```
##
## Re-fitting to get Hessian
```

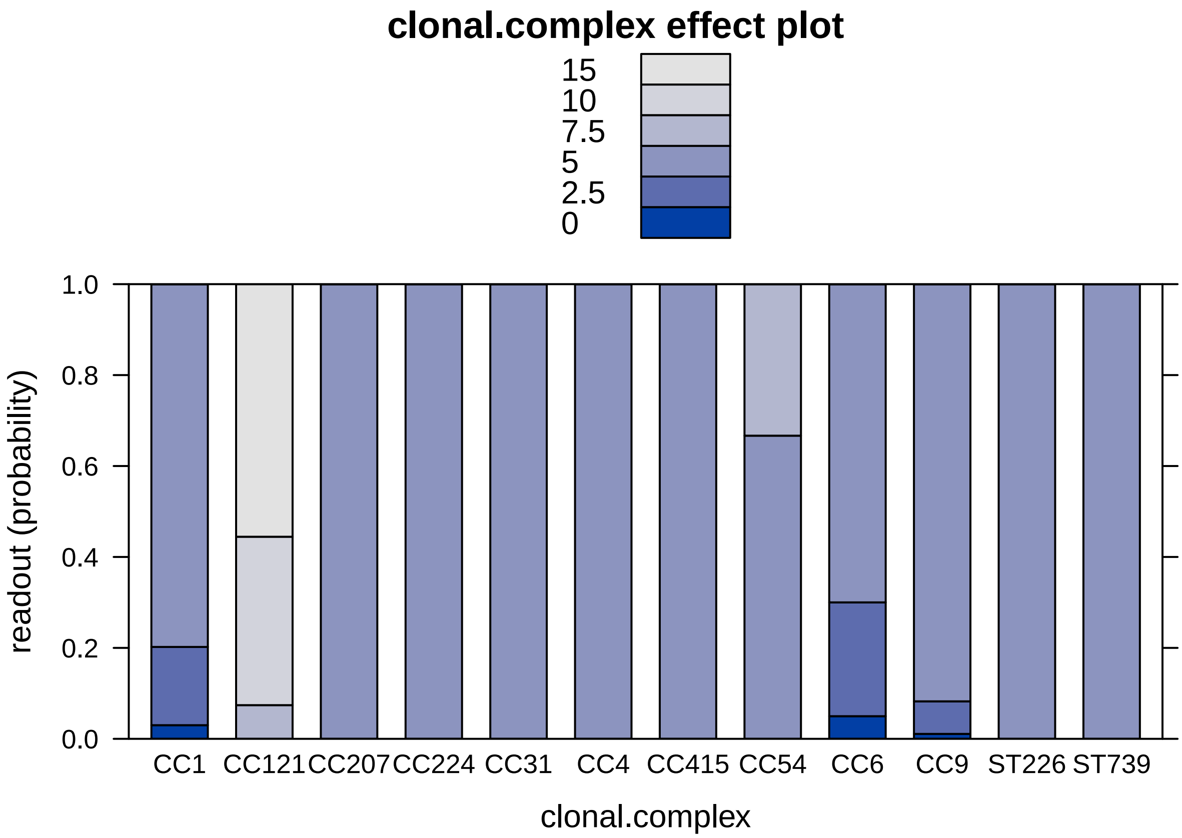

This is the fitted effects from the model.

Clonal complex

```
m_serotype <- polr( readout ~ serotype , data=bc)
Anova(m_serotype)
```

```
## Analysis of Deviance Table (Type II tests)
##
## Response: readout
##          LR Chisq Df Pr(>Chisq)
## serotype    64.32  4  3.579e-13 ***
## ---
## Signif. codes:  0 '***' 0.001 '**' 0.01 '*' 0.05 '.' 0.1 ' ' 1
```

```
summary(m_serotype) %>%
  coef() %>%
  data.frame() %>%
  tibble::rownames_to_column() %>%
  mutate(p=pnorm(abs(t.value),lower.tail=F)*2) %>%
  kable()
```

```
##
## Re-fitting to get Hessian
```

rowname	Value	Std..Error	t.value	p
serotype1/2b	-1.3176940	0.7410764	-1.7780812	0.0753905

rowname	Value	Std..Error	t.value	p
serotype1/2c	-3.5452259	0.8867488	-3.9980047	0.0000639
serotype3c	1.6533117	1.2169425	1.3585783	0.1742803
serotype4b	-4.5732967	0.8697539	-5.2581503	0.0000001
0|2.5	-7.9275225	1.0799651	-7.3405357	0.0000000
2.5|5	-5.8721724	0.8547343	-6.8701727	0.0000000
5|7.5	-0.4657314	0.3504167	-1.3290790	0.1838219
7.5|10	-0.2076983	0.3455925	-0.6009921	0.5478453
10|15	0.7503666	0.3563618	2.1056313	0.0352364

The ANOVA table here tells us that `serotype` does have a significant effect on BC resistance.

Serotype 1/2b, 1/2c, and 4b are all less likely to be highly resistant than 1/2a. Serotype 3c is more likely to be highly resistant than 1/2a. Of those, only the 1/2c vs. 1/2a and 4b vs. 1/2a comparisons are significant.

```
plot(Effect(mod=m_serotype,focal.predictors = "serotype"),style="stacked")
```

```
##
## Re-fitting to get Hessian
```

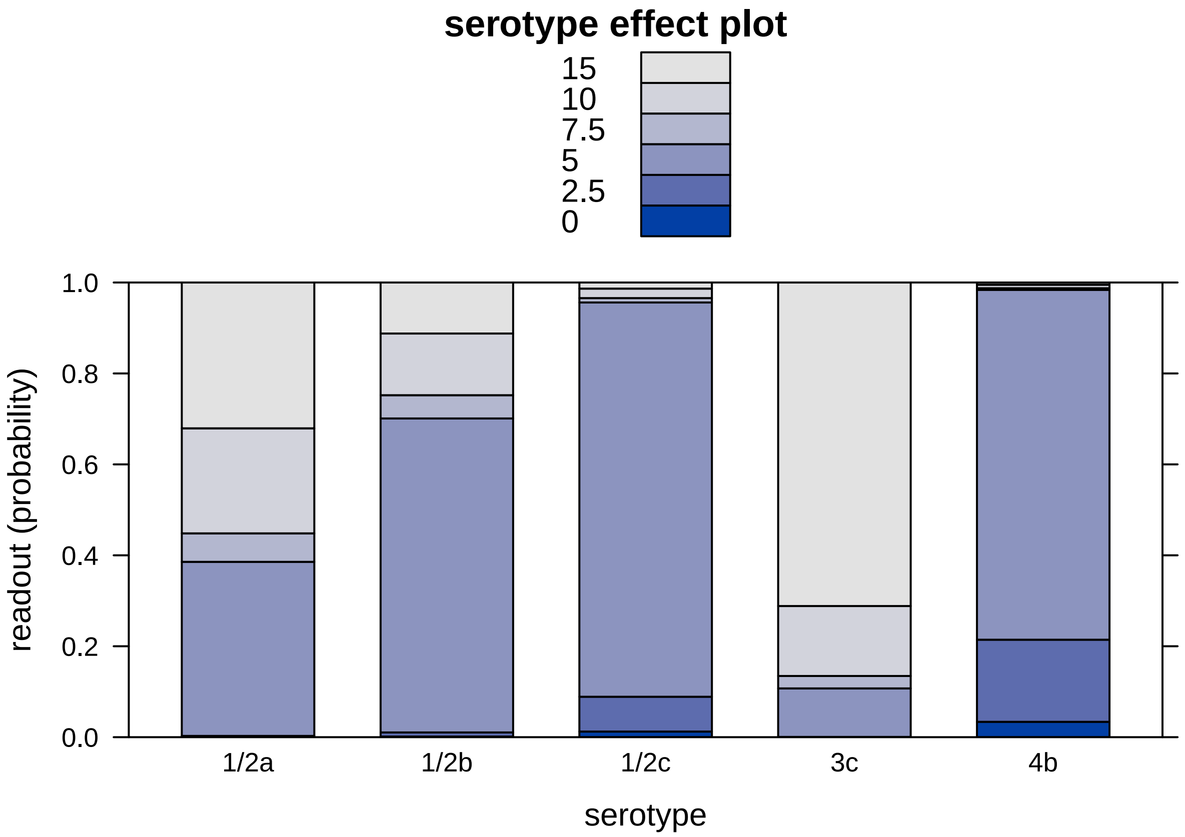

This is the fitted effects from the model.

Lineage

```
m_l <- polr( readout ~ lineage , data=bc)
Anova(m_l)
```

```
## Analysis of Deviance Table (Type II tests)
##
## Response: readout
##         LR Chisq Df Pr(>Chisq)
## lineage   35.619  1    2.4e-09 ***
## ---
## Signif. codes:  0 '***' 0.001 '**' 0.01 '*' 0.05 '.' 0.1 ' ' 1
```

```r
summary(m_l) %>%
  coef() %>%
  data.frame() %>%
  tibble::rownames_to_column() %>%
  mutate(p=pnorm(abs(t.value),lower.tail=F)*2) %>%
  kable()
```

```
##
## Re-fitting to get Hessian
```

rowname	Value	Std..Error	t.value	p
lineageII	2.907070	0.6370981	4.562986	5.00e-06
0|2.5	-3.325194	0.7215886	-4.608157	4.10e-06
2.5|5	-1.269114	0.3199027	-3.967186	7.27e-05
5|7.5	3.290908	0.6047171	5.442063	1.00e-07
7.5|10	3.471276	0.6118900	5.673039	0.00e+00
10|15	4.187147	0.6432987	6.508869	0.00e+00

Similarly, lineage also is significantly associated with BC resistance. Lineage II is more likely to be more highly resistant than lineage I.

```r
plot(Effect(mod=m_l,focal.predictors = "lineage"),style="stacked")
```

```
##
## Re-fitting to get Hessian
```

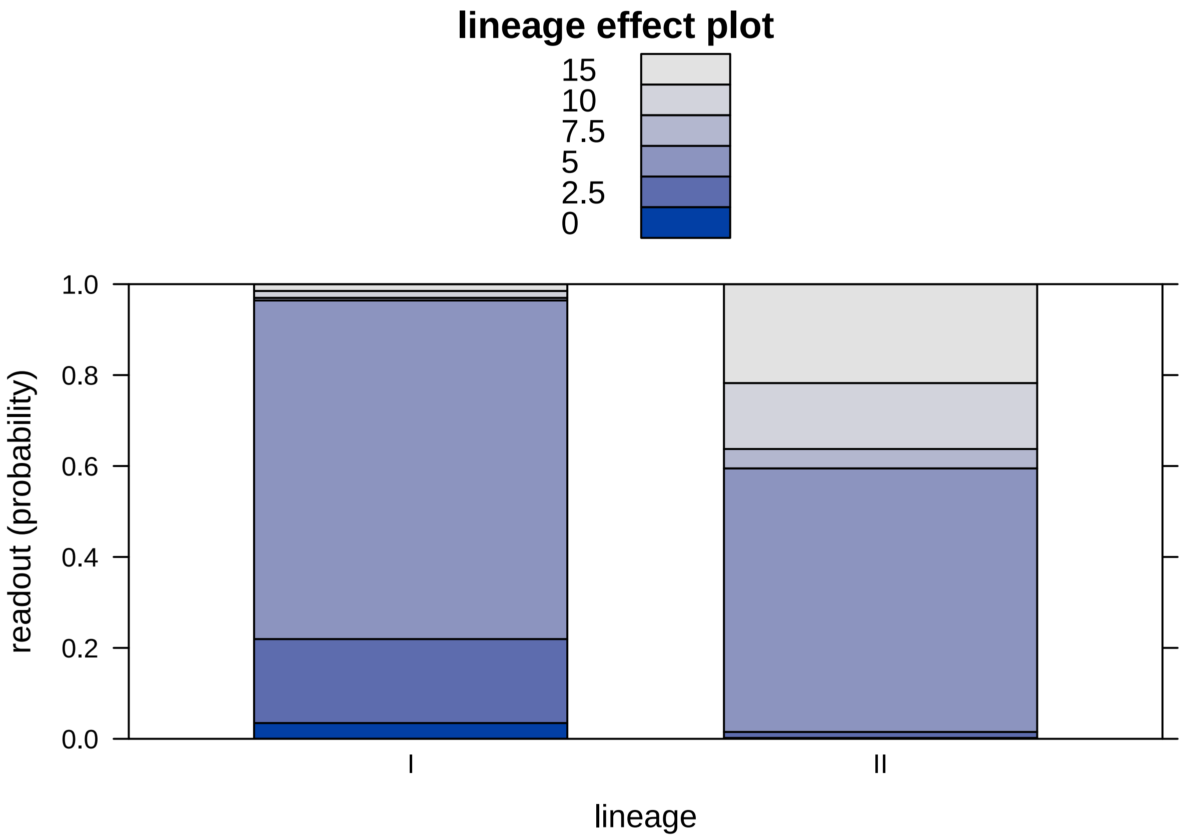

Source

```
m_source <- polr( readout ~ source , data=bc)
Anova(m_source)
```

```
## Analysis of Deviance Table (Type II tests)
##
## Response: readout
##        LR Chisq Df Pr(>Chisq)
## source   10.907  1   0.000958 ***
## ---
## Signif. codes:  0 '***' 0.001 '**' 0.01 '*' 0.05 '.' 0.1 ' ' 1
```

```
summary(m_source) %>%
  coef() %>%
  data.frame() %>%
  tibble::rownames_to_column() %>%
  mutate(p=pnorm(abs(t.value),lower.tail=F)*2) %>%
  kable()
```

```
##
## Re-fitting to get Hessian
```

rowname	Value	Std..Error	t.value	p
sourceFood	1.358868	0.4358211	3.117950	0.0018211
0|2.5	-3.507512	0.7289387	-4.811807	0.0000015

rowname	Value	Std..Error	t.value	p
2.5|5	-1.478288	0.3403126	-4.343912	0.0000140
5|7.5	2.126179	0.3970922	5.354371	0.0000001
7.5|10	2.279924	0.4041341	5.641502	0.0000000
10|15	2.910957	0.4397536	6.619519	0.0000000

Source is significantly associated with BC resistance. `Meat` is more likely to be more highly resistant than `Blood`.

```
plot(Effect(mod=m_source,focal.predictors = "source"),style="stacked")
```

```
##
## Re-fitting to get Hessian
```

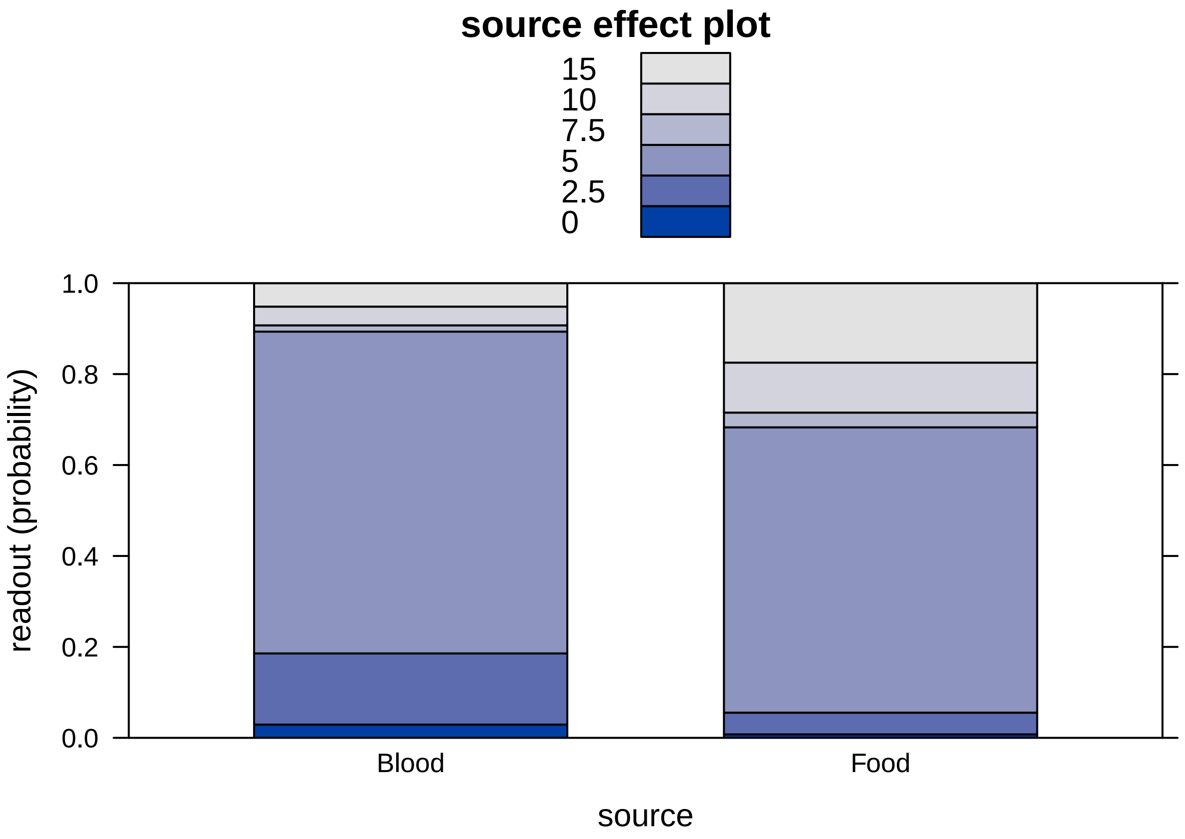

Hemolysis

David Kent (dk657)

11/25/2017

Contents

Method: 1

Readout: 1

Question: 2

The data 2
- Huge ugly table 2

Raw plots 5

Fit the models: 9
- Clonal Complex 9
  - Plot 12
- Serotype 13
  - Plot 16
- Lineage 17
  - Plot 18
- Source 19
  - Plot 21

Method:

Measure how much the supernatant of the strain lyses human blood. This is (mostly) a function of how much LLO (gene = hly) is secreted by the strain.

Three replicates. Fresh blood for each replicate (different donors, same blood type). Each biological replicate consists of 3 technical replicates (e.g. 3 wells with the same strain per run, which are reflected in the SD that is given for each replicate).

Readout:

These are OD measurements. Mix blood with supernatant, incubate. Spin down intact erythrocytes, measure the OD of the supernatant. The more erythrocytes were lysed, the more free hemoglobin is in the supernatant -> higher OD.

To account for possible differences in the blood, we included a highly hemolytic strain Prfa* (constitutively makes high amounts of LLO). We can either work with the absolute OD's or with the values relative to PrfA, which are %.

Question:

Is there a correlation between hemolytic potential and CC? Is there a correlation between hemolytic potential and source? Is there a correlation between hemolytic potential and lineage? Is there a correlation between hemolytic potential and serotype?

The data

Huge ugly table

```r
library(multcompView)
library(lmerTest)
```

```
## Warning: package 'lmerTest' was built under R version 3.4.4
## Loading required package: lme4
## Warning: package 'lme4' was built under R version 3.4.4
## Loading required package: Matrix
##
## Attaching package: 'lmerTest'
## The following object is masked from 'package:lme4':
##
##     lmer
## The following object is masked from 'package:stats':
##
##     step
```

```r
library(ggplot2)
library(lsmeans)
```

```
## Warning: package 'lsmeans' was built under R version 3.4.4
## The 'lsmeans' package is being deprecated.
## Users are encouraged to switch to 'emmeans'.
## See help('transition') for more information, including how
## to convert 'lsmeans' objects and scripts to work with 'emmeans'.
```

```r
library(knitr)
```

```
## Warning: package 'knitr' was built under R version 3.4.3
```

```r
library(kableExtra)
```

```
## Warning: package 'kableExtra' was built under R version 3.4.4
```

```r
library(dplyr)
```

```
## Warning: package 'dplyr' was built under R version 3.4.4
##
## Attaching package: 'dplyr'
## The following objects are masked from 'package:stats':
##
```

```
##     filter, lag
## The following objects are masked from 'package:base':
##
##     intersect, setdiff, setequal, union
```

```r
library(tidyr)
```

```
## Warning: package 'tidyr' was built under R version 3.4.4
##
## Attaching package: 'tidyr'
## The following object is masked from 'package:Matrix':
##
##     expand
```

```r
hemo <- read.csv(file="hemolysis.csv") %>%
  mutate(lineage=as.character(lineage)) %>%
  mutate(lineage=ifelse(clonal.complex=="CC1","I",lineage)) %>%
  as.tbl()
```

```
## Warning: package 'bindrcpp' was built under R version 3.4.4
```

```r
hemo %>%
  kable("latex", longtable = T, booktabs = T) %>%
  kable_styling(latex_options = c("repeat_header"), font_size = 5)
```

ID	clonal.complex	serotype	lineage	Date	source	NENT.Nr.	sequencer.ID	X	Mean.OD	SD.OD	relative.to.PrfA.
1	CC1	4b	I	03.02.17	Food	N12-0605	S176	1	0.654	0.167	81.784
1	CC1	4b	I	03.10.17	Food	N12-0605	S176	2	2.212	0.167	89.627
1	CC1	4b	I	3/31/2017	Food	N12-0605	S176	3	1.131	0.074	91.800
2	CC1	4b	I	03.02.17	Food	N12-1339	S181	1	0.676	0.066	84.577
2	CC1	4b	I	03.10.17	Food	N12-1339	S181	2	2.156	0.205	87.345
2	CC1	4b	I	3/31/2017	Food	N12-1339	S181	3	1.223	0.094	99.269
3	CC1	4b	I	03.02.17	Food	N12-1996	S191	1	0.632	0.106	79.033
3	CC1	4b	I	03.10.17	Food	N12-1996	S191	2	2.191	0.134	88.790
3	CC1	4b	I	3/31/2017	Food	N12-1996	S191	3	1.324	0.004	107.470
4	CC1	4b	I	03.02.17	Food	N13-0047	S195	1	0.627	0.026	78.408
4	CC1	4b	I	03.10.17	Food	N13-0047	S195	2	2.415	0.067	97.853
4	CC1	4b	I	3/31/2017	Food	N13-0047	S195	3	1.417	0.145	115.074
5	CC1	4b	I	03.02.17	Blood	N11-2292	S163	1	0.570	0.024	71.238
5	CC1	4b	I	3/16/2017	Blood	N11-2292	S163	2	1.158	0.081	69.823
5	CC1	4b	I	3/31/2017	Blood	N11-2292	S163	3	1.215	0.059	98.674
6	CC1	4b	I	03.02.17	Blood	LL195	LL195_CG	1	0.503	0.099	62.860
6	CC1	4b	I	3/16/2017	Blood	LL195	LL195_CG	2	1.316	0.166	79.353
6	CC1	4b	I	3/31/2017	Blood	LL195	LL195_CG	3	1.277	0.058	103.708
7	CC1	4b	I	03.02.17	Blood	N13-0987	21931_2#72	1	0.489	0.071	61.150
7	CC1	4b	I	3/16/2017	Blood	N13-0987	21931_2#72	2	1.523	0.111	91.858
7	CC1	4b	I	3/31/2017	Blood	N13-0987	21931_2#72	3	1.243	0.046	100.920
8	CC1	4b	I	3/16/2017	Blood	N13-1079	21931_2#75	1	1.463	0.095	94.549
8	CC1	4b	I	3/31/2017	Blood	N13-1079	21931_2#75	2	1.921	0.106	115.862
8	CC1	4b	I	04.12.17	Blood	N13-1079	21931_2#75	3	1.195	0.033	97.050
9	CC6	4b	I	03.02.17	Food	N12-0460		1	0.413	0.040	31.769
9	CC6	4b	I	3/16/2017	Food	N12-0460		2	1.531	0.080	92.360
9	CC6	4b	I	3/31/2017	Food	N12-0460		3	1.882	0.077	99.331
10	CC4	4b	I	03.02.17	Food	N12-1772	S187	1	0.472	0.023	36.282
10	CC4	4b	I	3/16/2017	Food	N12-1772	S187	2	1.081	0.467	65.179
10	CC4	4b	I	3/31/2017	Food	N12-1772	S187	3	1.958	0.045	103.360
11	CC6	4b	I	03.02.17	Food	N13-0703		1	0.477	0.009	36.692
11	CC6	4b	I	3/16/2017	Food	N13-0703		2	1.808	0.231	77.002
11	CC6	4b	I	3/31/2017	Food	N13-0703		3	1.960	0.053	103.448
12	CC6	4b	I	03.02.17	Food	N13-1184		1	0.462	0.063	35.564
12	CC6	4b	I	3/16/2017	Food	N13-1184		2	1.767	0.050	75.241
12	CC6	4b	I	3/31/2017	Food	N13-1184		3	1.886	0.094	99.560
13	CC6	4b	I	03.02.17	Blood	N11-2801	S166	1	0.215	0.138	16.564
13	CC6	4b	I	3/16/2017	Blood	N11-2801	S166	2	1.376	0.092	58.603
13	CC6	4b	I	3/31/2017	Blood	N11-2801	S166	3	1.528	0.077	80.630
14	CC6	4b	I	04.12.17	Blood	N12-1387	S182	1	0.920	0.063	59.448
14	CC6	4b	I	04.12.17	Blood	N12-1387	S182	2	1.021	0.062	65.999
14	CC6	4b	I	04.12.17	Blood	N12-1387	S182	3	0.985	0.085	63.672

(continued)

ID	clonal.complex	serotype	lineage	Date	source	NENT.Nr.	sequencer.ID	X	Mean.OD	SD.OD	relative.to.PrfA.
15	CC6	4b	I	03.02.17	Blood	N13-1271	21931_2#78	1	0.363	0.211	27.897
15	CC6	4b	I	3/16/2017	Blood	N13-1271	21931_2#78	2	2.123	0.150	90.403
15	CC6	4b	I	3/31/2017	Blood	N13-1271	21931_2#78	3	1.918	0.058	101.249
16	CC6	4b	I	03.02.17	Blood	N13-1507	21931_2#84	1	0.256	0.231	19.692
16	CC6	4b	I	3/16/2017	Blood	N13-1507	21931_2#84	2	2.039	0.021	86.854
16	CC6	4b	I	3/31/2017	Blood	N13-1507	21931_2#84	3	1.849	0.064	97.590
17	CC9	1/2c	II	03.07.17	Food	N11-1698	21931_2#78	1	1.020	0.097	91.482
17	CC9	1/2c	II	3/17/2017	Food	N11-1698	21931_2#78	2	1.465	0.020	96.806
17	CC9	1/2c	II	04.07.17	Food	N11-1698	21931_2#78	3	1.070	0.584	70.342
18	CC9	1/2c	II	03.07.17	Food	N12-0710	S142	1	0.741	0.107	66.467
18	CC9	1/2c	II	3/17/2017	Food	N12-0710	S142	2	1.311	0.048	86.608
18	CC9	1/2c	II	04.07.17	Food	N12-0710	S142	3	1.268	0.016	83.384
19	CC9	1/2c	II	03.07.17	Food	N12-0822	S143	1	0.999	0.091	89.600
19	CC9	1/2c	II	3/17/2017	Food	N12-0822	S143	2	1.472	0.059	97.269
19	CC9	1/2c	II	04.07.17	Food	N12-0822	S143	3	1.279	0.190	84.086
20	CC9	1/2c	II	03.07.17	Food	N11-1848		1	0.984	0.109	88.225
20	CC9	1/2c	II	3/17/2017	Food	N11-1848		2	1.326	0.185	87.621
20	CC9	1/2c	II	04.07.17	Food	N11-1848		3	1.119	0.465	73.564
21	CC9	1/2c	II	03.07.17	Food	N14-0261		1	0.880	0.202	78.870
21	CC9	1/2c	II	3/17/2017	Food	N14-0261		2	1.335	0.035	88.238
21	CC9	1/2c	II	04.07.17	Food	N14-0261		3	1.244	0.083	81.806
22	CC9	1/2a	II	03.07.17	Blood	N11-1837	S82	1	0.636	0.189	57.023
22	CC9	1/2a	II	3/17/2017	Blood	N11-1837	S82	2	1.289	0.054	85.154
22	CC9	1/2a	II	04.12.17	Blood	N11-1837	S82	3	1.367	0.063	88.386
23	CC9	1/2c	II	03.10.17	Blood	N12-0486	S139	1	1.835	0.151	84.420
23	CC9	1/2c	II	3/17/2017	Blood	N12-0486	S139	2	1.885	0.081	89.490
23	CC9	1/2c	II	04.07.17	Blood	N12-0486	S139	3	1.063	0.255	69.904
24	CC9	1/2c	II	03.10.17	Blood	N13-0001	S149	1	1.849	0.129	85.079
24	CC9	1/2c	II	3/17/2017	Blood	N13-0001	S149	2	1.832	0.158	86.990
24	CC9	1/2c	II	04.07.17	Blood	N13-0001	S149	3	1.293	0.009	85.050
25	CC121	1/2a	II	03.10.17	Food	N11-1218	S10	1	1.868	0.134	85.938
25	CC121	1/2a	II	3/17/2017	Food	N11-1218	S10	2	1.845	0.159	87.623
25	CC121	1/2a	II	04.07.17	Food	N11-1218	S10	3	1.558	0.239	82.018
26	CC121	1/2a	II	03.10.17	Food	N12-0571	S44	1	1.725	0.112	79.359
26	CC121	1/2a	II	3/17/2017	Food	N12-0571	S44	2	1.723	0.157	81.830
26	CC121	1/2a	II	04.07.17	Food	N12-0571	S44	3	1.439	0.306	75.754
27	CC121	1/2a	II	03.10.17	Food	N13-0369	S75	1	1.507	0.552	69.307
27	CC121	1/2a	II	3/17/2017	Food	N13-0369	S75	2	2.027	0.119	96.233
27	CC121	1/2a	II	04.07.17	Food	N13-0369	S75	3	2.026	0.190	106.632
28	CC121	1/2b	II	03.10.17	Food	N13-0836		1	1.528	0.258	70.281
28	CC121	1/2b	II	3/17/2017	Food	N13-0836		2	1.647	0.288	78.189
28	CC121	1/2b	II	04.07.17	Food	N13-0836		3	1.935	0.259	101.842
29	CC121	1/2a	II	03.08.17	Food	N14-0205	21903_6#63	1	1.378	0.120	87.622
29	CC121	1/2a	II	3/30/2017	Food	N14-0205	21903_6#63	2	1.323	0.052	109.066
29	CC121	1/2a	II	04.07.17	Food	N14-0205	21903_6#63	3	1.895	0.499	99.754
30	CC121	3c	II	03.08.17	Food	N14-0322	21903_6#64	1	1.276	0.126	81.136
30	CC121	3c	II	3/30/2017	Food	N14-0322	21903_6#64	2	1.292	0.014	106.511
30	CC121	3c	II	04.07.17	Food	N14-0322	21903_6#64	3	1.667	0.618	87.719
31	CC121	1/2a	II	03.08.17	Blood	N12-0367	S109	1	1.329	0.098	84.527
31	CC121	1/2a	II	3/30/2017	Blood	N12-0367	S109	2	1.244	0.027	102.555
31	CC121	1/2a	II	04.07.17	Blood	N12-0367	S109	3	1.408	0.671	74.105
32	CC121	1/2a	II	03.08.17	Blood	N13-0119	S102	1	1.069	0.272	67.953
32	CC121	1/2a	II	3/30/2017	Blood	N13-0119	S102	2	1.193	0.058	98.352
32	CC121	1/2a	II	04.07.17	Blood	N13-0119	S102	3	1.308	0.179	68.860
33	ST739	1/2a	II	03.08.17	Food	N11-2542	S30	1	1.431	0.117	90.971
33	ST739	1/2a	II	3/30/2017	Food	N11-2542	S30	2	1.369	0.045	112.830
33	ST739	1/2a	II	04.12.17	Food	N11-2542	S30	3	1.399	0.046	103.630
34	CC121	1/2a	II	03.08.17	Food	N13-0288	S74	1	1.663	0.421	105.765
34	CC121	1/2a	II	3/30/2017	Food	N13-0288	S74	2	1.261	0.082	103.929
34	CC121	1/2a	II	04.12.17	Food	N13-0288	S74	3	1.347	0.197	99.778
35	ST226	1/2a	II	03.08.17	Food	N13-2179	21903_6#61	1	1.606	0.170	81.111
35	ST226	1/2a	II	3/30/2017	Food	N13-2179	21903_6#61	2	1.625	0.179	103.393
35	ST226	1/2a	II	04.12.17	Food	N13-2179	21903_6#61	3	1.488	0.017	110.198
36	CC31	1/2a	II	03.08.17	Food	N13-0228	S70	1	1.489	0.194	75.219
36	CC31	1/2a	II	3/30/2017	Food	N13-0228	S70	2	1.822	0.160	115.925
36	CC31	1/2a	II	04.12.17	Food	N13-0228	S70	3	1.456	0.123	107.877
37	CC207	1/2a	II	03.08.17	Blood	N12-1107	S93	1	1.489	0.228	75.202
37	CC207	1/2a	II	3/30/2017	Blood	N12-1107	S93	2	1.895	0.091	120.526
37	CC207	1/2a	II	04.12.17	Blood	N12-1107	S93	3	1.297	0.114	96.074
38	CC415	1/2b	II	03.08.17	Blood	N13-0762	S133	1	1.536	0.116	77.593
38	CC415	1/2b	II	3/30/2017	Blood	N13-0762	S133	2	1.733	0.086	110.242
38	CC415	1/2b	II	04.12.17	Blood	N13-0762	S133	3	1.279	0.063	94.765
39	CC54	4b	I	03.08.17	Blood	N13-0177	S196	1	1.824	0.212	92.121
39	CC54	4b	I	3/30/2017	Blood	N13-0177	S196	2	1.665	0.042	105.937
39	CC54	4b	I	04.12.17	Blood	N13-0177	S196	3	1.355	0.047	100.346
40	CC224	1/2b	I	03.08.17	Blood	N12-1608	S122	1	1.806	0.088	91.212
40	CC224	1/2b	I	3/30/2017	Blood	N12-1608	S122	2	1.661	0.044	105.640
40	CC224	1/2b	I	04.12.17	Blood	N12-1608	S122	3	1.224	0.073	90.642

Raw plots

First let's check out the plots:

```
hemo %>%
  ggplot(aes(x=NENT.Nr., y=Mean.OD)) +
  theme(axis.text.x=element_text(angle=90,hjust=1))+
  geom_boxplot()
```

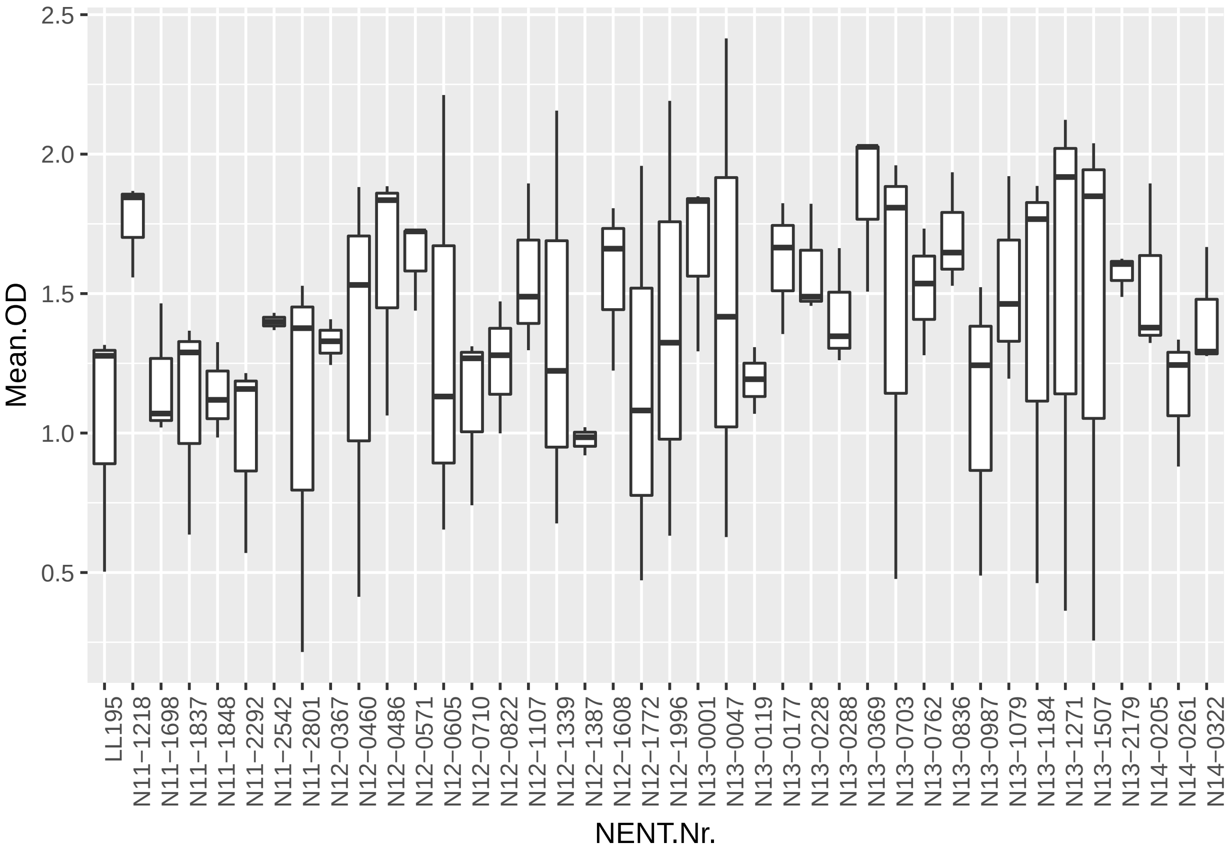


```
hemo %>%
  ggplot(aes(x=serotype, y=Mean.OD)) +
  geom_boxplot()
```

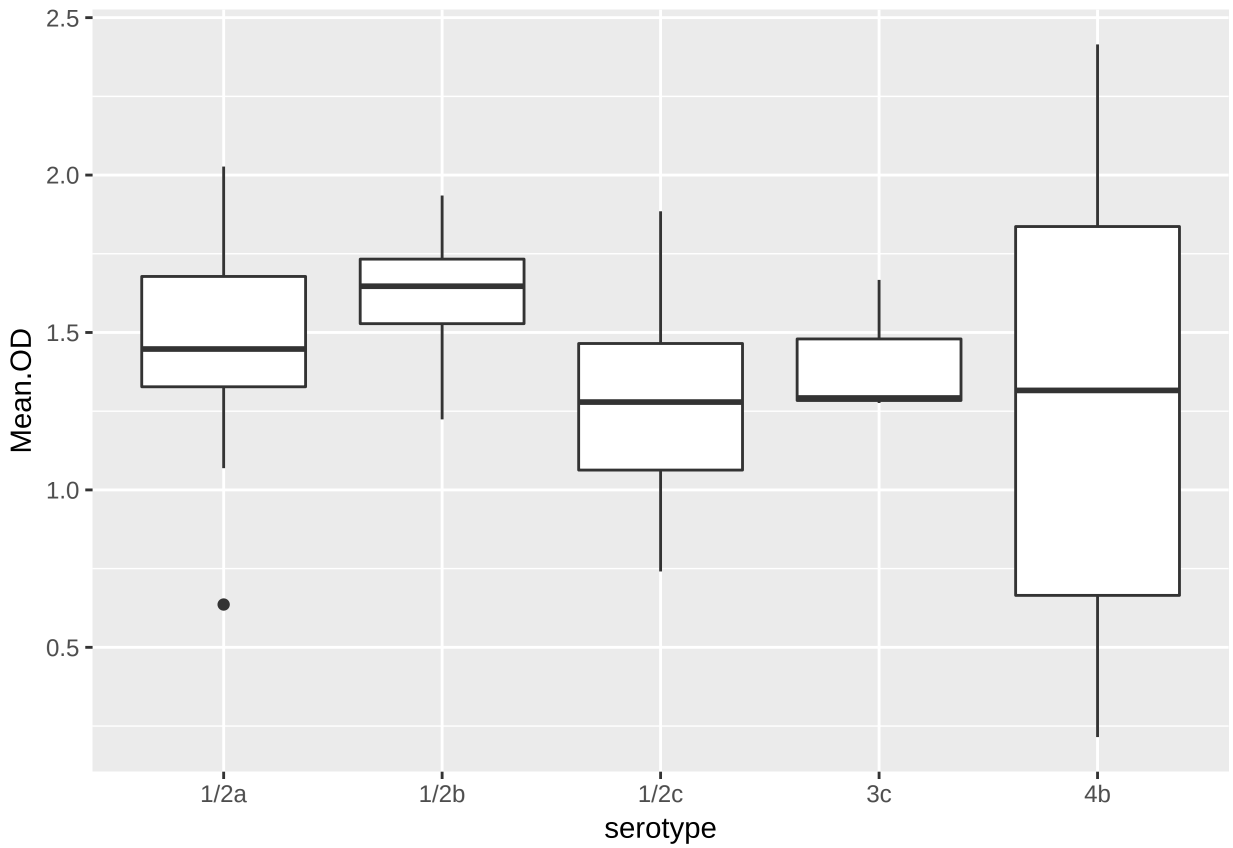


```r
hemo %>%
  ggplot(aes(x=clonal.complex, y=Mean.OD)) +
  geom_boxplot()
```

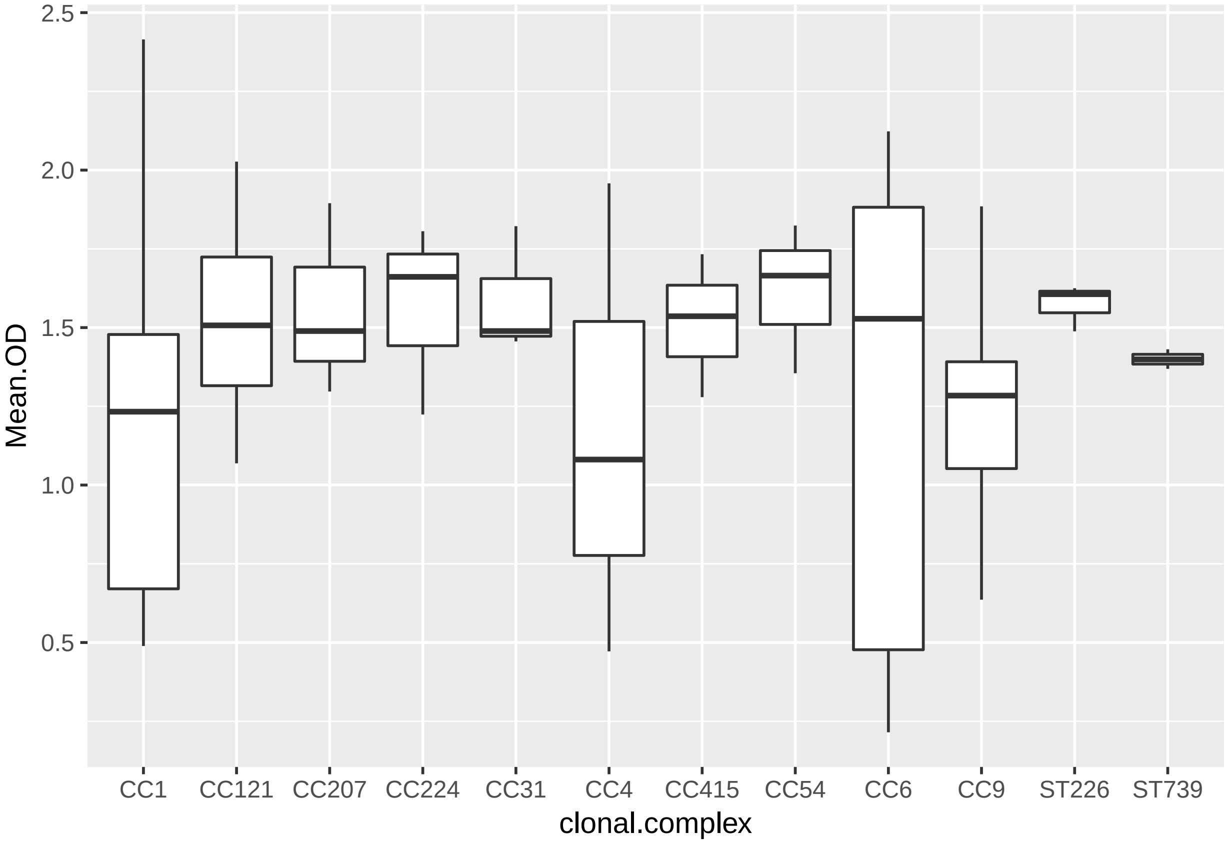


```
hemo %>%
  ggplot(aes(x=lineage, y=Mean.OD)) +
  geom_boxplot()
```

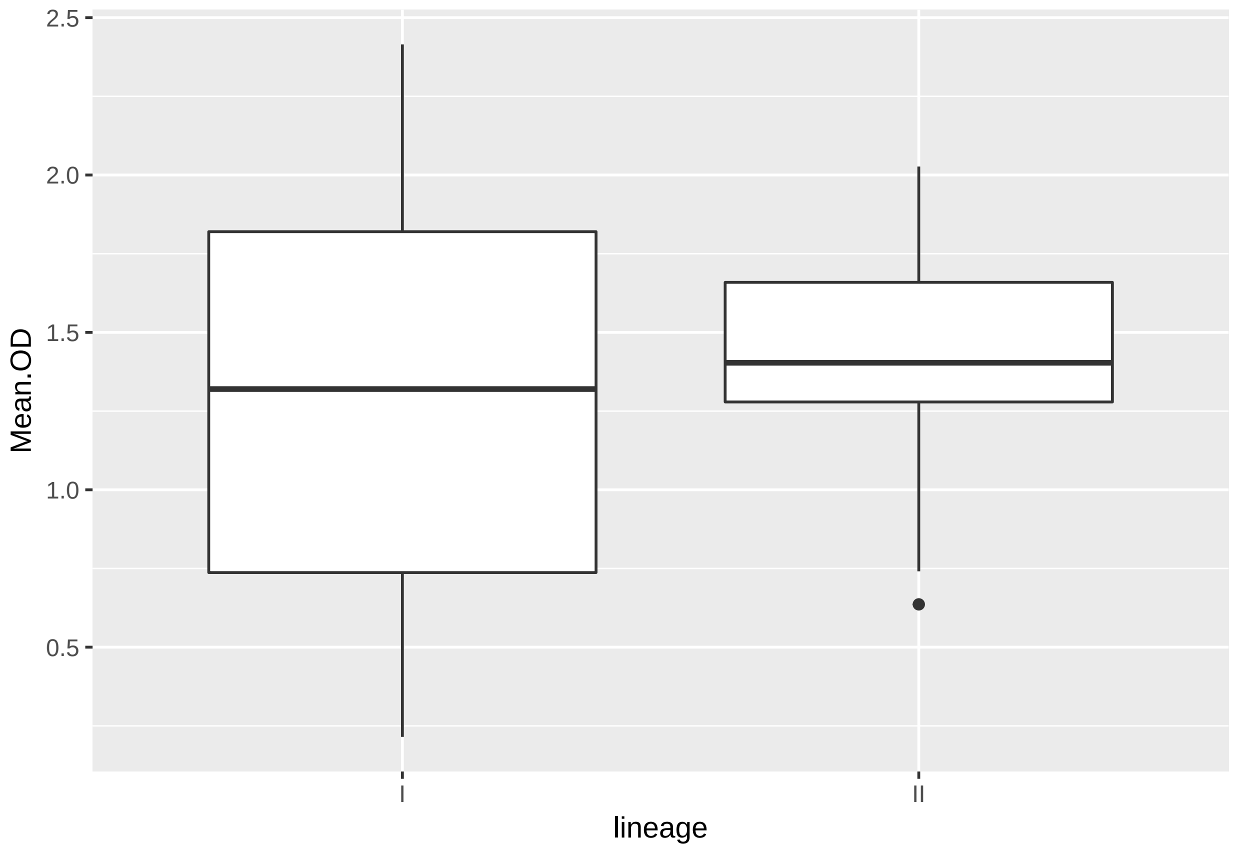


```r
hemo %>%
  ggplot(aes(x=source, y=Mean.OD)) +
  geom_boxplot()
```

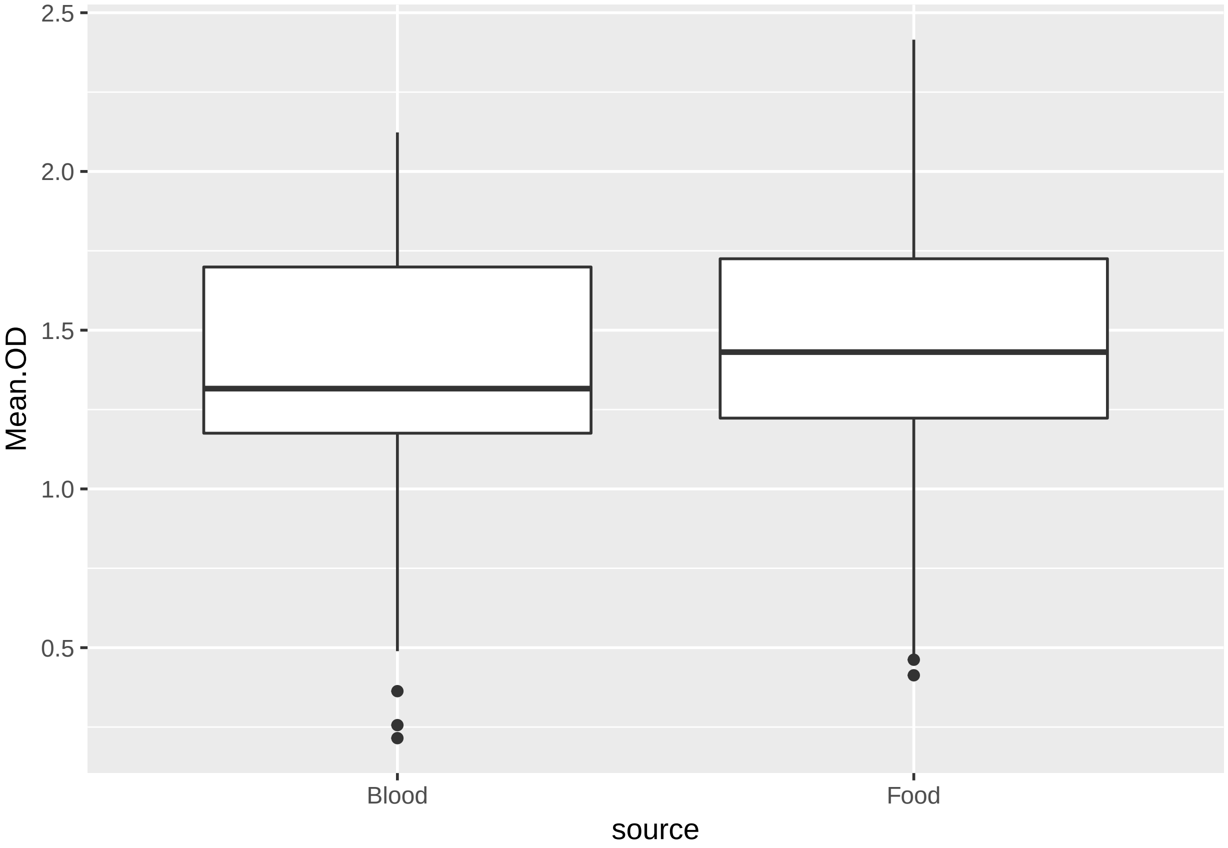

Fit the models:

The column ???date??? is a proxy for ???blood from person X" because we had new blood for every date. It is used as a blocking factor. Fitting the model on `Mean.OD` or `relative.to.PrfA.` *without* accounting for `Date` yields residuals which are not particularly normal. Once we add `Date` as a random factor, the residuals appear approximately normal. Since multiple strains are tested on each blood sample, doing it this way allows us to separate out what effect is due to the blood sample and what is due to the strain effect.

We therefore use `Mean.OD` as the response rather than `relative.to.PrfA.`, and control for the blood sample.

I've also chosen to use the False Discovery Rate method when adjusting for multiple comparisons – with this method, we're controlling the rate of false findings *among findings*. That is, we're adjusting so that 5% of the "significant" tests are false positives. Other methods control the proportion of false findings out of *all* tests, whether they have a significant result or not.

I only showed pairwise comparisons for the models which had a significant overall F-test.

Clonal Complex

```
m_cc <- lmer( Mean.OD ~ clonal.complex + (1|Date), data=hemo)
plot(predict(m_cc), resid(m_cc))
```

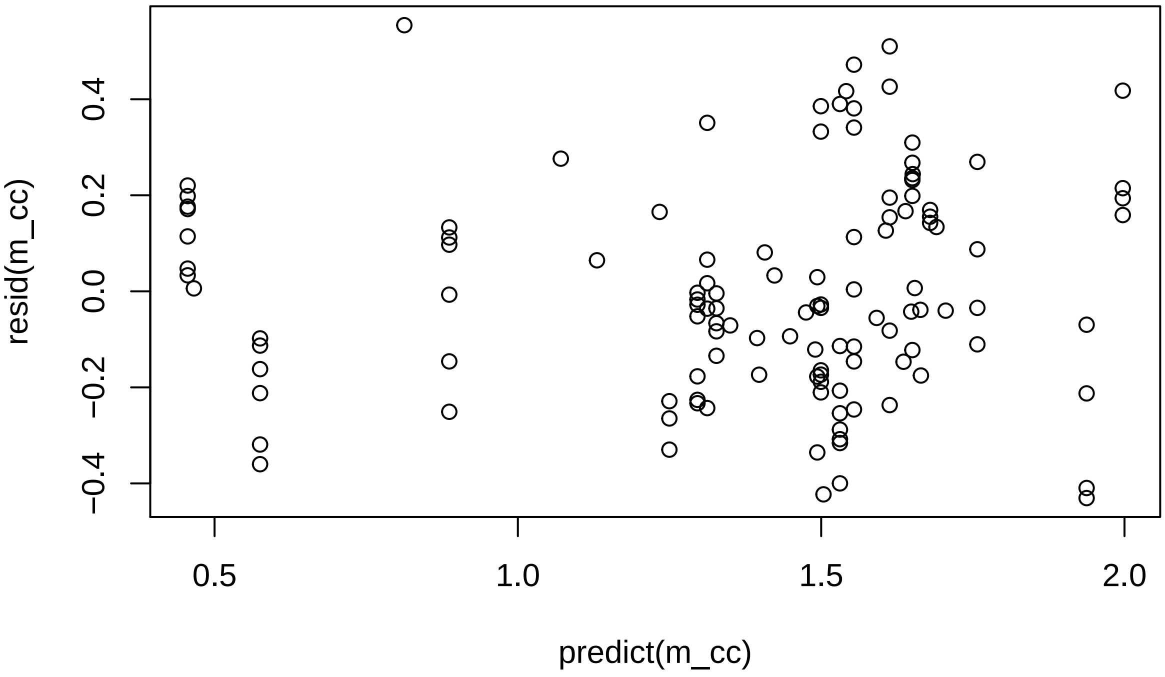


```r
qqnorm(resid(m_cc))
qqline(resid(m_cc))
```

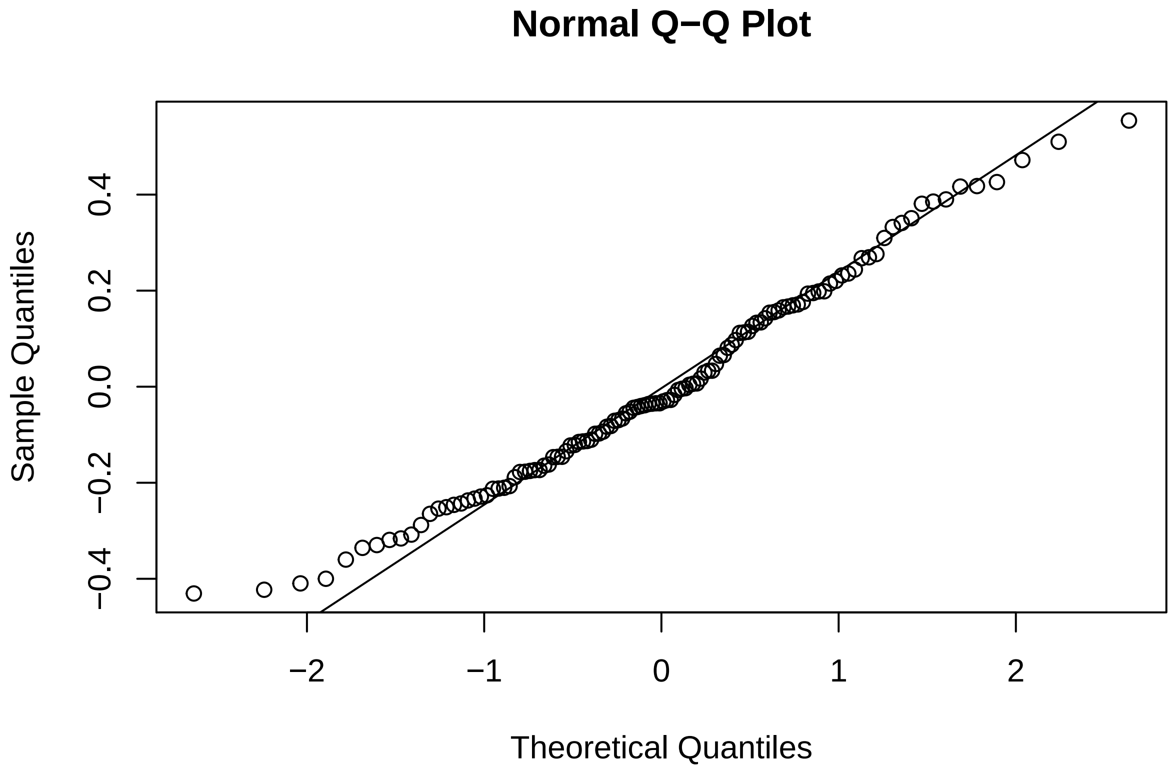

Diagnostics look OK.

```r
summary(m_cc)
```

```
## Linear mixed model fit by REML. t-tests use Satterthwaite's method [
## lmerModLmerTest]
```

```
## Formula: Mean.OD ~ clonal.complex + (1 | Date)
##    Data: hemo
##
## REML criterion at convergence: 51.8
##
## Scaled residuals:
##     Min      1Q  Median      3Q     Max
## -1.7781 -0.6886 -0.1343  0.6625  2.2885
##
## Random effects:
##  Groups   Name        Variance Std.Dev.
##  Date     (Intercept) 0.18650  0.4319
##  Residual             0.05864  0.2422
## Number of obs: 120, groups:  Date, 10
##
## Fixed effects:
##                      Estimate Std. Error        df t value Pr(>|t|)
## (Intercept)           1.40019    0.15588  13.41313   8.983 4.84e-07 ***
## clonal.complexCC121  -0.05962    0.11270 107.99161  -0.529   0.5979
## clonal.complexCC207   0.26394    0.17562 104.19193   1.503   0.1359
## clonal.complexCC224   0.26728    0.17562 104.19193   1.522   0.1311
## clonal.complexCC31    0.29261    0.17562 104.19193   1.666   0.0987 .
## clonal.complexCC4     0.01026    0.14999  99.39289   0.068   0.9456
## clonal.complexCC415   0.21961    0.17562 104.19193   1.250   0.2139
## clonal.complexCC54    0.31828    0.17562 104.19193   1.812   0.0728 .
## clonal.complexCC6     0.11935    0.07471  99.57177   1.598   0.1133
## clonal.complexCC9    -0.31760    0.12425 107.91823  -2.556   0.0120 *
## clonal.complexST226   0.27661    0.17562 104.19193   1.575   0.1183
## clonal.complexST739   0.10328    0.17562 104.19193   0.588   0.5578
## ---
## Signif. codes:  0 '***' 0.001 '**' 0.01 '*' 0.05 '.' 0.1 ' ' 1
##
## Correlation of Fixed Effects:
##             (Intr) c.CC12 c.CC20 c.CC22 c.CC31 cl.CC4 c.CC41 c.CC54 cl.CC6
## clnl.cCC121 -0.420
## clnl.cCC207 -0.259  0.495
## clnl.cCC224 -0.259  0.495  0.366
## clnl.cmCC31 -0.259  0.495  0.366  0.366
## clnl.cmpCC4 -0.075  0.072  0.054  0.054  0.054
## clnl.cCC415 -0.259  0.495  0.366  0.366  0.366  0.054
## clnl.cmCC54 -0.259  0.495  0.366  0.366  0.366  0.054  0.366
## clnl.cmpCC6 -0.202  0.251  0.198  0.198  0.198  0.246  0.198  0.198
## clnl.cmpCC9 -0.407  0.766  0.402  0.402  0.402  0.064  0.402  0.402  0.221
## clnl.cST226 -0.259  0.495  0.366  0.366  0.366  0.054  0.366  0.366  0.198
## clnl.cST739 -0.259  0.495  0.366  0.366  0.366  0.054  0.366  0.366  0.198
##             cl.CC9 c.ST22
## clnl.cCC121
## clnl.cCC207
## clnl.cCC224
## clnl.cmCC31
## clnl.cmpCC4
## clnl.cCC415
## clnl.cmCC54
## clnl.cmpCC6
```

```
## clnl.cmpCC9
## clnl.cST226  0.402
## clnl.cST739  0.402  0.366
```

```
anova(m_cc)
```

```
## Type III Analysis of Variance Table with Satterthwaite's method
##                 Sum Sq Mean Sq NumDF  DenDF F value   Pr(>F)
## clonal.complex 1.7728 0.16116    11 100.97  2.7483 0.003745 **
## ---
## Signif. codes:  0 '***' 0.001 '**' 0.01 '*' 0.05 '.' 0.1 ' ' 1
```

This tells us that `clonal.complex` does account for a significant amount of the variability in `Mean.OD`.

```
cld_cc <- lsmeans(m_cc,pairwise~clonal.complex) %>%
  cld(adjust="fdr")
cld_cc %>%
  kable("latex") %>%
  kable_styling(bootstrap_options=c("striped",
                                    "hover",
                                    "condensed",
                                    "responsive"))
```

	clonal.complex	lsmean	SE	df	lower.CL	upper.CL	.group
10	CC9	1.082587	0.1547885	13.16016	0.5492377	1.615935	1
2	CC121	1.340567	0.1491371	11.58582	0.8266913	1.854444	2
1	CC1	1.400188	0.1558789	13.41313	0.8630822	1.937294	12
6	CC4	1.410448	0.2080539	37.18913	0.6935652	2.127332	12
12	ST739	1.503464	0.2023778	35.33757	0.8061386	2.200790	12
9	CC6	1.519543	0.1586326	14.24914	0.9729483	2.066137	2
7	CC415	1.619798	0.2023778	35.33757	0.9224719	2.317123	2
3	CC207	1.664131	0.2023778	35.33757	0.9668053	2.361456	2
4	CC224	1.667464	0.2023778	35.33757	0.9701386	2.364790	2
11	ST226	1.676797	0.2023778	35.33757	0.9794719	2.374123	2
5	CC31	1.692797	0.2023778	35.33757	0.9954719	2.390123	2
8	CC54	1.718464	0.2023778	35.33757	1.0211386	2.415790	2

And here we have the pairwise comparisons of clonal complexes.

Plot

```
cld_cc %>%
  ggplot(aes(x=clonal.complex,
             y=lsmean)) +
  ylim(0,NA) +
  geom_point(aes(color=clonal.complex)) +
  geom_errorbar(aes(ymin=lower.CL,
                    ymax=upper.CL,
                    color=clonal.complex)) +
  geom_text(aes(label=.group,y=upper.CL),vjust=-1)
```

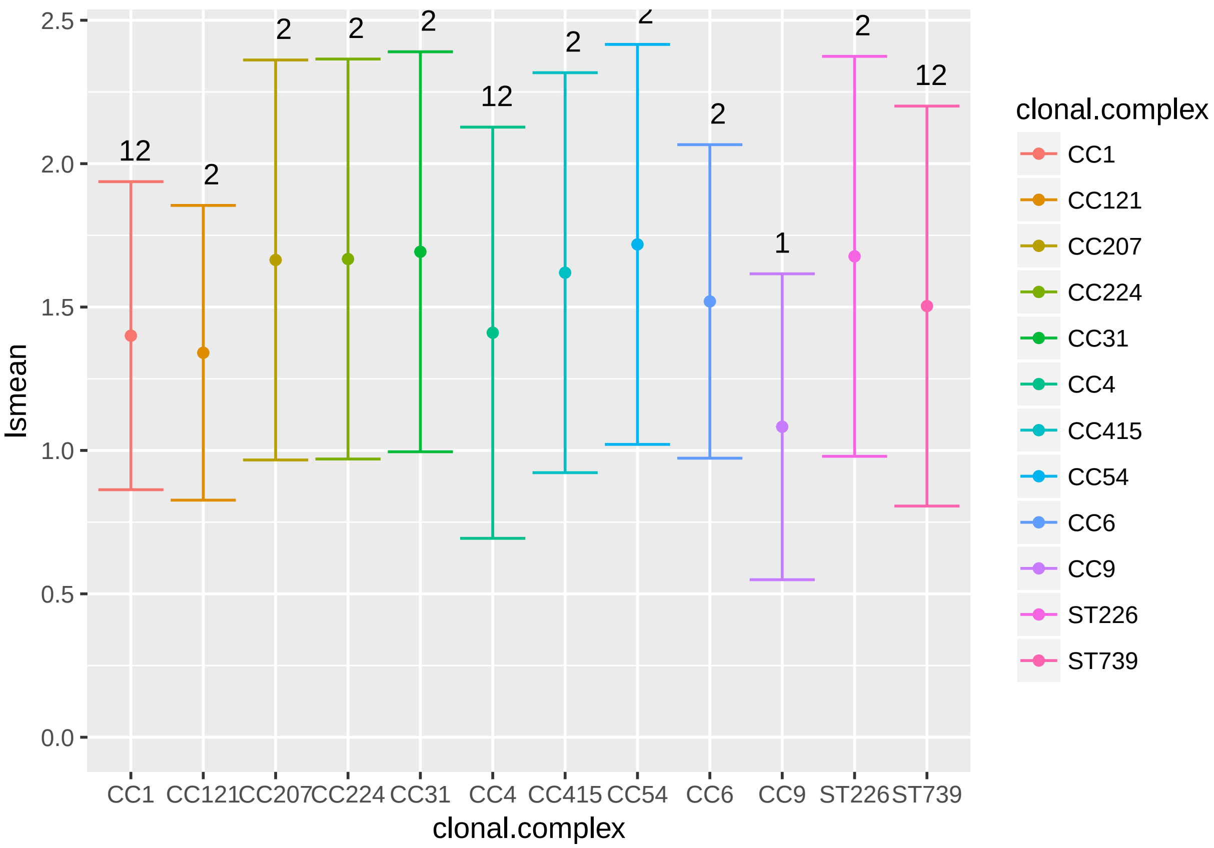

Serotype

```
m_s <- lmer( Mean.OD ~ serotype + (1|Date), data=hemo)
plot(predict(m_s), resid(m_s))
```

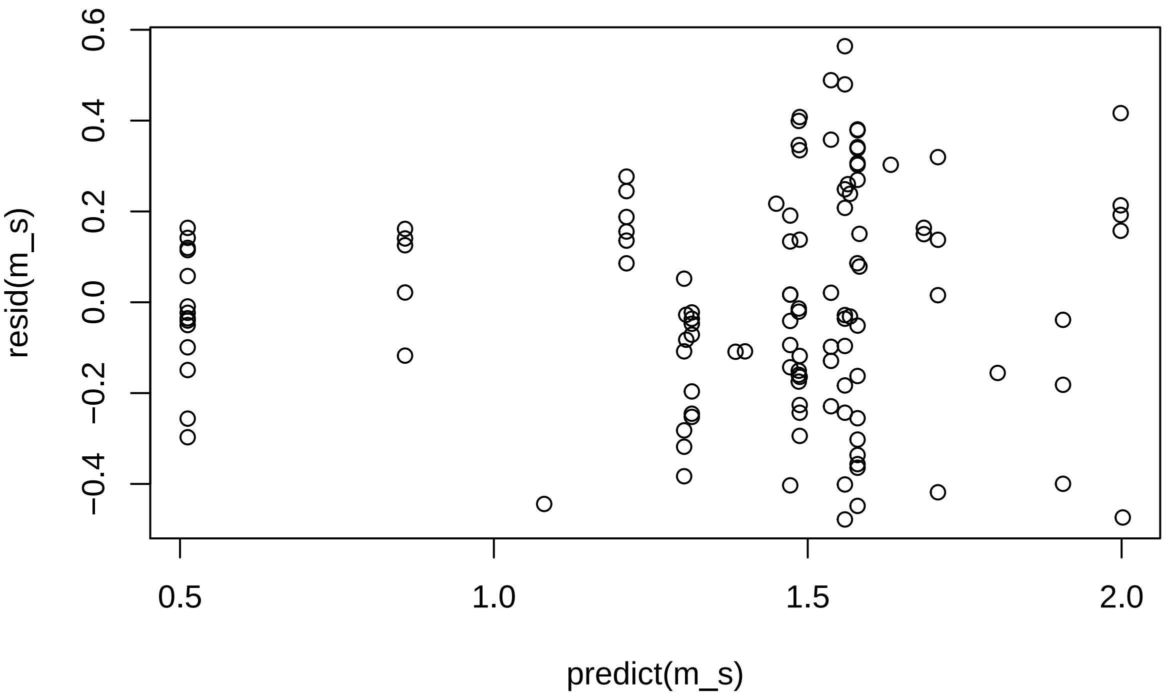


```r
qqnorm(resid(m_s))
qqline(resid(m_s))
```

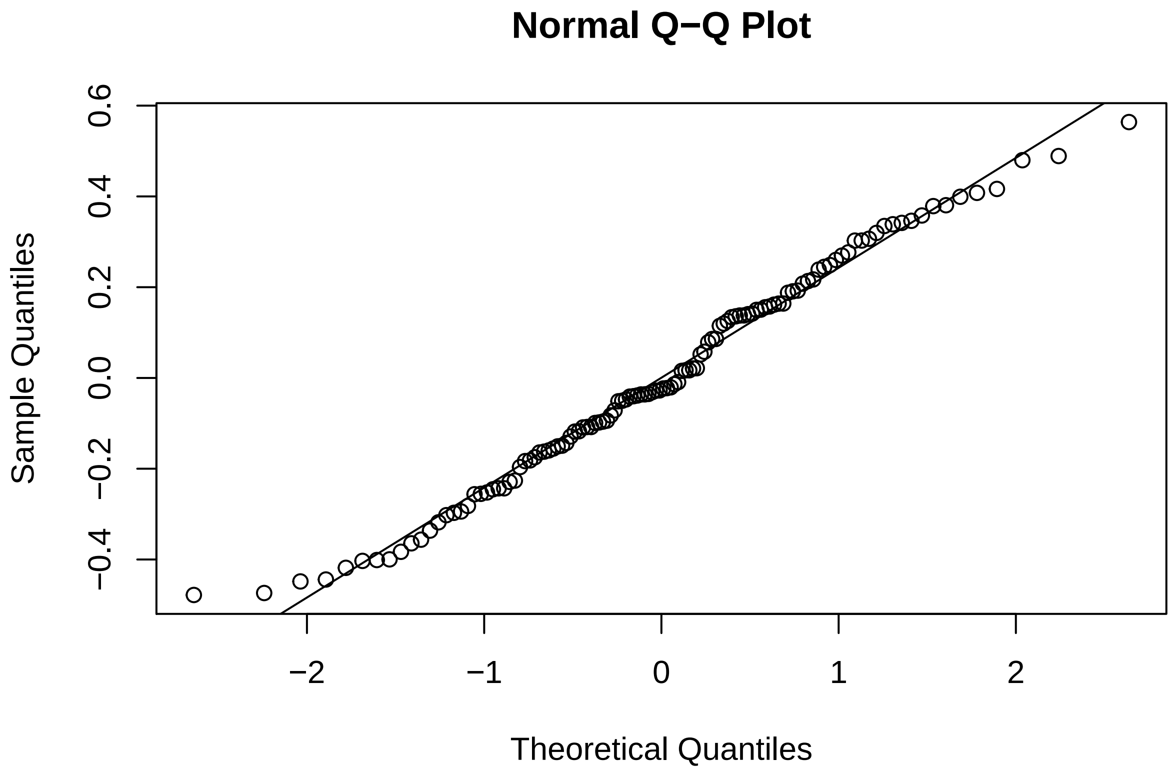

Diagnostics look OK.

```r
summary(m_s)
```

```
## Linear mixed model fit by REML. t-tests use Satterthwaite's method [
## lmerModLmerTest]
```

```
## Formula: Mean.OD ~ serotype + (1 | Date)
##    Data: hemo
##
## REML criterion at convergence: 55.8
##
## Scaled residuals:
##      Min       1Q   Median       3Q      Max
## -1.87064 -0.63696 -0.09906  0.64096  2.20555
##
## Random effects:
##  Groups   Name        Variance Std.Dev.
##  Date     (Intercept) 0.17294  0.4159
##  Residual             0.06535  0.2556
## Number of obs: 120, groups:  Date, 10
##
## Fixed effects:
##               Estimate Std. Error        df t value Pr(>|t|)
## (Intercept)    1.37770    0.14166  11.01105   9.725 9.68e-07 ***
## serotype1/2b   0.09519    0.09558 106.14280   0.996   0.3215
## serotype1/2c  -0.22167    0.08601 110.62016  -2.577   0.0113 *
## serotype3c    -0.08711    0.15548 106.24521  -0.560   0.5765
## serotype4b     0.09184    0.09394 114.95848   0.978   0.3303
## ---
## Signif. codes:  0 '***' 0.001 '**' 0.01 '*' 0.05 '.' 0.1 ' ' 1
##
## Correlation of Fixed Effects:
##             (Intr) srtyp1/2b srtyp1/2c srtyp3
## serotyp1/2b -0.136
## serotyp1/2c -0.181  0.189
## serotype3c  -0.067  0.115     0.118
## serotype4b  -0.297  0.228     0.166     0.078
```

```r
anova(m_s)
```

```
## Type III Analysis of Variance Table with Satterthwaite's method
##           Sum Sq Mean Sq NumDF  DenDF F value  Pr(>F)
## serotype 0.68157 0.17039     4 110.61  2.6075 0.03949 *
## ---
## Signif. codes:  0 '***' 0.001 '**' 0.01 '*' 0.05 '.' 0.1 ' ' 1
```

This says that `serotype` does explain a significant amount of the variability in `Mean.OD`

```r
cld_s <- lsmeans(m_s,pairwise~serotype) %>%
  cld(adjust="fdr")
cld_s %>%
  kable("latex") %>%
  kable_styling(bootstrap_options=c("striped",
                                    "hover",
                                    "condensed",
                                    "responsive"))
```

	serotype	lsmean	SE	df	lower.CL	upper.CL	.group
3	1/2c	1.156032	0.1518101	14.14188	0.6846345	1.627430	1
4	3c	1.290592	0.2032196	40.70598	0.6595586	1.921625	12
1	1/2a	1.377704	0.1416620	11.01105	0.9378185	1.817590	2
5	4b	1.469549	0.1448644	11.75824	1.0197195	1.919379	2
2	1/2b	1.472899	0.1597180	17.58200	0.9769455	1.968852	2

Here we have the pairwise differences in serotypes.

Plot

```
cld_s %>%
  ggplot(aes(x=serotype,
             y=lsmean)) +
  ylim(0,NA) +
  geom_point(aes(color=serotype)) +
  geom_errorbar(aes(ymin=lower.CL,
                    ymax=upper.CL,
                    color=serotype)) +
  geom_text(aes(label=.group,y=upper.CL),vjust=-1)
```

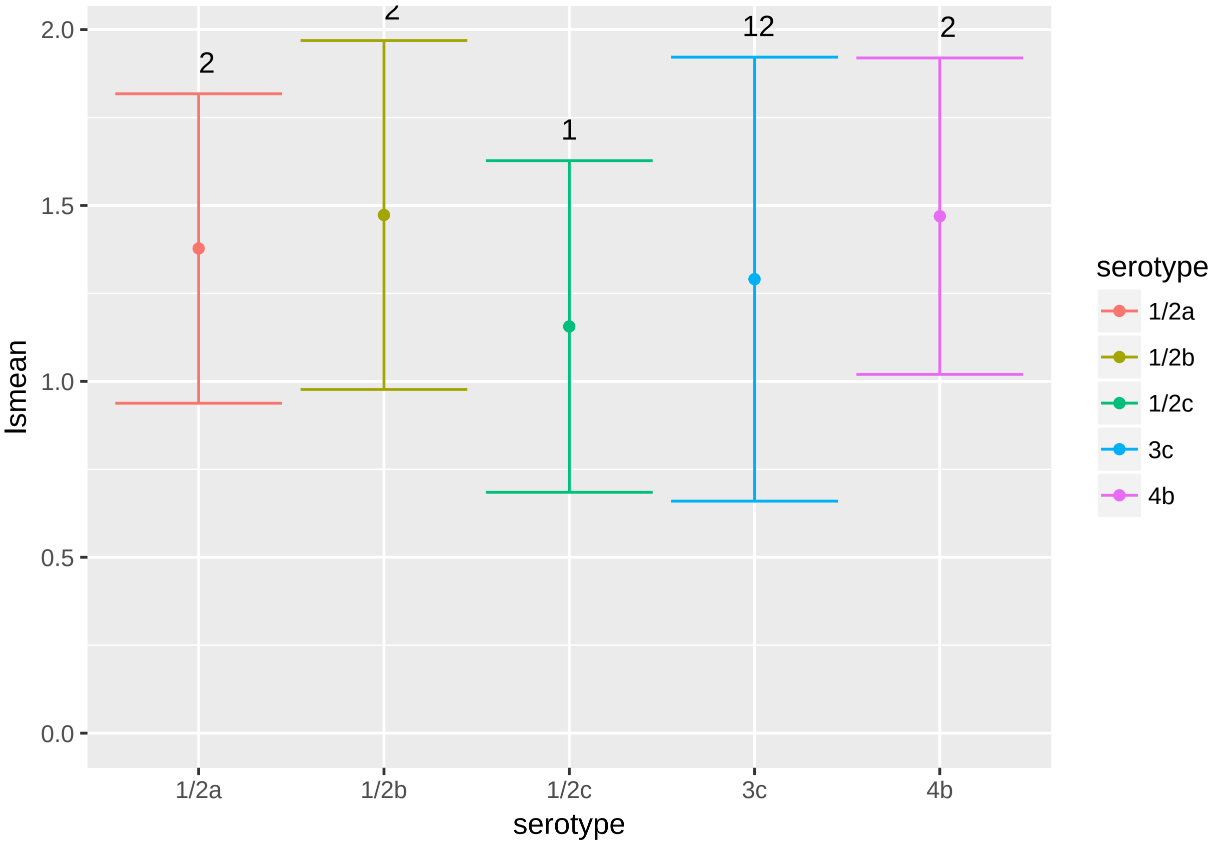

Lineage

```
m_l <- lmer( Mean.OD ~ lineage + (1|Date), data=hemo)
plot(predict(m_l), resid(m_l))
```

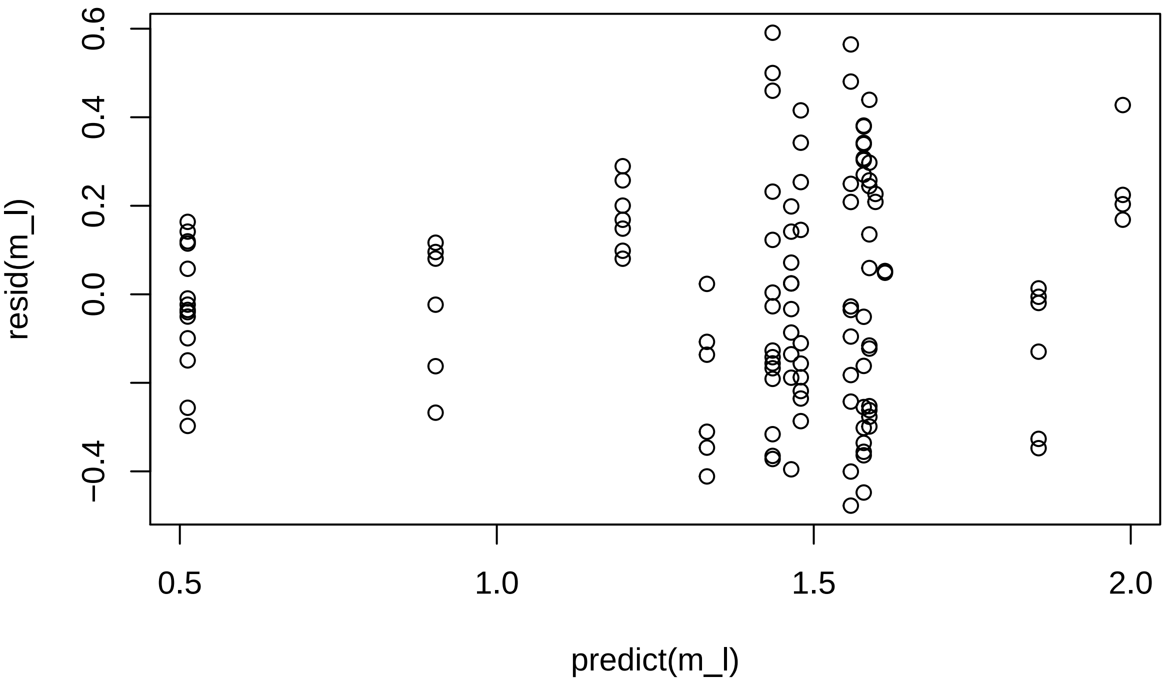


```
qqnorm(resid(m_l))
qqline(resid(m_l))
```

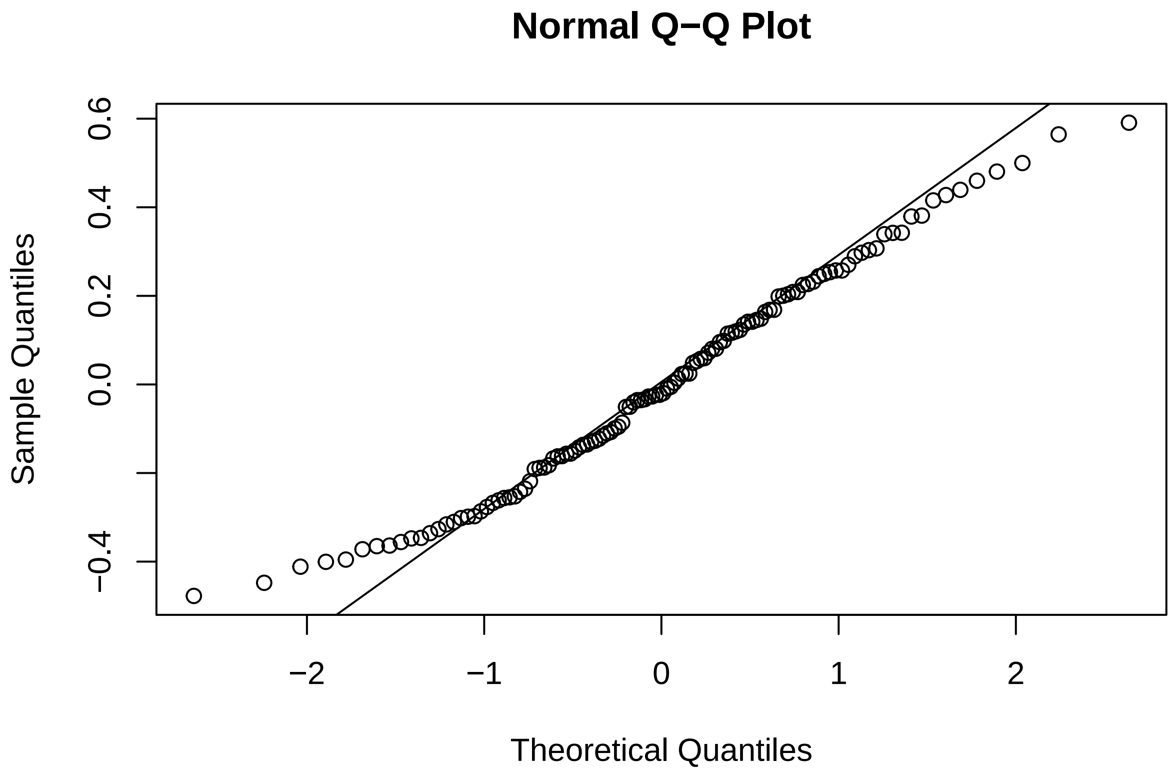

Diag-

nostics look OK.

```r
summary(m_l)
```

```
## Linear mixed model fit by REML. t-tests use Satterthwaite's method [
## lmerModLmerTest]
## Formula: Mean.OD ~ lineage + (1 | Date)
##    Data: hemo
##
## REML criterion at convergence: 55.9
##
## Scaled residuals:
##      Min       1Q   Median       3Q      Max
## -1.82894 -0.71924 -0.08231  0.76234  2.26353
##
## Random effects:
##  Groups   Name        Variance Std.Dev.
##  Date     (Intercept) 0.17560  0.4190
##  Residual             0.06815  0.2611
## Number of obs: 120, groups:  Date, 10
##
## Fixed effects:
##             Estimate Std. Error       df t value Pr(>|t|)
## (Intercept)   1.4503     0.1433  11.1436  10.118  5.9e-07 ***
## lineageII    -0.1328     0.0843 117.8207  -1.576    0.118
## ---
## Signif. codes:  0 '***' 0.001 '**' 0.01 '*' 0.05 '.' 0.1 ' ' 1
##
## Correlation of Fixed Effects:
##           (Intr)
## lineageII -0.341
```

```r
anova(m_l)
```

```
## Type III Analysis of Variance Table with Satterthwaite's method
##          Sum Sq Mean Sq NumDF  DenDF F value Pr(>F)
## lineage 0.16923 0.16923     1 117.82  2.4832 0.1178
```

This tells us that `lineage` does not explain a significant amount of the variability in `Mean.OD`.

Plot

```r
lsmeans(m_l,pairwise~lineage) %>%
  cld() %>%
  ggplot(aes(x=lineage,
             y=lsmean)) +
  ylim(0,NA) +
  geom_point(aes(color=lineage)) +
  geom_errorbar(aes(ymin=lower.CL,
                    ymax=upper.CL,
                    color=lineage))
```

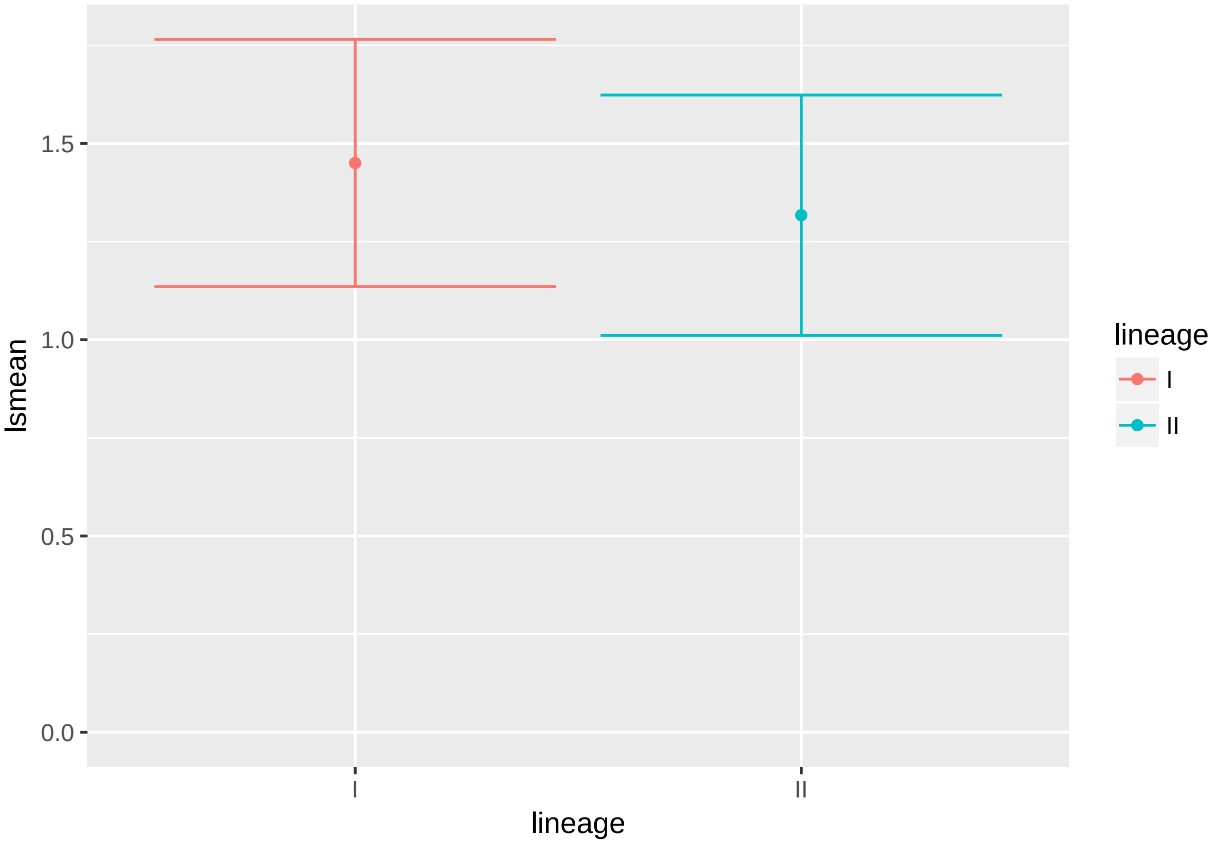

Source

Here we test whether `source`explains a significant amount of the variability in hemolysis? For the BHI/salt data I looked at whether it explained more than just CC alone, which is a slightly different question. Below ignores CC and just tests whether `source` by itself explains a significant amount of the variability. For the other question, you would do a nested F-test with `Mean.OD ~ clonal.complex` tested against `Mean.OD ~ clonal.complex + source` using the `anova()` function.

```
m_source <- lmer( Mean.OD ~ source + (1|Date), data=hemo)
plot(predict(m_source), resid(m_source))
```

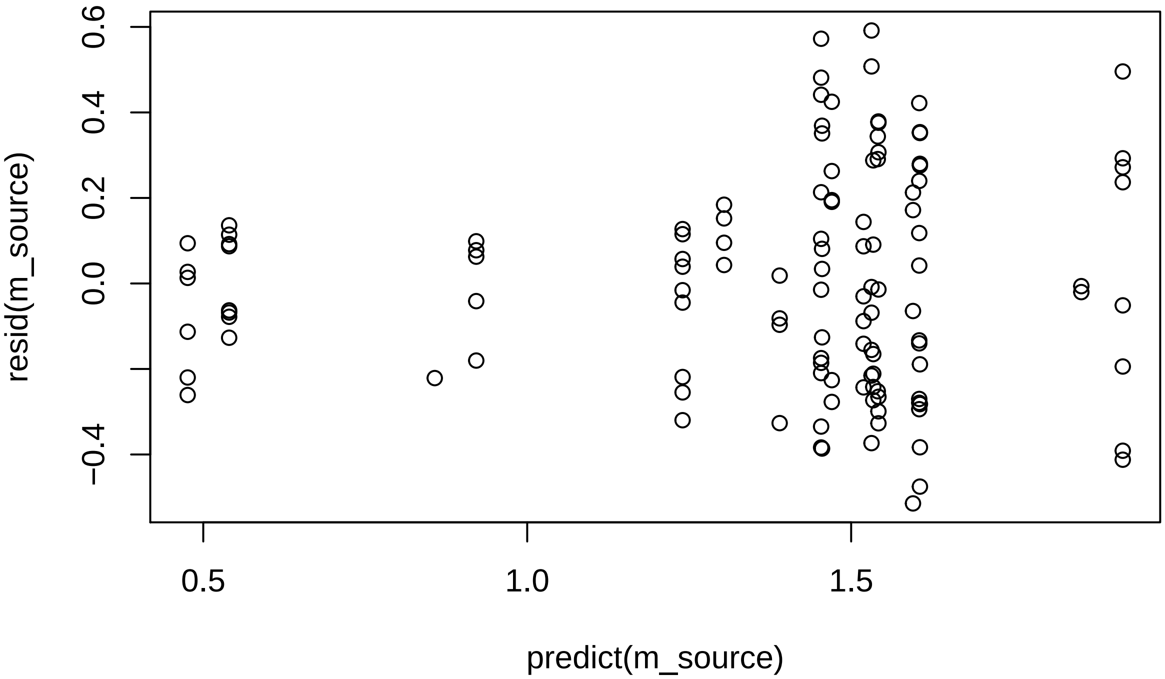


```r
qqnorm(resid(m_source))
qqline(resid(m_source))
```

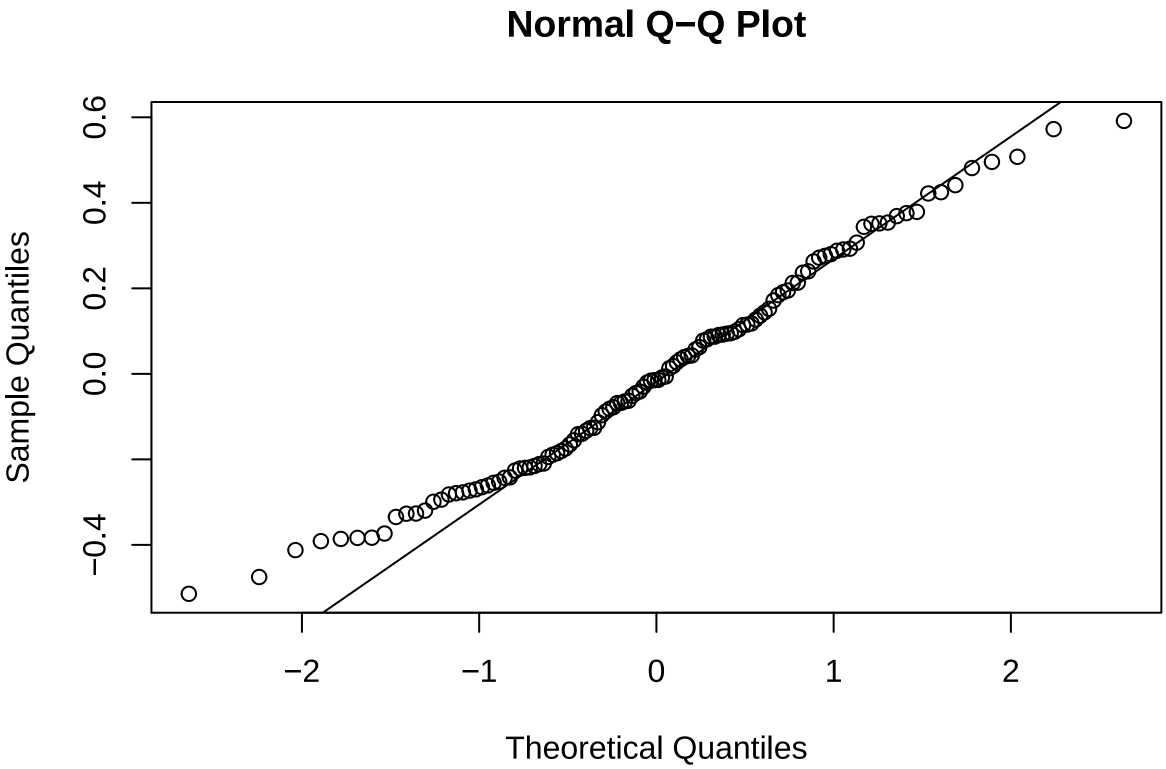

Diagnostics look OK.

```r
summary(m_source)
```

```
## Linear mixed model fit by REML. t-tests use Satterthwaite's method [
## lmerModLmerTest]
```

```
## Formula: Mean.OD ~ source + (1 | Date)
##    Data: hemo
##
## REML criterion at convergence: 57.7
##
## Scaled residuals:
##      Min       1Q   Median       3Q      Max
## -1.95654 -0.80713 -0.05476  0.66449  2.25000
##
## Random effects:
##  Groups   Name        Variance Std.Dev.
##  Date     (Intercept) 0.16215  0.4027
##  Residual             0.06913  0.2629
## Number of obs: 120, groups:  Date, 10
##
## Fixed effects:
##              Estimate Std. Error        df t value Pr(>|t|)
## (Intercept)   1.33579    0.13319   9.95288  10.029 1.61e-06 ***
## sourceFood    0.06402    0.05135 109.97274   1.247    0.215
## ---
## Signif. codes:  0 '***' 0.001 '**' 0.01 '*' 0.05 '.' 0.1 ' ' 1
##
## Correlation of Fixed Effects:
##            (Intr)
## sourceFood -0.227
```

```r
anova(m_source)
```

```
## Type III Analysis of Variance Table with Satterthwaite's method
##         Sum Sq Mean Sq NumDF  DenDF F value Pr(>F)
## source 0.10745 0.10745     1 109.97  1.5543 0.2151
```

This tells us that `source` does not explain a significant amount of the variability in `Mean.OD`. Also, peculiarly, the blood isolates have the lowest `Mean.OD` value of the three sources.

Plot

```r
lsmeans(m_source,pairwise~source) %>%
  cld() %>%
  ggplot(aes(x=source,
             y=lsmean)) +
  ylim(0,2.2) +
  geom_point(aes(color=source)) +
  geom_errorbar(aes(ymin=lower.CL,
                    ymax=upper.CL,
                    color=source))
```

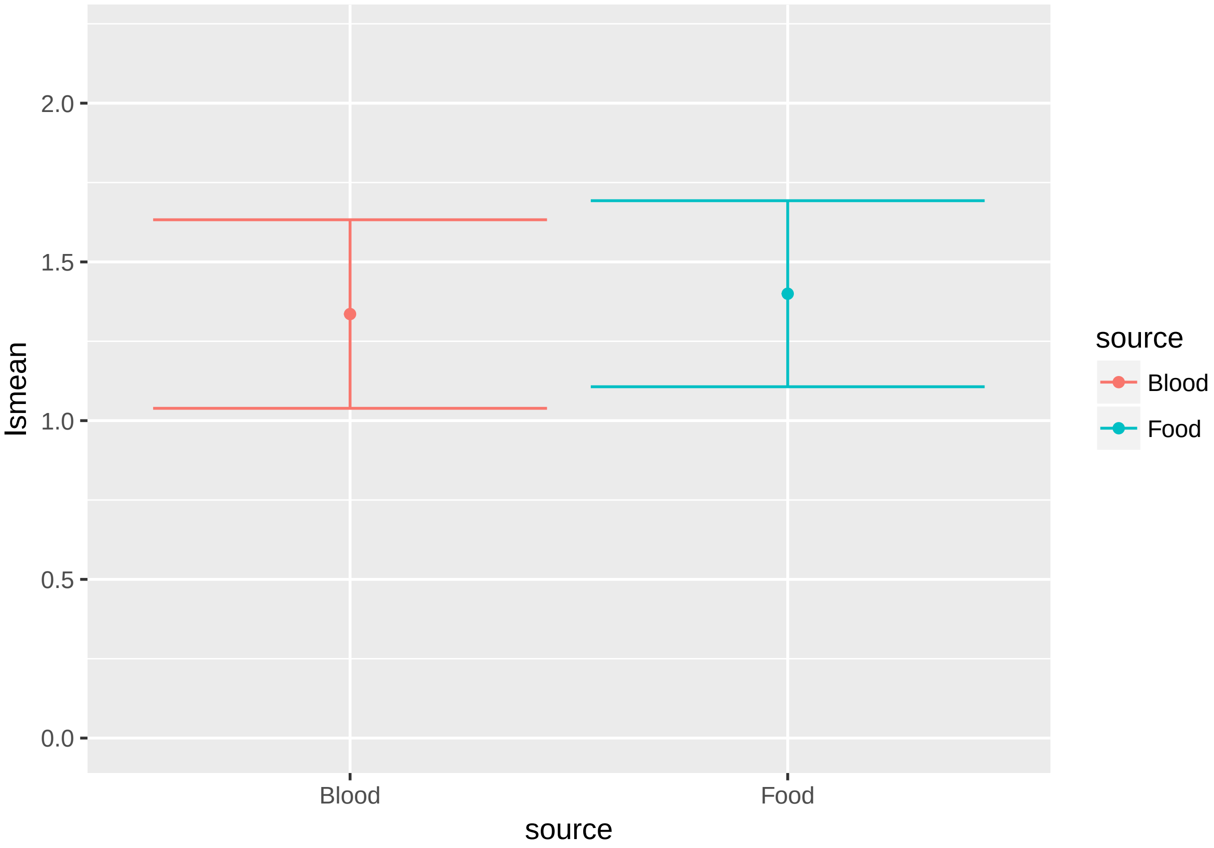
2.0
1.5
1.0
0.5
0.0
lsmean
Blood
Food
source
source
Blood
Food
